# An investigation of the allylation cascade reactions of substituted indigos†

**DOI:** 10.1039/d3ra00481c

**Published:** 2023-02-06

**Authors:** Matthew J. Perry, Anthony C. Willis, John B. Bremner, Paul A. Keller

**Affiliations:** a School of Chemistry and Molecular Bioscience, Molecular Horizons, and Illawarra Health and Medical Research Institute, University of Wollongong Wollongong NSW 2522 Australia keller@uow.edu.au; b Research School of Chemistry, Australian National University Canberra ACT 2601 Australia

## Abstract

In a continuation of the exploration of indigo cascade reactions, a series of –OMe, –Ph, –Br and –NO_2_ substituted indigos 1a–i were synthesised to probe electronic effects upon the outcome of allylation cascade reactions. When indigos 1a–i in the presence of base were reacted with allyl bromide, spiroindolinepyridoindolones 17–25 (36–75%) were obtained as the major products in each case, marking a shift in outcome relative to that previously reported for unsubstituted indigo. In electron-rich derivatives (–OMe, –Ph), *C*-allylspiroindolinepyridoindolediones 26–29 (3–11%) were also isolated, which are most likely formed *via* a Claisen rearrangement of the respective spiroindolinepyridoindolones 18–21. Additionally, the isolation of diallylbiindolone 16, oxazinobiindole 30 and *N*,*N′*-diallyl-3,3′-bis(allyloxy)biindole 31 each represented novel polyheterocyclic derivatives, providing intriguing new mechanistic insights, reaction pathways and in the case of 30 the first common heterocyclic skeletal outcome shared in both allylation and propargylation cascade reactions of indigo.

## Abstract 2

In a continuation of the exploration of indigo cascade reactions, a series of –OMe, –Ph, –Br and –NO_2_ substituted indigos 1a–i were synthesised to probe electronic effects upon the outcome of allylation cascade reactions.

## Graphical abstract

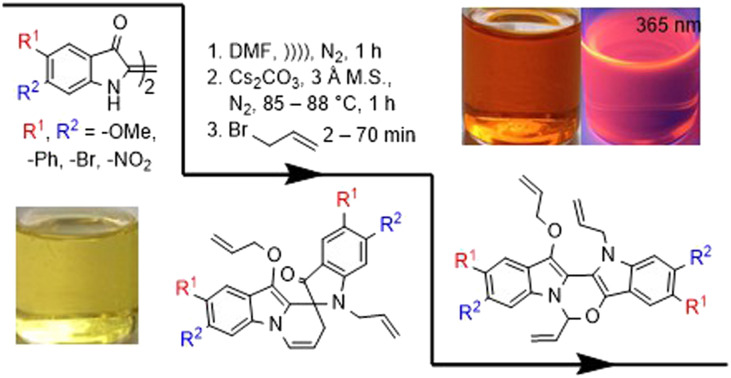

## Introduction

Indigo 1 and its derivatives have been the focus of intensive study in recent years due to their presence in biologically active natural products^1–3^ and unique photophysical^4^ and semiconducting properties.^5,6^ The industrial-scale synthesis and high degree of functionality of indigo 1 also make it an ideal target for use as an advanced synthetic building block for the synthesis of more complex heterocycles, as exemplified by the cascade reactions of indigo 1. Under basic conditions, one-pot reactions of indigo 1 with aziridine,^7^ oxirane,^7^ allyl^8,9^ and propargyl^10,11^ halides have provided access to diverse polyheterocyclic architectures with a range of anticancer, antitubercular and antimalarial activities, and materials with high fluorescence quantum yields and ready access to triplet states.

**Scheme 1:**
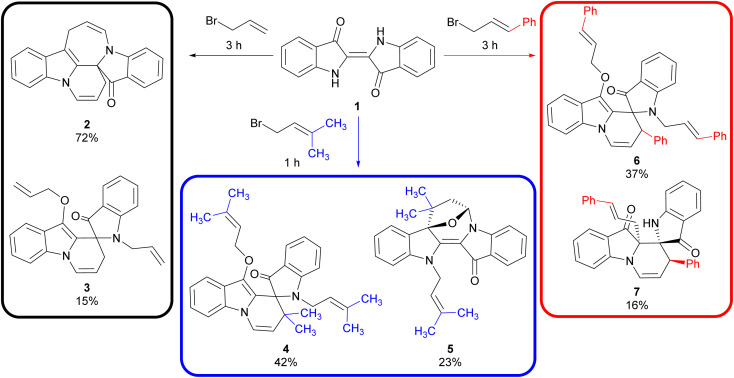
The cascade reactions of indigo 1 with allyl bromide, 1-bromo-3-methyl-2-butene and cinnamyl bromide electrophiles. Reaction conditions: (i) DMF, N_2_, 30 min; (ii) Cs_2_CO_3_, 4 Å M.S., N_2_, 85–88 °C, 30 min; (iii) electrophile, 1–3 h. Note – the relative stereochemistry is shown for compounds 5 and 7.

Due to the complex multi-step mechanisms involved in the cascade reactions of indigo 1 and the numerous mechanistic branchpoints available, the ability to accurately predict the outcome of any given cascade reaction has not yet been attained. Minor changes in the cascade reaction conditions were observed to lead to significantly altered outcomes, particularly when substituted electrophiles were employed.^7–11^ This was evident in the allylation cascade reactions, where the use of allyl bromide led to the production of pyridoindoloazepinoindolone 2 and spiroindolinepyridoindolone 3 in 72% and 15% yield, respectively (Scheme 1). In contrast, the use of 1-bromo-3-methyl-2-butene led to the synthesis of spiroindolinepyridoindolone analogue 4 (42%) as the major product along with epoxyazepinodiindolone 5 (23%, Scheme 1).^9^ Likewise, the use of cinnamyl bromide again produced the equivalent spiroindolinepyridoindolone 6 as the major outcome (37%) in addition to *C*-cinnamylated spiroindolinepyridoindoledione 7 (16%), highlighting the variability of outcome when substituted electrophiles are utilised (Scheme 1).^9^

Although intuitive mechanisms have been proposed to account for the outcomes observed in the cascade reactions of indigo 1, the isolation of key intermediates and direct evidence to substantiate the reported mechanisms is required. A deeper understanding of these mechanisms could also enable accurate prediction of cascade reaction outcomes and the ability to tune the reaction conditions to favour the formation of specific heterocyclic targets. To this point, all investigations into the cascade reactions of indigo 1 have focused upon changing the nature of the electrophile with no equivalent studies associated with the use of functionalised indigos. The use of indigos substituted with a spectrum of EDGs and EWGs could provide a more thorough understanding of how electronic factors affect key mechanistic branchpoints and provide a further insight into these cascade reactions. Reported herein is the synthesis of a range of substituted indigos, their evaluation in allylation cascade reactions, and the mechanistic implications arising from these outcomes.

## Results and discussion

### The synthesis of substituted indigos

**Fig. 1:**
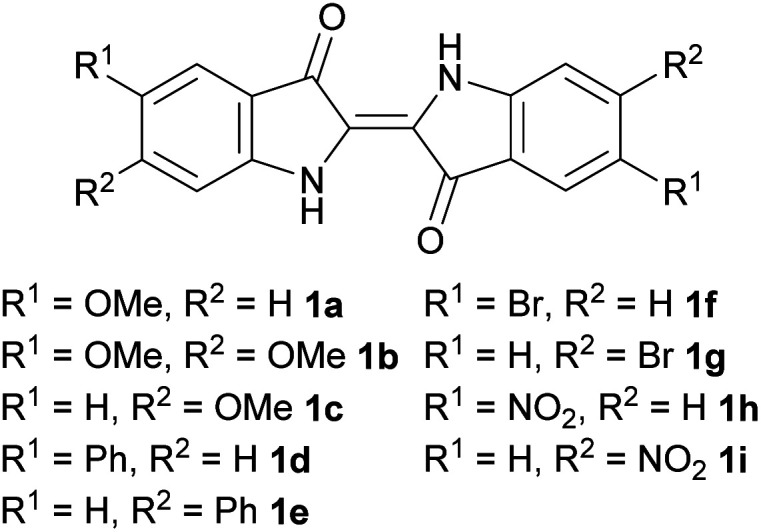
Substituted indigos targeted for investigation in allylation cascade reactions.

To systematically investigate the use of substituted indigos in the allylation cascade reaction, indigos substituted in the 5,5′- and 6,6′-positions with –OMe, –Br, –Ph and –NO_2_ functionalities (Fig. 1) were targeted. The one-pot Baeyer–Drewson indigo synthesis was used due to its procedural simplicity.^12^ Bromo- and methoxy-substituted 2-nitrobenzaldehyde starting materials were commercially available, while phenyl-substituted derivatives were prepared *via* a Suzuki coupling of PhB(OH)_2_ with the respective bromo-derivatives (see ESI,† Section S1.1). The methoxy-, bromo- and phenyl-substituted 2-nitrobenzaldehydes 8a–g were dissolved in acetone, stirred overnight with 1 M NaOH, the precipitate filtered, rinsed with organic solvents and where necessary recrystallised from ethyl benzoate to yield the respective 5,5′- and 6,6′-dibromo-, diphenyl-, and dimethoxy-indigos 1a–g in 22–58% yield (Table 1).

**Table 1:** The synthesis of methoxy-, phenyl- and bromo-substituted indigos

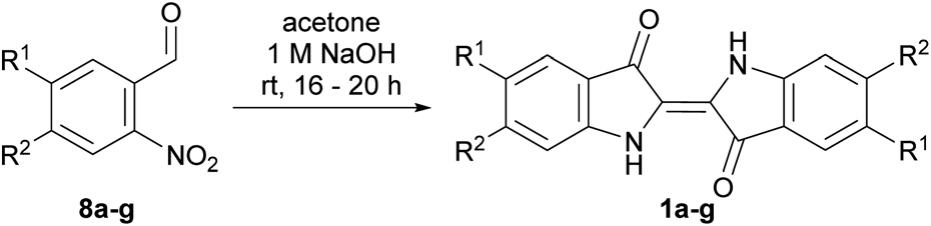						
Entry	R^1^	R^2^		Time	Yield	
1	OMe	H	8a	16 h	1a	58%
2	OMe	OMe	8b	20 h	1b	22%
3	H	OMe	8c	20 h	1c	30%
4	Ph	H	8d	20 h	1d	36%
5	H	Ph	8e	20 h	1e	28%
6	Br	H	8f	16 h	1f	35%
7	H	Br	8g	20 h	1g	32%

**Scheme 2:**
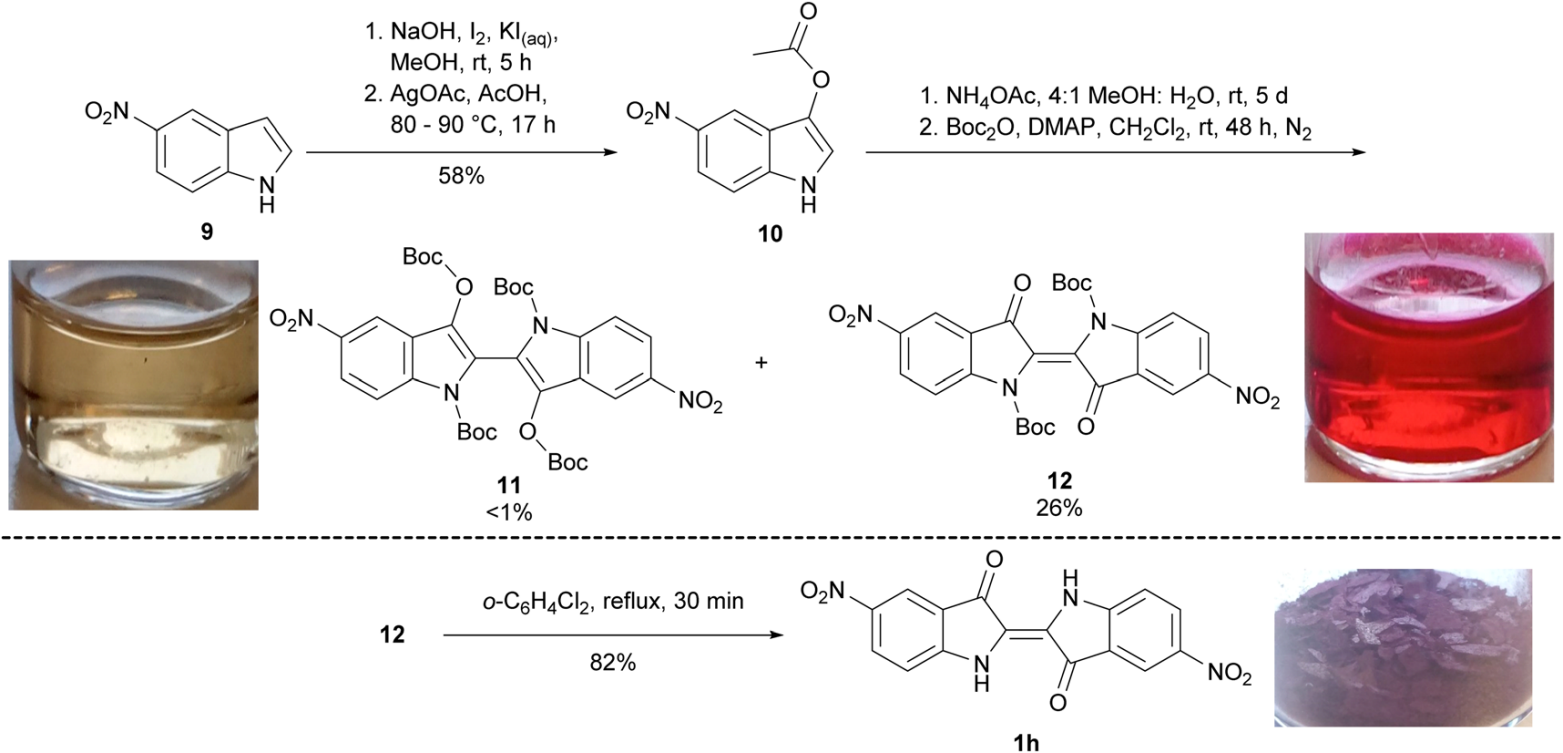
The synthesis of 5,5′-dinitroindigo 1h. The image of compound 1h and solutions of 11 and 12 in CH_2_Cl_2_ were taken under ambient lighting.

To synthesise 5,5′-dinitroindigo 1i, a literature procedure^13^ was modified, wherein 5-nitroindole 9 was 3-iodinated, and the product converted to 3-acetoxy-5-nitroindole 10 in the presence of AgOAc (58% yield over two steps, Scheme 2). Attempted hydrolysis of 3-acetoxy-5-nitroindole 10 under acidic or basic conditions produced 5,5′-dinitroindigo 1h as a crude solid which could not be further purified by washing with organic solvents or recrystallisation. Therefore, a known hydrolysis procedure^14^ was modified, in which 3-acetoxy-5-nitroindole 10 was dissolved in MeOH and a solution of NH_4_OAc in H_2_O added and stirred for 5 days. The resulting precipitate was filtered, dried, reacted with Boc_2_O and DMAP for 48 h and upon purification, yielded *N*,*N′*,*O*,*O′*-tetraBoc-5,5′-dinitrobiindole-3,3′-diol 11 (<1%) and *N*,*N′*-diBoc-5,5′-dinitroindigo 12 (26%, Scheme 2). Finally, heating *N*,*N′*-diBoc-5,5′-dinitroindigo 12 at reflux in 1,2-dichlorobenzene enabled thermal Boc-deprotection to yield 5,5′-dinitroindigo 1h in 82% yield.

**Scheme 3:**
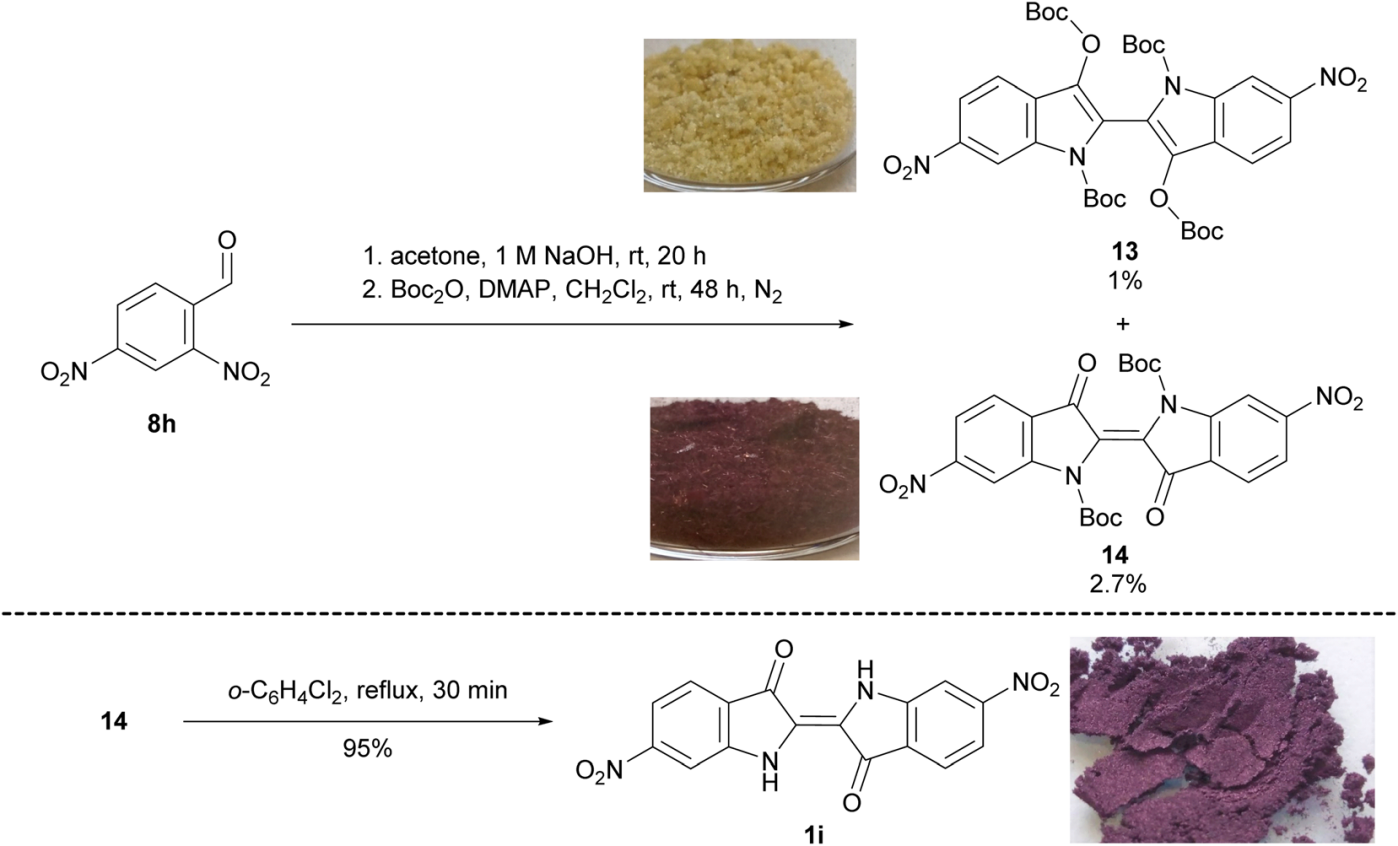
The synthesis of 6,6′-dinitroindigo 1i. Images of compounds 13, 14 and 1i were taken under ambient lighting.

Initial attempts involving the Baeyer–Drewson synthesis with 2,4-dinitrobenzaldehyde 8h produced 6,6′-dinitroindigo 1i in poor yield with impurities inseparable by recrystallisation or washing with organic solvents. Therefore, 2,4-dinitrobenzaldehyde 8h was reacted with acetone and 1 M NaOH for 20 h, the crude indigo was filtered, air-dried and reacted with Boc_2_O and DMAP in CH_2_Cl_2_ for 48 h. Subsequent multiple rounds of column chromatography and recrystallisation yielded *N*,*N′*,*O*,*O′*-tetraBoc-6,6′-dinitrobiindole-3,3′-diol 13 (1%) and *N*,*N′*-diBoc-6,6*′*-dinitroindigo 14 (2.7%, Scheme 3). Finally, the heating of *N*,*N′*-diBoc-6,6*′*-dinitroindigo 14 in 1,2-dichlorobenzene at reflux for 30 min furnished 6,6*′*-dinitroindigo 1i in excellent yield (95%), partly compensating for the very low yield of 14. Attempts to optimise the yield of indigo 1i in the initial step of this reaction were unsuccessful, suggesting an alternative route is required to improve the yield.

The isolation of *N*,*N′*,*O*,*O′*-tetraBoc-dinitrobiindole-3,3′-diols 11 and 13 was unexpected due to the two-electron reduction of the indigo moiety to a leucoindigo required to enable their formation. Initially, the synthesis of 13 (Scheme 3) was thought to be occurring as a result of disproportionation of the nitrobenzaldehyde 8h*via* a Cannizzaro reaction,^15^ however the isolation of 13 through a different synthetic pathway that did not utilise nitrobenzaldehyde starting materials suggested another mechanism of reduction was contributing to this result. One alternative mechanism of reduction could involve *tert*-butoxide, a by-product of Boc_2_O hydrolysis in the Boc-protection reactions. *tert*-Butoxide has been previously observed to reduce Ag(i) and Au(i) to the respective Ag(0) and Au(0) nanoparticles.^16^

### The allylation of substituted indigos

The investigation of the effects of substituted indigos on allylation cascade outcomes (Table 2) began with optimisations using 5,5′-dimethoxyindigo 1a. Therefore, a suspension of 5,5′-dimethoxyindigo 1a in DMF was sonicated for 60 min, cannulated into a flask containing pre-dried Cs_2_CO_3_ and activated 3 Å M.S. and heated at 85–88 °C for 60 min. Treatment with allyl bromide for 2 min followed by reaction workup, two rounds of column chromatography and recrystallisation yielded *N*-allyl-5,5′-dimethoxyindigo A (15, 6%), diallylbiindolone B (16, 3%) and spiroindolinepyridoindolone C (17, 37%, Table 2, entry 1). Repetition of this reaction with allyl bromide until the consumption of the *N*-allyl-5,5′-dimethoxyindigo 15 as determined by TLC analysis (5 min reaction time) led to the isolation of diallylbiindolone 16 in 3% yield and spiroindolinepyridoindolone 17 in an improved yield of 69% (Table 2, entry 2). Application of the optimised reaction conditions to indigos 1b–i with quenching upon the disappearance of *N*-allylindigos by TLC analysis led to the isolation of spiroindolinepyridoindolones C (18–25) as the major products in 36–75% yield (Table 2, entry 3–10). In the reactions of 5,5′,6,6′-tetramethoxy-, 6,6′-dimethoxy-, 5,5′-diphenyl- and 6,6′-diphenylindigos 1b–e, *C*-allylspiroindolinepyridoindolediones D (26–29) were also isolated in 3–11% yields (Table 2, entry 3–6). The reaction of 6,6′-dinitroindigo 1i was also observed to generate the novel oxazinodiindole derivative E (30, 13%) and *N*,*N′*-diallyl-3,3′-bis(allyloxy)biindole F (31, 1%) as additional minor outcomes (Table 2, entry 10).

**Table 2:** The allylation of substituted indigos. Images of solutions of 15, 16, 20, 28, 30 and 31 in CH_2_Cl_2_ were taken under ambient lighting or with 365 nm UV irradiation. Note – the relative stereochemistry is shown for spiroindolinepyridoindoledione D

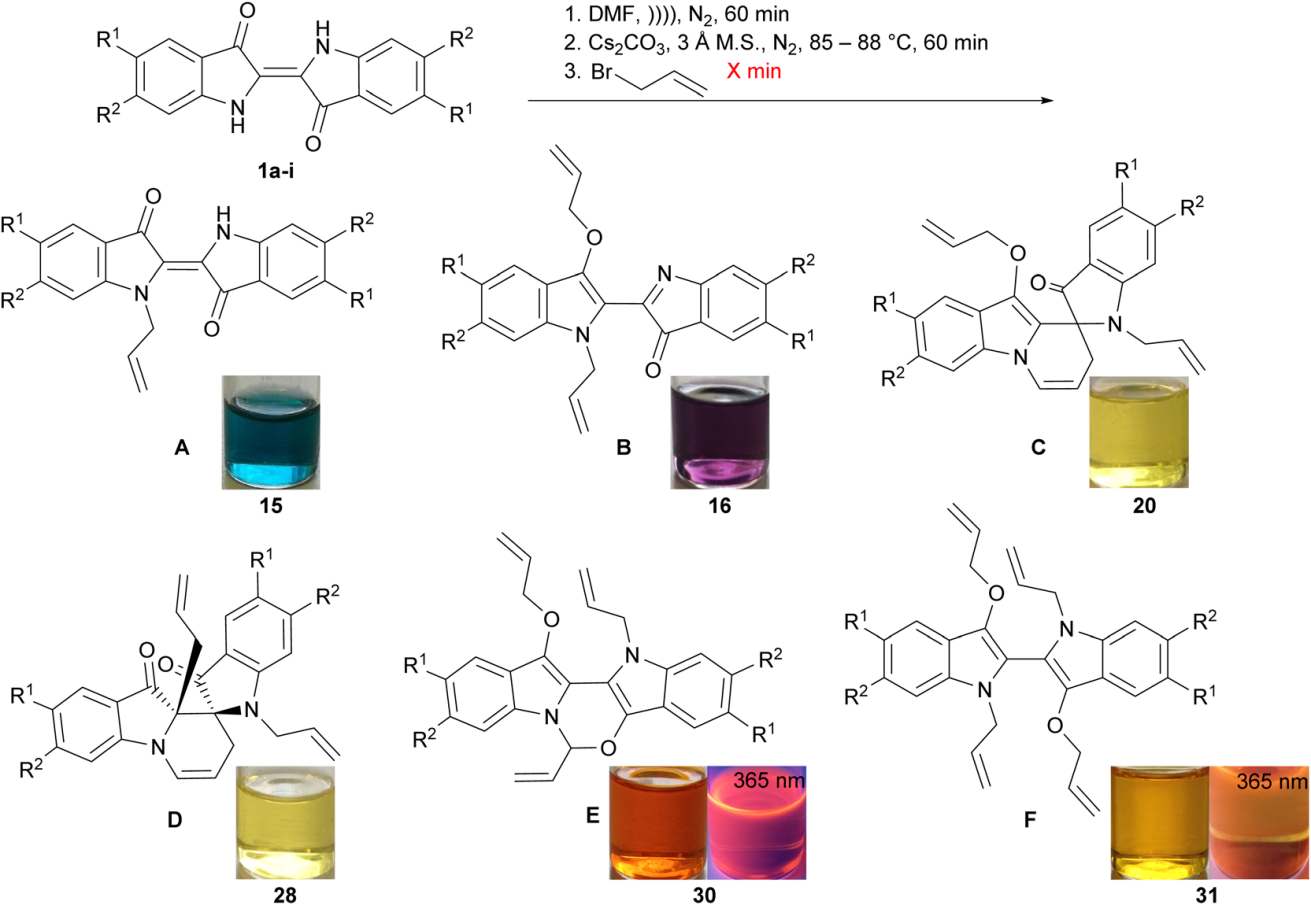																
Entry	R^1^	R^2^		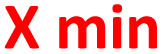	A		B		C		D		E		F	
1	OMe	H	1a	2	15	6%	16	3%^b^	17	37%	—		—		—	
2	OMe	H	1a	5	—		16	3%^b^	17	69%	—		—		—	
3	OMe	OMe	1b	60	—		—		18	56%	26	10%	—		—	
4	H	OMe	1c	60	—		—		19	43%^c^	27	11%^d^	—		—	
5	Ph	H	1d	17	—		—		20	75%	28	3%^b^	—		—	
6	H	Ph	1e	45	—		—		21	58%	29	3%^b^	—		—	
7	Br	H	1f	5	—		—		22^a^	62%	—		—		—	
8	H	Br	1g	60	—		—		23	69%	—		—		—	
9	NO_2_	H	1h	70	—		—		24	51%	—		—		—	
10	H	NO_2_	1i	8	—		—		25	36%	—		30	13%	31	1%

a X-ray quality crystals obtained – see ESI, Section S5.

b Presence of some grease/impurities in sample – <10 mg isolated precluding further purification.

c Corrected yield (NMR).

d Small amount of grease present in final sample.

The structure of diallylbiindole 16 was confirmed by the presence of strong 2D HMBC and HSQC correlations between the allyl substituents and the indole ring system and a ^13^C resonance at 156.4 ppm, characteristic of the imine moiety (see ESI,† Section S4.3). The generated spiroindolinepyridoindolones 17–25 showed similar spectral characteristics to those previously reported derivatives,^8,9^ with further confirmation provided by NMR analysis and an X-ray crystal structure of 5,2′-dibromo derivative 22 (see ESI,† Section S4.4 and S5). Key evidence suggesting the formation of *C*-allylspiroindolinepyridoindoledione 27 included the presence of two ^13^C resonances at 197.2 and 195.9 ppm, corresponding to two carbonyl moieties, and strong HMBC correlations from the spirocyclic carbon and the carbonyl at 195.9 ppm to the *C*-allyl methylene protons (see ESI,† Section S4.5). All *C*-allylspiroindolinepyridoindolediones 26–29 showed similar spectral characteristics and were all found to possess transoid relative stereochemistry (*vide infra*). The structure of oxazinobiindole 30 was determined based primarily upon the characteristic methine ^1^H and ^13^C NMR resonances at 6.81 ppm and 84.0 ppm, respectively, and relevant 2D COSY, HMBC and NOESY correlations to the vinyl pendant (see ESI,† Section S4.6). The simple ^1^H NMR and ^13^C NMR spectrum of *N*,*N′*-diallyl-3,3′-bis(allyloxy)biindole 31 suggested the structure was symmetrical. The structure of *N*,*N′*-diallyl-3,3′-bis(allyloxy)biindole 31 was proposed based upon analysis of the ^1^H NMR spectrum which showed resonances assigned to two distinct allyl substituents. Analysis of the ^13^C NMR spectrum showed an absence of resonances corresponding to carbonyl moieties. Importantly, the HRMS spectrum showed a peak at 537.1735, assigned to [M + Na]^+^ of the tetra-allylated 31 (see ESI,† Section S4.7).

### Mechanistic insights

**Scheme 4:**
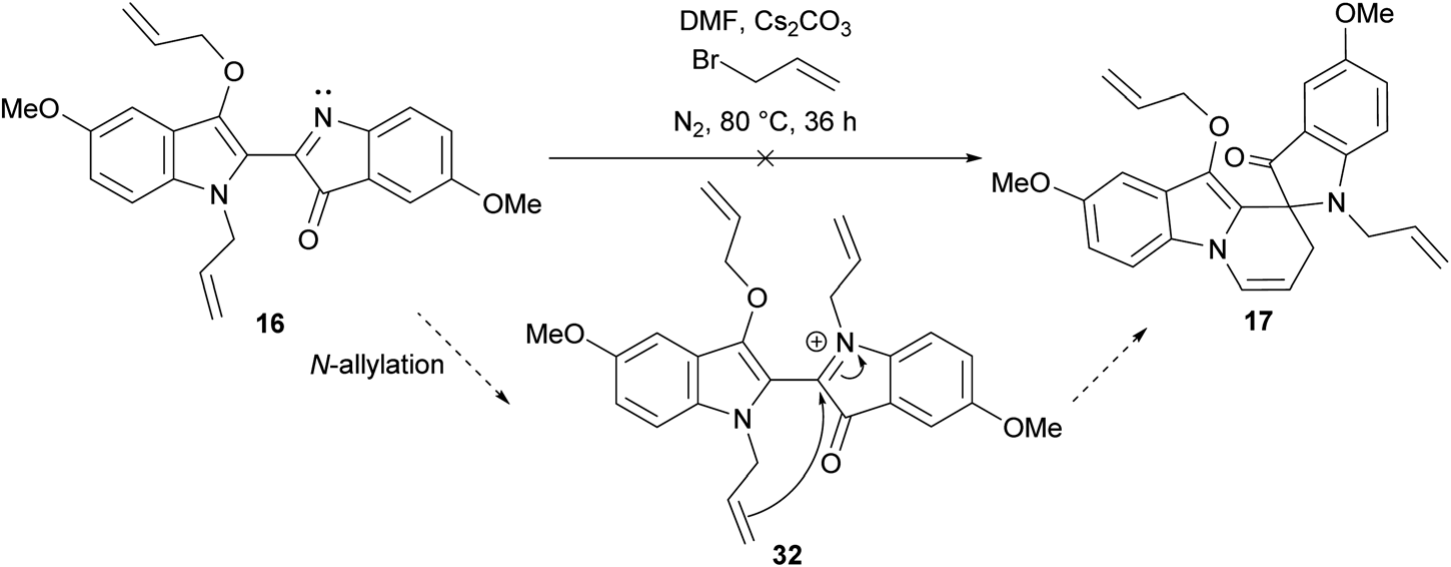
The proposed mechanism^8^ and investigation into the role of diallylbiindolone 16 as an intermediate to spiroindolinepyridoindolone 17.

**Scheme 5:**
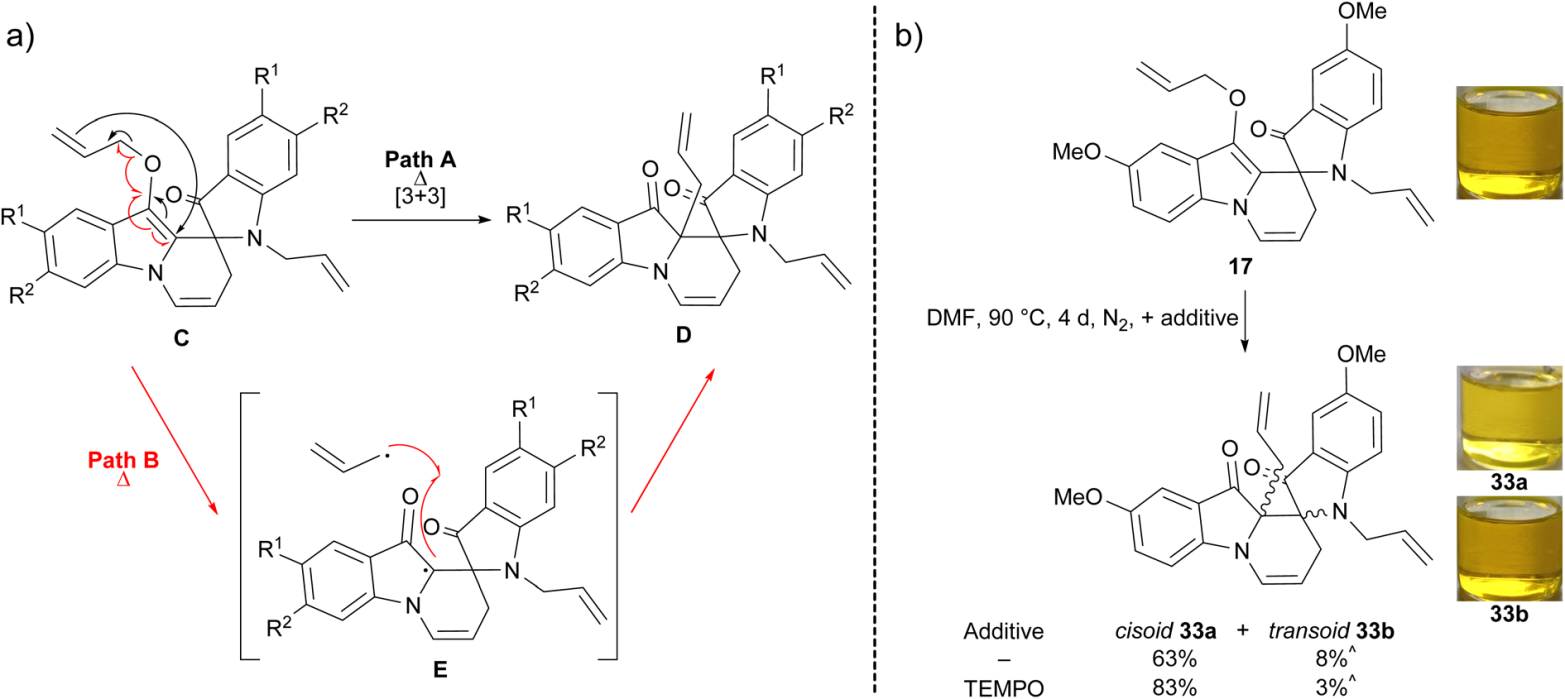
The (a) proposed mechanism of spiroindolinepyridoindolone C conversion to *C*-allylspiroindolinepyridoindoledione D*via* a Claisen rearrangement (
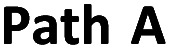
) or radical mechanism (
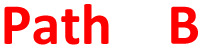
) and (b) mechanistic investigation of *C*-allylspiroindolinepyridoindoledione 33a–b formation. ^ presence of some grease in sample.

The synthesis of spiroindolinepyridoindolones C (17–25) as the major outcome of all the substituted indigo allylation reactions represented a shift from the allylation of indigo 1 itself, wherein spiroindolinepyridoindolone 3 represented a minor product (Scheme 1).^9^ The isolation of diallylbiindolone 16 was an intriguing outcome as the *N*-allylation of diallylbiindolone 16 to iminium intermediate 32 followed by 6-*exo-trig* cyclisation would furnish spiroindolinepyridoindolone 17, making it a potential mechanistic intermediate (Scheme 4).^8^ Treatment of diallylbiindolone 16 with Cs_2_CO_3_ and allyl bromide in DMF at 80 °C, however, showed baseline decomposition and the presence of unreacted 16 after 36 h, suggesting 16 is not a key intermediate involved in the generation of spiroindolinepyridoindolone 17 (Scheme 4). The isolation of *C*-allylspiroindolinepyridoindolediones D (26–29) was not the first observation of this scaffold, which has been isolated in the cascade reaction of indigo 1 with cinnamyl bromide (7, Scheme 1).^9^ The simplest mechanism to account for the formation of spiroindolinepyridoindoledione D is a thermally-induced Claisen rearrangement of spiroindolinepyridoindolone C (Scheme 5a, Path A). Alternatively, a radical mechanism is possible, wherein homolytic cleavage of the allyl ether of spiroindolinepyridoindolone C would generate an allyl radical and captodatively-stabilised radical E, which upon unification would yield *C*-allylspiroindolinepryridoindoledione D, as was previously proposed in the synthesis of cinnamyl *C*-allylspiroindolinepyridoindoledione 7 (Scheme 5a, Path B).^9^

To confirm the spiroindolinepyridoindolediones D form through a thermal electrocyclic process, a solution of 5,2′-dimethoxyspiroindolinepyridoindolone 17 in DMF was heated at 90 °C for 4 days, to yield cisoid- and transoid-spiroindolinepyridoindolediones 33a–b in 63% and 8% yield, respectively (Scheme 5b). Repetition of this reaction with the addition of TEMPO as a radical scavenger also yielded cisoid- and transoid-spiroindolinepyridoindolediones 33a–b in 83% and 3% yield respectively, suggesting this reaction proceeds *via* a Claisen rearrangement rather than a radical pathway (Scheme 5b). The cisoid- and transoid-isomers 33a–b were distinguished *via* NMR spectroscopy based upon differences in allyl methylene peak splittings due to restricted rotation in 33a and *N*-allyl substituent deshielding in 33b, thought to be the result of its closer proximity to the pyridoindolone aromatic ring relative to 33a (see ESI,† Sections S4.8 and S4.9). The cisoid-spiroindolinepyridoindoledione 33a possessed identical spectral characteristics to derivatives 26–29, strongly suggesting 26–29 also have cisoid relative stereochemistry.

**Scheme 6:**
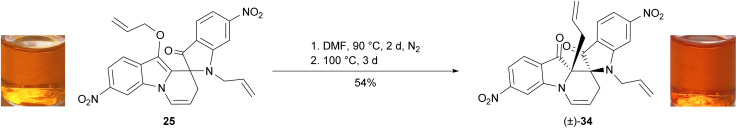
The synthesis of 6,3′-dinitrospiroindolinepyridoindoledione 34.

The generation of the *C*-allylspiroindolinepyridoindolediones D appeared to follow a positive correlation with electron-richness, with the greatest yields observed in reactions with methoxy-and phenyl-substituted indigos, while no formation was observed in bromo- and nitro-substituted indigos (Table 2). In the case of 5,5′-dimethoxyindigo 1a (Table 2, entry 1–2), the lack of *C*-allylspiroindolinepyridoindoledione D formation *in situ* was proposed to be due to a shorter heating time (2–5 min), which was shown to be the case with the isolation of 33a–b in high yield upon heating 5,2′-dimethoxyspiroindolone 17 at 90 °C for 4 days (Scheme 5b). To determine if electron-deficient derivatives of type D are accessible, 6,3′-dinitrospiroindolinepyridoindolone 25 was heated in DMF at 90 °C for 2 days, however no consumption of starting material was observed by TLC analysis. The reaction was therefore heated at 100 °C for a further 3 days, which upon workup, column chromatography and recrystallisation furnished 6,3′-dinitrospiroindolinepyridoindoledione 34 in 54% yield (Scheme 6).

**Scheme 7:**
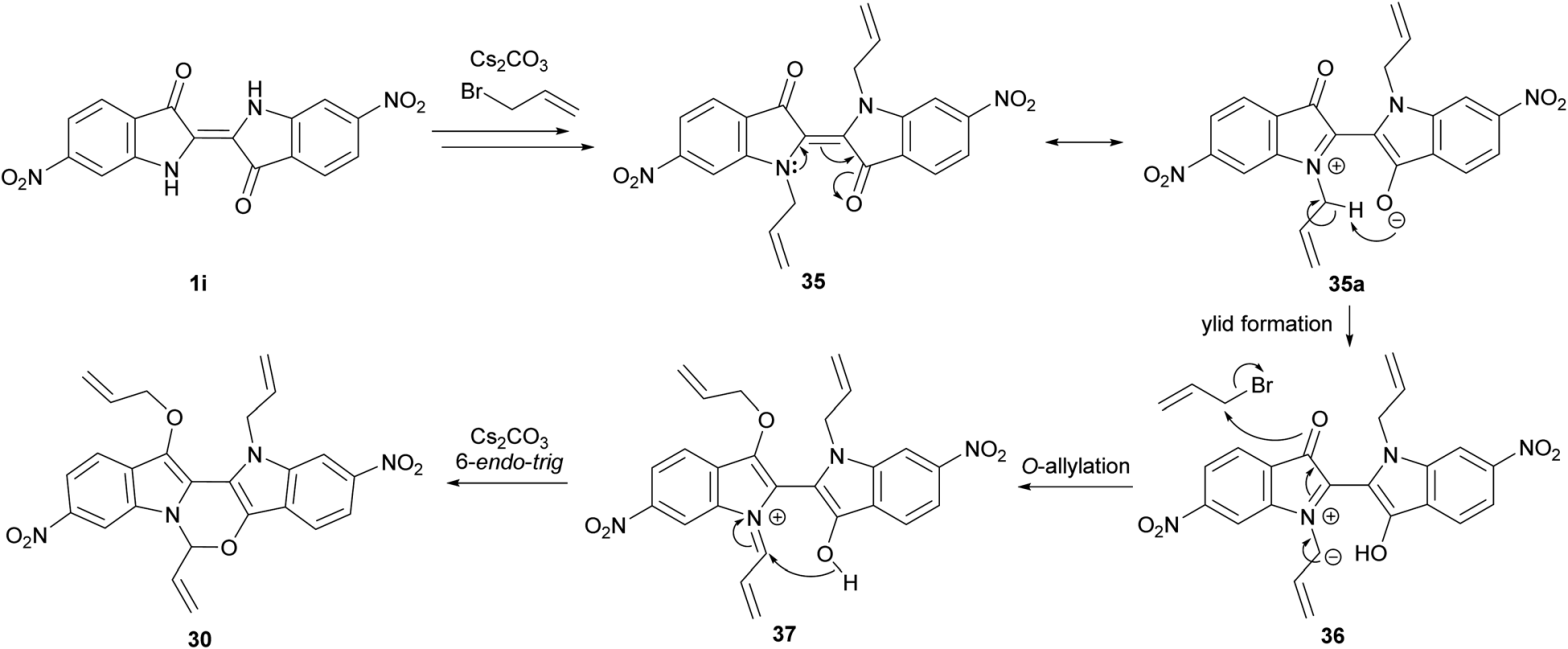
The proposed mechanism of oxazinodiindole 30 formation.^11^

**Scheme 8:**
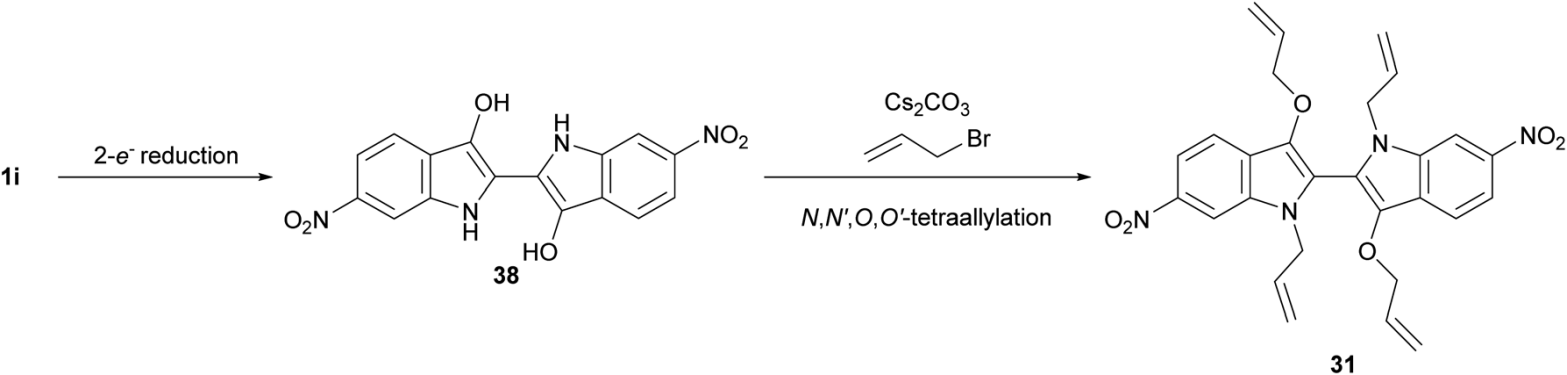
The proposed mechanism of *N*,*N′*-diallyl-3,3′-bis(allyloxy)biindole 31 formation.

Oxazinodiindole 30 represented a novel structural motif for the allylation cascade reactions, having only been observed in propargylation reactions previously,^11^ while also representing the first cascade product unifying allylation and propargylation reaction mechanisms. Analogous to the mechanism previously proposed for propargyl oxazinodiindole,^11^*N*,*N*′-diallylation of 6,6′-dinitroindigo 1i was proposed to produce 35, which mesomerises to enolate-iminium intermediate 35a and upon intramolecular methylene proton abstraction forms ylid 36 (Scheme 7). The formation of a methylene-based iminium ion and *O*-propargylation would generate 37, which could undergo a base-mediated 6-*endo-trig* cyclisation to provide oxazinodiindole 30. The isolation of *N*,*N′*-diallyl-3,3′-bis(allyloxy)biindole 31 also represented a new motif generated in the cascade reactions of indigo. Compound 31 was proposed to form *via* a 2-e^−^ reduction of 6,6′-dinitroindigo 1i to 6,6′-dinitroleucoindigo 38 followed by tetra-allylation, though *N*-allylation or *N*,*N′*-diallylation followed by reduction and *O*-allylation may also occur (Scheme 8). Another possibility is that diallylbiindole type B is reduced and alkylated to generate *N*,*N′*-diallyl-3,3′-bis(allyloxy)biindole 31, which could explain the lack of diallylbiindole B products in electron-deficient derivatives. While DMF could be a reductant *in situ*,^17–20^ this is unlikely as repetition of this reaction using anhydrous MeCN as the solvent also led to the formation of *N*,*N′*-diallyl-3,3′-bis(allyloxy)biindole 31, suggesting an alternate reductive process is present (see ESI,† Section S1.2). Another possibility is that indigo 1i is the reductant, generating diallylbiindole 31 and indigo oxidative by-products such as isatin and anthranilic acids *in situ*.

**Scheme 9:**
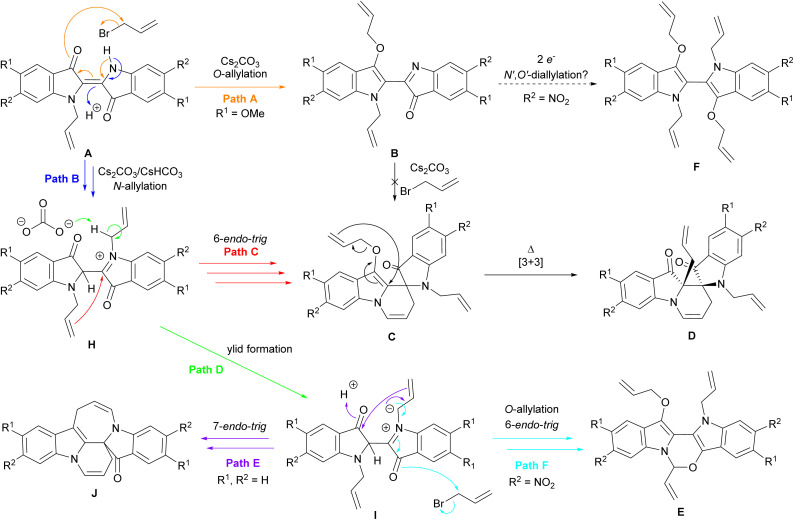
The updated mechanistic pathways of indigo allylation cascade reactions. Note – the relative stereochemistry is shown for spiroindolinepyridoindoledione D.

These investigations have provided substantial insights into the allylation cascade reactions of indigo and further expanded the mechanistic pathway (Scheme 9). Upon the formation of *N*-allylindigo A*in situ*, *O*-allylation to generate diallylbiindolone B (Path A) was shown to exist as a mechanistic pathway when R^1^ = OMe, though it is hypothesised that when R^2^ = NO_2_, B may be reduced and alkylated to generate *N*,*N′*-diallyl-3,3′-diallyloxybiindole F. The attempted *N*-allylation and cyclisation of B did not produce spiroindolinepyridoindolone C as predicted previously, suggesting the formation of *N*,*N′*-diallyl intermediate H (Path B) followed by 6-*endo-trig* cyclisation and *O*-allylation (Path C) is the dominant pathway contributing to the synthesis of spiroindolinepyridoindolones C. Further, spiroindolinepyridoindolones C were shown to undergo thermal Claisen rearrangement to generate *C*-allylspiroindolinepyridoindolediones D. Diallyl intermediate H was also suggested to generate ylid I (Path D), which was thought to be a mechanistic branchpoint giving rise to either azepinoindolone J (Path E) when R^1^ = R^2^ = H,^8,9^ or oxazinobiindole E when R^2^ = NO_2_ (Path F).

## Conclusions

A variety of substituted indigos 1a–i were successfully synthesised and evaluated in allylation cascade reactions, leading to the isolation of diallylbiindolone 16, oxazinobiindole 30 and *N*,*N′*-diallyl-3,3′-bis(allyloxy)biindole 31 as novel polyheterocyclic architectures. The isolation of these products provided evidence for, and therefore strong support of, the proposed mechanisms of indigo cascade reactions. These mechanistic investigations could also, with further development, enable the synthesis of specific cascade products by modifying the substituents attached to indigo and the electrophile. The isolation of tetra-alkylated dinitrobiindole derivatives 11, 13 and 31 also suggest that electron-deficient indigos more readily participate in redox processes, providing another avenue for future investigation.

## Experimental details

Detailed experimental data and characterisation can be found in the ESI, Section S2.† General procedures for indigo synthesis and allylation cascade reactions are provided below for convenient reference.

### General procedure A: the Baeyer–Drewson indigo synthesis

Following a modified procedure,^21^ to a solution of substituted 2-nitrobenzaldehydes 8a–g (1.0 eq.) in acetone (1.8 mL mmol^−1^), was added NaOH (1 M, 0.9 mL mmol^−1^) dropwise over 5–20 min at 2 °C. Upon addition, the ice bath was removed and the reaction stirred at rt. After 16–20 h, the reaction was diluted with H_2_O (4 mL mmol^−1^) and the precipitate filtered, rinsed with H_2_O until the filtrate was colourless (5 × 20–50 mL) and EtOAc (5 × 20–50 mL) until the filtrate was colourless or pale blue. Upon air drying, the crude solid was recrystallised (EtOBz, 10 mg mL^−1^) and the precipitate or crystals filtered and washed with EtOAc (3 × 20–50 mL) to yield substituted indigos 1a–g.

### General procedure B: the allylation of substituted indigos

A suspension of substituted indigos 1a–i (1.0 eq.) in anhydrous DMF (40 mL mmol^−1^) was sonicated for 60 min under a N_2_ atmosphere. The suspension was transferred to a flask containing pre-dried Cs_2_CO_3_ (4.0 eq.) and activated 3 Å M.S. (2 g mmol^−1^) and plunged into a pre-heated oil bath at 85–88 °C under N_2_ with stirring. After 60 min, the N_2_ flow was cut and allyl bromide (5.0 eq.) was added and stirred for 2–70 min. The reaction was quenched with ice (200 g mmol^−1^) and diluted with brine (to 400 mL mmol^−1^). This mixture was extracted with EtOAc (4 × 40–100 mL mmol^−1^), and the combined organic phases washed with H_2_O (5 × 50 mL mmol^−1^) and brine (2 × 25 mL mmol^−1^), dried (MgSO_4_) and concentrated *in vacuo*. The residue was subjected to multiple rounds of purification by silica gel column chromatography, PTLC and/or recrystallisation to furnish the titled cascade products 15–31.

## Author contributions

Matthew Perry: conceptualization; formal analysis; investigation; methodology; visualisation; writing – original draft preparation; writing – review and editing; Anthony Willis: formal analysis; investigation; methodology; John Bremner: conceptualization; formal analysis; methodology; writing – review and editing; Paul Keller: conceptualization; formal analysis; methodology; project administration; supervision; writing – review and editing.

## Conflicts of interest

There are no conflicts to declare.

## Supplementary Material

RA-013-D3RA00481C-s001

RA-013-D3RA00481C-s002
