# Supplementary material for: An investigation of the allylation cascade reactions of substituted indigos
Source: RSC Adv. 2023 Feb 6;13(7):4865–73. doi: 10.1039/d3ra00481c (PMC9901421; doi:10.1039/d3ra00481c)
Supplement: RA-013-D3RA00481C-s001 [file RA-013-D3RA00481C-s001.pdf]

Electronic Supplementary Material (ESI) for RSC Advances.
This journal is © The Royal Society of Chemistry 2023

Electronic Supplementary Information

Investigation of the Allylation Cascade Reactions of Substituted Indigos

Matthew J. Perry,[a] Anthony C. Willis,[b] John B. Bremner,[a] and Paul A. Keller[a]*

[a] School of Chemistry and Molecular Bioscience, Molecular Horizons, and Illawarra Health and Medical Research Institute, University of Wollongong, Wollongong, NSW, 2522

[b] Research School of Chemistry, Australian National University, Canberra, ACT, 2601

Table of Contents

S1. Supplementary Reactions 2

S1.1 The Synthesis of phenyl-2-nitrobenzaldehydes **8d-e** 2

S1.2 The Allylation of 6,6'-dinitroindigo **1i** in MeCN 2

S2. Experimental Details 4

S2.1 General Information 4

S2.2 The Synthesis of Substituted Indigos 6

S2.3 The Cascade Reactions of Substituted Indigos 15

S2.4 Mechanistic Experiments 31

S3. NMR Characterisation 36

S3.1 NMR spectra of substituted indigos and key intermediates 36

S3.2 NMR spectra of allylation cascade products 47

S4. Structural Elucidation of Novel Polyheterocycles 66

S4.1 Structural elucidation of 3-acetoxy-5-nitroindole **10** 67

S4.2 Structural elucidation of *N,N'*,*O,O'*-tetraBoc-biindole-3,3'-diols **11** and **13** 69

S4.3 Structural elucidation of diallylbiindolone **16** 70

S4.4 Structural elucidation of spiroindolinepyridoindolones **17 – 25** 73

S4.5 Structural elucidation of *cisoid* *C*-allylspiroindolinepyridoindolediones **26 – 29** 75

S4.6 Structural elucidation of oxazinodiindole **30** 79

S4.7 Structural elucidation of *N,N'*-diallyl-3,3'-bis(allyloxy)biindole **31** 82

S4.8 Structural elucidation of *transoid* *C*-allylspiroindolinepyridoindoledione **33b** 84

S4.9 Assignment of *C*-allylspiroindolinepyridoindoledione **33a-b** relative stereochemistry 86

S5. X-Ray Crystal Data of Spiroindolinepyridoindolone **22** 89

S6. References 90

S1. Supplementary Reactions

S1.1 The Synthesis of phenyl-2-nitrobenzaldehydes 8d-e

Based on a known procedure,[1] a Schlenk flask charged with $PhB(OH)_2$, $Pd(OAc)_2$ (2 mol%), acac (6 mol%) and either 5-bromo-2-nitrobenzaldehyde **8f** or 4-bromo-2-nitrobenzaldehyde **8g** was purged with N_2, and degassed solutions of EtOH and 1 M Na_2CO_3 were added and the mixture heated at 90 °C for 1.5 – 2 h, which upon workup, column chromatography and recrystallisation yielded 2-nitro-5- and 4-phenylbenzaldehydes **8d** and **8e** in 73% and 68% yield, respectively (Scheme S1).

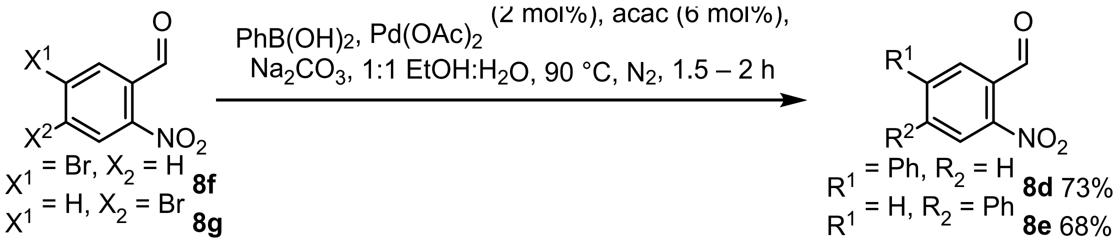

Scheme S1: The synthesis of phenyl-2-nitrobenzaldehydes **8d-e**.

S1.2 The Allylation of 6,6'-dinitroindigo 1i in MeCN

To probe whether DMF was acting as a reducing agent in the allylation of 6,6'-dinitroindigo **1i** to enable *N*,*N'*-diallyl-3,3'-bis(allyloxy)biindole **31** formation, the cascade reaction was repeated using anhydrous MeCN as the solvent. Therefore, 6,6'-dinitroindigo **1i** was sonicated for 1 h in anhydrous MeCN, deprotonated for 1 h at 85 – 88 °C and treated with allyl bromide (Scheme S2a). After 20 min, TLC analysis showed the presence of *N*,*N'*-diallyl-3,3'-bis(allyloxy)biindole **31** (Scheme S2b-c), suggesting DMF is not the principal reductant in this reaction.

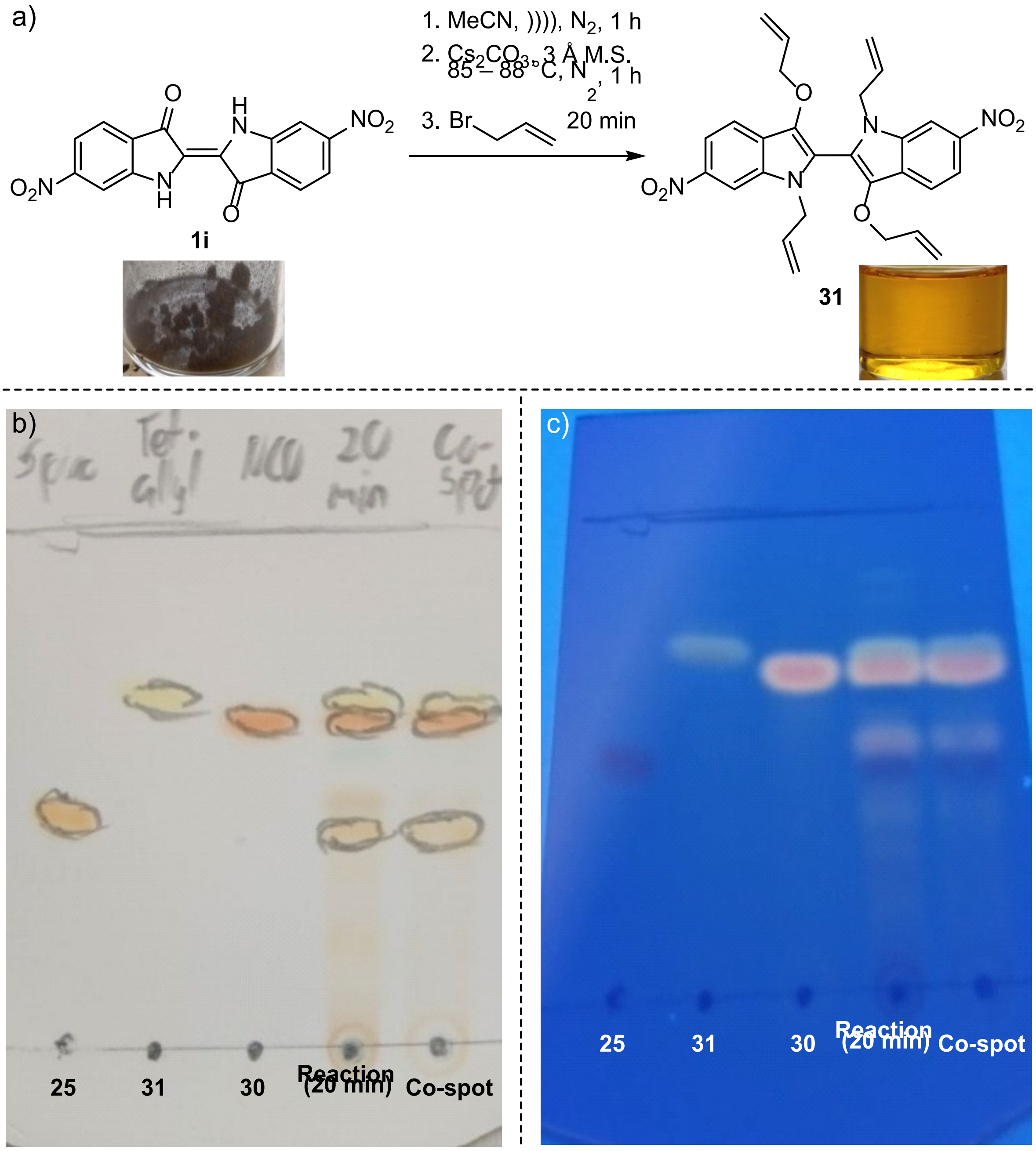

Scheme S2: a) The allylation cascade reaction of 6,6'-dinitroindigo **8i** using MeCN as the solvent with TLC analyses under b) ambient lighting and c) 365 nm UV irradiation. Lanes from left to right are pure samples of 1) spiroindolinepyridoindolone **25**, 2) *N*,*N'*-diallyl-3,3'-bis(allyloxy)biindole **31** and 3) oxazinodiindole **30**, 4) A sample of the crude reaction mixture after 20 min and 5) a co-spot of all previous lanes. Images of **1i** and the solution of **31** in CH_2Cl_2 were taken under ambient lighting.

S2. Experimental Details

S2.1 General Information

Reagent grade solvents were used with the exception of HPLC grade CH_2Cl_2, CH_3OH, MeCN, acetone and hex, which were used without purification as purchased from Ajax Finechem or Chemsupply. Petroleum spirit (pet) used had a boiling range of 40 – 60 °C. Anhydrous DMF was obtained from Sigma-Aldrich and was stored sealed under a static N_2 atmosphere. Activation of 3 Å M.S. was achieved by heating in a furnace at >300 °C for a minimum of 24 h followed by storage in a desiccator. The N_2 used in reactions was desiccated using two drying tubes filled with silica gel beads and finely powdered anhydrous $CaCl_2$.

Water used in the reactions and workup procedures was purified by reverse osmosis (RO). All other reagents were procured from Sigma-Aldrich, AK-Scientific or Thermo Fisher Scientific. Glassware was cleaned using technical grade acetone, detergent, and where necessary, 36% HCl. Glassware used was standard grade and was oven-dried at 100 °C.

Sonication was performed using a UC-S3200H Ultrasonic Cleaner with a 40 KHz frequency. Drying *in vacuo* was achieved by rotary evaporation followed by drying on a high vacuum pump for a minimum of 6 h. Column chromatography was performed with silica gel 60 (40 – 63 µM, 230 – 400 mesh) under positive pressure (compressed air).

Analytical TLC was performed using 0.20 mm silica gel plates on aluminium backing, with the retention factor (R_f) measured from the centre of the spot. Reaction progress was monitored by TLC analysis and for reactions under an inert N_2 or Ar atmosphere, the sample (0.1 mL) was obtained through the rubber septum using a 1.0 mL syringe with an attached steel needle. For reactions conducted in DMF the sample (0.1 mL) was partitioned between H_2O (1 mL) and EtOAc (0.5 mL) and TLC analysis was performed on the EtOAc fraction. Visualisation of TLC plates was accomplished under either ambient light, 254 nm UV or 365 nm UV light. Preparative TLC (PTLC) purification was performed on glass-backed PTLC plates (silica gel 60, 20 x 20 cm, 0.5 mm thickness).

A Buchi M-565 apparatus was used to determine melting points. Infrared spectrometry was performed using a Bruker Vertex FTIR spectrometer in the ATR mode. Samples prepared as thin films were dissolved in Et_2O or isopropanol and deposited onto the ATR crystal. Spectral

peak intensities are reported strong (s), medium (m) and weak (w), and where applicable, peaks are labelled broad (b). Electron Impact (EI) Low-Resolution Mass Spectrometry (LRMS) was performed on a Shimadzu QP5050 in EI mode. To obtain EI-MS for nitro-, bromo-, phenyl- and methoxy-substituted indigos, a method of rt → 350 °C, 80 °C/min was applied. All other spectra were obtained through a rt → 200 °C, 50 °C/min method. Electrospray ionisation (ESI) LRMS was performed on a Shimadzu LCMS-2020 spectrometer. High-resolution mass spectrometry (HRMS) was performed on a Waters Quadrupole-Time of Flight (QTOF) Xevo spectrometer on ESI^+, ESI^- or $ASAP^+$ mode using a Leucine-Enkephalin standard. Samples were prepared in HPLC grade MeOH or MeCN. The ion mass-to-charge (*m*/*z*) values are reported as the relative abundance compared to the base peak with bromine and tin isotopes indicated where necessary. The molecular ion is reported as M. Proton (^{1}H), and carbon (^{13}C) NMR experiments were performed using either a Bruker Advance III Nanotray 400 MHz or Bruker Advance NEO 500 MHz spectrometer fitted with a cryoprobe utilising IconNMR software. Chemical shifts were recorded in parts per million (ppm) with coupling constants (*J*) reported in Hertz (Hz) and multiplicities reported as singlets (s), broad singlets (bs), doublets (d), triplets (t), multiplets (m) or apparent (appt.) multiplicities. Processing and display of NMR data was accomplished with Mestrelab Research Mestrenova 9.0.1. All spectra run in $CDCl_3$ were referenced to TMS (δ = 0.00 ppm) while ^{1}H/^{13}C NMR spectra performed in DMSO-d_6 (δ = 2.50/39.52 ppm) or acetone-d_6 (δ = 2.05/29.84 ppm) were referenced to the residual solvent resonances.

New compounds synthesised are reported underlined and known compounds are reported in **bold** lettering. Yields notated with: ^ = presence of some grease in sample - >10 mg isolated precluding further purification; † = Corrected yield (NMR); # = small amount of grease present in final sample. *C*-Allylated-spiro derivatives **28b** and **29**, the ‘impurity’ present is likely the *transoid* diastereomer.

S2.2 The Synthesis of Substituted Indigos

2.2.1 The synthesis of phenyl 2-nitrobenzaldehydes 8d-e

2-nitro-5-phenylbenzaldehyde 8d

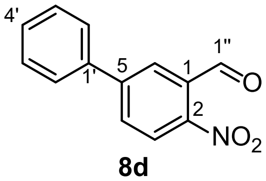

8d

To a 50 mL Schlenk flask equipped with a septum was added 5-bromo-2-nitrobenzaldehyde **8f** (1.840 g, 8.00 mmol), phenylboronic acid (1.366 g, 11.20 mmol), $Pd(OAc)_2$ (36 mg, 0.16 mmol, 2 mol%) and acac (49 µL, 0.48 mmol, 6 mol%), and purged with high vacuum/N_2 (3x). Degassed EtOH (12 mL) and degassed aqueous Na_2CO_3 (1 M, 12 mL) were added and the mixture heated and stirred for 2 h at 90 °C under a N_2 atmosphere. The reaction was cooled to rt, poured into brine (50 mL) and extracted with EtOAc (1 x 100 mL, 2 x 50 mL). The combined organic phases were washed with H_2O (2 x 50 mL) and brine (50 mL), dried ($MgSO_4$) and concentrated *in vacuo*. The residue was adsorbed onto silica (10 g) and subjected to column chromatography (100 g silica, 5% EtOAc/hex → 30% EtOAc/hex) to yield 2-nitro-5-phenylbenzaldehyde **8d** as a pale yellow solid (1.333 g, 73%). Spectral data matched previously reported values.[1] ^{1}H NMR (400 MHz, $CDCl_3$) δ 10.52 (s, 1H, H1''), 8.22 (d, *J* = 8.5 Hz, 1H, H3), 8.14 (d, *J* = 2.1 Hz, 1H, H6), 7.94 (dd, *J* = 8.5, 2.1 Hz, 1H, H4), 7.68 – 7.63 (m, 2H, H2' & H6'), 7.56 – 7.45 (m, 3H, H3' & H5', H4'); ^{13}C NMR (101 MHz, $CDCl_3$) δ 188.4 (C1''), 148.1 (C4), 147.4 (C1), 137.6 (C1'), 132.1 (C3), 131.6 (C6), 129.5 (C4'), 129.4 (C3' & C5'), 127.9 (C2), 127.4 (C2' & C6'), 125.4 (C5); LRMS (EI) *m/z* 197 (86%) $[M\text{-}CHO\text{-}H]^{\bullet+}$, 227 (100%) $[M]^{\bullet+}$.

2-nitro-4-phenylbenzaldehyde 8e

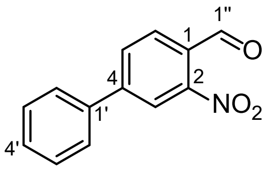

8e

To a 50 mL Schlenk flask equipped with a septum was added 4-bromo-2-nitrobenzaldehyde **8g** (1.840 g, 8.00 mmol), phenylboronic acid (1.366 g, 11.20 mmol), $Pd(OAc)_2$ (35 mg, 0.16 mmol, 2 mol%) and acac (49 µL, 0.48 mmol, 6 mol%) and purged with high vacuum/N_2 (3x). Degassed EtOH (12 mL) and degassed aqueous Na_2CO_3 (1 M, 12 mL) were added and the mixture heated and stirred for 90 min at 90 °C under a N_2 atmosphere. The reaction was cooled to rt and poured into brine (20 mL), extracted with EtOAc (3 x 20 mL) and the combined organic phases washed with H_2O (3 x 20 mL) and brine (20 mL), dried ($MgSO_4$) and concentrated *in vacuo*. The residue was adsorbed onto silica (10 g) and subjected to column chromatography (100 g silica, 5% → 10% EtOAc/hex) to yield two major fractions (F_1 and F_2).

Fraction F_1 contained 857 mg of pure 2-nitro-4-phenylbenzaldehyde **8e** and recrystallisation of F_2 (1:4 CH_2Cl_2:hex, 28 mL) yielded an additional 384 mg of **8e** as a pale yellow solid (total 1.241 g, 68%). Reported previously,[2-4] however no spectral data was published. mp 79 – 83 °C; R_f (20% EtOAc/hex) = 0.44; FTIR (thin film): ν_{max} 2899 (w), 1686 (s), 1612 (m), 1523 (s), 1507 (s), 1347 (s), 1261 (w), 1209 (w), 745 (m), 693 (m); ^{1}H NMR (400 MHz, $CDCl_3$) δ 10.46 (s, 1H, H1''), 8.31 (d, *J* = 1.7 Hz, 1H, H3), 8.05 (d, *J* = 8.0 Hz, 1H, H6), 7.99 (dd, *J* = 8.1, 1.7 Hz, 1H, H5), 7.69 – 7.64 (m, 2H, H2' & H6'), 7.56 – 7.47 (m, 3H, H3' & H5', H4'); ^{13}C NMR (101 MHz, $CDCl_3$) δ 187.8 (C1''), 150.3 (C2), 147.2 (C4), 137.4 (C1'), 132.0 (C5), 130.3 (C6), 129.6 (C4'), 129.4 (C3' & C5'), 129.3 (C1), 127.3 (C2' & C6'), 122.8 (C3); LRMS (EI) *m/z* 197 (100%) $[M-CHO-H]^{•+}$, 227 (17%) $[M]^{•+}$; HRMS (ASAP$^+$) Calculated for $C_{13}H_9NO_3^{•+}$ $[M]^{•+}$ 227.0582, found 227.0588.

2.2.2 The synthesis of methoxy- and bromo- and phenyl-substituted indigos

5,5'-dimethoxyindigo 1a

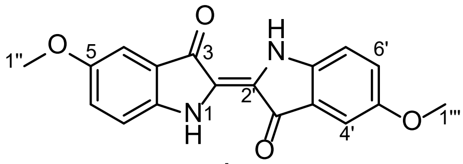

1a

Following a modified procedure,[5] to a solution of 5-methoxy-2-nitrobenzaldehyde **8a** (4.373 g, 24.1 mmol) in acetone (45 mL) and H_2O (44 mL) was added NaOH (1 M, 24 mL) dropwise over 10 min at 2 °C. The ice bath was removed and the reaction stirred at rt for 16 h, then diluted with H_2O (100 mL) and filtered. The precipitate was rinsed with H_2O (5 x 50 mL) and EtOAc (3 x 50 mL), air-dried and the crude material recrystallised (EtOBz, 230 mL), filtered and washed with EtOAc (3 x 50 mL) to yield 5,5'-dimethoxyindigo **1a** (2.276 g, 58 %) as purple plate crystals. Spectral data matched previously reported values.[6, 7] mp >250 °C; FTIR (neat): ν_{max} 3381 (b/m), 1624 (m), 1611 (m), 1480 (s), 1330 (m), 1270 (m), 1125 (s), 1068 (s), 1023 (s), 811 (s), 678 (s), 570 (s); ^{1}H NMR (400 MHz, DMSO-d_6, 80 °C) δ 10.00 (bs, 2H, H1 & H1'), 7.28 (d, *J* = 8.7 Hz, 2H, H7 & H7'), 7.17 (dd, *J* = 8.7, 2.7 Hz, 2H, H6 & H6'), 7.11 (d, *J* = 2.6 Hz, 2H, H4 & H4'), 3.80 (s, 6H, H1'' & H1'''); LRMS (EI) *m/z* 322 (100%) $[M]^{•+}$; HRMS (ESI$^+$) Calculated for $C_{18}H_{15}N_2O_4^+$ $[M+H]^+$ 323.1026, found 323.1026.

6,6'-dimethoxyindigo 1b

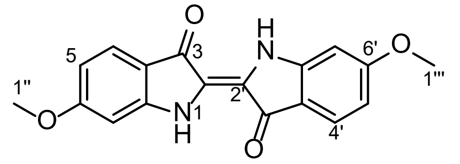

1b

Following General Procedure A, to a solution of 4-methoxy-2-nitrobenzaldehyde **8b** (2.899 g, 16.00 mmol) in acetone (29 mL) was added NaOH (1 M, 14.5 mL) dropwise over 10 min at 2 °C. The ice bath was removed and the reaction stirred at rt for 20 h. The precipitate was filtered and rinsed with H_2O (5 x 20 mL) and EtOAc (5 x 20 mL), recrystallised (EtOBz, 90 mL), filtered and rinsed with EtOAc (3 x 20 mL) to yield 6,6'-dimethoxyindigo **1b** as a pink-purple solid (776 mg, 30%). Synthesised previously,[8-12] however only the UV-Vis spectrum was reported.[11] mp >250 °C; FTIR (neat); ν_{max} 3261 (b/m), 2837 (m), 1611 (s), 1581 (s), 1446 (s), 1379 (m), 1332 (s), 1288 (s), 1206 (m), 1129 (s), 1101 (s), 1064 (s), 1029 (s), 849 (m), 762 (m), 677 (m), 601 (s); ^{1}H NMR (400 MHz, DMSO-d_6, 80 °C) δ 10.01 (bs, 2H, H1 & H1'), 7.53 (d, J = 8.6 Hz, 2H, H4 & H4'), 6.88 (d, J = 2.2 Hz, 2H, H7 & H7'), 6.53 (dd, J = 8.6, 2.2 Hz, 2H, H5 & H5'), 3.87 (s, 6H, H1'' & H1'''); LRMS (EI) m/z 322 (100%) $[M]^{\bullet+}$; HRMS (ESI$^+$) Calculated for $C_{18}H_{15}N_2O_4^+$ $[M+H]^+$ 323.1032, found 323.1043.

5,5',6,6'-tetramethoxyindigo 1c

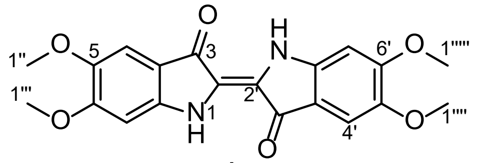

1c

Following General Procedure A, to a solution of 2-nitroveratraldehyde **8c** (2.956 g, 14.00 mmol) in acetone (25 mL) was added NaOH (1 M, 12.7 mL) dropwise over 10 min at rt, then stirred for 20 h. The precipitate was filtered, rinsed with H_2O (4 x 20 mL) and EtOAc (6 x 20 mL), recrystallised (EtOBz, 63 mL), filtered and rinsed with EtOAc (5 x 20 mL) and boiling acetone (2 x 20 mL) to yield 5,5',6,6'-tetramethoxyindigo **1c** as a purple solid (603 mg, 22%). Spectral data matched previously reported values.[13] mp >250 °C; FTIR (neat): ν_{max} 3381 (b/w), 2840 (w), 1612 (s), 1589 (m), 1483 (s), 1462 (m), 1439 (s), 1204 (s), 1142 (s), 1119 (s), 1056 (s), 1021 (s), 840 (s), 651 (m); ^{1}H NMR (400 MHz, DMSO-d_6, 80 °C) δ 9.99 (bs, 2H, H1 & H1'), 7.02 (s, 2H, H4 & H4'), 6.96 (s, 2H, H7 & H7'), 3.85 (s, 6H, H1''' & H1'''''), 3.76 (s, 6H, H1'' & H1''''); LRMS (EI) m/z 367 (43%) $[M-CH_3]^{\bullet+}$, 382 (100%) $[M]^{\bullet+}$; HRMS (ESI$^+$) Calculated for $C_{20}H_{18}N_2NaO_6^+$ $[M+Na]^+$ 405.1063, found 405.1046.

5,5'-diphenylindigo 1d

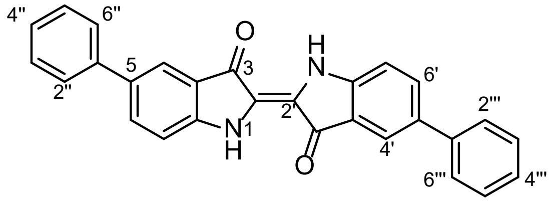

1d

Following General Procedure A, to a solution of 2-nitro-5-phenylbenzaldehyde **8d** (1.330 g, 5.85 mmol) in acetone (10.6 mL) was added NaOH (1 M, 5.3 mL) over 5 min at 2 °C. The ice bath was removed and the reaction stirred at rt for 20 h, then diluted with H_2O (20 mL) and filtered. The precipitate was rinsed with H_2O (5 x 20 mL) and EtOAc (5 x 20 mL), air-dried and the crude material was recrystallised (EtOBz, 57 mL). The solid was filtered and washed with EtOAc (3 x 50 mL) and boiling acetone (7 x 20 mL) to yield 5,5'-diphenylindigo **1d** as a fluffy purple solid (447 mg, 36%). The IR and LRMS data matched reported values,[14, 15] however HRMS and ^{1}H NMR data were not reported. mp >250 °C; FTIR (neat): ν_{max} 3373 (b/m), 1621 (s), 1588 (m), 1455 (m), 1429 (m), 1262 (m) 1137 (s), 825 (m), 756 (s); ^{1}H NMR (400 MHz, DMSO-d_6, 90 °C) δ 10.32 (bs, 2H, H1 & H1') 7.91 – 7.81 (m, 4H, H4 & H4', H6 & H6'), 7.68 (dt, J = 7.0, 1.3 Hz, 4H, H2'' & H6'' & H2''' & H6'''), 7.51 – 7.42 (m, 6H; H7 & H7', H3'' & H5'' & H3''' & H5'''), 7.35 (tt, J = 7.3, 1.3 Hz, 2H, H4'' & H4'''); LRMS (EI) m/z 414 (100%) $[M]^{\bullet+}$; HRMS (ESI$^+$) Calculated for $C_{28}H_{19}N_2O_2^+$ $[M+H]^+$ 415.1441, found 415.1442.

6,6'-diphenylindigo 1e

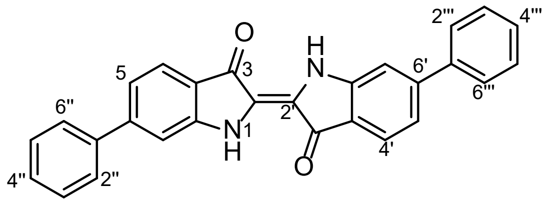

1e

Following General Procedure A, to a solution of 2-nitro-4-phenylbenzaldehyde **8e** (1.230 g, 5.41 mmol) in acetone (9.8 mL) was added NaOH (1 M, 4.9 mL) dropwise over 20 min at 2 °C. The ice bath was removed and the reaction stirred at rt for 20 h. The precipitate was filtered and rinsed with H_2O (5 x 20 mL) and EtOAc (5 x 20 mL). The electric blue solid was recrystallised (EtOBz, 35 mL), filtered and rinsed with EtOAc (3 x 20 mL) and boiling acetone (7 x 20 mL) to yield 6,6'-diphenylindigo **1e** as fine purple needles (321 mg, 28%). Synthesised previously,[3] however only FTIR and elemental analysis were reported. mp >250 °C; FTIR (neat): ν_{max} 3303 (b/m), 1611 (s), 1583 (s), 1427 (s), 1136 (s), 1110 (s), 1068 (s), 750 (s), 693 (s), 648 (s), 587 (s), 533 (s); ^{1}H NMR (400 MHz, DMSO-d_6, 80 °C) δ 10.30 (bs, 2H, H1 & H1'), 7.73 – 7.68 (m, 6H, H4 & H4', H2'' & H6'' & H2''' & H6'''), 7.64 (d, J = 1.5 Hz, 2H, H7 & H7'), 7.53 (t, J = 7.5 Hz, 4H, H3'' & H5'' & H3''' & H5'''), 7.45 (tt, J = 7.3, 1.5

Hz, 2H, H4'' & H4'''), 7.27 (dd, *J* = 8.0, 1.5 Hz, 1H, H5 & H5'); LRMS (EI) *m/z* 414 (100%) $[M]^{\bullet+}$; HRMS (ESI^+) Calculated for $C_{28}H_{19}N_2O_2^+$ $[M+H]^+$ 415.1447, found 415.1465.

5,5'-dibromoindigo 1f

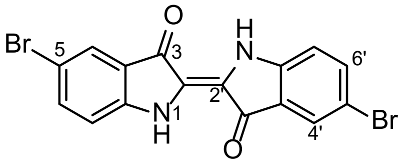

1f

Alternatively, Following General Procedure A, to a solution of 5-bromo-2-nitrobenzaldehyde **8f** (4.037 g, 17.6 mmol) in acetone (40 mL) was added NaOH (1 M, 20 mL) dropwise over 20 min at 2 °C, then warmed to rt and stirred for 16 h. The precipitate was filtered, rinsed with H_2O (5 x 50 mL), EtOAc (50 mL) and EtOH (2 x 50 mL) to yield 5,5'-dibromoindigo **1f** as a blue solid (1.295 g, 35%). Spectral data matched previously reported values.[16] mp >250 °C; FTIR (neat) ν_{max} 3278 (b/w), 1627 (s), 1604 (s), 1455 (s), 1439 (s), 1396 (m), 1255 (m), 1180 (s), 1113 (s), 611 (s); LRMS (EI) *m/z* 418 (52%) $[^{79}Br, ^{79}Br, M]^{\bullet+}$, 420 (100%) $[^{79}Br, ^{81}Br, M]^{\bullet+}$, 422 (53%) $[^{81}Br, ^{81}Br, M]^{\bullet+}$; HRMS ($ESI^+$) Calculated for $C_{16}H_9{}^{79}Br_2N_2O_2^+$ $[M+H]^+$ 418.9031, found 418.9043.

6,6'-dibromoindigo 1g

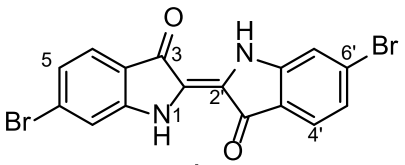

1g

Following General Procedure A, to a solution of 4-bromo-2-nitrobenzaldehyde **8g** (1.627 g, 7.07 mmol) in acetone (12.5 mL) was added NaOH (1 M, 6.5 mL) dropwise over 20 min at 2 °C. Upon addition, the ice bath was removed and the reaction stirred for 20 h at rt. The precipitate was filtered and rinsed with H_2O (5 x 20 mL) and EtOAc (4 x 20 mL) to yield 6,6'-dibromoindigo **1g** as a purple solid (474 mg, 32%). Spectral data matched previously reported values.[17] mp >250 °C; FTIR (neat): ν_{max} 3383 (b/m), 1632 (s), 1611 (s), 1577 (m), 1438 (m), 1312 (s), 1203 (s); LRMS (ESI^+) *m/z* 418 (50%) $[^{79}Br, ^{79}Br, M+H]^+$, 420 (100%) $[^{79}Br, ^{81}Br, M+H]^+$, 422 (49%) $[^{81}Br, ^{81}Br, M+H]^+$; HRMS ($ESI^-$) Calculated for $C_{16}H_7N_2O_2{}^{79}Br_2^-$ $[M-H]^-$ 416.8874, found 416.8863.

2.2.3 The synthesis of 5,5'-dinitroindigo 1h

3-acetoxy-5-nitroindole 10

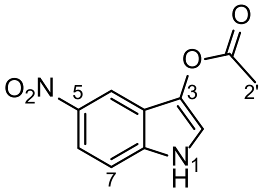
10

To a solution of 5-nitroindole **9** (3.405 g, 21.0 mmol) and NaOH (848 mg, 21.2 mmol) in MeOH (70 mL) was added I_2 (5.337 g, 21.0 mmol) and a solution of KI (3.490 g, 21.0 mmol) in H_2O (7 mL) and the reaction stirred at rt. After 5 h, H_2O (140 mL) was added and the 5-nitro-3-iodoindole precipitate collected by vacuum filtration, rinsed with H_2O (3 x 50 mL), air dried for 1 h and transferred to a RBF for immediate use. Silver acetate (6.993 g, 41.9 mmol) and AcOH (110 mL) were added and the reaction heated at 85 – 90 °C for 16 h. The reaction was cooled to rt, the excess AgOAc and AgI precipitate removed by vacuum filtration and the filtrate was concentrated *in vacuo*. The crude material was purified by column chromatography (120 g silica, 20% hex/CH_2Cl_2 → 100% CH_2Cl_2) to yield 3-acetoxy-5-nitroindole **10** as a yellow solid (2.677 g, 58%). Spectral data differed from reported values (see section S4.1, Table S1).[18] mp 139 – 143 °C; R_f (30% EtOAc/hex) = 0.20; FTIR (thin film): ν_{max} 3284 (b/m), 1726 (m), 1625 (m), 1517 (m), 1476 (m), 1371 (m), 1331 (s), 1252 (s), 1221 (s), 1120 (m), 1098 (w), 737 (m); ^{1}H NMR (500 MHz, $CDCl_3$) δ 8.57 (dd, *J* = 2.3 Hz, 1H, H4), 8.27 (bs, 1H, H1), 8.13 (dd, *J* = 9.0, 2.2 Hz, 1H, H6), 7.56 (d, *J* = 2.6 Hz, 1H, H2), 7.39 (d, *J* = 9.0 Hz, 1H, H7), 2.41 (s, 3H, H2'); ^{13}C NMR (101 MHz, $CDCl_3$) δ 168.4 (C1'), 141.9 (C5), 135.5 (C7a), 132.0 (C3), 119.5 (C3a), 118.3 (C6), 116.4 (C2), 115.3 (C4), 111.6 (C7), 20.9 (C2'); LRMS (ESI$^-$) *m/z* 219 (100%) [M-H]$^-$; HRMS (ESI$^-$) Calculated for $C_{10}H_7N_2O_4^-$ [M-H]$^-$ 219.0406, found 219.0397.

The synthesis of Boc-protected 5,5'-dinitroindigos

Following a modified procedure,[19] to a solution of 3-acetoxy-5-nitroindole **10** (1.115 g, 5.06 mmol) in MeOH (96 mL) was added a solution of NH_4OAc (3.13 g, 40.6 mmol) in H_2O (24 mL) and stirred at rt for 5 d. The crude 5,5'-dinitroindigo **1h** precipitate was filtered, rinsed with H_2O (5 x 20 mL) and EtOAc (3 x 40 mL), and air-dried. The crude 5,5'-dinitroindigo **1h** (701 mg, 2.00 mmol) was suspended in CH_2Cl_2 (15 mL) and DMAP (244 mg, 2.0 mmol) was added followed by a solution of Boc_2O (2.18 g, 10.0 mmol) in CH_2Cl_2 (5 mL) and stirred at rt under a N_2 atmosphere for 39 h. The reaction was washed with HCl (1 M, 3 x 10 mL), $NaHCO_3$ (sat., 20 mL), dried ($MgSO_4$) and concentrated *in vacuo*. The residue was adsorbed onto silica

(15 g) and subjected to column chromatography (150 g silica, 10% → 40% EtOAc/hex) to generate two major fractions (F_1 and F_2). Fraction F_2 contained pure *N*,*N'*-diBoc-5,5'-dinitroindigo **12**, isolated as a pink solid (364 mg, 26%) while adsorption of F_1 onto silica (2 g) and column chromatography (20 g silica, 5% → 20% EtOAc/hex) yielded *N*,*N'*,*O*,*O'*-tetraBoc-5,5'-dinitrobiindole-3,3'-diol **11** as a pale yellow solid (30 mg, 1%).

di-*tert*-butyl 3,3'-bis((*tert*-butoxycarbonyl)oxy)-5,5'-dinitro-1*H*,1'*H*-[2,2'-biindole]-1,1'-dicarboxylate **11**

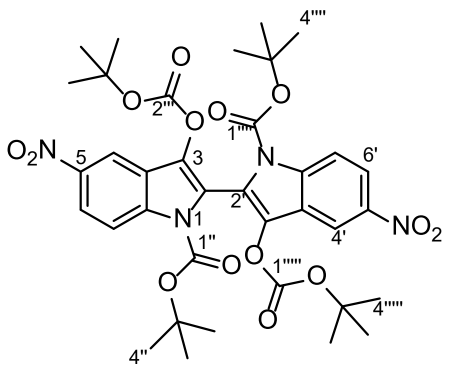

11

mp decomp. at >155 °C; R_f (20% EtOAc/hex) = 0.57; FTIR (thin film): ν_{max} 2980 (w), 2934 (w), 2870 (w), 1766 (m), 1740 (s), 1525 (m), 1450 (m), 1371 (m), 1344 (s), 1248 (s), 1221 (s), 1141 (s), 1126 (s), 1053 (m); ^{1}H NMR (400 MHz, $CDCl_3$) δ 8.53 (d, *J* = 9.3 Hz, 2H, H7 & H7'), 8.45 (d, *J* = 2.2 Hz, 2H, H4 & H4'), 8.30 (dd, *J* = 9.3, 2.3 Hz, 2H, H6 & H6'), 1.42 (s, 18H, H4''' & H4'''''), 1.25 (s, 18H, H4'' & H4''''); ^{13}C NMR (101 MHz, $CDCl_3$) δ 150.1 (C1''' & C1'''''), 148.9 (C1'' & C1''''), 143.8 (C5 & C5'), 137.5 (C7a & C7a'), 134.8 (C3 & C3'), 122.4 (C3a & C3a'), 121.1 (C6 & C6'), 119.7 (C2 & C2'), 116.3 (C7 & C7'), 114.9 (C4 & C4'), 85.5 (C3'' & C3''''), 84.9 (C3''' & C3'''''), 27.5 (C4''' & C4'''''), 27.3 (C4'' & C4''''); LRMS (ESI$^+$) *m/z* 777 (100%) $[M+Na]^+$; HRMS (ESI$^+$) Calculated for $C_{36}H_{42}N_4NaO_{14}^+$ $[M+Na]^+$ 777.2595, found 777.2603.

di-*tert*-butyl (*E*)-5,5'-dinitro-3,3'-dioxo-[2,2'-biindolinylidene]-1,1'-dicarboxylate 12

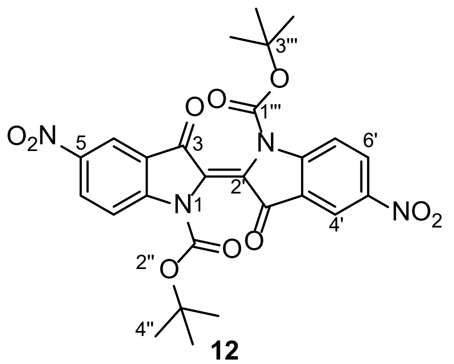

12

All spectral data matched previously reported values.[9] mp decomp. at >155 °C; R_f (20% EtOAc/hex) = 0.41; FTIR (thin film): ν_{max} 2979 (w), 2929 (w), 2855 (w), 1741 (m), 1688 (w), 1611 (m), 1464 (m), 1331 (s), 1250 (s), 1145 (s), 1091 (s), 815 (w); ^{1}H NMR (400 MHz, $CDCl_3$) δ 8.64 (d, *J* = 2.3 Hz, 2H, H4 & H4'), 8.53 (dd, *J* = 9.1, 2.4 Hz, 2H, H6 & H6'), 8.17 (d, *J* = 9.1 Hz, 2H, H7 & H7'), 1.66 (s, 18H, H4'' & H4'''); ^{13}C NMR (101 MHz, $CDCl_3$) δ 181.5 (C3 & C3'), 151.9 (C7a & C7a'), 148.8 (C1'' & C1'''), 144.6 (C5 & C5'), 130.9 (C6 & C6'), 125.2 (C2 & C2'), 122.9 (C3a & C3a'), 120.2 (C4 & C4'), 117.1 (C7 & C7'), 86.7 (C3'' & C3'''), 28.1 (C4'' & C4'''); LRMS (ESI$^+$) *m/z* 575 (100%) $[M+Na]^+$; HRMS (ESI$^+$) Calculated for $C_{26}H_{24}N_4NaO_{10}^+$ $[M+Na]^+$ 575.1390, found 575.1392.

5,5'-dinitroindigo 1h

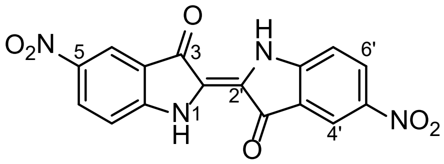

1h

A solution of *N*,*N'*-diBoc-5,5'-dinitroindigo **12** (357 mg, 0.647 mmol) in 1,2-dichlorobenzene (12.9 mL) in a conical flask equipped with a condenser was heated at reflux for 30 min under a N_2 atmosphere. The reaction was cooled to rt, the precipitate was filtered, rinsed with EtOAc (6 x 10 mL) and CH_2Cl_2 (3 x 20 mL) and dried *in vacuo* to furnish 5,5'-dinitroindigo **1h** as a fluffy purple solid (187 mg, 82%). Reported previously,[9, 20-22] however no spectral data was provided. mp >250 °C; FTIR (neat): ν_{max} 3381 (b/m), 3111 (w), 1659 (m), 1616 (m), 1590 (m), 1522 (m), 1466 (m), 1405 (m), 1321 (s), 1270 (m), 1186 (m), 1150 (s), 1122 (s), 1102 (s), 830 (m), 744 (m), 695 (s); ^{1}H NMR (400 MHz, DMSO-d_6, 90 °C) δ 11.20 (bs, 2H, H1 & H1'), 8.48 – 8.33 (m, 4H, H4 & H4', H6 & H6'), 7.54 (d, *J* = 8.9 Hz, 2H, H7 & H7'); LRMS (EI) *m/z* 352 (100%) $[M]^{\bullet+}$; HRMS (ESI$^-$) Calculated for $C_{16}H_7N_4O_6^-$ $[M-H]^-$ 351.0366, found 351.0354.

2.2.4 The synthesis of 6,6'-dinitroindigo 1i

The synthesis of Boc-protected 6,6'-dinitroindigos

To a solution of 2,4-dinitrobenzaldehyde **8h** (13.728 g, 70.0 mmol) in acetone (130 mL) was added NaOH (1 M, 65 mL) dropwise over 20 min at 2 °C. Upon addition, the ice bath was removed and the reaction stirred for 20 h at rt. The precipitate was filtered, rinsed with H_2O (5 x 50 mL) and EtOAc (5 x 50 mL), and air dried. The crude solid was recrystallised (EtOBz, 200 mL), filtered, rinsed with EtOAc (5 x 30 mL) and dried *in vacuo*. To a suspension of the crude 6,6'-dinitroindigo **1i** (1.901 g, 5.40 mmol) in CH_2Cl_2 (54 mL) was added Boc_2O (2.593 g, 11.9 mmol) and DMAP (66 mg, 0.54 mmol, 10 mol%), and stirred at rt for 2 d under a N_2 atmosphere. The reaction mixture was washed with HCl (1 M, 3 x 10 mL), $NaHCO_3$ (sat., 10 mL) and brine (10 mL), dried ($MgSO_4$) and concentrated *in vacuo*. The crude solid was adsorbed onto silica (30 g) and subjected to column chromatography (300 g silica, 10% → 30% EtOAc/hex) to yield two major fractions (F_1 and F_2). Recrystallisation of F_1 (CH_2Cl_2/hex) yielded *N*,*N'*,*O*,*O'*-tetraBoc-6,6'-dinitrobiindole-3,3'-diol **13** as yellow crystals (183 mg, <1%). Recrystallisation of F_2 (CH_2Cl_2/hex) furnished *N*,*N'*-diBoc-6,6'-dinitroindigo **14** (691 mg) as purple crystals, and adsorption of the mother liquor onto silica (10 g) and column

chromatography (100 g silica, 10% → 30% EtOAc/hex) yielded additional **14** (352 mg) as a purple solid (total 1.043 g, 2.7%).

di-*tert*-butyl 3,3'-bis((*tert*-butoxycarbonyl)oxy)-6,6'-dinitro-1*H*,1'*H*-[2,2'-biindole]-1,1'-dicarboxylate **13**

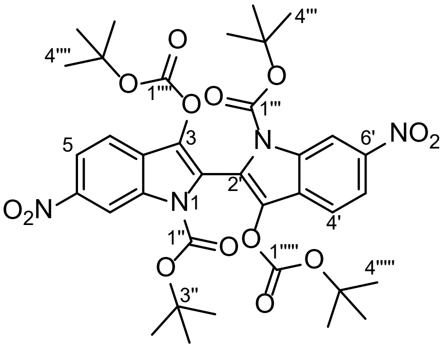

13

mp decomp. at >155 °C; R_f (20% EtOAc/hex) = 0.69; FTIR (thin film): ν_{max} 3135 (w), 2980 (w), 1766 (m), 1739 (m), 1525 (m), 1369 (m), 1321 (s), 1270 (m), 1246 (s), 1221 (s), 1144 (s), 1121 (s), 1100 (s), 1082 (s), 1050 (s), 1028 (s), 900 (w), 872 (w), 854 (w), 829 (w), 810 (w), 756 (w), 731 (w); ^{1}H NMR (400 MHz, $CDCl_3$) δ 9.34 (d, *J* = 2.0 Hz, 2H, H7 & H7'), 8.21 (dd, *J* = 8.7, 2.0 Hz, 2H, H5 & H5'), 7.62 (d, *J* = 8.8 Hz, 2H, H4 & H4'), 1.41 (s, 18H, H3'''' & H3'''''), 1.31 (s, 18H, H4'' & H4'''); ^{13}C NMR (101 MHz, $CDCl_3$) δ 150.1 (C1'''' & C1'''''), 148.6 (C1'' & C1'''), 146.2 (C6 & C6'), 134.0 (C3 & C3'), 133.5 (C7a & C7a'), 126.7 (C3a & C3a'), 121.7 (C2 & C2'), 118.7 (C4 & C4'), 118.3 (C5 & C5'), 112.5 (C7 & C7'), 85.6 (C3'''' & C3'''''), 84.8 (C3'' & C3'''), 27.6 (C4'' & C4'''), 27.3 (C4'''' & C4'''''); LRMS (ESI$^+$) *m/z* 777 (100%) [M+Na]$^+$; HRMS (ESI$^+$) Calculated for $C_{36}H_{42}N_4NaO_{14}^+$ [M+Na]$^+$ 777.2595, found 777.2612.

di-*tert*-butyl (*E*)-6,6'-dinitro-3,3'-dioxo-[2,2'-biindolinylidene]-1,1'-dicarboxylate 14

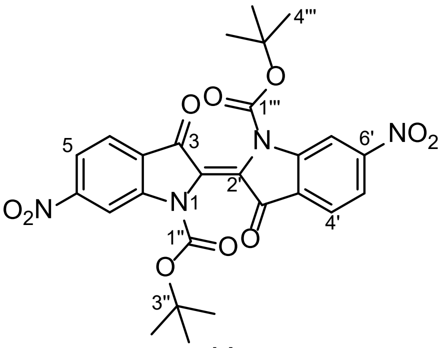

14

A HRMS of **14** is the only spectrum previously reported for this compound.[9] mp decomp. at >155 °C; FTIR (thin film): ν_{max} 3135 (w), 2979 (w), 2932 (w), 1743 (m), 1685 (m), 1620 (w), 1537 (s), 1436 (m), 1347 (s), 1335 (s), 1283 (m), 1242 (m), 1179 (m), 1143 (s), 1097 (m), 1067 (w), 1011 (w), 826 (w), 735 (w); ^{1}H NMR (400 MHz, $CDCl_3$) δ 8.88 (d, *J* = 1.9 Hz, 2H, H7 & H7'), 8.10 (dd, *J* = 8.3, 1.9 Hz, 2H, H5 & H5'), 7.93 (d, *J* = 8.3 Hz, 2H, H4 & H4'), 1.70 (s, 18H, H4'' & H4'''); ^{13}C NMR (101 MHz, $CDCl_3$) δ 181.9 (C3 & C3'), 152.3 (C6 & C6'), 148.8 (C1'' & C1'''), 148.5 (C7a & C7a'), 126.4 (C3a & C3a'), 125.0 (C4 & C4'), 124.7 (C2 & C2'), 119.6 (C5 & C5'), 112.7 (C7 & C7'), 86.6 (C3'' & C3'''), 28.1 (C4'' & C4'''); LRMS (ESI$^+$) *m/z* 575 (100%) [M+Na]$^+$; HRMS (ESI$^+$) Calculated for $C_{26}H_{24}N_4NaO_{10}^+$ [M+Na]$^+$ 575.1390, found 575.1378.

6,6'-dinitroindigo 1i

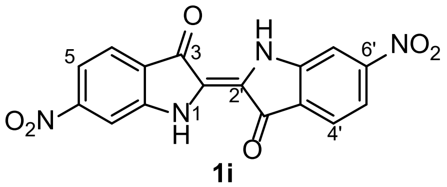

1i

A solution of *N*,*N'*-diBoc-6,6'-dinitroindigo **14** (430 mg, 0.775 mmol) in 1,2-dichlorobenzene (15.5 mL) in a conical flask equipped with a condenser was heated at reflux for 30 min under a N_2 atmosphere. The reaction was cooled to rt, the precipitate filtered, rinsed with EtOAc (3 x 20 mL) and CH_2Cl_2 (3 x 20 mL) and dried *in vacuo* to furnish 6,6'-dinitroindigo **1i** as a fluffy purple solid (261 mg, 96%). Spectral data matched previously reported values.[23] mp >250 °C; FTIR (neat): ν_{max} 3322 (b/w), 1640 (s), 1618 (s), 1543 (s), 1442 (s), 1347 (s), 1148 (b/s), 1081 (s), 828 (s), 732 (s), 531 (s); ^{1}H NMR (400 MHz, DMSO-d_6, 80 °C) δ 10.98 (bs, 2H, H1 & H1'), 8.20 (d, *J* = 2.0 Hz, 2H, H7 & H7'), 7.89 (d, *J* = 8.4 Hz, 2H, H4 & H4'), 7.75 (dd, *J* = 8.3, 2.1 Hz, 2H, H5 & H5'); LRMS (ESI$^-$) *m/z* 351 (100%) [M-H]$^-$; HRMS (ESI$^-$) Calculated for $C_{16}H_7N_4O_6^-$ [M-H]$^-$ 351.0366, found 351.0364.

S2.3 The Cascade Reactions of Substituted Indigos

2.3.1 The allylation of 5,5'-dimethoxyindigo 1a (2 min)

Following General Procedure B, a suspension of 5,5'-dimethoxyindigo **1a** (160 mg, 0.496 mmol) in anhydrous DMF (20 mL) was sonicated for 60 min under a N_2 atmosphere. The suspension was transferred to a flask containing pre-dried Cs_2CO_3 (650 mg, 1.995 mmol) and activated 3 Å M.S. (1 g) and plunged into a pre-heated oil bath at 85 – 88 °C under N_2 with stirring. After 1 h, the N_2 flow was cut and allyl bromide (0.22 mL, 310 mg, 2.56 mmol) was added and stirred for 2 min. The reaction was quenched with ice (100 g) and diluted to 200 mL with brine, extracted with EtOAc (3 x 70 mL) and the combined organic phases washed with H_2O (5 x 25 mL) and brine (2 x 20 mL), dried ($MgSO_4$) and concentrated *in vacuo*. The residue was adsorbed onto silica (4 g) and subjected to column chromatography (60 g silica, 10% → 90% EtOAc/hex) to yield two fractions (F_1 and F_2). Fraction F_1 contained 1-allyl-3-(allyloxy)-5,5'-dimethoxy-1*H*,3'*H*-[2,2'-biindol]-3'-one **16** and column chromatography of F_2 (60 g silica, 50% CH_2Cl_2/hex → 5% EtOAc/CH_2Cl_2) yielded (*E*)-1-allyl-5,5'-dimethoxy-[2,2'-biindolinylidene]-3,3'-dione **15** and 1-allyl-10'-(allyloxy)-2',5-dimethoxy-8'*H*-spiro[indoline-2,9'-pyrido[1,2-*a*]indol]-3-one **17**.

(*E*)-1-allyl-5,5'-dimethoxy-[2,2'-biindolinylidene]-3,3'-dione **15**

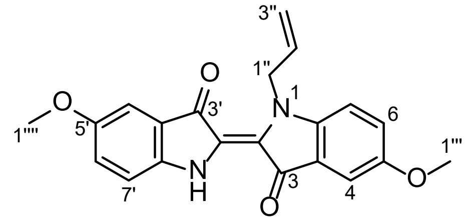

15

Isolated as a papery blue solid (11 mg, 6%). mp >250 °C; R_f (20% EtOAc/hex) = 0.26; FTIR (thin film): ν_{max} 3312 (b/w), 2942 (w), 2834 (w), 1631 (m), 1487 (s), 1437 (m), 1383 (m), 1328 (m), 1275 (m), 1229 (m), 1170 (m), 1106 (m), 1070 (m), 1036 (m), 800 (m), 673 (m); ^{1}H NMR (500 MHz, $CDCl_3$) δ 10.48 (bs, 1H, H1'), 7.15 (d, *J* = 2.1 Hz, 1H, H4), 7.15 – 7.11 (m, 2H, H6, H7'), 7.07 (dd, *J* = 8.6, 2.6 Hz, 1H, H6'), 7.00 (d, *J* = 8.5 Hz, 1H, H7), 6.90 (d, *J* = 8.6 Hz, 1H, H7'), 5.90 (ddt, *J* = 17.0, 10.4, 5.2 Hz, 1H, H2''), 5.16 – 5.07 (m, 2H, H3''), 5.05 (dt, *J* = 5.1, 1.6 Hz, 2H, H1''), 3.82 (s, 3H, H1'''), 3.79 (s, 3H, H1''''); ^{13}C NMR (126 MHz, $CDCl_3$) δ 189.3 (C3), 187.0 (C3'), 154.6 (C5), 154.5 (C5'), 148.0 (C7a), 146.4 (C7a'), 133.3 (C2''), 126.5 (C2'), 125.1 (C6'), 125.0 (C7), 123.8 (C2), 121.2 (C3a), 120.3 (C3a'), 116.8 (C3''), 112.9 (C7'), 112.4 (C7), 106.3 (C4'), 105.2 (C4), 55.9 (C1'''), 55.8 (C1''''), 50.0 (C1''); LRMS (ESI^+) *m/z* 363 (100%) $[M+H]^+$; HRMS (ESI^+) Calculated for $C_{21}H_{19}N_2O_4^+$ $[M+H]^+$ 363.1345, found 363.1341.

1-allyl-3-(allyloxy)-5,5'-dimethoxy-1*H*,3'*H*-[2,2'-biindol]-3'-one **16**

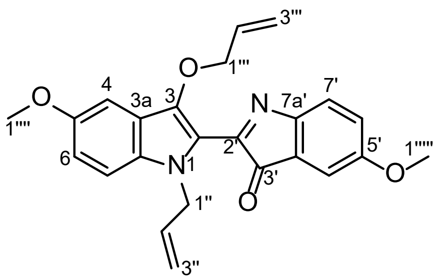

16

Isolated as a purple wax (5.5 mg, 3%). R_f (20% hex/CH_2Cl_2) = 0.69; FTIR (thin film): ν_{max} 1737 (m), 1620 (m), 1550 (m), 1479 (s), 1281 (m), 1226 (s), 1026 (m), 926 (w), 765 (w); ^{1}H NMR (500 MHz, $CDCl_3$) δ 7.28 (d, *J* = 8.3 Hz, 1H, H7'), 7.22 (d, *J* = 9.0 Hz, 1H, H7), 7.13 (d, *J* = 2.6 Hz, 1H, H4'), 7.08 (d, *J* = 2.4 Hz, 1H, H4), 6.99 – 6.96 (m, 2H, H6, H6'), 6.14 (ddt, *J* = 17.2, 10.4, 5.6 Hz, 1H, H2'''), 5.89 (ddt, *J* = 17.1, 10.3, 5.2 Hz, 1H, H2''), 5.41 (appt. dq, *J* = 17.2, 1.6 Hz, 1H, H3'''a), 5.25 (appt. dq, *J* = 10.5, 1.4 Hz, 1H, H3'''b), 5.11 – 5.00 (m, 3H, H1'', H3''a), 4.89 (appt. dq, *J* = 16.9, 1.6 Hz, 1H, H3''b), 4.78 (dt, *J* = 5.6, 1.4 Hz, 2H, H1'''), 3.86 (s, 3H, H1''''), 3.85 (s, 3H, H1'''''); ^{13}C NMR (126 MHz, $CDCl_3$) δ 191.3 (C3'), 159.9 (C5'), 156.4 (C2'), 154.1 (C5), 153.6 (C7a'), 144.1 (C3), 134.2 (C2'''), 134.13 (C2''), 134.08 (C7a), 123.4 (C3a'), 122.5 (C7'), 120.7 (C3a), 120.2 (C6), 119.4 (C2), 117.8 (C3'''), 116.8 (C6'), 116.3 (C3''), 111.8 (C7), 111.0 (C4'), 100.1 (C4), 76.2 (C1'''), 55.8 (C1'''' or C1'''''), 47.0 (C1''); LRMS (ESI^+) *m/z* 403 (100%) $[M+H]^+$; HRMS (ESI^+) Calculated for $C_{24}H_{23}N_2O_4^+$ $[M+H]^+$ 403.1658, found 403.1645.

1-allyl-10'-(allyloxy)-2',5-dimethoxy-8'*H*-spiro[indoline-2,9'-pyrido[1,2-*a*]indol]-3-one **17**

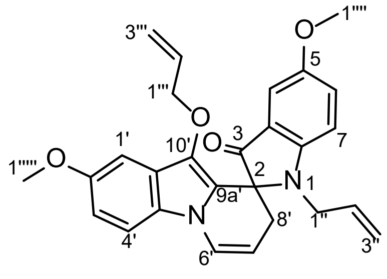

17

Isolated as a yellow solid (84 mg, 38%). mp 86 – 89 °C; R$_f$ (20% hex/CH_2Cl_2) = 0.57; FTIR (thin film): ν_{max} 1692 (m), 1495 (s), 1450 (m), 1338 (m), 1270 (m), 1226 (m), 1159 (m), 1030 (m); ^{1}H NMR (400 MHz, $CDCl_3$) δ 7.28 (d, *J* = 9.0 Hz, 1H, H4'), 7.18 – 7.13 (m, 2H, H6, H6'), 7.12 (d, *J* = 2.6 Hz, 1H, H4), 6.94 (d, *J* = 2.3 Hz, 1H, H1'), 6.89 (dd, *J* = 8.9, 2.4 Hz, 1H, H3'), 6.68 (d, *J* = 8.9 Hz, 1H, H7), 5.76 – 5.61 (m, 2H, H2'', H2'''), 5.42 (ddd, *J* = 7.8, 5.6, 3.1 Hz, 1H, H7'), 5.10 (appt. dq, *J* = 17.2, 1.7 Hz, 1H, H3''a), 5.07 – 4.98 (m, 3H, H3''b, H3'''), 4.24 (ddt, *J* = 12.1, 6.0, 1.3 Hz, 1H, H1'''a), 4.17 (ddt, *J* = 12.1, 5.6, 1.4 Hz, 1H, H1'''b), 3.82 (s, 3H, H1'''''), 3.79 (s, 3H, H1''''), 3.87 – 3.71 (m, 2H, H1''), 2.89 (appt. dt, *J* = 17.4, 3.0 Hz, 1H, H8'a), 2.43 (ddd, *J* = 17.5, 5.6, 0.9 Hz, 1H, H8'b); ^{13}C NMR (101 MHz, $CDCl_3$) δ 200.7 (C3), 155.7 (C7a), 154.5 (C2'), 152.4 (C5), 135.6 (C10'), 134.3 (C2''), 133.7 (C2'''), 128.2 (C6), 127.5 (C4a'), 121.7 (C10a'), 121.6 (C6'), 119.9 (C9a'), 118.8 (C3a), 117.4 (C3'''), 115.9 (C3''), 113.2 (C3'), 110.4 (C7), 109.8 (C4'), 105.3 (C4), 104.4 (C7'), 100.5 (C1'), 74.5 (C1'''), 67.0 (C2/C9'), 56.0 (C1''''), 55.9 (C1'''''), 47.2 (C1''), 31.9 (C8'); LRMS (ESI$^+$) *m/z* 443 (100%) [M+H]$^+$; HRMS (ESI$^+$) Calculated for $C_{27}H_{27}N_2O_4^+$ [M+H]$^+$ 443.1971, found 443.1967.

2.3.2 The allylation of 5,5'-dimethoxyindigo 1a (5 min)

Following General Procedure B, a suspension of 5,5'-dimethoxyindigo **1a** (160 mg, 0.500 mmol) in anhydrous DMF (20 mL) was sonicated for 60 min under a N_2 atmosphere. The suspension was transferred to a flask containing pre-dried Cs_2CO_3 (653 mg, 2.00 mmol) and activated 3 Å M.S. (1 g) and plunged into a pre-heated oil bath at 85 – 88 °C under N_2 with stirring. After 1 h, the N_2 flow was cut and allyl bromide (0.22 mL, 310 mg, 2.56 mmol) was added and stirred for 5 min, the reaction quenched with ice (100 g), diluted to 200 mL with brine, extracted with EtOAc (4 x 70 mL) and the combined organic phases washed with H_2O (5 x 25 mL) and brine (2 x 20 mL), dried ($MgSO_4$) and concentrated *in vacuo*. The residue was adsorbed onto silica (4 g) and subjected to column chromatography (60 g silica, 20% EtOAc/hex) to yield 1-allyl-3-(allyloxy)-5,5'-dimethoxy-1*H*,3'*H*-[2,2'-biindol]-3'-one **16** and 1-allyl-10'-(allyloxy)-2',5-dimethoxy-8'*H*-spiro[indoline-2,9'-pyrido[1,2-*a*]indol]-3-one **17**.

1-allyl-3-(allyloxy)-5,5'-dimethoxy-1*H*,3'*H*-[2,2'-biindol]-3'-one **16**

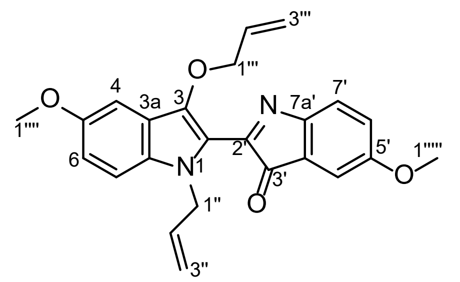

16

Isolated as a purple wax (6 mg, 3%). Spectral characteristics were identical to those observed previously (see section S2.3.1).

1-allyl-10'-(allyloxy)-2',5-dimethoxy-8'*H*-spiro[indoline-2,9'-pyrido[1,2-*a*]indol]-3-one **17**

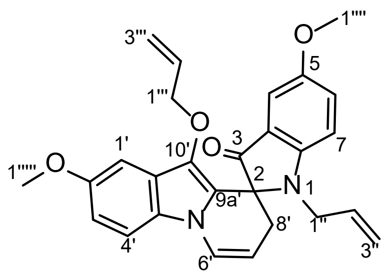

17

Isolated as a yellow powder (153 mg, 69%). Spectral characteristics were identical to those observed previously (see section S2.3.1).

2.3.3 The allylation of 5,5',6,6'-tetramethoxyindigo 1b

Following General Procedure B, a suspension of 5,5',6,6'-tetramethoxyindigo **1b** (191 mg, 0.500 mmol) in anhydrous DMF (20 mL) was sonicated for 60 min under a N_2 atmosphere. The suspension was transferred to a flask containing pre-dried Cs_2CO_3 (653 mg, 2.00 mmol) and activated 3 Å M.S. (1 g) and plunged into a pre-heated oil bath at 85 – 88 °C under N_2 with stirring. After 60 min, the N_2 flow was cut and allyl bromide (0.22 mL, 310 mg, 2.56 mmol) was added and stirred for 60 min. The reaction was quenched with ice (100 g), diluted to 200 mL with brine, extracted with EtOAc (4 x 50 mL) and the combined organic phases washed with H_2O (5 x 30 mL) and brine (2 x 25 mL), dried ($MgSO_4$) and concentrated *in vacuo*. The residue was adsorbed onto silica (5 g) and subjected to column chromatography (90 g silica, 10% → 100% EtOAc/hex) to generate three major fractions (F_1, F_2 and F_3). Fraction F_1 contained pure 1-allyl-10'-(allyloxy)-2',3',5,6-tetramethoxy-8'*H*-spiro[indoline-2,9'-pyrido[1,2-*a*]indol]-3-one **18** (74 mg) and PTLC of F_2 (50% EtOAc/hex, eluted 2x) yielded additional **18** (78 mg) as a yellow solid (total 152 mg, 61%). Fraction F_3 contained pure 1-allyl-10'-(allyloxy)-2',3',5,6-tetramethoxy-8'*H*-spiro[indoline-2,9'-pyrido[1,2-*a*]indol]-3-one **26**, isolated as an orange solid (26 mg, 10%).

1-allyl-10'-(allyloxy)-2',3',5,6-tetramethoxy-8'*H*-spiro[indoline-2,9'-pyrido[1,2-*a*]indol]-3-one **18**

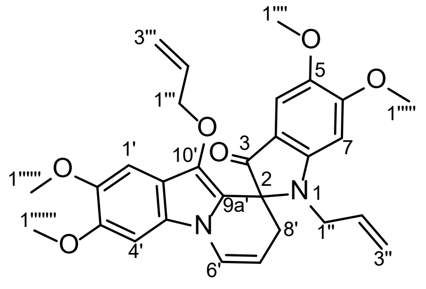

18

mp 127 – 128 °C; R_f = 0.39 (30% EtOAc/hex); FTIR (thin film): ν_{max} 2965 (w), 2834 (w), 1676 (m), 1622 (m), 1581 (w), 1487 (s), 1444 (m), 1386 (m), 1339 (m), 1286 (m), 1242 (s), 1224 (s), 1209 (s), 1171 (m), 1115 (m), 1023 (m), 841 (m); ^{1}H NMR (400 MHz, $CDCl_3$) δ 7.13 (ddd, *J* = 7.8, 2.9, 0.9 Hz, 1H, H6'), 7.09 (s, 1H, H4), 6.92 (s, 1H, H1'), 6.86 (s, 1H, H4'), 6.16 (s, 1H, H7), 5.82 – 5.64 (m, 2H, H2'', H2'''), 5.42 (ddd, *J* = 7.7, 5.6, 3.2 Hz, 1H, H7'), 5.13 (appt. dq, *J* = 17.1, 1.7 Hz, 1H, H3''a), 5.10 – 5.01 (m, 3H, H3''b, H3'''), 4.28 (ddt, *J* = 12.1, 5.9, 1.4 Hz, 1H, H1'''a), 4.18 (ddt, *J* = 12.1, 5.5, 1.5 Hz, 1H, H1'''b), 3.94 (s, 3H, H1'''''''), 3.92 (s, 3H, H1'''''), 3.89 (s, 3H, H1''''''), 3.86 (s, 3H, H1''''), 3.87 – 3.80 (m, 1H, H1''a), 3.78 (ddt, *J* = 17.5, 5.0, 1.8 Hz, 1H, H1''b), 2.89 (appt. dt, *J* = 17.5, 3.0 Hz, 1H, H8'a), 2.40 (ddd, *J* = 17.4, 5.6, 0.9 Hz, 1H, H8'b); ^{13}C NMR (101 MHz, $CDCl_3$) δ 198.4 (C3), 158.9 (C6), 157.3 (C7a), 148.0 (C3'), 145.5 (C2'), 143.1 (C5), 136.0 (C10'), 134.4 (C2''), 134.0 (C2'''), 126.8 (C4a'), 121.5 (C6'), 117.5 (C9a'), 117.2 (C3'''), 115.8 (C3''), 114.2 (C10a'), 110.2 (C3a), 105.1 (C4), 104.6 (C7'), 100.3 (C1'), 92.5 (C4'), 91.5 (C7), 74.6 (C1'''), 67.0 (C2/C9'), 56.4 (C1'''', C1''''''), 56.24 (C1'''''''), 56.15 (C1'''''), 47.1 (C1''), 31.9 (C8'); LRMS (ESI$^+$) *m/z* 503 (100%) $[M+H]^+$; HRMS (ESI$^+$) Calculated for $C_{29}H_{31}N_2O_6^+$ $[M+H]^+$ 503.2182, found 503.2201.

cisoid- 1-allyl-10'-(allyloxy)-2',3',5,6-tetramethoxy-8'*H*-spiro[indoline-2,9'-pyrido[1,2-*a*]indol]-3-one **26**

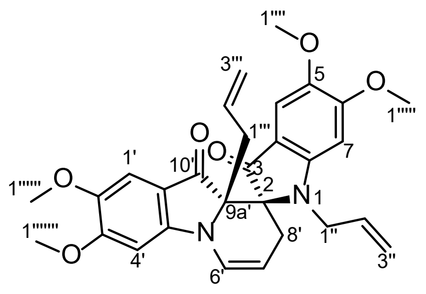

(±)-**26**

mp 146 – 147 °C; R_f = 0.27 (30% EtOAc/hex); FTIR (thin film): ν_{max} 2919 (w), 2850 (w), 1677 (m), 1618 (s), 1584 (m), 1495 (s), 1464 (m), 1443 (m), 1338 (m), 1278 (m), 1443 (s), 1215 (m), 1153 (m), 1132 (m), 1027 (m), 843 (w), 821 (w); ^{1}H NMR (400 MHz, $CDCl_3$) δ 7.12 (s, 1H, H4), 6.92 (s, 1H, H1'), 6.84 (dt, *J* = 7.7, 1.9 Hz, 1H, H6'), 6.39 (s, 1H, H4'), 6.14 (s, 1H, H7), 5.39 – 5.20 (m, 3H, H7', H2'', H2'''), 5.02 (ddt, *J* = 16.9, 2.4, 1.3 Hz, 1H, H3'''a), 4.94 (appt. dq, *J* = 17.3, 1.7 Hz, 1H, H3''a), 4.89 – 4.81 (m, 2H, H3''b, H3'''b), 4.05 – 3.98 (m, 4H, H1''a, H1'''''''), 3.85 (s, 3H, H1''''), 3.80 (s, 6H, H1'''''', H1'''''), 3.66 (ddt, *J* = 17.1, 6.1, 1.6 Hz, 1H, H1''b), 3.16 (ddt, *J* = 14.1, 6.8, 1.1 Hz, 1H, H1'''a), 2.94 (appt. dt, *J* = 19.3, 2.3 Hz, 1H, H8'a),

2.58 (ddt, *J* = 14.1, 7.7, 1.1 Hz, 1H, H1'''b), 2.06 (ddd, *J* = 19.3, 4.7, 1.8 Hz, 1H, H8'b); ^{13}C NMR (101 MHz, $CDCl_3$) δ 197.3 (C3), 196.2 (C10'), 158.2 (C3'), 158.04 (C6), 158.02 (C7a), 152.6 (C4a'), 143.9 (C2'), 143.8 (C5), 135.0 (C2''), 131.1 (C2'''), 122.1 (C6'), 119.1 (C3'''), 115.8 (C3''), 113.5 (C3a), 113.1 (C10a'), 104.7 (C1'), 104.3 (C7'), 103.8 (C4), 93.3 (C7), 90.7 (C4'), 73.4 (C9a'), 68.9 (C2/C9'), 56.4 (C1'''''''), 56.1 (C1'''', C1'''''), 55.9 (C1''''''), 47.8 (C1''), 34.7 (C1'''), 29.7 (C8'); LRMS (ESI$^+$) *m/z* 503 (100%) $[M+H]^+$; HRMS (ESI$^+$) Calculated for $C_{29}H_{31}N_2O_6^+$ $[M+H]^+$ 503.2182, found 503.2188.

2.3.4 The allylation of 6,6'-dimethoxyindigo 1c

Following General Procedure B, a suspension of 6,6'-dimethoxyindigo **1c** (161 mg, 0.500 mmol) in anhydrous DMF (20 mL) was sonicated for 60 min under a N_2 atmosphere. The suspension was transferred to a flask containing pre-dried Cs_2CO_3 (652 mg, 2.00 mmol) and activated 3 Å M.S. (1 g) and plunged into a pre-heated oil bath at 85 – 88 °C under N_2 with stirring. After 60 min, the N_2 flow was cut and allyl bromide (0.22 mL, 310 mg, 2.56 mmol) was added and stirred for 60 min. The reaction was quenched with ice (100 g) and diluted to 200 mL with brine, extracted with EtOAc (3 x 50 mL), and the combined organic phases washed with H_2O (5 x 25 mL) and brine (2 x 20 mL), dried ($MgSO_4$) and concentrated *in vacuo*. The residue was adsorbed onto silica (5 g) and subjected to column chromatography (90 g silica, 10% → 80% EtOAc/hex) to generate two fractions (F_1 and F_2). Fraction F1 contained pure 1-allyl-10'-(allyloxy)-3',6-dimethoxy-8'*H*-spiro[indoline-2,9'-pyrido[1,2-*a*]indol]-3-one **19** (75 mg), isolated as a yellow solid. Purification of F_2 by PTLC (15% EtOAc/hex, eluted 4x) yielded additional **19** (23 mg; total 96 mg, 43%, NMR corrected yield) and 1,9a'-diallyl-3',6-dimethoxy-8'*H*-spiro[indoline-2,9'-pyrido[1,2-*a*]indole]-3,10'(9a'*H*)-dione **27** (23 mg, 11%), isolated as an orange solid.

<u>1-allyl-10'-(allyloxy)-3',6-dimethoxy-8'*H*-spiro[indoline-2,9'-pyrido[1,2-*a*]indol]-3-one</u> **19**

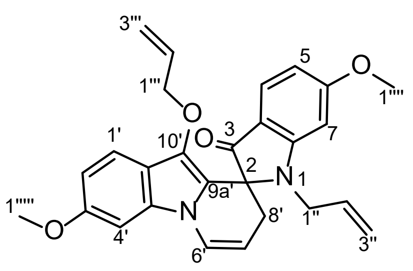

19

mp 66 – 70 °C; R_f = 0.44 (20% EtOAc/hex); FTIR (thin film): ν_{max} 2919 (w), 2848 (w), 1690 (m), 1610 (s), 1580 (m), 1482 (m), 1457 (m), 1338 (m), 1223 (s), 1172 (m), 1136 (m), 1104 (m), 1092 (m), 1027 (w), 979 (w), 929 (w), 815 (m); ^{1}H NMR (500 MHz, $CDCl_3$) δ 7.57 (d, *J* = 8.6 Hz, 1H, H4), 7.39 (d, *J* = 8.6 Hz, 1H, H1'), 7.14 (ddd, *J* = 7.7, 2.7, 0.9 Hz, 1H, H6'), 6.84

(d, *J* = 2.2 Hz, 1H, H4'), 6.74 (dd, *J* = 8.7, 2.1 Hz, 1H, H2'), 6.35 (dd, *J* = 8.6, 2.1 Hz, 1H, H5), 6.08 (d, *J* = 2.0 Hz, 1H, H7), 5.76 – 5.63 (m, 2H, H2'', H2'''), 5.41 (ddd, *J* = 7.7, 5.4, 3.2 Hz, 1H, H7'), 5.13 (appt. dq, *J* = 17.1, 1.7 Hz, 1H, H3''a), 5.09 – 4.99 (m, 3H, H3''b, H3'''), 4.31 (ddt, *J* = 11.9, 6.1, 1.3 Hz, 1H, H1'''a), 4.20 (ddt, *J* = 11.9, 5.6, 1.4 Hz, 1H, H1'''b), 3.89 (ddt, *J* = 17.5, 5.3, 1.8 Hz, 1H, H1''a), 3.85 (s, 3H, H1'''''), 3.83 (s, 3H, H1''''), 3.80 (ddt, *J* = 17.5, 5.0, 1.9 Hz, 1H, H1''b), 2.87 (appt. dt, *J* = 17.5, 3.0 Hz, 1H, H8'a), 2.42 (ddd, *J* = 17.5, 5.4, 1.0 Hz, 1H, H8'b); ^{13}C NMR (126 MHz, $CDCl_3$) δ 198.1 (C3), 168.2 (C6), 161.3 (C7a), 157.3 (C3'), 136.1 (C10'), 133.9 (C2'''), 133.7 (C2''), 133.0 (C4a'), 126.5 (C4), 121.4 (C6'), 119.5 (C1'), 117.6 (C9a'), 117.5 (C3'''), 116.0 (C3''), 115.8 (C10a'), 112.7 (C3a), 110.2 (C2'), 106.7 (C5), 104.6 (C7'), 92.6 (C4'), 91.8 (C7), 74.6 (C1'''), 67.0 (C2/C9'), 55.7 (C1'''''), 55.5 (C1''''), 46.7 (C1''), 31.7 (C8'); LRMS (ESI$^+$) *m/z* 443 (100%) [M+H]$^+$; HRMS (ESI$^+$) Calculated for $C_{27}H_{27}N_2O_4^+$ [M+H]$^+$ 443.1971, found 443.1983.

cisoid-1,9a'-diallyl-3',6-dimethoxy-8'*H*-spiro[indoline-2,9'-pyrido[1,2-*a*]indole]-3,10'(9a'*H*)-dione **27**

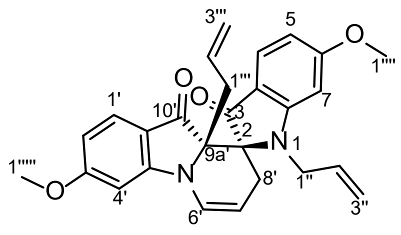

(±)-**27**

mp 84 – 86 °C; R$_f$ = 0.28 (20% EtOAc/hex); FTIR (thin film): ν_{max} 2974 (w), 2842 (w), 1688 (m), 1607 (s), 1582 (m), 1474 (m), 1458 (m), 1229 (s), 1095 (m), 1024 (w), 823 (w), 652 (w); ^{1}H NMR (500 MHz, $CDCl_3$) δ 7.61 (d, *J* = 8.6 Hz, 1H, H4), 7.38 (d, *J* = 8.6 Hz, 1H, H1'), 6.83 (dt, *J* = 7.7, 2.0 Hz, 1H, H6'), 6.39 – 6.35 (m, 2H, H5, H2'), 6.32 (d, *J* = 2.0 Hz, 1H, H4'), 6.02 (d, *J* = 2.1 Hz, 1H, H7), 5.35 – 5.25 (m, 3H, H7', H2'', H2'''), 5.01 (ddt, *J* = 16.9, 2.4, 1.3 Hz, 1H, H3'''a), 4.93 (appt. dq, *J* = 17.2, 1.7 Hz, 1H, H3''a), 4.87 (dt, *J* = 10.4, 1.6 Hz, 1H, H3''b), 4.83 (ddt, *J* = 10.0, 2.0, 0.9 Hz, 1H, H3'''b), 3.94 (ddt, *J* = 17.1, 4.8, 1.9 Hz, 1H, H1''a), 3.88 (s, 3H, H1'''''), 3.75 (s, 3H, H1''''), 3.70 (ddt, *J* = 17.2, 5.9, 1.7 Hz, 1H, H1''b), 3.11 (ddt, *J* = 14.1, 6.8, 1.1 Hz, 1H, H1'''a), 2.94 (appt. dt, *J* = 19.3, 2.3 Hz, 1H, H8'a), 2.57 (ddt, *J* = 14.1, 7.8, 1.1 Hz, 1H, H1'''b), 2.07 (ddd, *J* = 19.3, 4.7, 1.8 Hz, 1H, H8'b); ^{13}C NMR (126 MHz, $CDCl_3$) δ 197.2 (C3), 195.9 (C10'), 167.7 (C3'), 167.4 (C6), 162.2 (C7a), 157.4 (C4a'), 134.2 (C2''), 130.9 (C2'''), 126.2 (C1'), 125.6 (C4), 122.0 (C6'), 119.3 (C3'''), 116.0 (C3''), 115.6 (C3a), 115.4 (C10a'), 107.9 (C2'), 107.7 (C5), 105.3 (C7'), 93.8 (C7), 91.7 (C4'), 73.7 (C9a'), 68.8 (C2/C9'), 55.7 (C1'''''), 55.4 (C1''''), 47.3 (C1''), 35.0 (C1'''), 29.9 (C8'); LRMS (ESI$^+$) *m/z* 443 (100%) [M+H]$^+$; HRMS (ESI$^+$) Calculated for $C_{27}H_{27}N_2O_4^+$ [M+H]$^+$ 443.1971, found 443.1987.

2.3.5 The allylation of 5,5'-diphenylindigo 1d

Following General Procedure B, a suspension of 5,5'-diphenylindigo **1d** (209 mg, 0.501 mmol) in anhydrous DMF (20 mL) was sonicated for 60 min under a N_2 atmosphere. The suspension was transferred to a flask containing pre-dried Cs_2CO_3 (652 mg, 2.00 mmol) and activated 3 Å M.S. (1 g) and plunged into a pre-heated oil bath at 85 – 88 °C under N_2 with stirring. After 60 min, the N_2 flow was cut and allyl bromide (0.22 mL, 310 mg, 2.56 mmol) was added and stirred for 17 min. The reaction was quenched with ice (100 g), diluted to 200 mL with brine, extracted with EtOAc (4 x 50 mL), and the combined organic phases washed with H_2O (5 x 30 mL) and brine (2 x 25 mL), dried ($MgSO_4$) and concentrated *in vacuo*. The residue was adsorbed onto silica (6 g), and subjected to column chromatography (90 g silica, 10% → 30% EtOAc/hex) to generate two fractions (F_1 and F_2). Fraction F_1 contained 1-allyl-10'-(allyloxy)-2',5-diphenyl-8'*H*-spiro[indoline-2,9'-pyrido[1,2-*a*]indol]-3-one **20** (178 mg), isolated as a yellow solid. Purification of F_2 by PTLC (10% EtOAc/hex, eluted 5x) yielded additional **20** (22 mg; total 200 mg, 75%) and 1,9a'-diallyl-2',5-diphenyl-8'*H*-spiro[indoline-2,9'-pyrido[1,2-*a*]indole]-3,10'(9a'*H*)-dione **28** (9 mg, 3%), isolated as an orange solid.

<u>1-allyl-10'-(allyloxy)-2',5-diphenyl-8'*H*-spiro[indoline-2,9'-pyrido[1,2-*a*]indol]-3-one</u> **20**

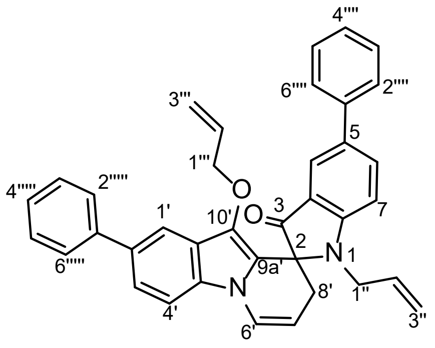

20

mp 140 – 141 °C; R$_f$ (20% EtOAc/hex) = 0.62; FTIR (thin film): ν_{max} 3060 (w), 2864 (w), 1701 (s), 1620 (s), 1513 (w), 1471 (s), 1456 (s), 1406 (m), 1348 (m), 1261 (m), 761 (s), 698 (m); ^{1}H NMR (400 MHz, $CDCl_3$) δ 7.91 (dd, *J* = 2.1, 0.6 Hz, 1H, H4), 7.77 (dd, *J* = 8.6, 2.0 Hz, 1H, H6), 7.72 (dd, *J* = 1.7, 0.7 Hz, 1H, H1'), 7.62 – 7.55 (m, 4H, H2'''' & H6'''', H2''''' & H6'''''), 7.49 (dd, *J* = 8.6, 1.7 Hz, 1H, H3'), 7.47 – 7.39 (m, 5H, H4', H3'''' & H5'''', H3''''' & H5'''''), 7.34 – 7.29 (m, 2H, H4'''', H4'''''), 7.24 (ddd, *J* = 7.8, 2.8, 1.0 Hz, 1H, H6'), 6.80 (dd, *J* = 8.6, 0.6 Hz, 1H, H7), 5.80 – 5.61 (m, 2H, H2'', H2'''), 5.49 (ddd, *J* = 7.8, 5.5, 3.2 Hz, 1H, H7'), 5.17 (appt. dq, *J* = 17.1, 1.6 Hz, 1H, H3''a), 5.08 – 4.95 (m, 3H, H3''b, H3'''), 4.36 (ddt, *J* = 12.0, 6.0, 1.3 Hz, 1H, H1'''a), 4.27 (ddt, *J* = 11.9, 5.6, 1.4 Hz, 1H, H1'''b), 3.97 (ddt, *J* = 17.4, 5.4, 1.7 Hz, 1H, H1''a), 3.87 (ddt, *J* = 17.4, 5.1, 1.8 Hz, 1H, H1''b), 2.95 (appt. dt, *J* = 17.5, 3.0 Hz, 1H, H8'a), 2.51 (ddd, *J* = 17.5, 5.5, 1.0 Hz, 1H, H8'b); ^{13}C NMR (101 MHz, $CDCl_3$) δ 200.4 (C3), 158.7 (C7a), 141.9 (C1'''''), 140.3 (C1''''), 136.9 (C6), 136.3 (C10'), 134.1 (C2'), 133.7 (C2''), 133.4 (C2'''), 131.6 (C4a'), 130.9 (C5), 128.9 (C3'''' & C5''''),

128.7 (C3''''' & C5'''''), 127.3 (C2'''' & C6''''), 126.69 (C4'''''), 126.68 (C4''''), 126.4 (C2''''' & C6'''''), 123.0 (C3'), 122.9 (C4), 121.8 (C10a'), 121.6 (C6'), 119.57 (C9a'), 119.55 (C3a), 117.6 (C3''), 117.2 (C1'), 116.3 (C3'''), 109.3 (C7), 109.3 (C4'), 104.9 (C7'), 74.7 (C1'''), 66.7 (C2/C9'), 46.9 (C1''), 31.6 (C8'); LRMS (ESI$^+$) *m/z* 535 (100%) [M+H]$^+$; HRMS (ESI$^+$) Calculated for $C_{37}H_{30}N_2NaO_2^+$ [M+Na]$^+$ 557.2205, found 557.2221.

cisoid-1,9a'-diallyl-2',5-diphenyl-8'*H*-spiro[indoline-2,9'-pyrido[1,2-*a*]indole]-3,10'(9a'*H*)-dione **28**

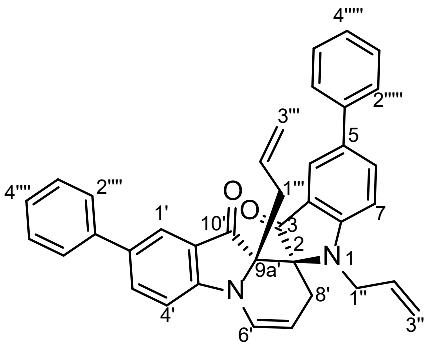

(±)-**28**

mp 107 – 110 °C; R$_f$ (20% EtOAc/hex) = 0.58; FTIR (thin film): ν_{max} 2923 (m), 2863 (w), 1699 (s), 1618 (s), 1480 (s), 1280 (m), 761 (m), 697 (m); ^{1}H NMR (400 MHz, $CDCl_3$) δ 7.94 (d, *J* = 2.0 Hz, 1H, H4), 7.79 (dd, *J* = 8.6, 2.0 Hz, 1H, H3'), 7.71 (d, *J* = 2.0 Hz, 1H, H1'), 7.65 (dd, *J* = 8.5, 2.0 Hz, 1H, H6), 7.54 (appt. td, *J* = 8.6, 1.3 Hz, 4H, H2'''' & H6'''', H2''''' & H6'''''), 7.40 (appt. td, *J* = 7.7, 2.6 Hz, 4H, H3'''' & H5'''', H3''''' & H5'''''), 7.31 – 7.28 (m, 2H, H4'''', H4'''''), 7.03 (d, *J* = 8.5 Hz, 1H, H4'), 6.93 (dt, *J* = 7.6, 2.0 Hz, 1H, H6'), 6.71 (d, *J* = 8.6 Hz, 1H, H7), 5.42 – 5.29 (m, 3H, H7', H2'', H2'''), 5.06 (appt. dq, *J* = 16.8, 1.4 Hz, 1H, H3'''a), 4.93 (appt. dq, *J* = 17.3, 1.6 Hz, 1H, H3''a), 4.89 – 4.82 (m, 2H, H3''b, H3'''b), 4.04 (ddt, *J* = 17.2, 4.7, 1.9 Hz, 1H, H1''a), 3.76 (ddt, *J* = 17.2, 5.8, 1.7 Hz, 1H, H1''b), 3.17 (dd, *J* = 14.0, 6.9 Hz, 1H, H1'''a), 2.99 (appt. dt, *J* = 19.3, 2.4 Hz, 1H, H8'a), 2.66 (dd, *J* = 14.0, 7.7 Hz, 1H, H1'''b), 2.15 (ddd, *J* = 19.3, 4.6, 1.7 Hz 1H, H8'b); ^{13}C NMR (101 MHz, $CDCl_3$) δ 199.9 (C3), 198.3 (C10'), 159.6 (C7a), 154.8 (C4a'), 140.2 (C1'''''), 139.7 (C1''''), 136.33 (C3'), 136.26 (C6), 133.8 (C2''), 132.6 (C2'), 132.0 (C5), 130.5 (C2'''), 128.9 (C3''''' & C5'''''), 128.8 (C3'''' & C5''''), 126.6 (C4'''', C4'''''), 126.3 (C2'''' & C6'''', C2''''' & C6'''''), 122.5 (C1', C6'), 122.3 (C3a), 122.2 (C10a'), 122.0 (C4), 119.8 (C3'''), 116.3 (C3''), 111.1 (C7), 108.6 (C4'), 105.0 (C7'), 73.8 (C9a'), 68.9 (C2/C9'), 47.5 (C1''), 34.9 (C1'''), 29.8 (C8'); LRMS (ESI$^+$) *m/z* 535 (100%) [M+H]$^+$; HRMS (ESI$^+$) Calculated for $C_{37}H_{30}N_2NaO_2^+$ [M+Na]$^+$ 557.2205, found 557.2221.

2.3.6 The allylation of 6,6'-diphenylindigo 1e

Following General Procedure B, a suspension of 6,6'-diphenylindigo **1e** (209 mg, 0.505 mmol) in anhydrous DMF (20 mL) was sonicated for 60 min under a N_2 atmosphere. The suspension was transferred to a flask containing pre-dried Cs_2CO_3 (652 mg, 2.00 mmol) and activated 3 Å

M.S. (1 g) and plunged into a pre-heated oil bath at 85 – 88 °C under N_2 with stirring. After 60 min, the N_2 flow was cut and allyl bromide (0.22 mL, 310 mg, 2.56 mmol) was added and stirred for 45 min. The reaction was quenched with ice (100 g), diluted to 200 mL with brine, extracted with EtOAc (3 x 50 mL) and the combined organic phases washed with H_2O (5 x 30 mL) and brine (2 x 25 mL), dried ($MgSO_4$) and concentrated *in vacuo*. The residue was adsorbed onto silica (6 g) and subjected to column chromatography (90 g silica, 10% → 60% EtOAc/hex) to yield two fractions (F_1 and F_2). Fraction F_1 was adsorbed onto silica (2.5 g) and subjected to column chromatography (30 g silica, 10% → 30% EtOAc/hex) to yield 1-allyl-10'-(allyloxy)-3',6-diphenyl-8'*H*-spiro[indoline-2,9'-pyrido[1,2-*a*]indol]-3-one **21** (133 mg) isolated as a yellow solid. Purification of F_2 by PTLC (10% EtOAc/hex, eluted 3x) yielded additional **21** (22 mg; total 155 mg, 58%) and 1,9a'-diallyl-3',6-diphenyl-8'*H*-spiro[indoline-2,9'-pyrido[1,2-*a*]indole]-3,10'(9a'*H*)-dione **29** (9.1 mg, 3%), isolated as an orange solid.

<u>1-allyl-10'-(allyloxy)-3',6-diphenyl-8'*H*-spiro[indoline-2,9'-pyrido[1,2-*a*]indol]-3-one</u> **21**

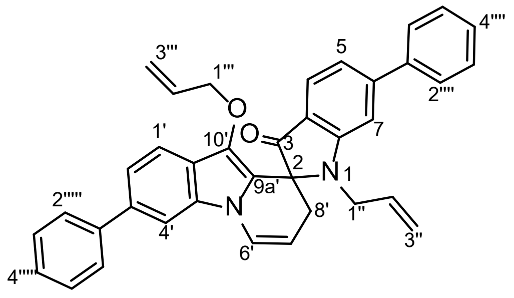

21

mp 68 – 72 °C; R_f = 0.54 (20% EtOAc/hex); FTIR (thin film): ν_{max} 3060 (w), 2865 (w), 1702 (s), 1620 (s), 1513 (w), 1471 (s), 1457 (s), 1406 (m), 1348 (m), 1261 (m), 761 (s), 698 (m); ^{1}H NMR (400 MHz, $CDCl_3$) δ 7.72 (d, *J* = 8.0 Hz, 1H, H4), 7.66 – 7.57 (m, 6H, H1', H4', H2'''' & H6'''', H2''''' & H6'''''), 7.48 – 7.34 (m, 7H, H2', H3'''' & H5'''', H4'''', H3''''' & H5''''', H4'''''), 7.29 (ddd, *J* = 7.8, 2.8, 1.0 Hz, 1H, H6'), 7.02 (dd, *J* = 8.0, 1.4 Hz, 1H, H5), 6.87 (d, *J* = 1.3 Hz, 1H, H7), 5.81 – 5.65 (m, 2H, H2'', H2'''), 5.49 (ddd, *J* = 7.8, 5.4, 3.3 Hz, 1H, H7'), 5.18 (appt. dq, *J* = 17.1, 1.7 Hz, 1H, H3''a), 5.11 – 4.97 (m, 3H, H3''b, H3'''), 4.37 (ddt, *J* = 11.9, 6.1, 1.3 Hz, 1H, H1'''a), 4.25 (ddt, *J* = 11.9, 5.7, 1.4 Hz, 1H, H1'''b), 4.00 (ddt, *J* = 17.5, 5.2, 1.8 Hz, 1H, H1''a), 3.88 (ddt, *J* = 17.5, 5.0, 1.8 Hz, 1H, H1''b), 2.94 (appt. dt, *J* = 17.5, 3.1 Hz, 1H, H8'a), 2.52 (ddd, *J* = 17.5, 5.5, 1.1 Hz, 1H, H8'b); ^{13}C NMR (101 MHz, $CDCl_3$) δ 199.9 (C3), 159.7 (C7a), 150.8 (C6), 141.8 (C1'''''), 140.9 (C1''''), 136.8 (C3'), 135.9 (C10'), 133.8 (C2''), 133.5 (C2'''), 132.7 (C4a'), 128.83 (C3'''' & C5''''), 128.78 (C3''''' & C5'''''), 128.4 (C4'''''), 127.40 (C2'''' & C6''''), 127.36 (C2''''' & C6'''''), 127.0 (C4''''), 125.3 (C4), 121.4 (C6'), 120.5 (C10a'), 120.3 (C2'), 119.5 (C9a'), 119.1 (C1'), 118.0 (C3a), 117.7 (C3'''), 117.5 (C5), 116.1 (C3''), 107.5 (C4'), 107.3 (C7), 104.9 (C7'), 74.7 (C1'''), 66.8 (C2/C9'), 46.8 (C1''),

31.7 (C8'); LRMS (ESI+) *m/z* 535 (100%) [M+H]+; HRMS (ESI+) Calculated for $C_{37}H_{31}N_2O_2^+$ [M+H]+ 535.2386, found 535.2379.

cisoid-1,9a'-diallyl-3',6-diphenyl-8'*H*-spiro[indoline-2,9'-pyrido[1,2-*a*]indole]-3,10'(9a'*H*)-dione **29**

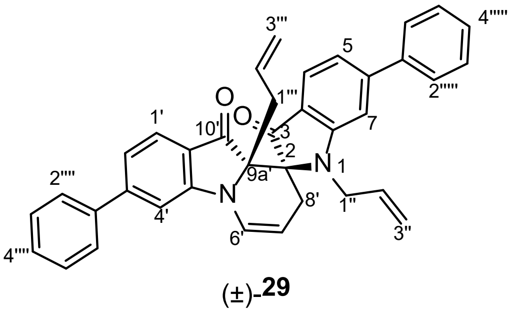

(±)-**29**

mp 110 – 112 °C; R_f = 0.50 (30% EtOAc/hex); FTIR (thin film): ν_{max} 3062 (w), 2922 (w), 2852 (w), 1696 (m), 1610 (s), 1572 (m), 1460 (m), 1428 (m), 1375 (m), 1324 (w), 1252 (w), 1179 (m), 989 (m), 759 (m), 697 (m); ^{1}H NMR (500 MHz, $CDCl_3$) δ 7.76 (d, *J* = 8.0 Hz, 1H, H4), 7.65 – 7.61 (m, 2H, H2'''' & H6''''), 7.53 – 7.34 (m, 9H, H1', H3'''' & H5'''', H4'''', H2''''' & H6''''', H3''''' & H5''''', H4'''''), 7.10 (d, *J* = 1.3 Hz, 1H, H4'), 7.07 – 7.01 (m, 2H, H5, H2'), 6.97 (dt, *J* = 7.7, 1.9 Hz, 1H, H6'), 6.78 (d, *J* = 1.3 Hz, 1H, H7), 5.43 – 5.29 (m, 3H, H7', H2'', H2'''), 5.06 (ddt, *J* = 16.9, 2.4, 1.3 Hz, 1H, H3'''a), 4.92 (appt. dq, *J* = 17.2, 1.6 Hz, 1H, H3''a), 4.87 (ddt, *J* = 10.1, 1.9, 0.9 Hz, 1H, H3'''b), 4.83 (appt. dq, *J* = 10.4, 1.6 Hz, 1H, H3''b), 4.07 (ddt, *J* = 17.2, 4.9, 1.9 Hz, 1H, H1''a), 3.78 (ddt, *J* = 17.3, 5.7, 1.7 Hz, 1H, H1''b), 3.17 (ddt, *J* = 14.2, 7.0, 1.2 Hz, 1H, H1'''a), 2.98 (appt. dt, *J* = 19.2, 2.3 Hz, 1H, H8'a), 2.65 (ddt, *J* = 14.0, 7.7, 1.1 Hz, 1H, H1'''b), 2.14 (ddd, *J* = 19.2, 4.6, 1.8 Hz, 1H, H8'b); ^{13}C NMR (126 MHz, $CDCl_3$) δ 199.4 (C3), 197.8 (C10'), 160.6 (C7a), 156.0 (C4a'), 150.4 (C3'), 150.0 (C6), 140.8 (C1'''''), 140.3 (C1''''), 133.9 (C2''), 130.7 (C2'''), 128.9 (C3'''' & C5''''), 128.74 (C3''''' & C5'''''), 128.69 (C4'''''), 128.3 (C4''''), 127.44 (C2''''' & C6'''''), 127.37 (C2'''' & C6''''), 124.8 (C1'), 124.3 (C4), 122.3 (C6'), 121.0 (C3a), 120.6 (C10a'), 119.6 (C3'''), 119.1 (C2'), 118.6 (C5), 116.1 (C3''), 109.0 (C7), 106.5 (C4'), 105.0 (C7'), 73.8 (C9a'), 68.9 (C2/C9'), 47.4 (C1''), 34.9 (C1'''), 29.9 (C8'); LRMS (ESI+) *m/z* 535 (100%) [M+H]+; HRMS (ESI+) Calculated for $C_{37}H_{31}N_2O_2^+$ [M+H]+ 535.2386, found 535.2379.

2.3.7 The allylation of 5,5'-dibromoindigo 1f

1-allyl-10'-(allyloxy)-2',5-dibromo-8'*H*-spiro[indoline-2,9'-pyrido[1,2-*a*]indol]-3-one **22**

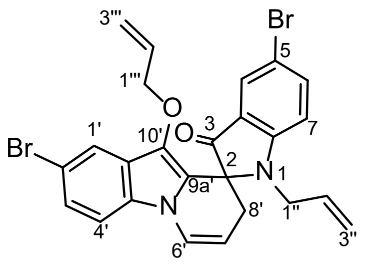

22

Following General Procedure B, a suspension of 5,5'-dibromoindigo **1f** (209.7 mg, 0.499 mmol) in anhydrous DMF (20 mL) was sonicated for 65 min under a N_2 atmosphere. The suspension was transferred to a flask containing pre-dried Cs_2CO_3 (652 mg, 2.00 mmol) and activated 3 Å M.S. (1 g) and plunged into a pre-heated oil bath at 85 – 88 °C under N_2 with stirring. After 1 h, the N_2 flow was cut and allyl bromide (0.22 mL, 310 mg, 2.56 mmol) was added and stirred for 5 min. The reaction was quenched with ice (100 g), diluted to 200 mL with brine, extracted with EtOAc (3 x 60 mL) and the combined organic phases washed with H_2O (5 x 25 mL) and brine (2 x 20 mL), dried ($MgSO_4$) and concentrated *in vacuo*. The residue was adsorbed onto silica (6 g) and subjected to column chromatography (30 g silica, 10% EtOAc/hex → 100% EtOAc) to yield 1-allyl-10'-(allyloxy)-2',5-dibromo-8'*H*-spiro[indoline-2,9'-pyrido[1,2-*a*]indol]-3-one **22** as an orange amorphous solid (166 mg, 62%). mp 109 – 112 °C; R_f (20% EtOAc/hex) = 0.48; FTIR (thin film): ν_{max} 1704 (s), 1654 (w), 1610 (s), 1476 (s), 1462 (s), 1278 (m), 1260 (s), 1017 (m), 795 (s), 750 (s); ^{1}H NMR (400 MHz, $CDCl_3$) δ 7.74 (d, *J* = 2.0 Hz, 1H, H4), 7.65 (d, *J* = 1.6 Hz, 1H, H1'), 7.52 (dd, *J* = 8.7, 2.1 Hz, 1H, H6), 7.32 (dd, *J* = 8.8, 1.8 Hz, 1H, H3'), 7.26 (d, *J* = 8.7 Hz, 1H, H4'), 7.15 (ddd, *J* = 7.8, 2.9, 1.0 Hz, 1H, H6'), 6.61 (d, *J* = 8.8 Hz, 1H, H7), 5.74 – 5.56 (m, 2H, H2'', H2'''), 5.48 (ddd, *J* = 7.8, 5.5, 3.2 Hz, 1H, H7'), 5.11 – 4.98 (m, 4H, H3'', H3'''), 4.30 (ddt, *J* = 12.0, 5.9, 1.3 Hz, 1H, H1'''a), 4.19 (ddt, *J* = 11.9, 5.6, 1.4 Hz, 1H, H1'''b), 3.88 (ddt, *J* = 17.4, 5.3, 1.7 Hz, 1H, H1''a), 3.79 (ddt, *J* = 17.5, 5.0, 1.8 Hz, 1H, H1''b), 2.86 (appt. dt, *J* = 17.6, 3.1 Hz, 1H, H8'a), 2.45 (ddd, *J* = 17.6, 5.5, 1.0 Hz, 1H, H8'b); ^{13}C NMR (101 MHz, $CDCl_3$) δ 199.0 (C3), 157.8 (C7a), 140.1 (C6), 135.3 (C10'), 133.03 (C2'''), 133.01 (C2''), 130.7 (C4a'), 127.4 (C4), 126.2 (C3'), 122.6 (C10a'), 121.4 (C1'), 121.3 (C6'), 120.5 (C3a), 119.6 (C9a'), 118.0 (C3'''), 116.4 (C3''), 113.8 (C2'), 110.7 (C7), 110.5 (C4'), 109.6 (C5), 105.2 (C7'), 74.6 (C1'''), 66.4 (C2/C9'), 46.7 (C1''), 31.4 (C8'); LRMS (ESI$^+$) *m/z* 561 (49%) [^{79}Br, ^{79}Br, M+Na]$^+$, 563 (100%) [^{79}Br, ^{81}Br, M+Na]$^+$, 565 (51%) [^{81}Br, ^{81}Br, M+Na]$^+$; HRMS (ESI$^+$) Calculated for $C_{25}H_{20}{}^{79}Br_2N_2NaO_2^+$ [M+Na]$^+$ 560.9789, found 560.9795.

2.3.8 The allylation of 6,6'-dibromoindigo 1g

1-allyl-10'-(allyloxy)-3',6-dibromo-8'*H*-spiro[indoline-2,9'-pyrido[1,2-*a*]indol]-3-one **23**

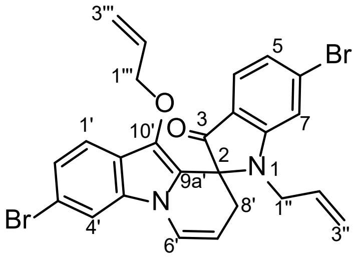

23

Following General Procedure B, a suspension of 6,6'-dibromoindigo **1g** (210 mg, 0.500 mmol) in anhydrous DMF (20 mL) was sonicated for 60 min under a N_2 atmosphere. The suspension was transferred to a flask containing pre-dried Cs_2CO_3 (654 mg, 2.01 mmol) and activated 3 Å M.S. (1 g) and plunged into a pre-heated oil bath at 85 – 88 °C under N_2 with stirring. After 60 min, the N_2 flow was cut and allyl bromide (0.22 mL, 310 mg, 2.56 mmol) was added and stirred for 15 min. The reaction was quenched with ice (100 g), diluted to 200 mL with brine, extracted with EtOAc (3 x 50 mL) and the combined organic phases washed with H_2O (5 x 25 mL) and brine (2 x 20 mL), dried ($MgSO_4$) and concentrated *in vacuo*. The residue was adsorbed onto silica (6 g), and subjected to column chromatography (90 g silica, 10% → 30% EtOAc/hex) to yield two fractions (F_1 and F_2). Fraction F_1 contained pure 1-allyl-10'-(allyloxy)-3',6-dibromo-8'*H*-spiro[indoline-2,9'-pyrido[1,2-*a*]indol]-3-one **200**, isolated as a yellow solid (166 mg) and purification of F_2 by PTLC (15% EtOAc/H, eluted 2x) yielded additional **23** (20 mg; total 186 mg, 69%). mp 138 – 141 °C; R_f (20% EtOAc/hex) = 0.60; FTIR (thin film): ν_{max} 3081 (w), 2924 (w), 1706 (s), 1605 (s), 1555 (w), 1466 (s), 1312 (m), 1244 (w), 1056 (w), 925 (w); ^{1}H NMR (500 MHz, $CDCl_3$) δ 7.55 (d, *J* = 1.6 Hz, 1H, H4'), 7.48 (d, *J* = 8.2 Hz, 1H, H4), 7.39 (d, *J* = 8.5 Hz, 1H, H1'), 7.20 (dd, *J* = 8.6, 1.4 Hz, 1H, H2'), 7.13 (dd, *J* = 7.9, 2.7 Hz, 1H, H6'), 6.90 (dd, *J* = 8.1, 1.5 Hz, 1H, H5), 6.87 (d, *J* = 1.4 Hz, 1H, H7), 5.74 – 5.56 (m, 2H, H2'', H2'''), 5.48 (ddd, *J* = 8.2, 5.4, 3.1 Hz, 1H, H7'), 5.14 – 5.00 (m, 4H, H3'', H3'''), 4.31 (ddt, *J* = 12.0, 6.0, 1.4 Hz, 1H, H1'''a), 4.19 (ddt, *J* = 12.0, 5.7, 1.4 Hz, 1H, H1'''b), 3.88 (ddt, *J* = 17.5, 5.4, 1.8 Hz, 1H, H1''a), 3.78 (ddt, *J* = 17.5, 5.1, 1.8 Hz, 1H, H1''b), 2.87 (appt. dt, *J* = 17.6, 3.1 Hz, 1H, H8'a), 2.44 (dd, *J* = 17.6, 5.5 Hz, 1H, H8'b); ^{13}C NMR (126 MHz, $CDCl_3$) δ 199.2 (C3), 159.4 (C7a), 136.0, (C10'), 133.3 (C6), 133.1 (C2'''), 132.8 (C2''), 132.7 (C4a'), 126.0 (C4), 123.8 (C2'), 121.2 (C6'), 121.1 (C5), 120.1 (C1'), 120.0 (C10a'), 119.0 (C9a'), 118.0 (C3'''), 117.9 (C3a), 117.1 (C3'), 116.6 (C3''), 112.2 (C4'), 112.0 (C7), 105.4 (C7'), 74.7 (C1'''), 66.4 (C2/C9'), 46.7 (C1''), 31.5 (C8'); LRMS (ESI$^+$) *m/z* 539 (68%) [^{79}Br, ^{79}Br, M+H]$^+$, 541 (100%) [^{79}Br, ^{81}Br, M+H]$^+$, 543 (49%) [^{81}Br, ^{81}Br, M+H]$^+$; HRMS (ESI$^+$) Calculated for $C_{25}H_{20}{}^{79}Br_2N_2NaO_2^+$ [M+Na]$^+$ 560.9789, found 560.9796.

2.3.9 The allylation of 5,5'-dinitroindigo 1h

<u>1-allyl-10'-(allyloxy)-2',5-dinitro-8'*H*-spiro[indoline-2,9'-pyrido[1,2-*a*]indol]-3-one</u> **24**

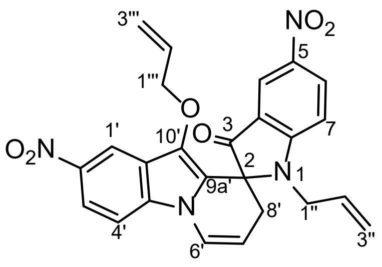

24

Following General Procedure B, a suspension of 5,5'-dinitroindigo **1h** (53.3 mg, 0.151 mmol) in anhydrous DMF (6.0 mL) was sonicated for 60 min under a N_2 atmosphere. The suspension was transferred to a flask containing pre-dried Cs_2CO_3 (196 mg, 0.601 mmol) and activated 3 Å M.S. (0.3 g) and plunged into a pre-heated oil bath at 85 – 88 °C under N_2 with stirring. After 60 min, the N_2 flow was cut and allyl bromide (0.07 mL, 110 mg, 0.91 mmol) was added and stirred. After 70 min, the reaction was cooled to rt, diluted to 50 mL with brine, extracted with EtOAc (4 x 20 mL) and the combined organic phases washed with H_2O (5 x 10 mL) and brine (2 x 10 mL), dried ($MgSO_4$) and concentrated *in vacuo*. The residue was adsorbed onto silica (1.2 g) and subjected to column chromatography (20 g silica, 10% EtOAc/hex → 100% EtOAc) to yield 1-allyl-10'-(allyloxy)-2',5-dinitro-8'*H*-spiro[indoline-2,9'-pyrido[1,2-*a*]indol]-3-one **24** (36.2 mg, 51%) as a dark orange amorphous solid. mp 143 – 146 °C; R_f (30% EtOAc/hex) = 0.25; FTIR (thin film): ν_{max} 2923 (m), 2853 (w), 1719 (m), 1613 (s), 1514 (m), 1489 (m), 1453 (m), 1317 (s), 1262 (m), 1071 (m); ^{1}H NMR (400 MHz, $CDCl_3$) δ 8.55 (d, *J* = 2.3 Hz, 1H, H4), 8.52 (d, *J* = 2.1 Hz, 1H, H1'), 8.39 (dd, *J* = 9.1, 2.4 Hz, 1H, H6), 8.17 (dd, *J* = 9.2, 2.1 Hz, 1H, H3'), 7.47 (d, *J* = 9.2 Hz, 1H, H4'), 7.28 (ddd, *J* = 7.9, 2.9, 0.9 Hz, 1H, H6'), 6.75 (d, *J* = 9.2 Hz, 1H, H7), 5.77 – 5.56 (m, 3H, H7', H2'', H2'''), 5.15 – 5.02 (m, 4H, H3'', H3'''), 4.49 (ddt, *J* = 11.9, 5.8, 1.3 Hz, 1H, H1'''a), 4.33 (ddt, *J* = 11.9, 5.7, 1.3 Hz, 1H, H1'''b), 4.06 (ddt, *J* = 17.5, 5.5, 1.7 Hz, 1H, H1''a), 3.96 (ddt, *J* = 17.4, 5.0, 1.8 Hz, 1H, H1''b), 2.96 (appt. dt, *J* = 17.9, 3.1 Hz, 1H, H8'a), 2.57 (ddd, *J* = 17.9, 5.5, 1.1 Hz, 1H, H8'b); ^{13}C NMR (101 MHz, $CDCl_3$) δ 198.0 (C3), 160.9 (C7a), 142.2 (C2'), 139.4 (C5), 137.9 (C10'), 134.2 (C4a'), 133.1 (C6), 132.5 (C2'''), 131.5 (C2''), 122.3 (C4), 121.2 (C6'), 120.3 (C9a'), 120.1 (C10a'), 119.1 (C3'), 118.7 (C3'''), 118.5 (C3a), 117.5 (C3''), 116.5 (C1'), 109.4 (C4'), 108.5 (C7), 107.1 (C7'), 74.8 (C1'''), 67.2 (C2/C9'), 46.7 (C1''), 31.0 (C8'); LRMS (ESI$^-$) *m/z* 471 (100%) [M-H]$^-$; HRMS (ESI$^-$) Calculated for $C_{25}H_{19}N_4O_6^-$ [M-H]$^-$ 471.1305, found 471.1314.

2.3.10 The allylation of 6,6'-dinitroindigo 1i

Following General Procedure B, a suspension of 6,6'-dinitroindigo **1i** (176 mg, 0.500 mmol) in anhydrous DMF (20 mL) was sonicated for 60 min under a N_2 atmosphere. The suspension

was transferred to a flask containing pre-dried Cs_2CO_3 (652 mg, 2.00 mmol) and activated 3 Å M.S. (1 g) and plunged into a pre-heated oil bath at 85 – 88 °C under N_2 with stirring. After 60 min, the N_2 flow was cut and allyl bromide (0.22 mL, 310 mg, 2.56 mmol) was added and stirred for 8 min. The reaction was quenched with ice (100 g), diluted to 200 mL with brine, extracted with EtOAc (3 x 30 mL) and the combined organic phases washed with H_2O (5 x 25 mL) and brine (2 x 20 mL), dried ($MgSO_4$) and concentrated *in vacuo*. The residue was adsorbed onto silica (10 g) and subjected to column chromatography (100 g silica, 10% → 70% EtOAc/hex) to yield three major fractions (F_1, F_2 and F_3).

Recrystallisation of F_2 (10% EtOAc/hex) yielded 1-allyl-10'-(allyloxy)-3',6-dinitro-8'*H*-spiro[indoline-2,9'-pyrido[1,2-*a*]indol]-3-one **25** (62 mg). The mother liquor and F_3 were combined, adsorbed onto silica (4 g) and subjected to column chromatography (50 g silica, 20% → 50% EtOAc/hex) to yield additional **25** (23 mg).

1-allyl-10'-(allyloxy)-3',6-dinitro-8'*H*-spiro[indoline-2,9'-pyrido[1,2-*a*]indol]-3-one **25**

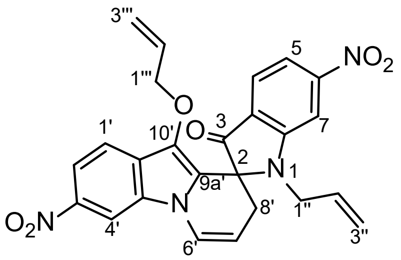

25

Isolated as either red crystals or an amorphous orange solid (85 mg, 36%). mp 169 – 173 °C; R_f = 0.33 (20% EtOAc/hex); FTIR (thin film): ν_{max} 3098 (w), 2852 (w), 1718 (m), 1625 (m), 1531 (s), 1470 (m), 1337 (s), 1248 (m), 734 (m); ^1H NMR (400 MHz, $CDCl_3$) δ 8.40 (d, *J* = 1.9 Hz, 1H, H4'), 8.00 (dd, *J* = 8.9, 2.0 Hz, 1H, H2'), 7.77 (d, *J* = 8.4 Hz, 1H, H4), 7.62 (d, *J* = 8.8 Hz, 1H, H1'), 7.61 (dd, *J* = 8.4, 1.9 Hz, 1H, H5), 7.51 (d, *J* = 1.8 Hz, 1H, H7), 7.32 (ddd, *J* = 7.9, 3.0, 0.9 Hz, 1H, H6'), 5.74 – 5.55 (m, 3H, H7', H2'', H2'''), 5.15 – 5.01 (m, 4H, H3'', H3'''), 4.41 (ddt, *J* = 12.0, 5.8, 1.3 Hz, 1H, H1'''a), 4.27 (ddt, *J* = 12.0, 5.7, 1.4 Hz, 1H, H1'''b), 4.01 (ddt, *J* = 17.4, 5.5, 1.7 Hz, 1H, H1''a), 3.91 (ddt, *J* = 17.5, 5.0, 1.8 Hz, 1H, H1''b), 2.98 (appt. dt, *J* = 17.9, 3.1 Hz, 1H, H8'a), 2.56 (ddd, *J* = 17.8, 5.7, 1.0 Hz, 1H, H8'b); ^{13}C NMR (101 MHz, $CDCl_3$) δ 199.0 (C3), 158.5 (C7a), 154.5 (C6), 144.2 (C3'), 136.2 (C10'), 132.6 (C2'''), 131.8 (C2''), 130.6 (C4a'), 126.0 (C4), 124.7 (C10a'), 123.9 (C9a'), 122.9 (C3a), 121.0 (C6'), 119.2 (C1'), 118.4 (C3'''), 117.3 (C3''), 115.8 (C2'), 112.5 (C5), 106.7 (C7'), 106.2 (C4'), 103.9 (C7), 74.7 (C1'''), 66.6 (C2/C9'), 46.8 (C1''), 31.4 (C8'); LRMS (ESI$^+$) *m/z* 473 (100%) $[M+H]^+$; HRMS (ESI$^+$) Calculated for $C_{25}H_{21}N_4O_6^+$ $[M+H]^+$ 473.1461, found 473.1471.

Recrystallisation of F_1 (10% CH_2Cl_2/hex) yielded 12-allyl-13-(allyloxy)-3,10-dinitro-6-vinyl-6*H*,12*H*-[1,3]oxazino[3,4-*a*:5,6-*b*']diindole **30**.

12-allyl-13-(allyloxy)-3,10-dinitro-6-vinyl-6*H*,12*H*-[1,3]oxazino[3,4-*a*:5,6-*b*']diindole **30**

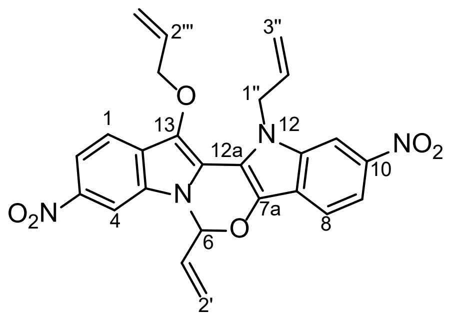

30

Isolated as brown-red radial crystals (31 mg, 13%). mp 160 – 163 °C; R_f = 0.56 (20% EtOAc/hex); FTIR (thin film): ν_{max} 3085 (w), 2850 (w), 1721 (m), 1534 (m), 1519 (m), 1331 (s), 1194 (w), 932 (w), 804 (w), 734 (w); ^{1}H NMR (400 MHz, $CDCl_3$) δ 8.31 (d, *J* = 1.9 Hz, 1H, H11), 8.25 (d, *J* = 1.9 Hz, 1H, H4), 8.04 (appt. dd, *J* = 8.8, 1.9 Hz, 2H, H2, H9), 7.72 (d, *J* = 8.8 Hz, 1H, H1), 7.70 (d, *J* = 8.9 Hz, 1H, H8), 6.81 (dt, *J* = 4.5, 1.4 Hz, 1H, H6), 6.18 – 5.90 (m, 3H, H1', H2'', H2'''), 5.49 – 5.41 (m, 2H, H1''a, H3'''a), 5.35 (appt. dq, *J* = 10.4, 1.2 Hz, 1H, H3'''b), 5.30 (dd, *J* = 10.4, 1.3 Hz, 1H, H2'a), 5.25 (ddt, *J* = 17.3, 4.2, 1.9 Hz, 1H, H1''b), 5.12 (appt. dq, *J* = 10.4, 1.5 Hz, 1H, H3''a), 4.94 (dd, *J* = 17.1, 1.4 Hz, 1H, H2'b), 4.83 (appt. dq, *J* = 17.1, 1.4 Hz, 1H, H3''b), 4.78 (ddt, *J* = 12.4, 5.6, 1.4 Hz, 1H, H1'''a), 4.72 (ddt, *J* = 12.4, 5.8, 1.4 Hz, 1H, H1'''b); ^{13}C NMR (126 MHz, $CDCl_3$) δ 144.3 (C10), 144.2 (C3), 134.8 (C11a), 133.5 (C12a), 132.8 (C2''), 132.7 (C2'''), 132.1 (C4a), 131.7 (C13), 131.6 (C1'), 125.8 (C13a), 122.0 (C7a), 121.8 (C7b), 121.0 (C2'), 120.3 (C12b), 119.4 (C3'''), 118.7 (C1), 117.8 (C8), 116.7 (C3''), 115.8 (C2), 115.7 (C9), 107.6 (C11), 106.0 (C4), 84.0 (C6), 76.1 (C1'''), 48.4 (C1''); LRMS (ESI$^+$) *m/z* 473 (100%) $[M+H]^+$; HRMS (ESI$^+$) Calculated for $C_{25}H_{21}N_4O_6^+$ $[M+H]^+$ 473.1461, found 473.1474.

The mother liquor (F_1 recrystallisation) was concentrated *in vacuo*, subjected to two PTLC plates (40% Et_2O/hex), and the crude residue rinsed with hex (30 x 3 mL). Combination and evaporation of the hexane washes followed by recrystallisation (CH_2Cl_2/hex) yielded 12-allyl-13-(allyloxy)-3,10-dinitro-6-vinyl-6*H*,12*H*-[1,3]oxazino[3,4-*a*:5,6-*b*']biindole **31**.

1,1'-diallyl-3,3'-bis(allyloxy)-6,6'-dinitro-1*H*,1'*H*-2,2'-biindole **31**

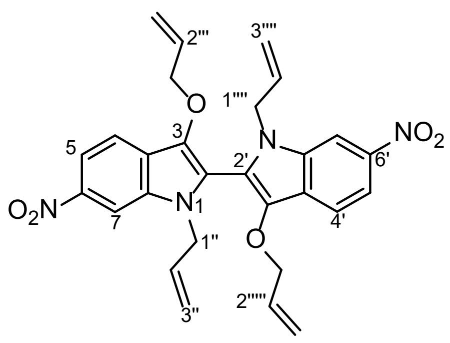

31

Isolated as bright orange needles (2.8 mg, 1%). mp 108 – 110 °C; R_f = 0.62 (30% EtOAc/hex); FTIR (thin film): ν_{max} 3084 (w), 2922 (w), 2856 (w), 1515 (m), 1457 (w), 1322 (s), 1308 (m), 1202 (w), 929 (w), 734 (w); ^{1}H NMR (500 MHz, $CDCl_3$) δ 8.37 (dd, *J* = 2.0, 0.6 Hz, 2H, H7 & H7'), 8.05 (dd, *J* = 8.8, 2.0 Hz, 2H, H5 & H5'), 7.79 (dd, *J* = 8.8, 0.5 Hz, 2H, H4 & H4'), 5.89 (ddt, *J* = 17.2, 10.4, 5.6 Hz, 2H, H2''' & H2'''''), 5.79 (dddd, *J* = 17.1, 10.5, 5.8, 5.0 Hz, 2H, H2'' & H2''''), 5.22 (appt. dq, *J* = 17.2,

1.5 Hz, 2H, H3''a & H3''''a), 5.14 (appt. dq, *J* = 10.4, 1.3 Hz, 2H, H3''b & H3''''b), 5.11 (appt. dq, *J* = 10.1, 1.4 Hz, 2H, H3'''a & H3'''''a), 4.92 (dtd, *J* = 17.1, 1.7, 0.9 Hz, 2H, H3'''b & H3'''''b), 4.78 (ddt, *J* = 16.8, 5.8, 1.6 Hz, 2H, H1''a & H1''''a), 4.73 (ddt, *J* = 16.8, 5.0, 1.7 Hz, 2H, H1''b & H1''''b), 4.53 (ddt, *J* = 12.5, 5.6, 1.4 Hz, 2H, H1'''a & H1'''''a), 4.47 (ddt, *J* = 12.6, 5.7, 1.4 Hz, 2H, H1'''b & H1'''''b); ^{13}C NMR (126 MHz, $CDCl_3$) δ 144.1 (C6 & C6'), 138.2 (C3 & C3'), 133.5 (C7a & C7a'), 133.3 (C2''' & C2'''''), 132.7 (C2'' & C2''''), 124.6 (C3a & C3a'), 122.0 (C2 & C2'), 118.8 (C4 & C4'), 118.3 (C3''' & C3'''''), 117.8 (C3'' & C3''''), 114.9 (C5 & C5'), 107.8 (C7 & C7'), 74.8 (C1''' & C1'''''), 47.4 (C1'' & C1''''); LRMS (ESI$^+$) *m/z* 537 (100%) [M+Na]$^+$; HRMS (ESI$^+$) Calculated for $C_{28}H_{26}N_4NaO_6^+$ [M+Na]$^+$ 537.1750, found 537.1735.

S2.4 Mechanistic Experiments

2.4.1 The attempted conversion of diallylbiindolone 16 to spiroindolinepyridoindolone 17

To a solution of diallylbiindolone **16** (2.5 mg, 0.006 mmol) in anhydrous DMF (0.2 mL) was added pre-dried Cs_2CO_3 (2.6 mg, 0.008 mmol), allyl bromide (1 µL, 1.4 mg, 0.0116 mmol) and stirred at 80 °C under a N_2 atmosphere. After 3 h, TLC analysis indicated minimal SM consumption had occurred and additional Cs_2CO_3 (2.5 mg, 0.0077 mmol) and allyl bromide **37** (10 µL, 7 mg, 0.058 mmol) was added and the reaction stirred for a further 36 h, then cooled to rt and concentrated *in vacuo*. Analysis by ^{1}H NMR indicated that complete conversion of SM to baseline decomposition had occurred with no evidence of spiroindolinepyridoindolone **17** formation.

2.4.2 The Claisen rearrangements of spiroindolinepyridoindolones

2.4.2.1 The Claisen rearrangement of dimethoxyspiroindolinepyridoindolone 17

A solution of 1-allyl-10'-(allyloxy)-2',5-dimethoxy-8'*H*-spiro[indoline-2,9'-pyrido[1,2-*a*]indol]-3-one **17** (44 mg, 0.100 mmol) in DMF (4.0 mL) was heated at 90 °C under a N_2 atmosphere for 5 d. The reaction was quenched with brine (20 mL), extracted with EtOAc (2 x 15 mL), and the combined organic phases washed with H_2O (5 x 5 mL) and brine (2 x 5 mL), dried ($MgSO_4$) and concentrated *in vacuo*. The crude residue was adsorbed onto silica (600 mg) and subjected to column chromatography (20 g silica, 10% → 30% EtOAc/hex), then PTLC (20% EtOAc/hex) to furnish *cisoid*- and *transoid*-1,9a'-diallyl-2',5-dimethoxy-8'*H*-spiro[indoline-

2,9'-pyrido[1,2-*a*]indole]-3,10'(9a'*H*)-dione **33a** as a yellow solid (28 mg, 63%) and **33b** as a yellow solid (3.6 mg, 8%).

<u>*cisoid*-1,9a'-diallyl-2',5-dimethoxy-8'*H*-spiro[indoline-2,9'-pyrido[1,2-*a*]indole]-3,10'(9a'*H*)-dione</u> **33a**

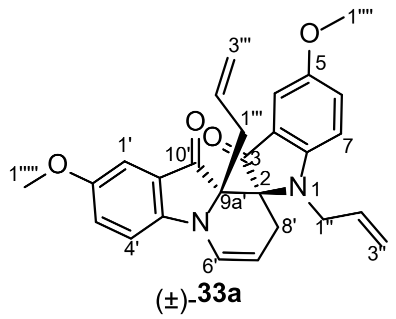

(±)-**33a**

mp 113 – 117 °C; R_f (20% EtOAc/hex) = 0.25; FTIR (thin film): ν_{max} 2923 (w), 2852 (w), 1688 (s), 1637 (m), 1689 (s), 1437 (m), 1330 (m), 1289 (m), 1222 (m), 1118 (m), 1030 (m), 918 (m), 816 (m); ^{1}H NMR (400 MHz, $CDCl_3$) δ 7.15 (d, *J* = 2.8 Hz, 1H, H4), 7.14 (dd, *J* = 8.9, 2.7 Hz, 1H, H3'), 7.04 (dd, *J* = 8.9, 2.7 Hz, 1H, H6), 6.93 – 6.85 (m, 2H, H1', H4'), 6.85 (dt, *J* = 7.6, 1.9 Hz, 1H, H6'), 6.61 (d, *J* = 8.9 Hz, 1H, H7), 5.39 – 5.14 (m, 3H, H7', H2'', H2'''), 5.02 (ddt, *J* = 16.9, 2.4, 1.3 Hz, 1H, H3'''a), 4.91 – 4.78 (m, 3H, H3'', H3'''b), 3.99 (ddt, *J* = 17.2, 4.7, 2.0 Hz, 1H, H1''a), 3.79 (s, 3H, H1''''), 3.73 (s, 3H, H1'''''), 3.63 (ddt, *J* = 17.1, 5.8, 1.6 Hz, 1H, H1''b), 3.11 (ddt, *J* = 14.0, 7.0, 1.1 Hz, 1H, H1'''a), 2.91 (appt. dt, *J* = 19.2, 2.3 Hz, 1H, H8'a), 2.59 (ddt, *J* = 13.9, 7.5, 1.1 Hz, 1H, H1'''b), 2.06 (ddd, *J* = 19.3, 4.7, 1.8 Hz, 1H, H8'b); ^{13}C NMR (101 MHz, $CDCl_3$) δ 200.1 (C3), 198.4 (C10'), 156.4 (C7a), 153.4 (C2'), 153.1 (C5), 151.1 (C4a'), 134.5 (C2''), 130.8 (C2'''), 127.5 (C6), 127.1 (C3'), 122.6 (C6'), 122.1 (C3a), 121.8 (C10a'), 119.3 (C3'''), 115.9 (C3''), 112.2 (C4), 109.4 (C4'), 105.0 (C1'), 104.0 (C7), 103.5 (C7'), 73.8 (C9a'), 69.2 (C2/C9'), 55.7 (C1'''', C1'''''), 47.8 (C1''), 34.6 (C1'''), 29.5 (C8'); LRMS (ESI$^+$) *m/z* 443 (100%) $[M+H]^+$; HRMS (ESI$^+$) Calculated for $C_{27}H_{27}N_2O_4^+$ $[M+H]^+$ 443.1971, found 443.1983.

<u>*transoid*-1,9a'-diallyl-2',5-dimethoxy-8'*H*-spiro[indoline-2,9'-pyrido[1,2-*a*]indole]-3,10'(9a'*H*)-dione</u> **33b**

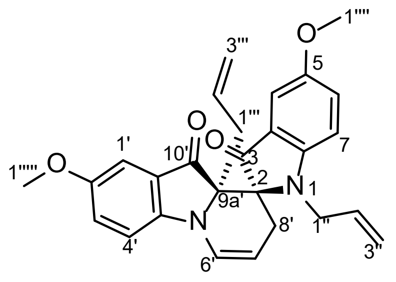

(±)-**33b**

mp 96 – 100 °C; R_f (20% EtOAc/hex) = 0.15; FTIR (thin film): ν_{max} 2917 (w), 1690 (m), 1653 (m), 1494 (s), 1450 (m), 1439 (m), 1334 (m), 1271 (m), 1227 (m), 1031 (m), 818 (m); ^{1}H NMR (500 MHz, $CDCl_3$) δ 7.16 (dd, *J* = 8.9, 2.7 Hz, 1H, H3'), 7.10 (dd, *J* = 9.0, 2.7 Hz, 1H, H6), 7.00 (dd, *J* = 8.9, 0.6 Hz, 1H, H4'), 6.90 (dt, *J* = 7.7, 2.0 Hz, 1H, H6'), 6.86 – 6.80 (m, 2H, H1', H7), 6.78 (d, *J* = 2.7 Hz, 1H, H4), 5.95 (ddt, *J* = 17.2, 10.5, 4.4 Hz, 1H, H2''), 5.65 (appt. dq, *J* = 17.1, 1.9 Hz, 1H, H3''a), 5.41 – 5.28 (m, 2H, H3''b, H2'''), 5.06 – 5.00 (m, 2H, H7', H3'''a), 4.88 (ddt, *J* = 10.0, 1.9, 0.9 Hz, 1H, H3'''b), 4.39 (ddt, *J* = 17.9, 4.4, 2.1 Hz, 1H, H1''a), 4.04 (ddt, *J* = 17.9, 4.2,

1.9 Hz, 1H, H1''b), 3.70 (s, 3H, H1'''''), 3.67 (s, 3H, H1''''), 2.81 – 2.75 (m, 2H, H8'a, H1'''a), 2.66 (ddt, *J* = 12.5, 7.3, 1.1 Hz, 1H, H1'''b), 2.05 (ddd, *J* = 18.1, 5.1, 1.8 Hz, 1H, H8'b); ^{13}C NMR (126 MHz, $CDCl_3$) δ 197.7 (C3), 197.4 (C10'), 156.6 (C7a), 153.3 (C2'), 152.74 (C5), 152.65 (C4a'), 133.8 (C2''), 130.6 (C2'''), 128.0 (C3'), 127.6 (C5), 123.6 (C6'), 122.0 (C10a'), 119.6 (C3a), 119.5 (C3'''), 117.9 (C3''), 112.2 (C7), 110.0 (C4'), 104.5 (C4), 104.2 (C1'), 99.2 (C7'), 69.5 (C9a'), 69.1 (C2/C9'), 55.7 (C1''''), 55.6 (C1'''''), 47.5 (C1''), 37.4 (C1'''), 29.9 (C8'); LRMS (ESI$^+$) *m/z* 443 (100%) $[M+H]^+$; HRMS (ESI$^+$) Calculated for $C_{27}H_{26}N_2NaO_4^+$ $[M+Na]^+$ 465.1790, found 465.1784.

2.4.2.2 The Claisen rearrangement of dimethoxyspiroindolinepyridoindolone 17 with TEMPO

A solution of 1-allyl-10'-(allyloxy)-2',5-dimethoxy-8'*H*-spiro[indoline-2,9'-pyrido[1,2-*a*]indol]-3-one **17** (44 mg, 0.100 mmol) in DMF (4.0 mL) was heated at 90 °C under N_2 with TEMPO (34 mg, 0.21 mmol) for 5 d. The reaction was quenched with brine (20 mL), extracted with EtOAc (2 x 15 mL) and the combined organic phases washed with H_2O (5 x 5 mL) and brine (2 x 5 mL), dried ($MgSO_4$) and concentrated *in vacuo*. The crude residue was adsorbed onto silica (600 mg) and subjected to column chromatography (20 g silica, 10% → 30% EtOAc/hex), then PTLC (20% EtOAc/hex) to furnish *cisoid*- and *transoid*-1,9a'-diallyl-2',5-dimethoxy-8'*H*-spiro[indoline-2,9'-pyrido[1,2-*a*]indole]-3,10'(9a'*H*)-dione **33a** as a yellow solid (36 mg, 83%) and **33b** as a yellow solid (1.3 mg, 3%).

***cisoid*-1,9a'-diallyl-2',5-dimethoxy-8'*H*-spiro[indoline-2,9'-pyrido[1,2-*a*]indole]-3,10'(9a'*H*)-dione 33a**

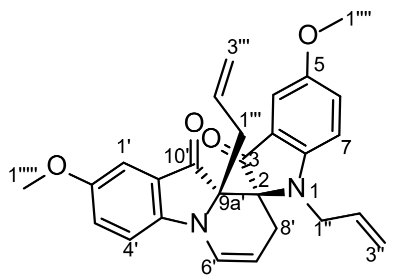

(±)-**33a**

All spectral data matched previously observed values (see section S2.4.2.1).

***transsoid*-1,9a'-diallyl-2',5-dimethoxy-8'*H*-spiro[indoline-2,9'-pyrido[1,2-*a*]indole]-3,10'(9a'*H*)-dione 33b**

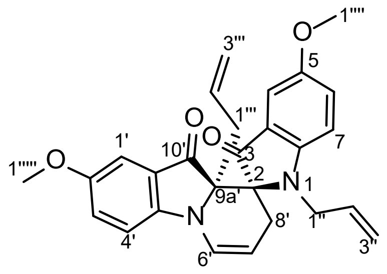

(±)-**33b**

All spectral data matched previously observed values (see section S2.4.2.1).

2.4.2.3 The Claisen rearrangement of dinitrospiroindolinepyridoindolone 25

1,9a'-diallyl-3',6-dinitro-8'*H*-spiro[indoline-2,9'-pyrido[1,2-*a*]indole]-3,10'(9a'*H*)-dione **34**

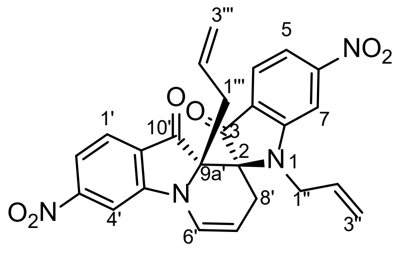

(±)-**34**

A solution of 1-allyl-10'-(allyloxy)-3',6-dinitro-8'*H*-spiro[indoline-2,9'-pyrido[1,2-*a*]indol]-3-one **25** (35 mg, 0.075 mmol) in DMF (3.0 mL) was heated at 90 °C under N_2 for 2 d, then heated for a further 3 d at 100 °C.* The reaction was quenched with brine (20 mL), extracted with EtOAc (2 x 10 mL) and the combined organic phases washed with H_2O (5 x 5 mL) and brine (2 x 5 mL), dried ($MgSO_4$) and concentrated *in vacuo*. The crude residue was adsorbed onto silica (1 g) and subjected to column chromatography (15 g silica, 20% → 40% EtOAc/hex) to yield two fractions (F_1 and F_2). Purification of F_1 by PTLC (20% EtOAc/hex) yielded 1,9a'-diallyl-3',6-dinitro-8'*H*-spiro[indoline-2,9'-pyrido[1,2-*a*]indole]-3,10'(9a'*H*)-dione **34** (14 mg) as an orange-red solid and recrystallisation of F_2 (EtOAc/hex) yielded additional **34** (5 mg; total 19 mg, 54%) as dark red radial crystals. mp 207 – 210 °C; R_f (30% EtOAc/hex) = 0.42; FTIR (thin film): ν_{max} 3089 (w), 2974 (w), 1711 (s), 1623 (s), 1529 (s), 1471 (m), 1345 (s), 1329 (s), 1099 (w), 885 (w), 735 (w); ^{1}H NMR (400 MHz, $CDCl_3$) δ 7.83 (d, *J* = 8.3 Hz, 1H, H4), 7.75 (d, *J* = 1.8 Hz, 1H, H4'), 7.67 (dd, *J* = 8.4, 1.8 Hz, 1H, H5), 7.63 (dd, *J* = 8.3, 1.8 Hz, 1H, H2'), 7.55 (d, *J* = 8.3 Hz, 1H, H1'), 7.42 (d, *J* = 1.8 Hz, 1H, H7), 6.97 (dt, *J* = 7.8, 2.0 Hz, 1H, H6'), 5.45 (ddd, *J* = 7.5, 4.7, 2.5 Hz, 1H, H7'), 5.36 – 5.23 (m, 2H, H2'', H2'''), 5.05 (appt. dq, *J* = 16.9, 1.3 Hz, 1H, H3'''a), 4.93 – 4.85 (m, 3H, H3'', H3'''b), 4.03 (ddt, *J* = 17.4, 5.0, 1.8 Hz, 1H, H1''a), 3.80 (ddt, *J* = 17.5, 5.2, 1.8 Hz, 1H, H1''b), 3.07 – 2.96 (m, 2H, H8'a, H1'''a), 2.63 (ddt, *J*

* Analysis by TLC revealed no conversion from SM to products had occurred after heating for 2 d at 90 °C.

= 14.0, 7.6, 1.0 Hz, 1H, H1'''b), 2.21 (ddd, J = 19.5, 4.7, 1.8 Hz, 1H, H8'b); ^{13}C NMR (101 MHz, $CDCl_3$) δ 198.5 (C3), 197.4 (C10'), 159.5 (C7a), 155.2 (C4a'), 154.1 (C3'), 153.8 (C6), 132.0 (C2''), 129.1 (C2'''), 125.8 (C3a), 125.6 (C1'), 125.2 (C10a'), 125.1 (C4), 121.8 (C6'), 121.0 (C3'''), 117.5 (C3''), 114.4 (C2'), 113.8 (C5), 106.6 (C7'), 105.8 (C7), 103.4 (C4'), 74.7 (C9a'), 69.6 (C2/C9'), 47.4 (C1''), 34.8 (C1'''), 29.6 (C8'); LRMS (EI) m/z 472 (100%) $[M]^{\bullet+}$; HRMS (ESI$^+$) Calculated for $C_{25}H_{21}N_4O_6^+$ $[M+H]^+$ 473.1461, found 473.1466.

2.4.3 The cascade reaction of 6,6'-dinitroindigo 1i in MeCN

A suspension of 6,6'-dinitroindigo **1i** (21 mg, 0.060 mmol) in anhydrous MeCN (2.4 mL) was sonicated for 60 min under a N_2 atmosphere. The suspension was transferred to a flask containing pre-dried Cs_2CO_3 (78 mg, 0.240 mmol) and activated 3 Å M.S. (120 mg) and plunged into a pre-heated oil bath at 85 – 88 °C under N_2 with stirring. After 60 min, the N_2 flow was cut and allyl bromide (0.22 mL, 310 mg, 2.56 mmol) was added and stirred. Monitoring the reaction by TLC after 20 min (see section S1.2) revealed the reaction was proceeding identically to the equivalent reaction in DMF (see section S2.3.10) and was therefore quenched and disposed.

S3. NMR Characterisation

S3.1 NMR spectra of substituted indigos and key intermediates

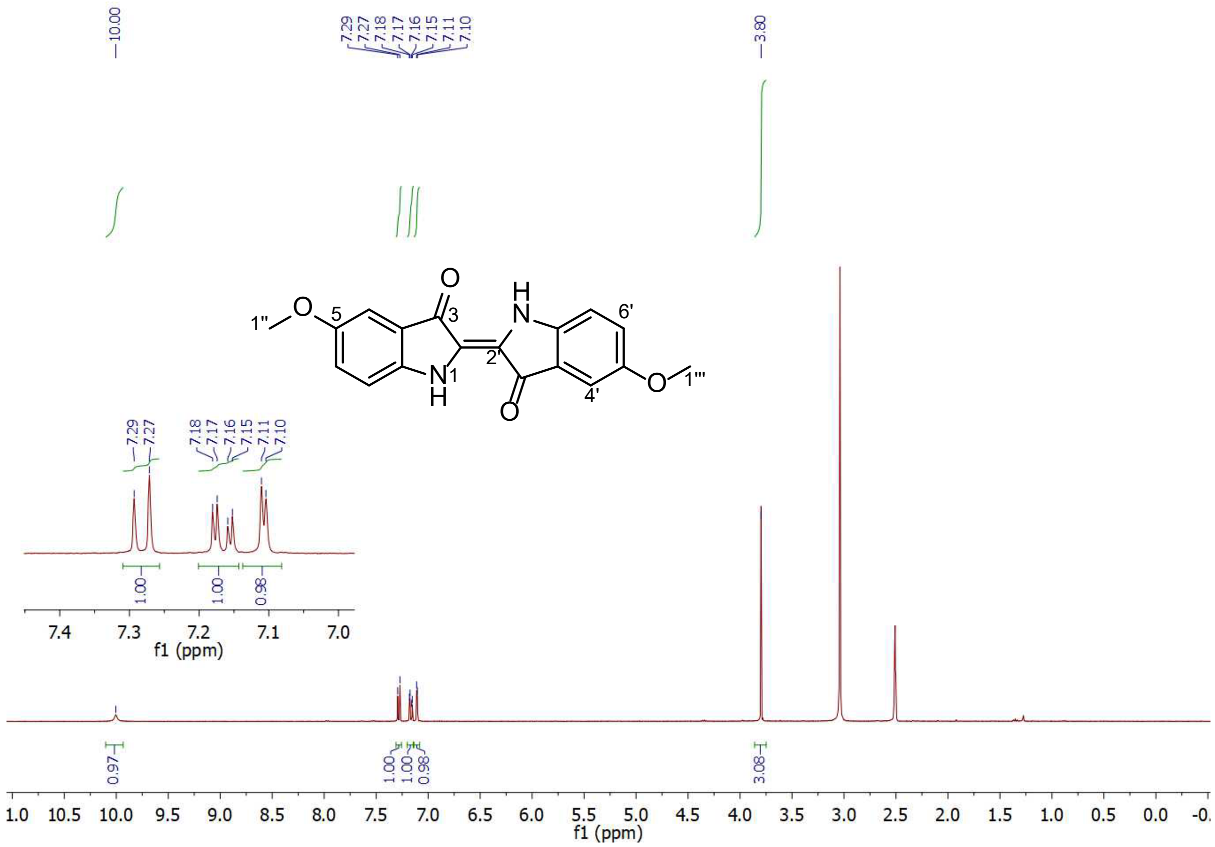

Figure S1: The ^{1}H NMR spectrum of 5,5'-dimethoxyindigo **1a** (400 MHz, DMSO-d_6, 80 °C).

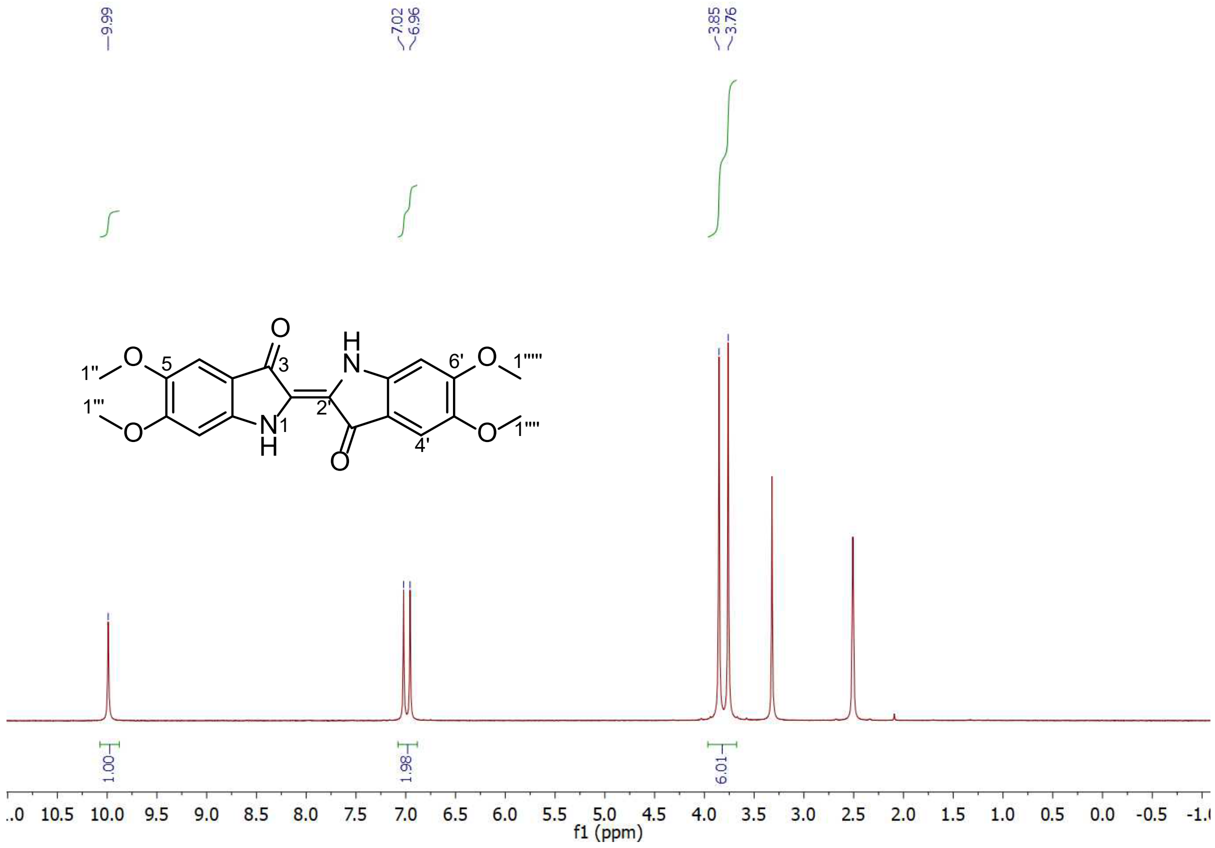

Figure S2: The ^{1}H NMR spectrum of 5,5',6,6'-tetramethoxyindigo **1b** (400 MHz, DMSO-d_6, 80 °C).

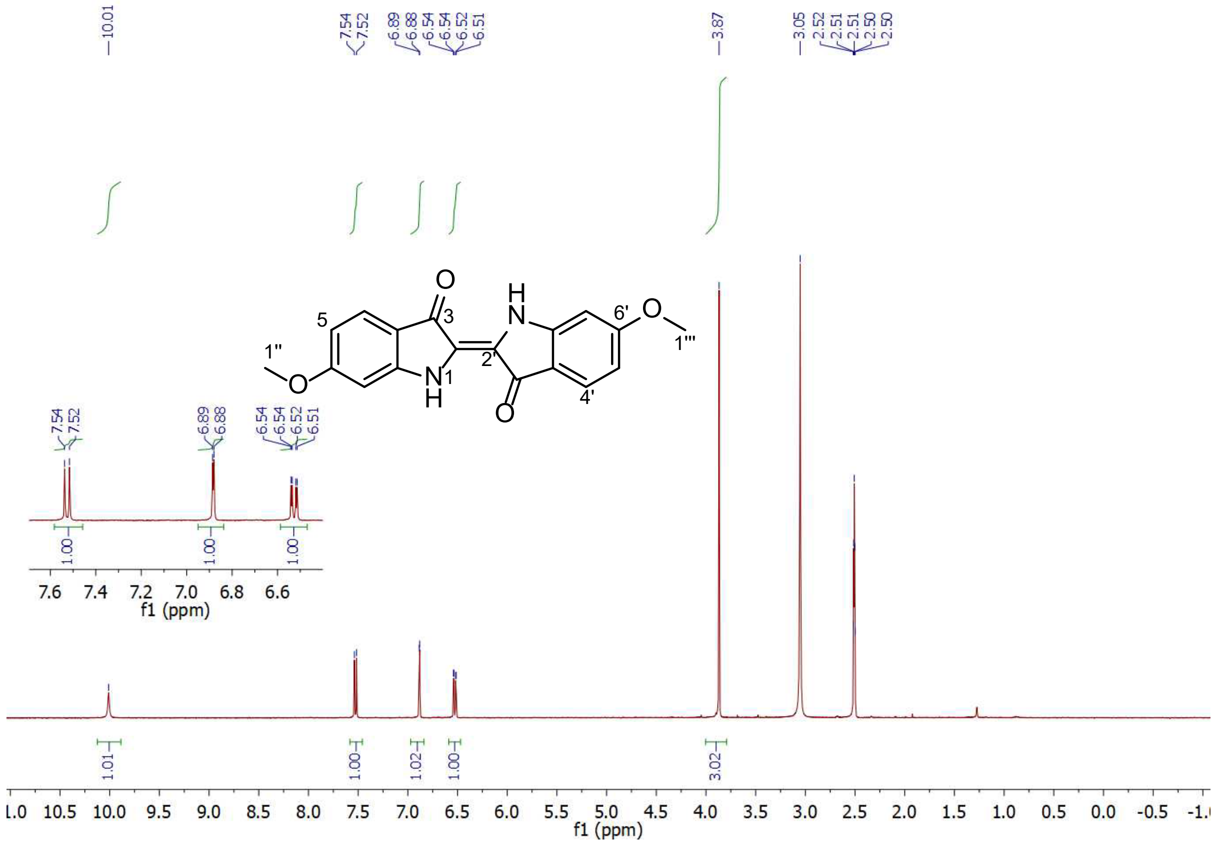

Figure S3: The ^{1}H NMR spectrum of 6,6'-dimethoxyindigo **1c** (400 MHz, DMSO-d_6, 80 °C).

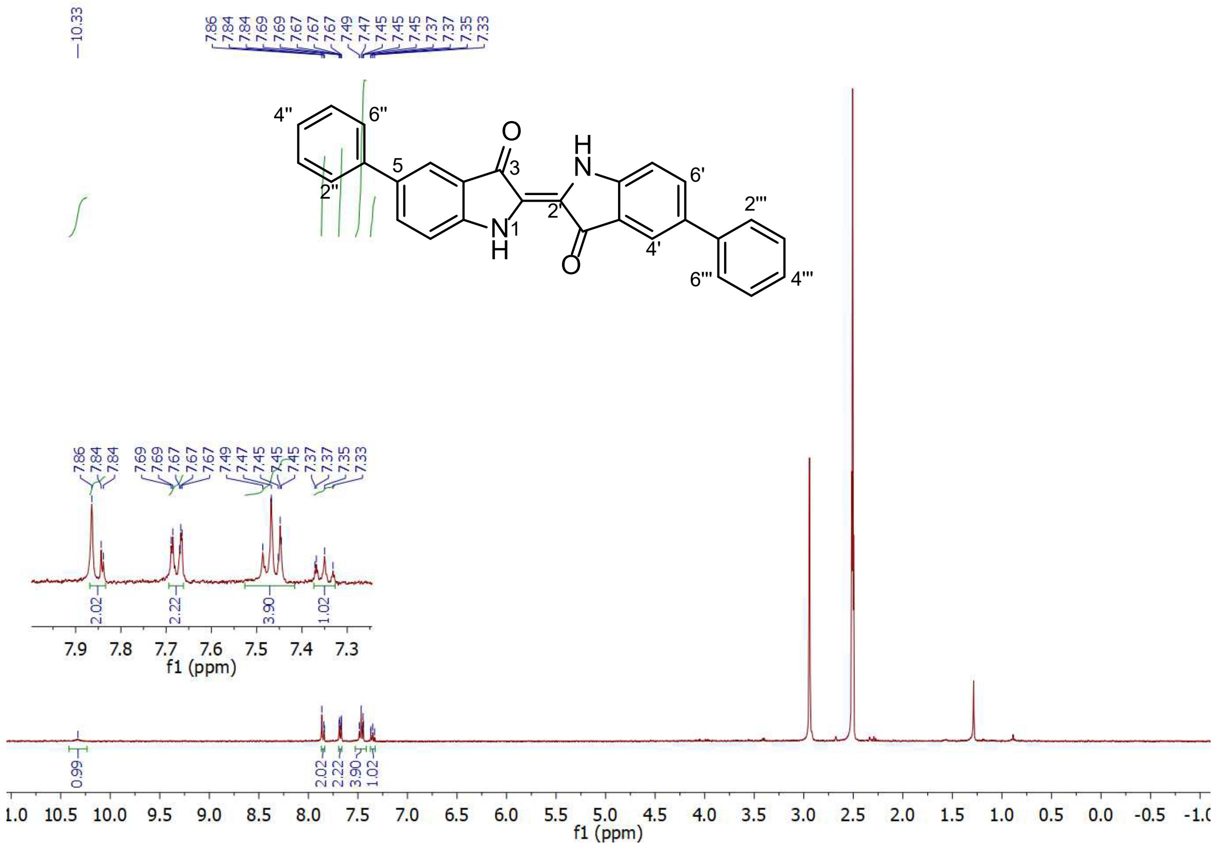

Figure S4: The ^{1}H NMR spectrum of 5,5'-diphenylindigo **1d** (400 MHz, DMSO-d_6, 90 °C).

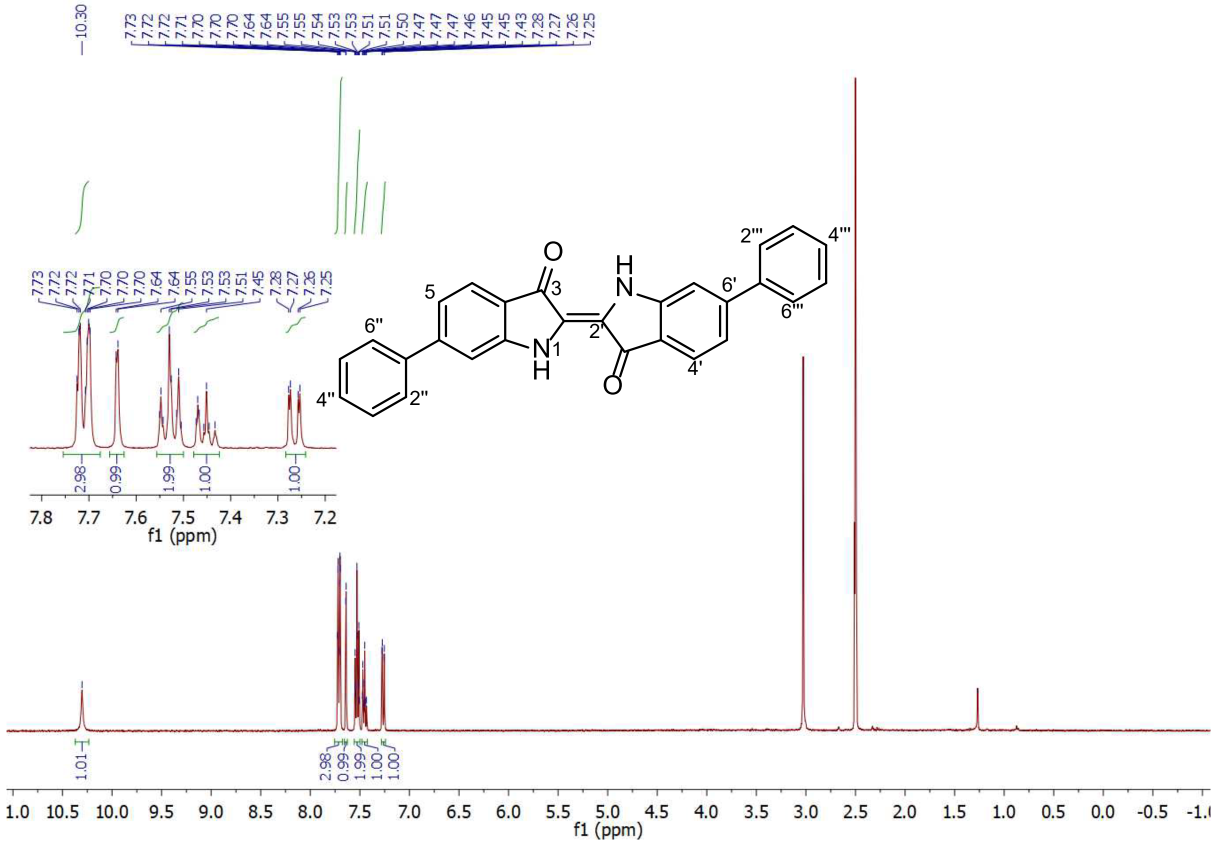

Figure S5: The ^{1}H NMR spectrum of 6,6'-diphenylindigo **1e** (400 MHz, DMSO-d_6, 80 °C).

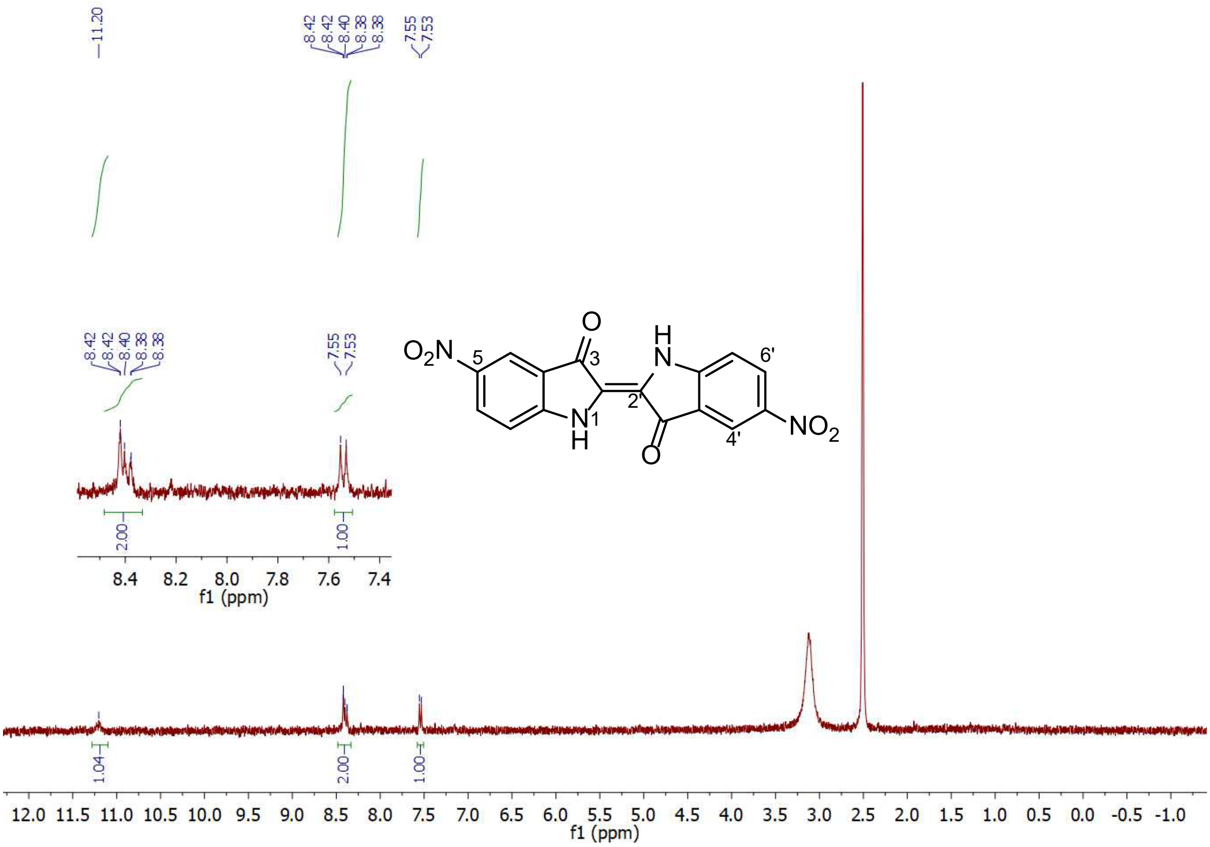

Figure S6: The ^{1}H NMR spectrum of 5,5'-dinitroindigo **1h** (400 MHz, DMSO-d_6, 90 °C). The large H_2O and DMSO resonances in this spectrum arise from the insolubility of compound **1h** leading to exaggerated resonances from the solvent. To obtain this spectrum, **1h** was suspended in DMSO-d_6, sonicated for 1 h, heated at 120 °C for 1 h, then the spectrum was obtained.

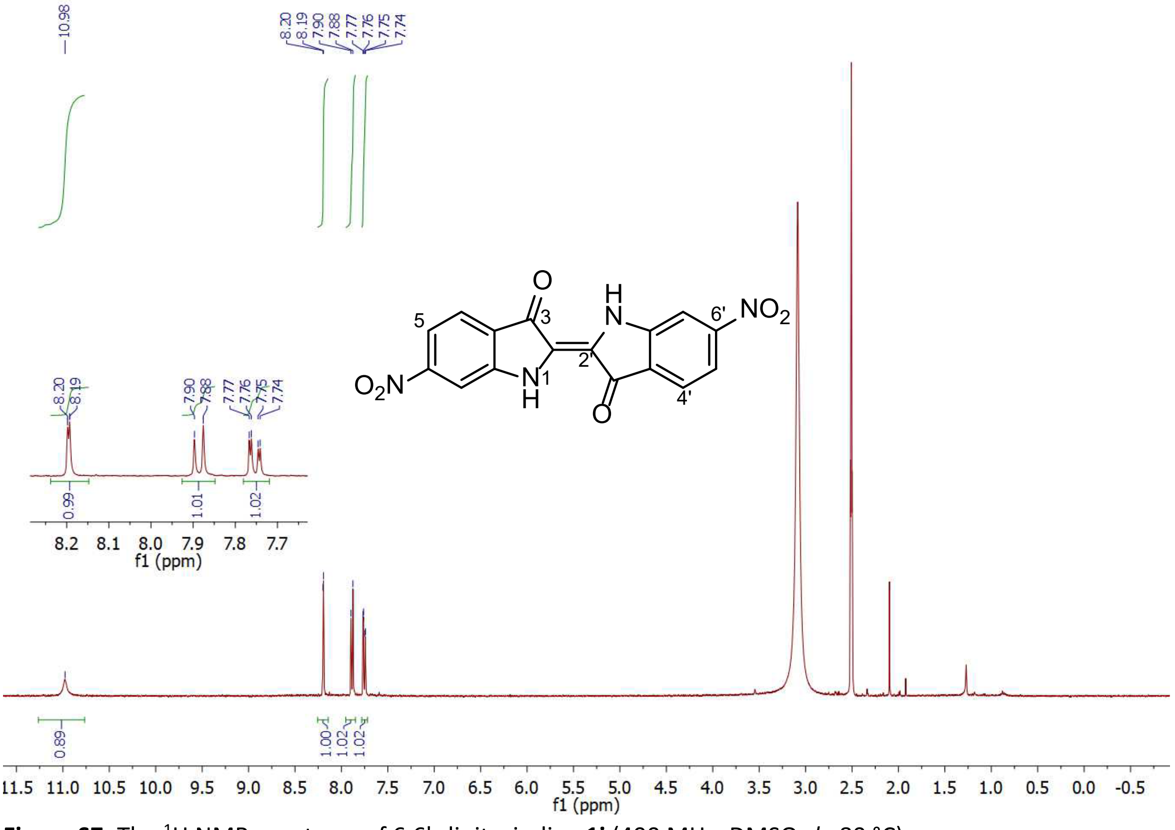

Figure S7: The ^{1}H NMR spectrum of 6,6'-dinitroindigo **1i** (400 MHz, DMSO-d_6, 80 °C).

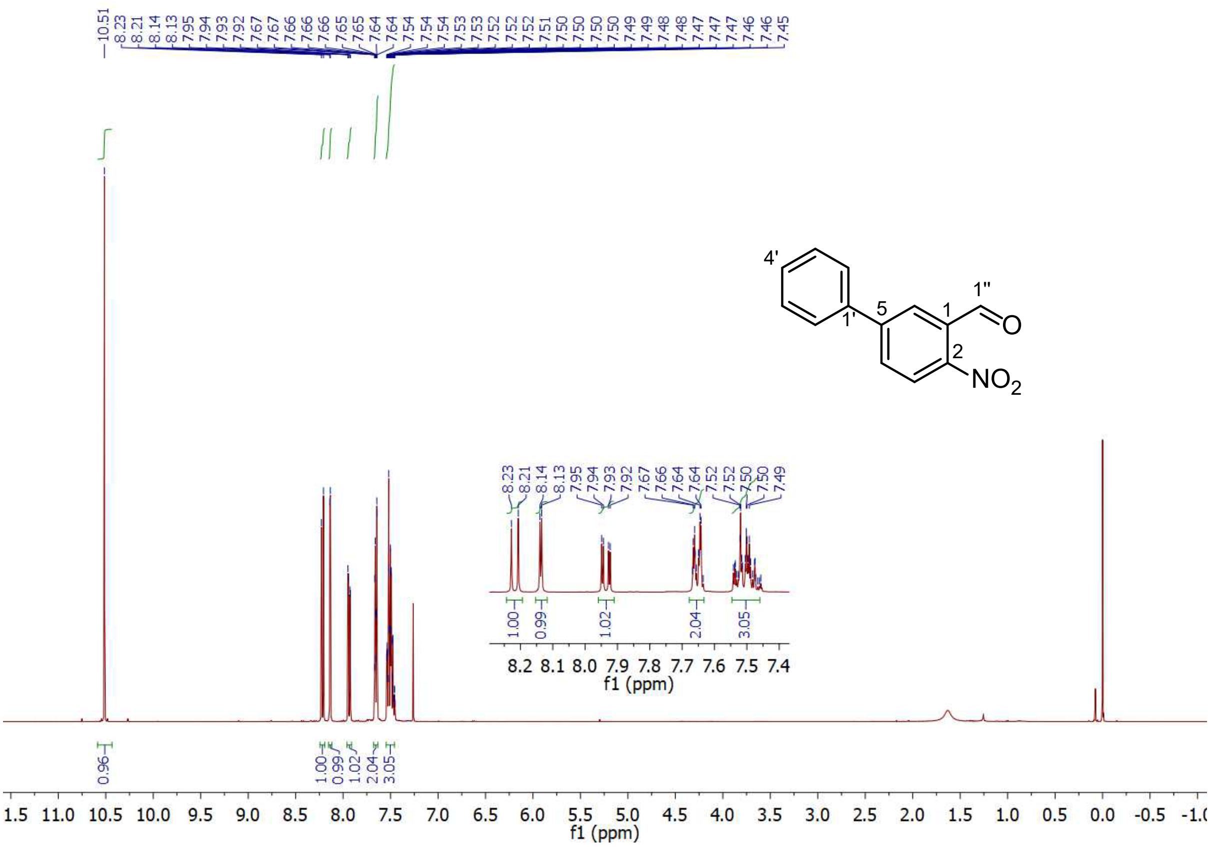

Figure S8: The ^{1}H NMR spectrum of 2-nitro-5-phenylbenzaldehyde **8d** (400 MHz, $CDCl_3$).

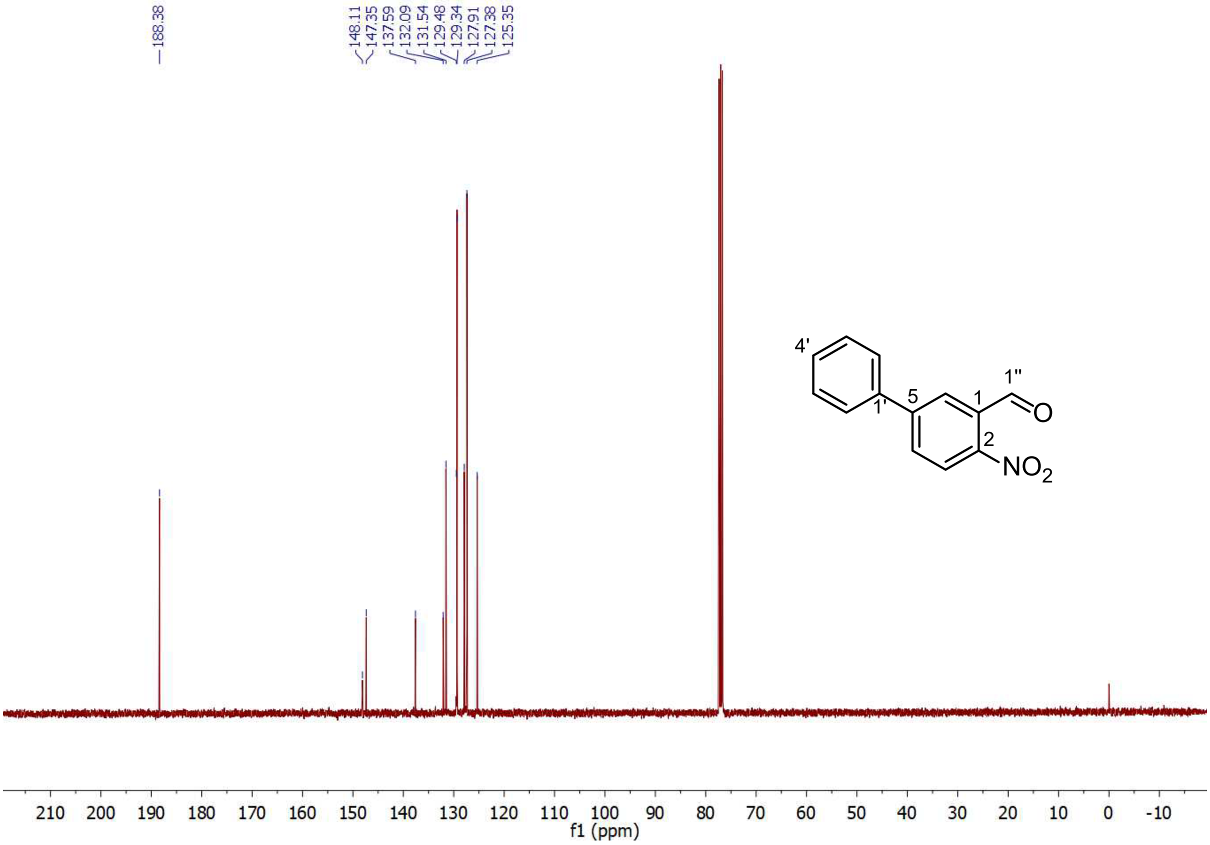

Figure S9: The ^{13}C NMR spectrum of 2-nitro-5-phenylbenzaldehyde **8d** (101 MHz, $CDCl_3$).

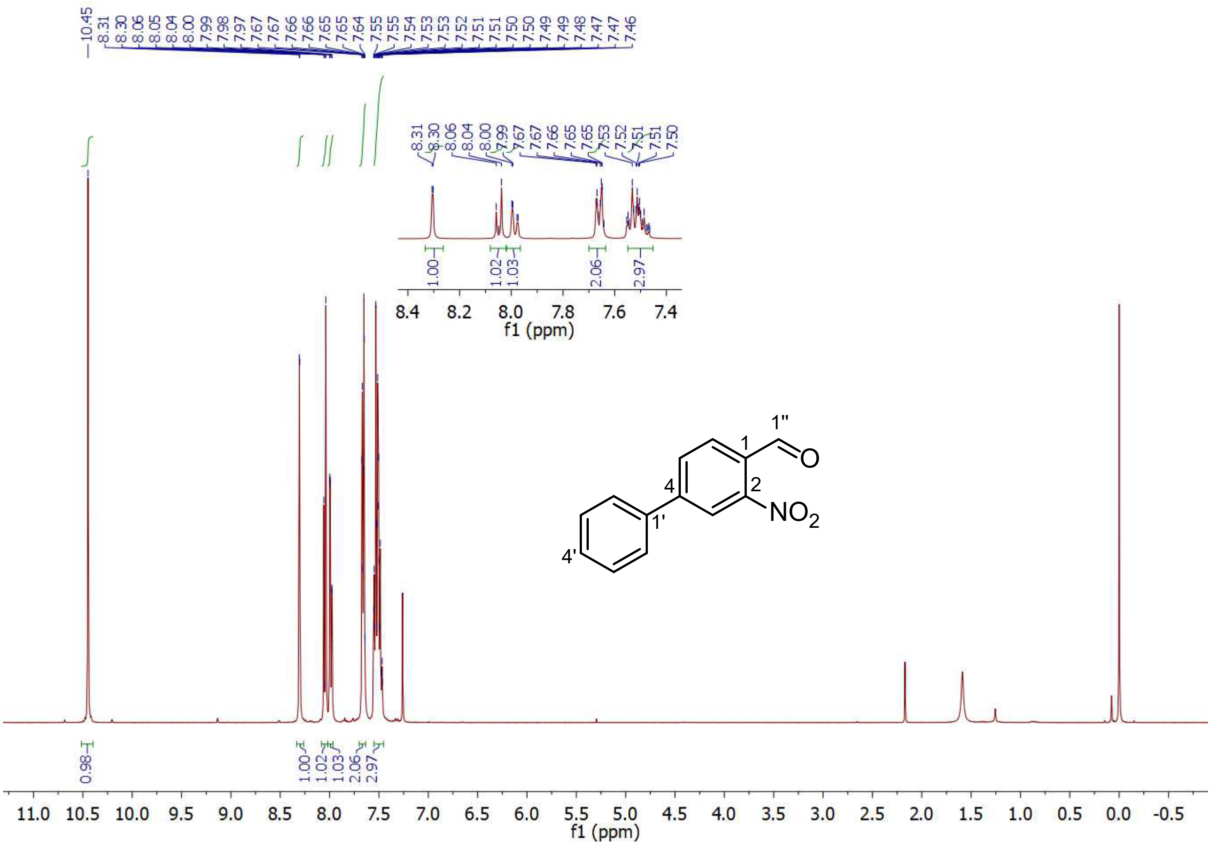

Figure S10: The ^{1}H NMR spectrum of 2-nitro-4-phenylbenzaldehyde **8e** (400 MHz, $CDCl_3$).

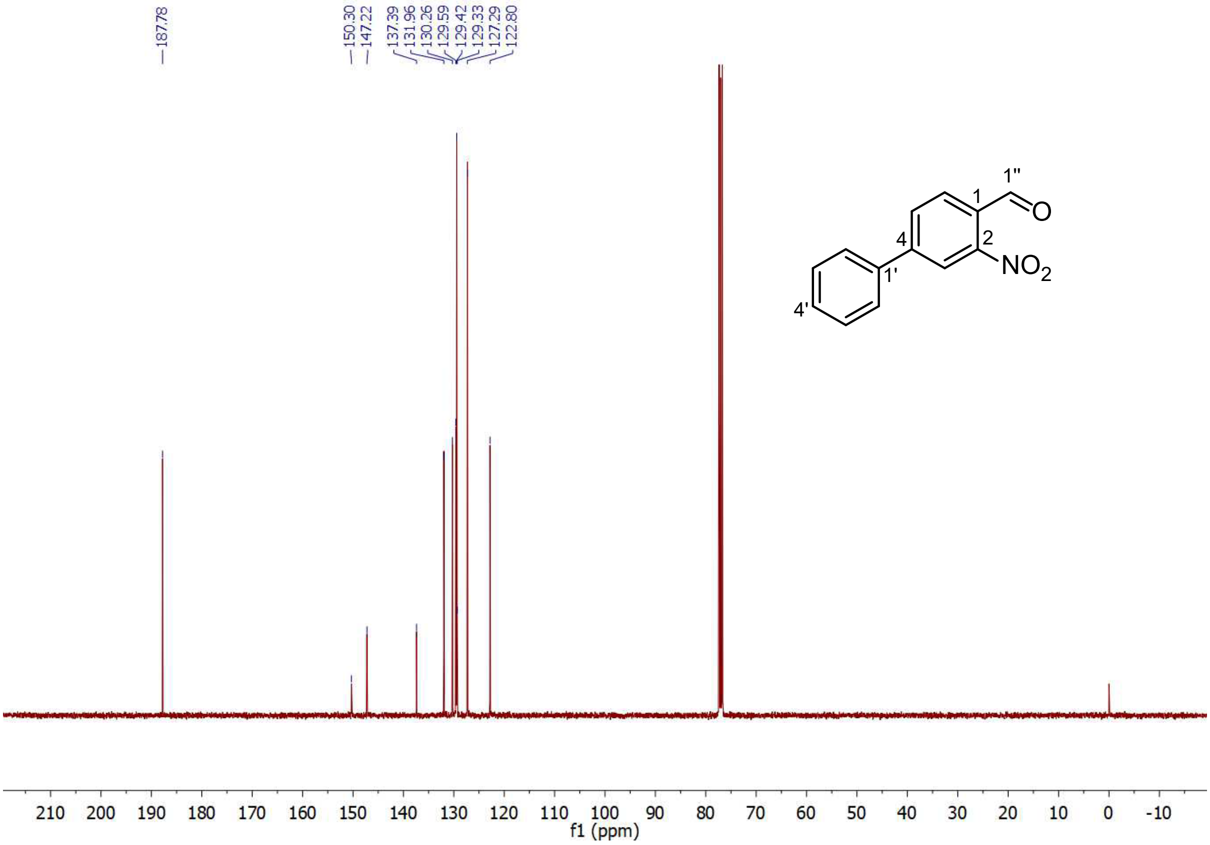

Figure S11: The ^{13}C NMR spectrum of 2-nitro-4-phenylbenzaldehyde **8e** (101 MHz, $CDCl_3$).

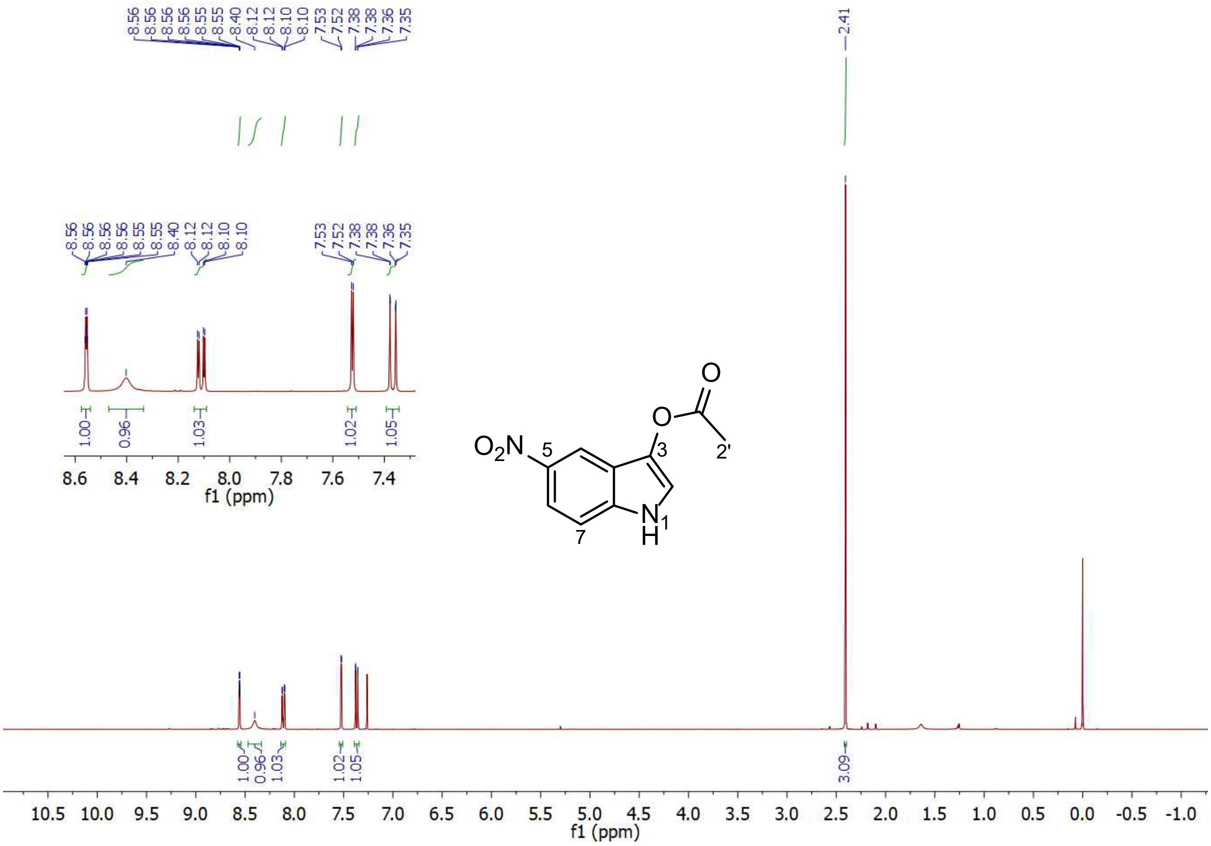

Figure S12: The ^{1}H NMR spectrum of 3-acetoxy-5-nitroindole **10** (400 MHz, $CDCl_3$).

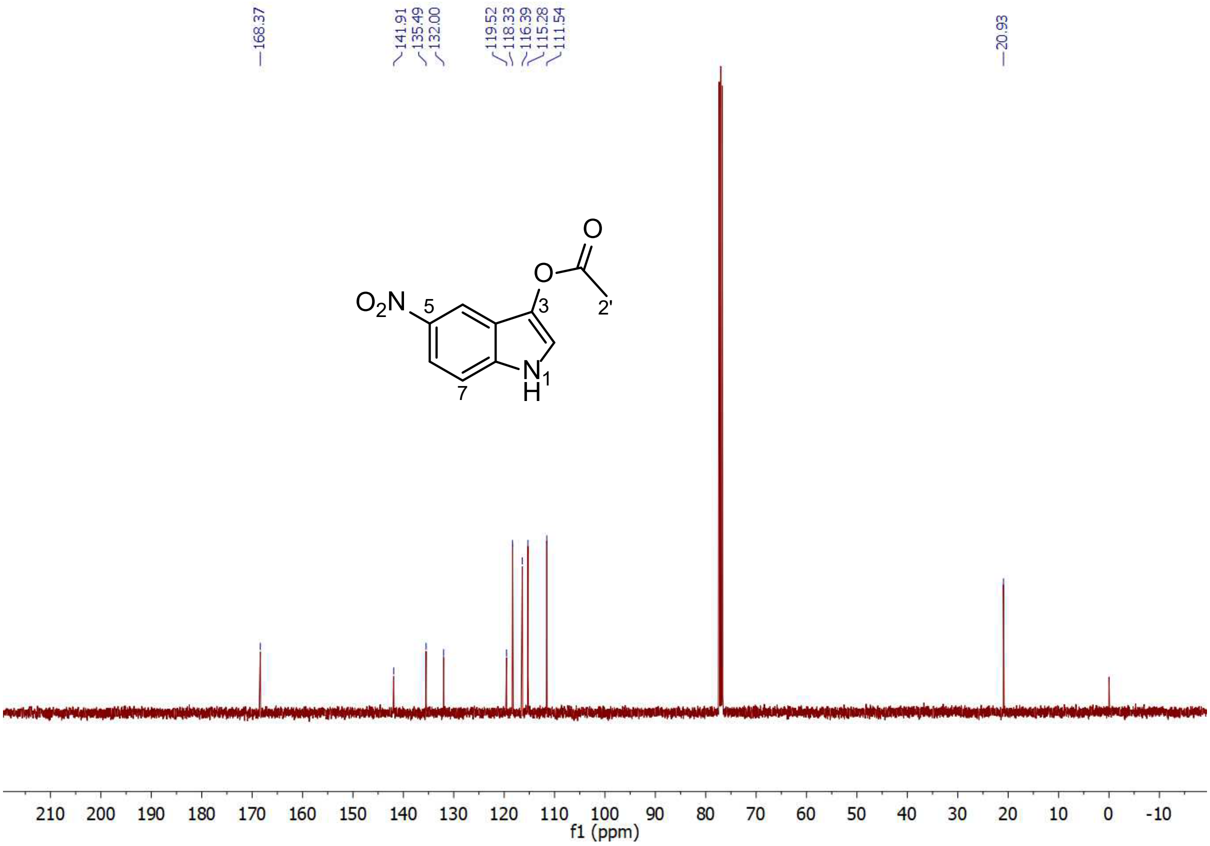

Figure S13: The ^{13}C NMR spectrum of 3-acetoxy-5-nitroindole **10** (101 MHz, $CDCl_3$).

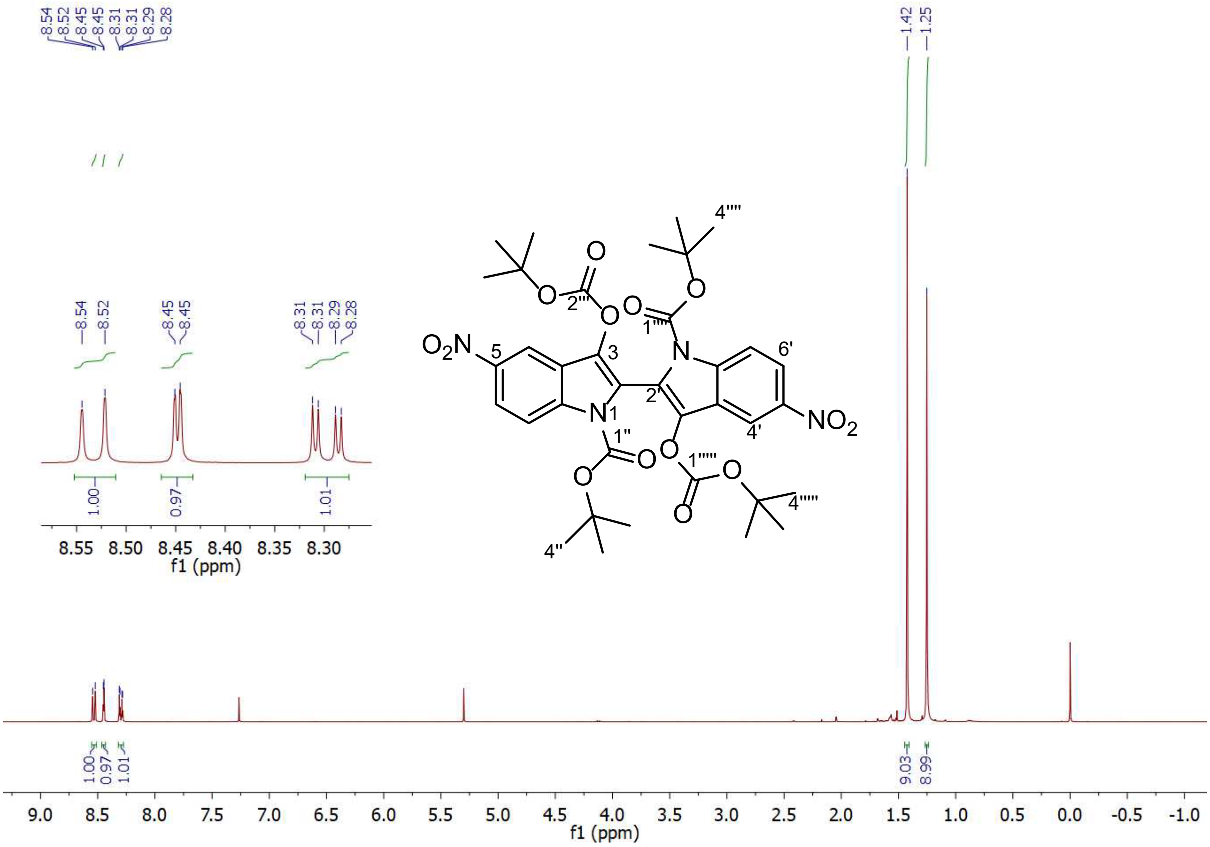

Figure S14: The ^{1}H NMR spectrum of *N*,*N'*,*O*,*O'*-tetraBoc-5,5'-dinitrobiindole-3,3'-diol **11** (400 MHz, $CDCl_3$).

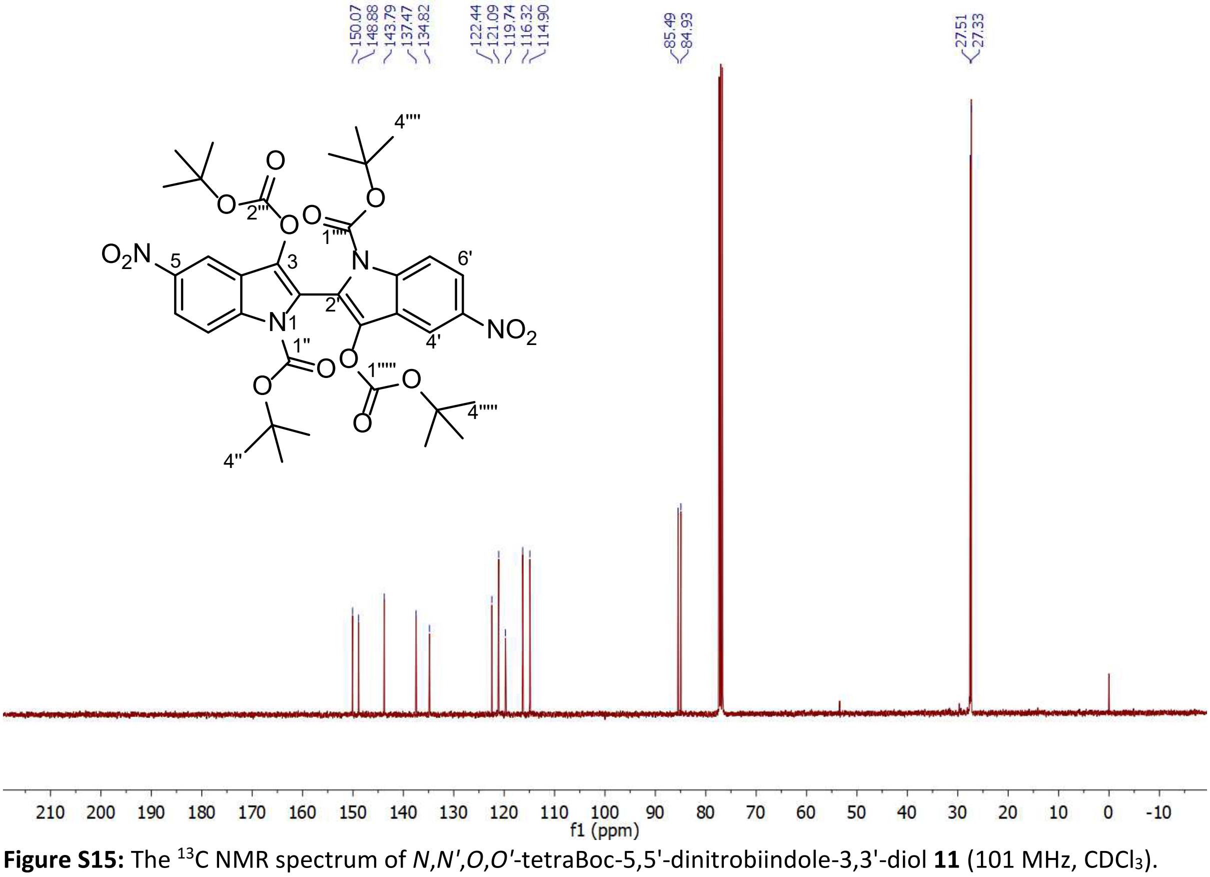

Figure S15: The ^{13}C NMR spectrum of *N*,*N'*,*O*,*O'*-tetraBoc-5,5'-dinitrobiindole-3,3'-diol **11** (101 MHz, $CDCl_3$).

``

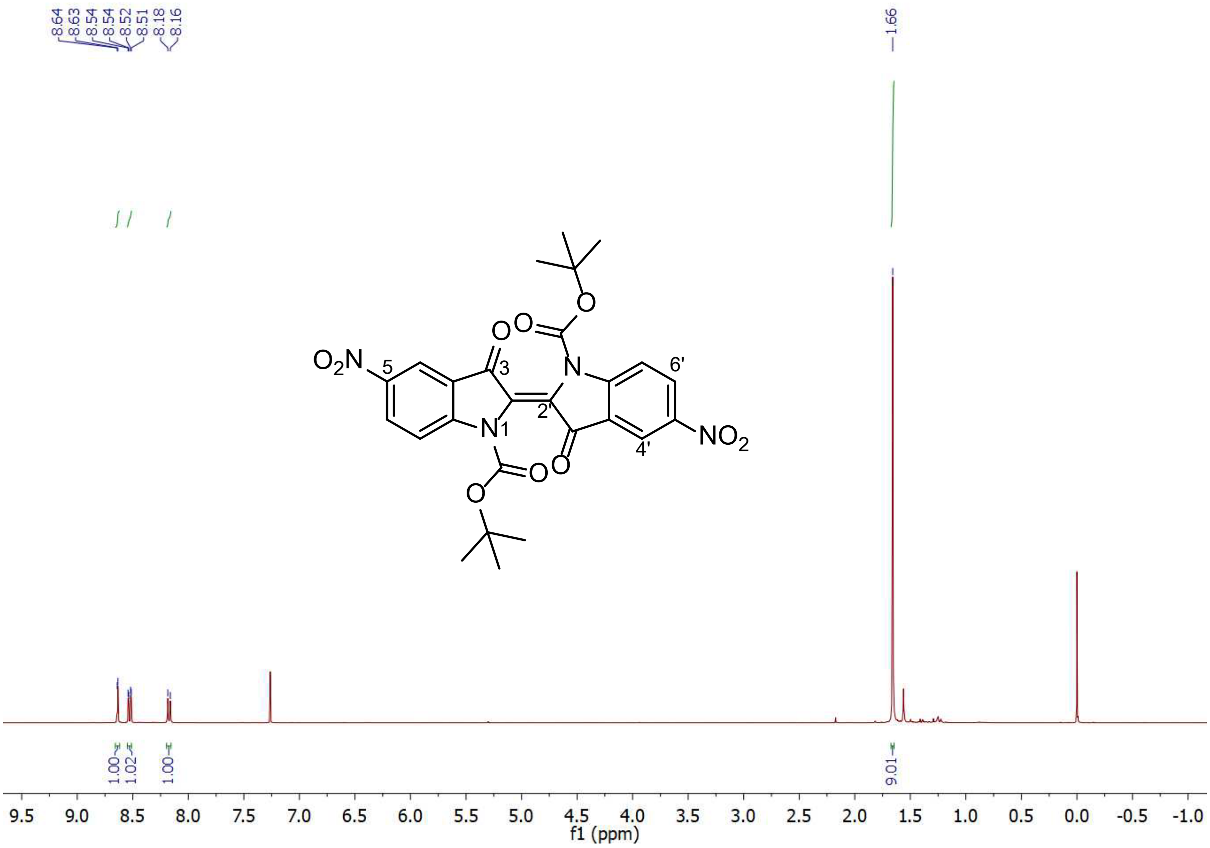

Figure S16: The ^{1}H NMR spectrum of *N*,*N'*-diBoc-5,5'-dinitroindigo **12** (400 MHz, $CDCl_3$).

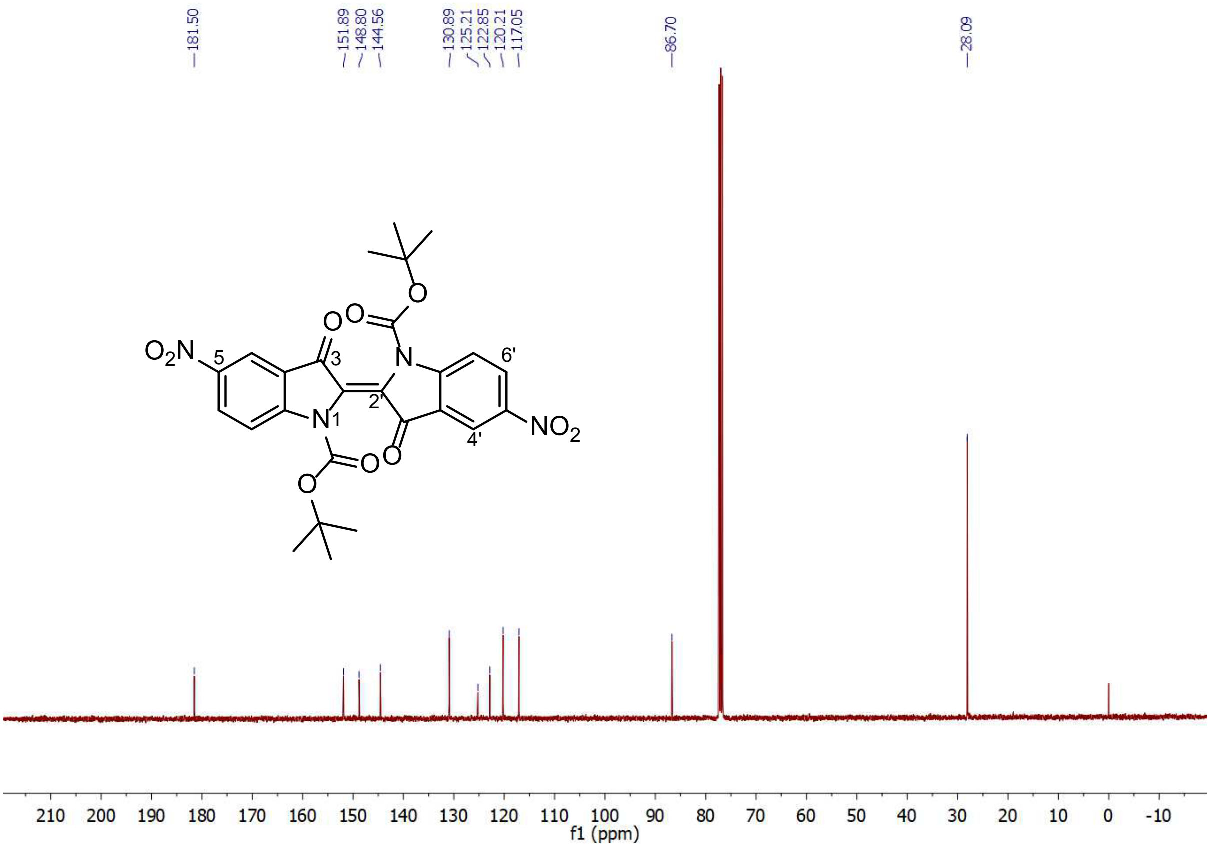

Figure S17: The ^{13}C NMR spectrum of *N*,*N'*-diBoc-5,5'-dinitroindigo **12** (101 MHz, $CDCl_3$).

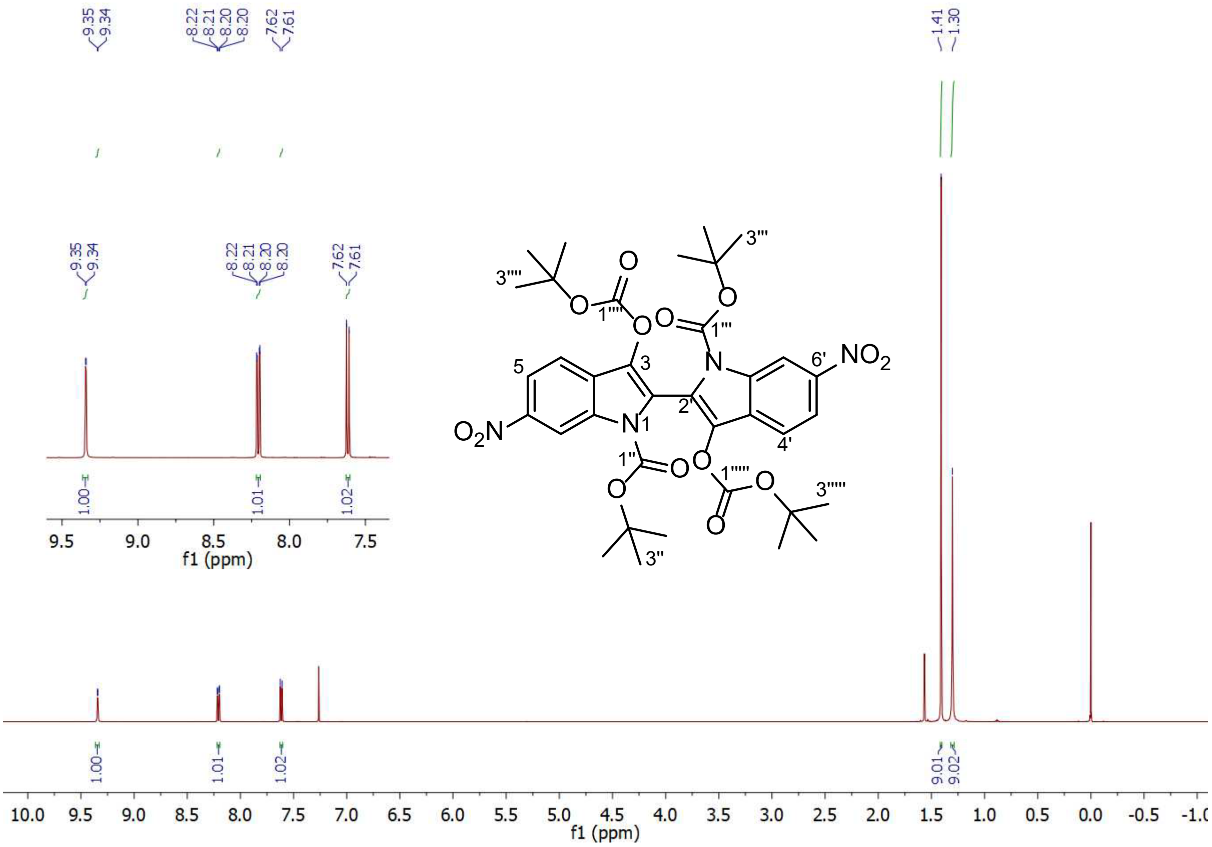

Figure S18: The ^{1}H NMR spectrum of *N*,*N'*,*O*,*O'*-tetraBoc-6,6'-dinitrobiindole-3,3'-diol **13** (500 MHz, $CDCl_3$).

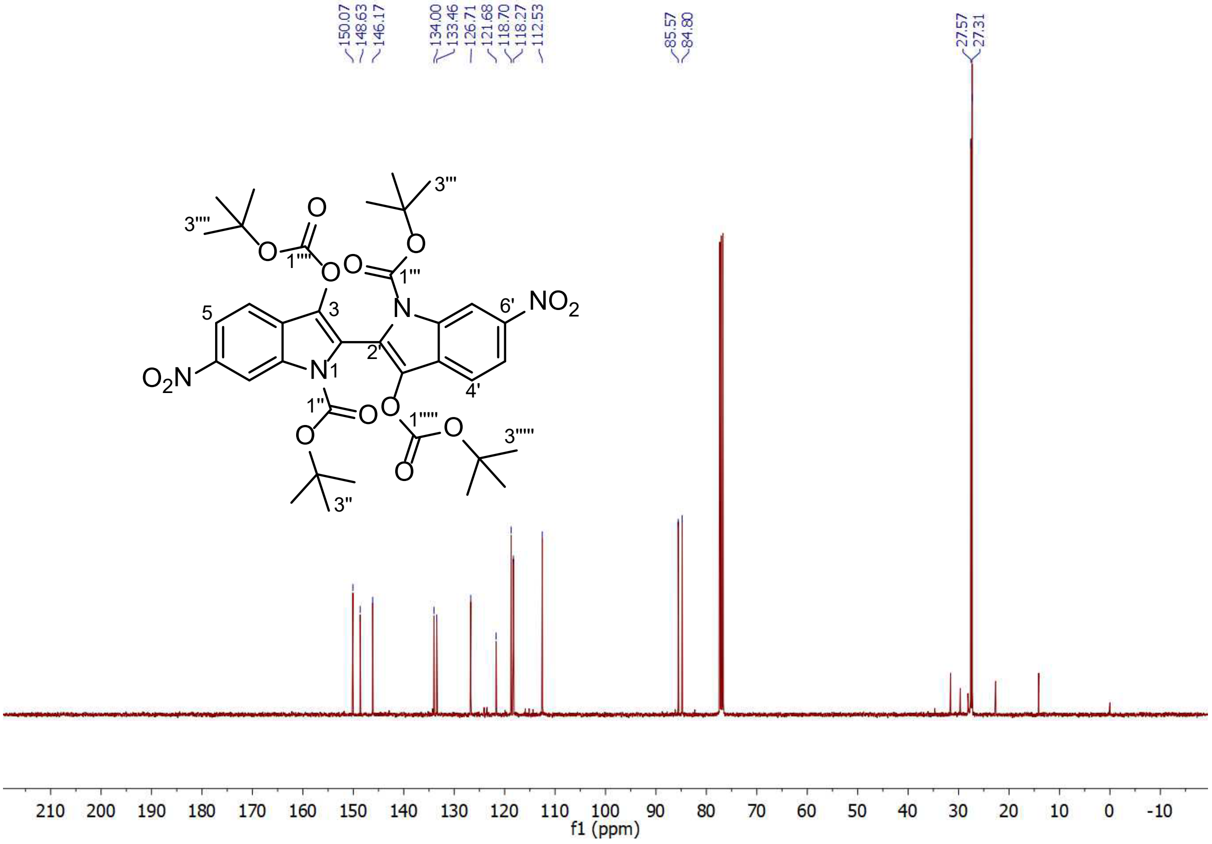

Figure S19: The ^{13}C NMR spectrum of *N*,*N'*,*O*,*O'*-tetraBoc-6,6'-dinitrobiindole-3,3'-diol **13** (101 MHz, $CDCl_3$).

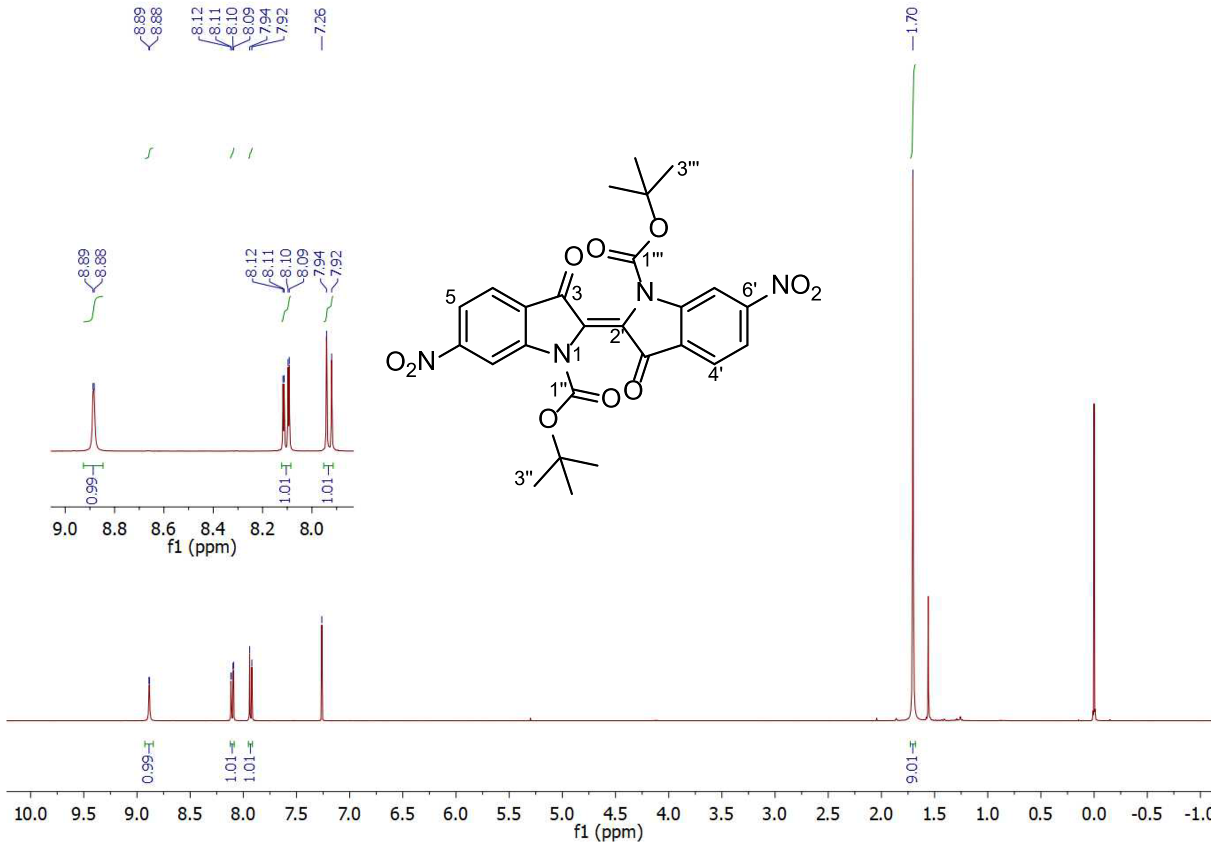

Figure S20: The ^1H NMR spectrum of *N*,*N'*-diBoc-6,6'-dinitroindigo **14** (400 MHz, $CDCl_3$).

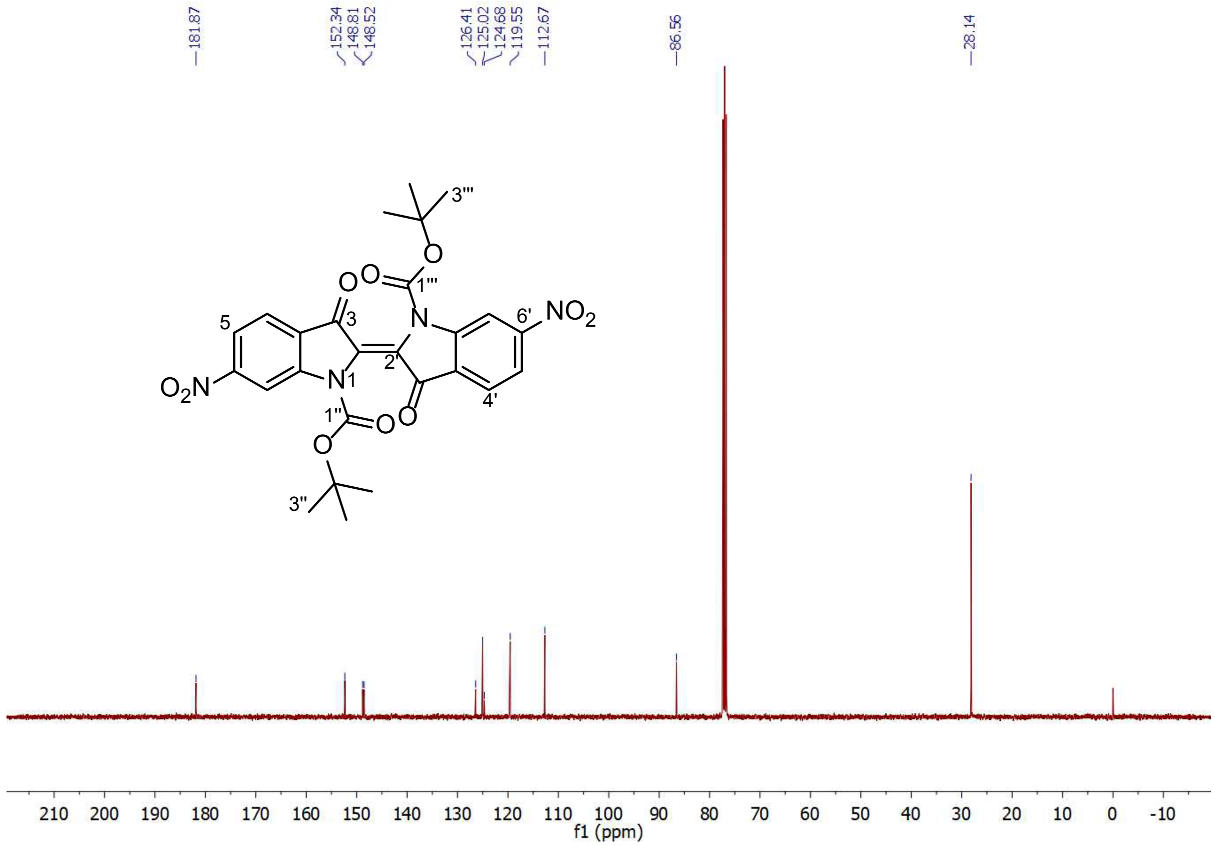

Figure S21: The ^{13}C NMR spectrum of *N*,*N'*-diBoc-6,6'-dinitroindigo **14** (101 MHz, $CDCl_3$).

S3.2 NMR spectra of allylation cascade products

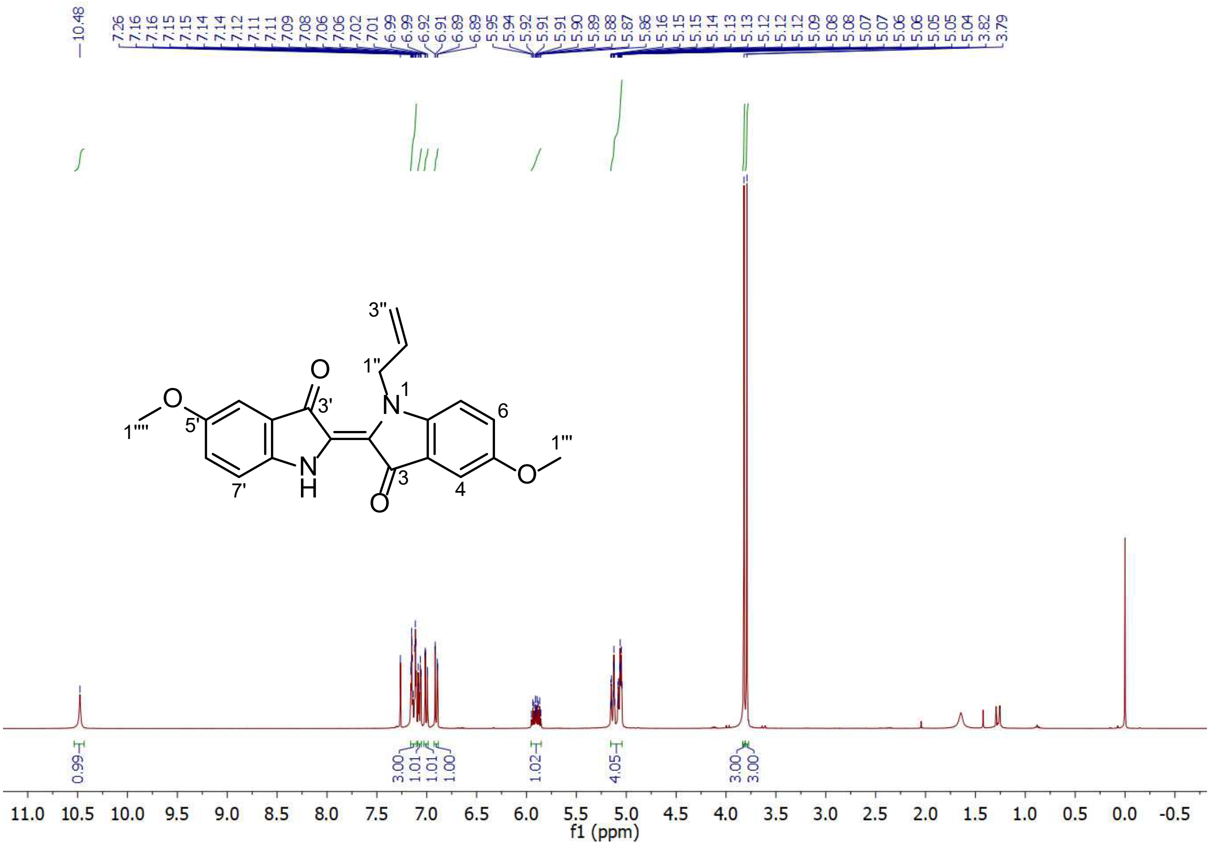

Figure S22: The ^{1}H NMR spectrum of *N*-allyl-5,5'-dimethoxyindigo **15** (500 MHz, $CDCl_3$).

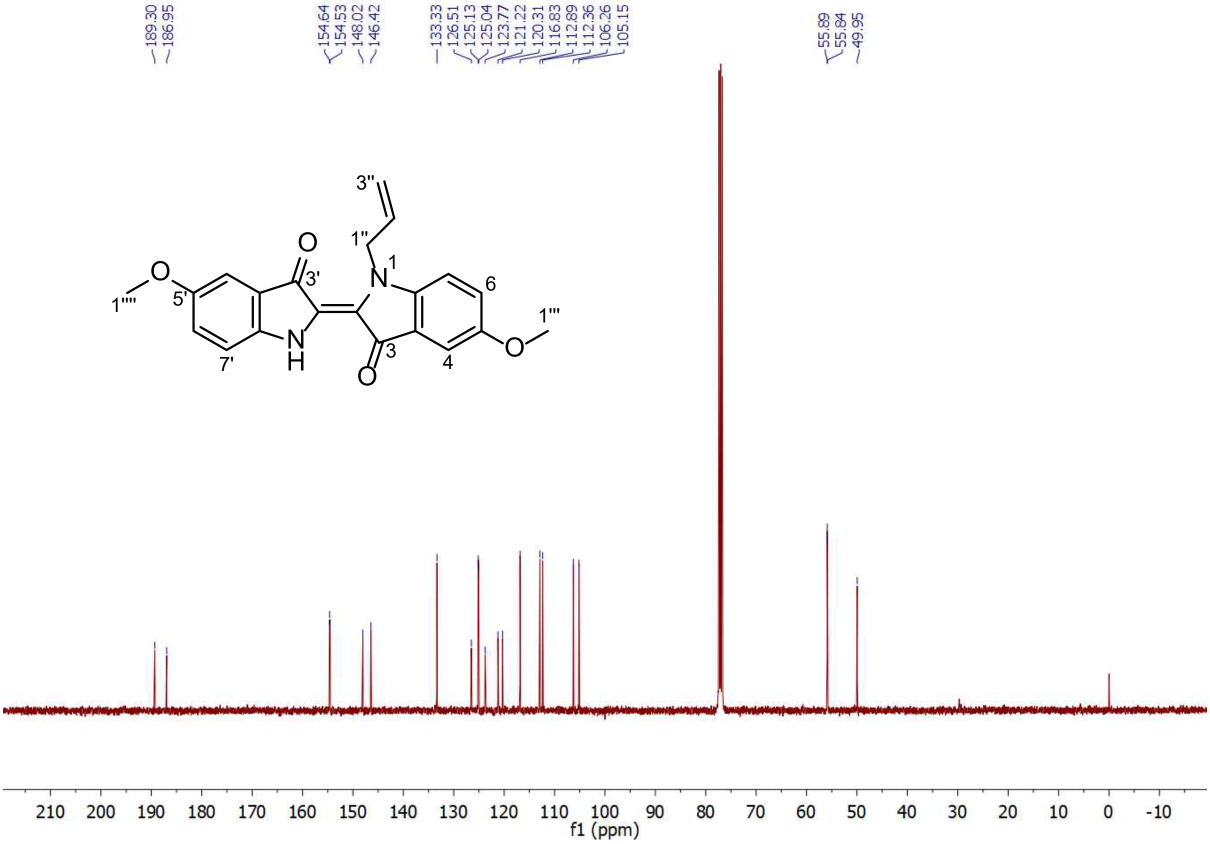

Figure S23: The ^{13}C NMR spectrum of *N*-allyl-5,5'-dimethoxyindigo **15** (126 MHz, $CDCl_3$).

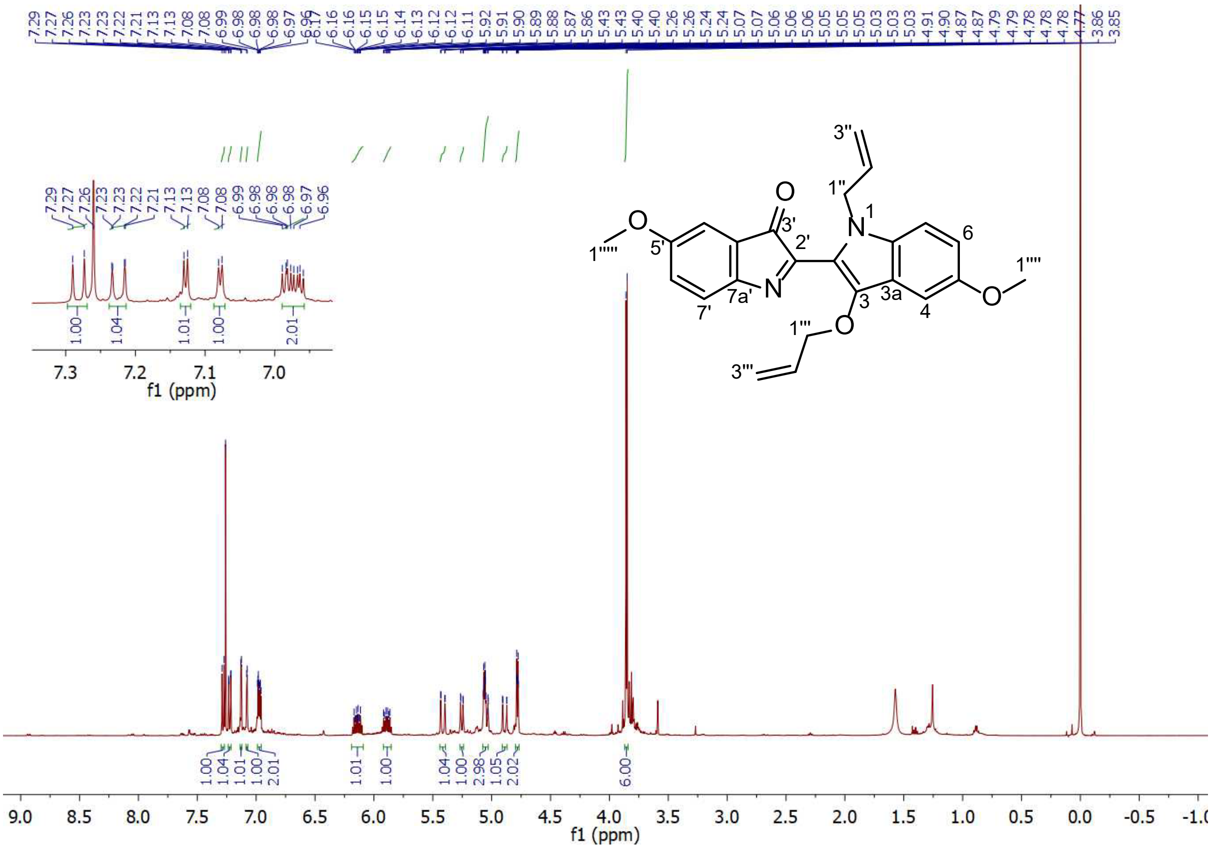

Figure S24: The ^{1}H NMR spectrum of 5,5'-dimethoxydiallylbiindolone **16** (500 MHz, $CDCl_3$).

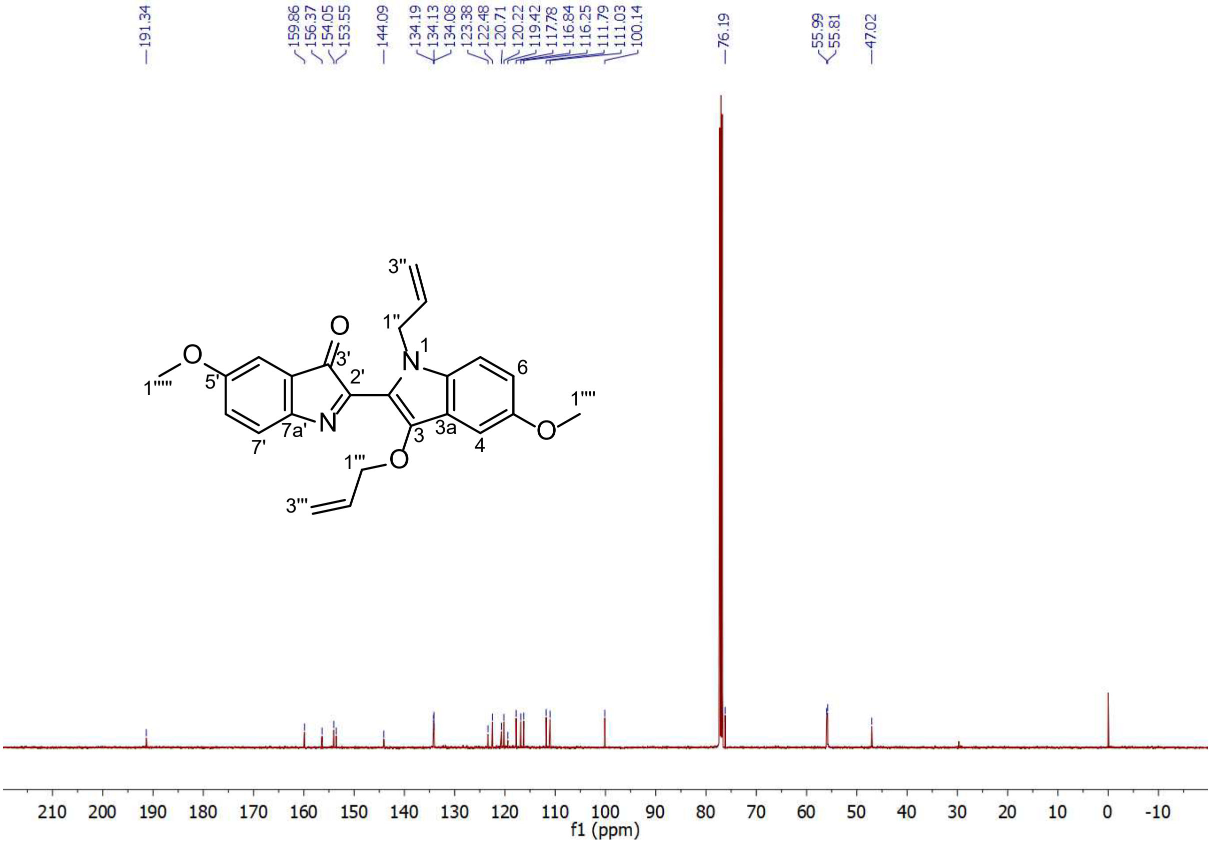

Figure S25: The ^{13}C NMR spectrum of 5,5'-dimethoxydiallylbiindolone **16** (126 MHz, $CDCl_3$).

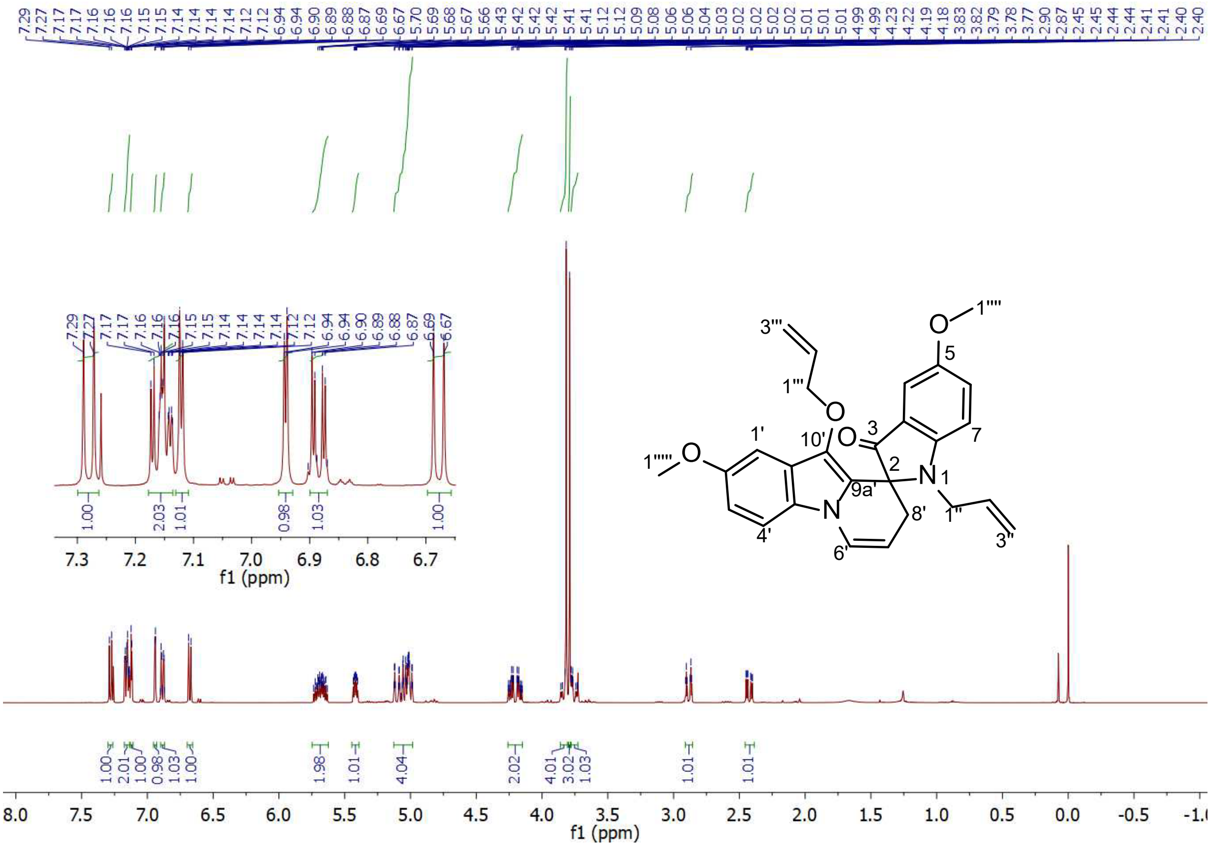

Figure S26: The ^{1}H NMR spectrum of 5,2'-dimethoxyspiroindolinepyridoindolone **17** (500 MHz, $CDCl_3$).

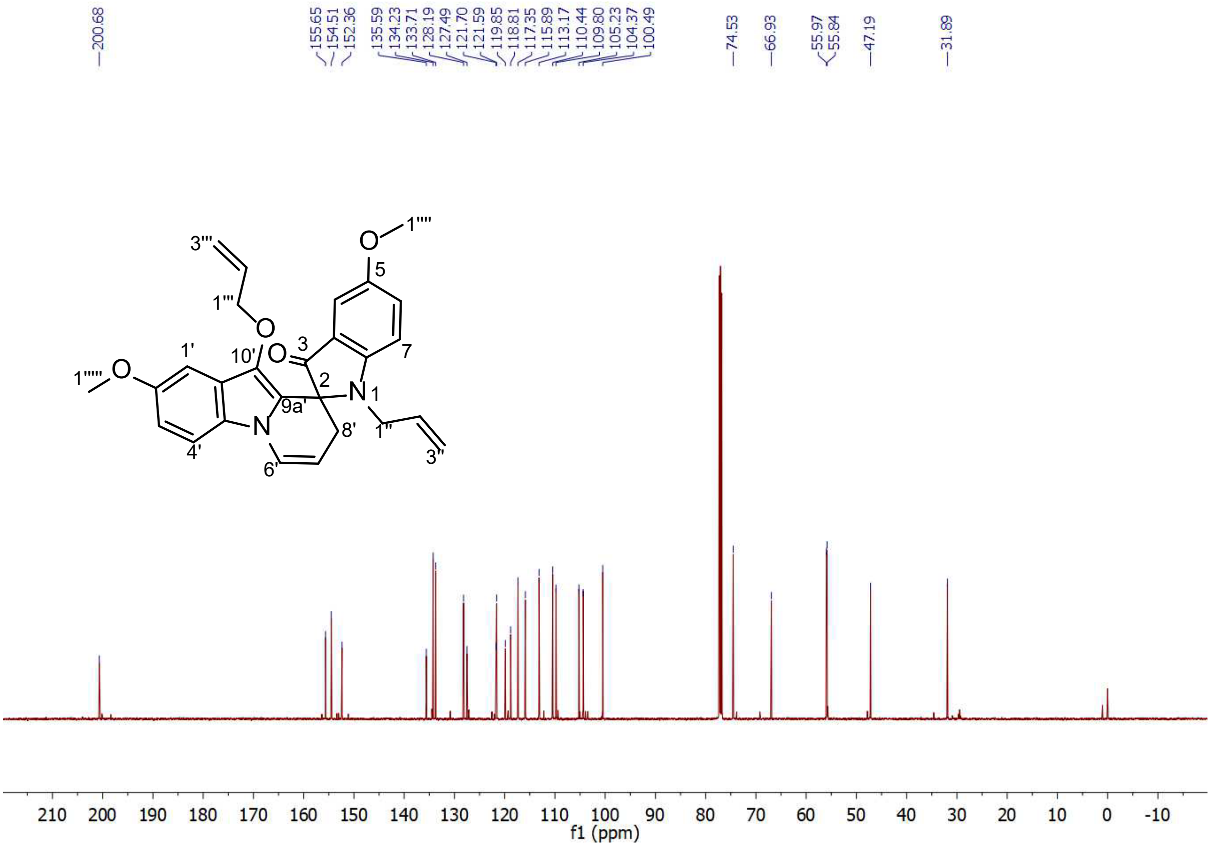

Figure S27: The ^{13}C NMR spectrum of 5,2'-dimethoxyspiroindolinepyridoindolone **17** (126 MHz, $CDCl_3$).

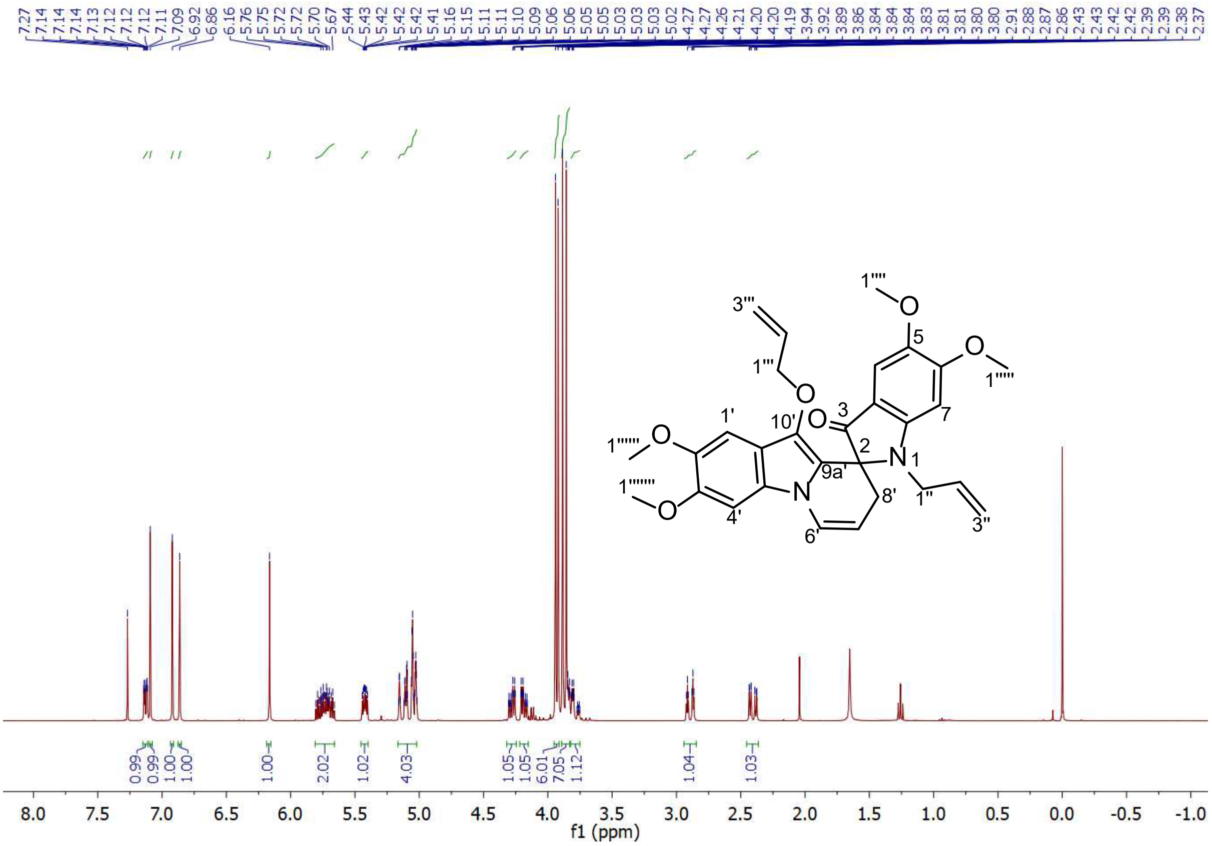

Figure S28: The ^{1}H NMR spectrum of 5,6,2',3'-tetramethoxyspiroindolinepyridoindolone **18** (400 MHz, $CDCl_3$).

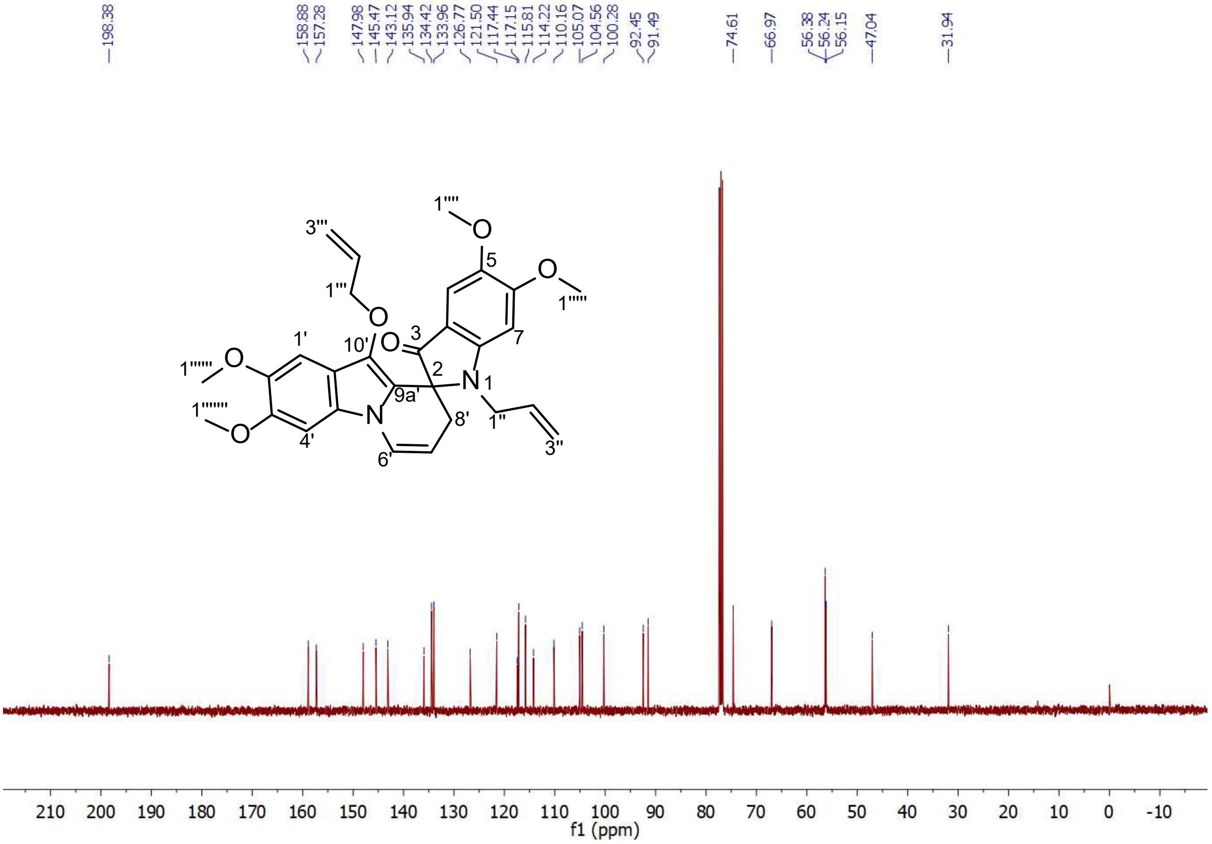

Figure S29: The ^{13}C NMR spectrum of 5,6,2',3'-tetramethoxyspiroindolinepyridoindolone **18** (101 MHz, $CDCl_3$).

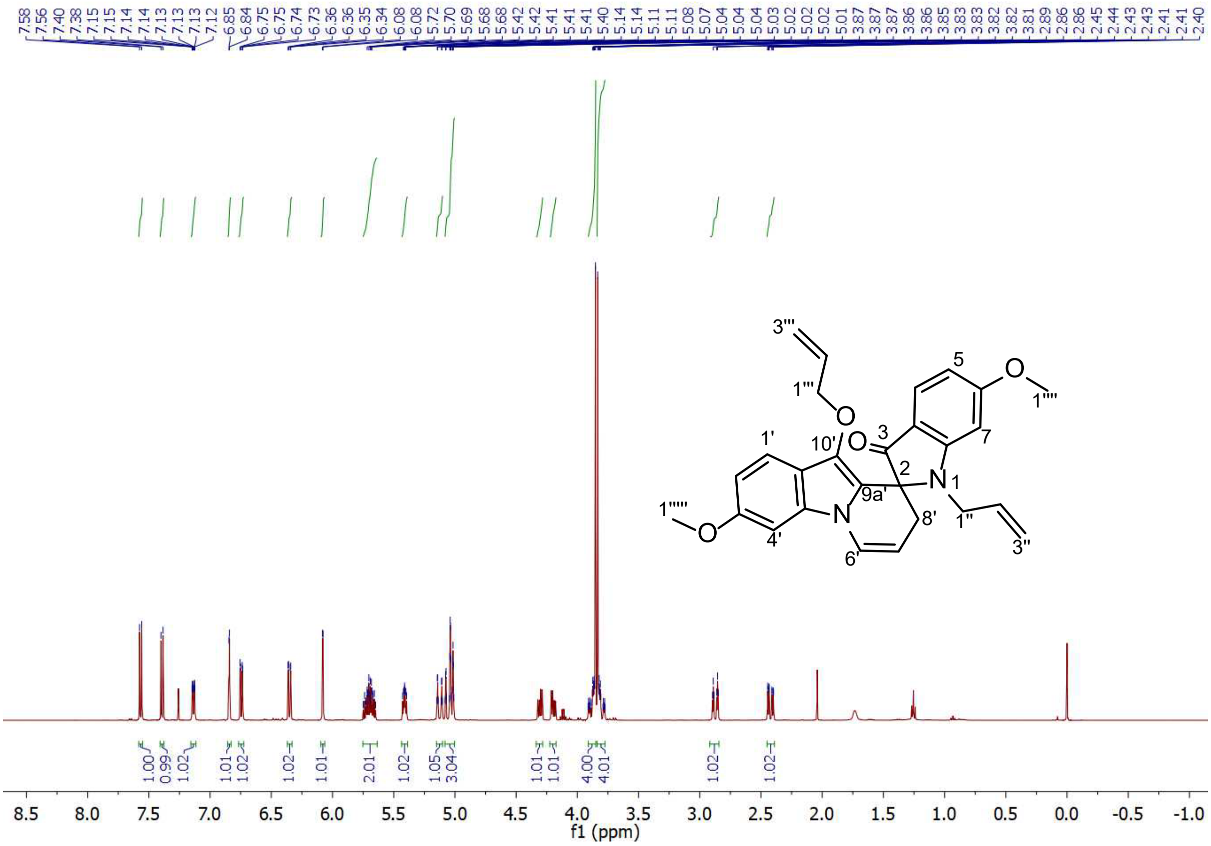

Figure S30: The ^{1}H NMR spectrum of 6,3'-dimethoxyspiroindolinepyridoindolone **19** (500 MHz, $CDCl_3$).

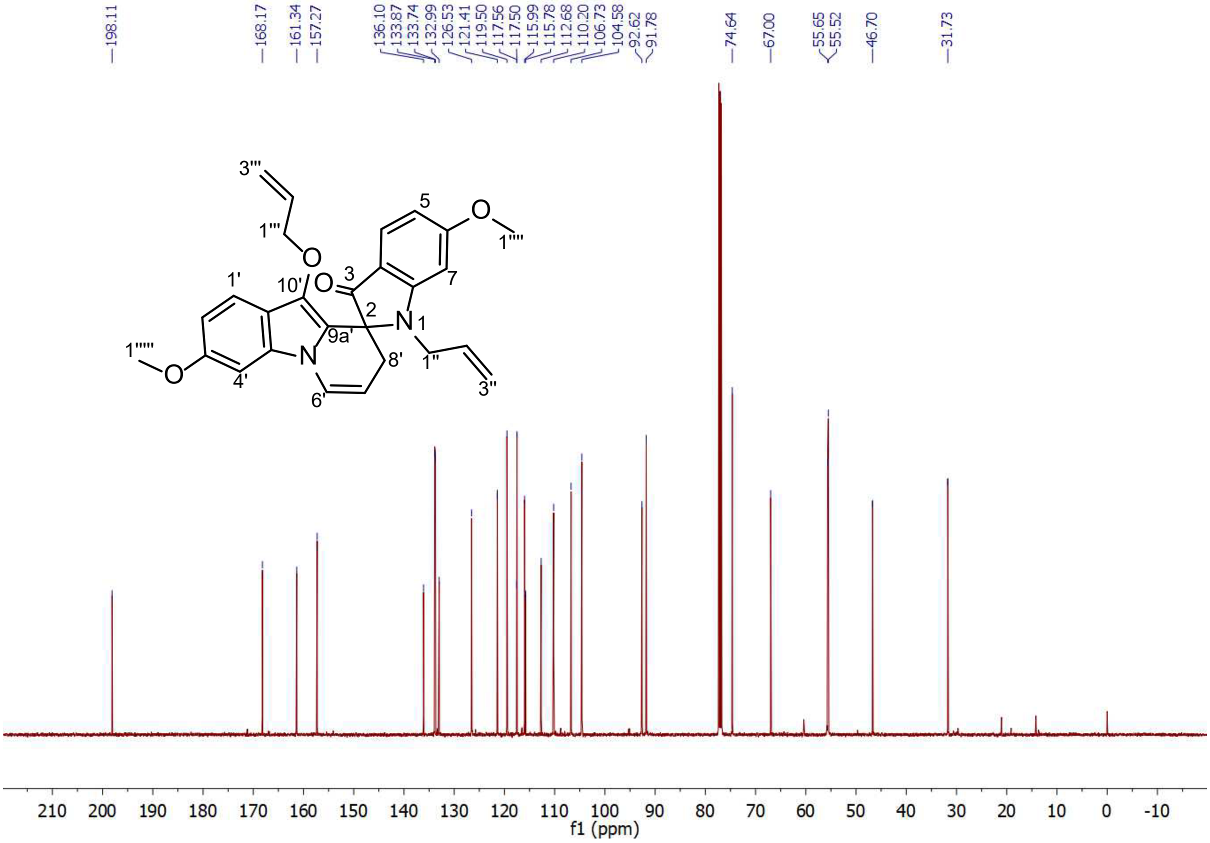

Figure S31: The ^{13}C NMR spectrum of 6,3'-dimethoxyspiroindolinepyridoindolone **19** (126 MHz, $CDCl_3$).

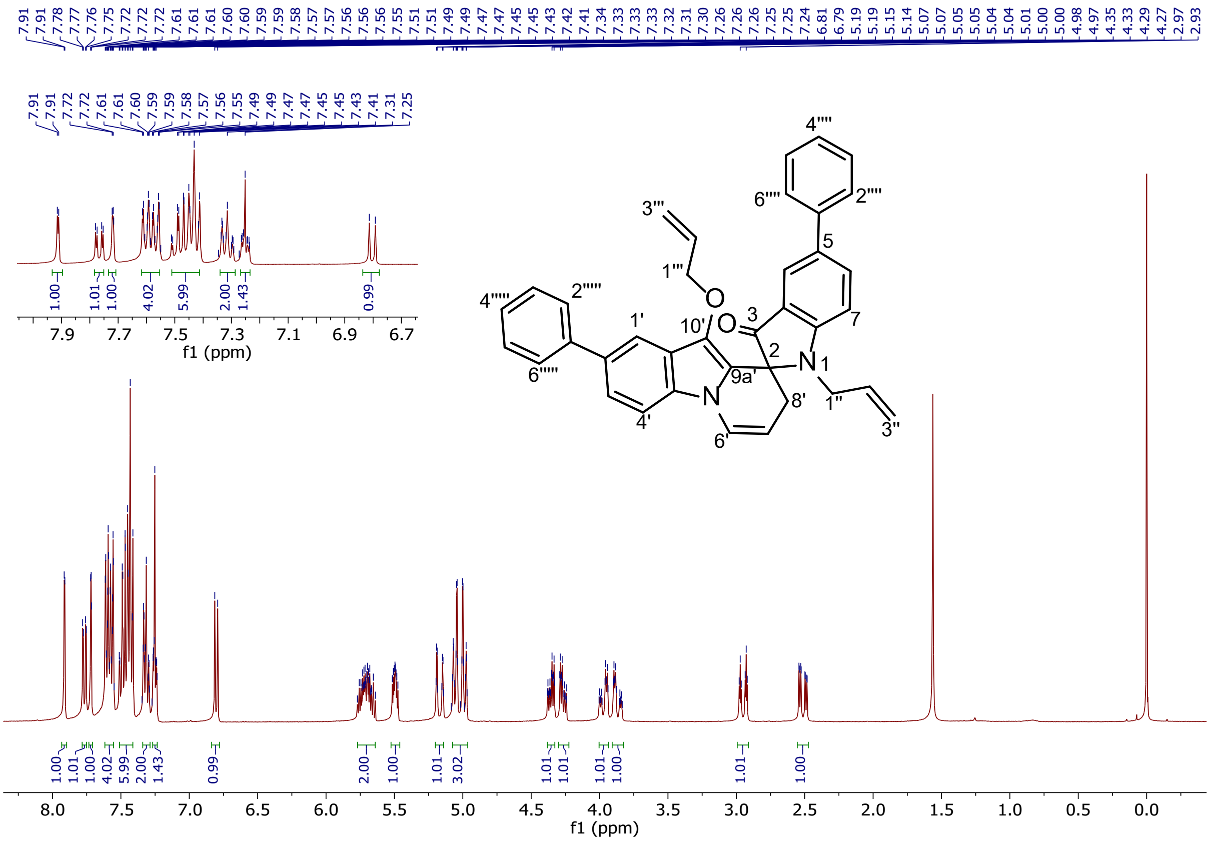

Figure S32: The ^{1}H NMR spectrum of 5,2'-diphenylspiroindolinepyridoindolone **20** (400 MHz, $CDCl_3$).

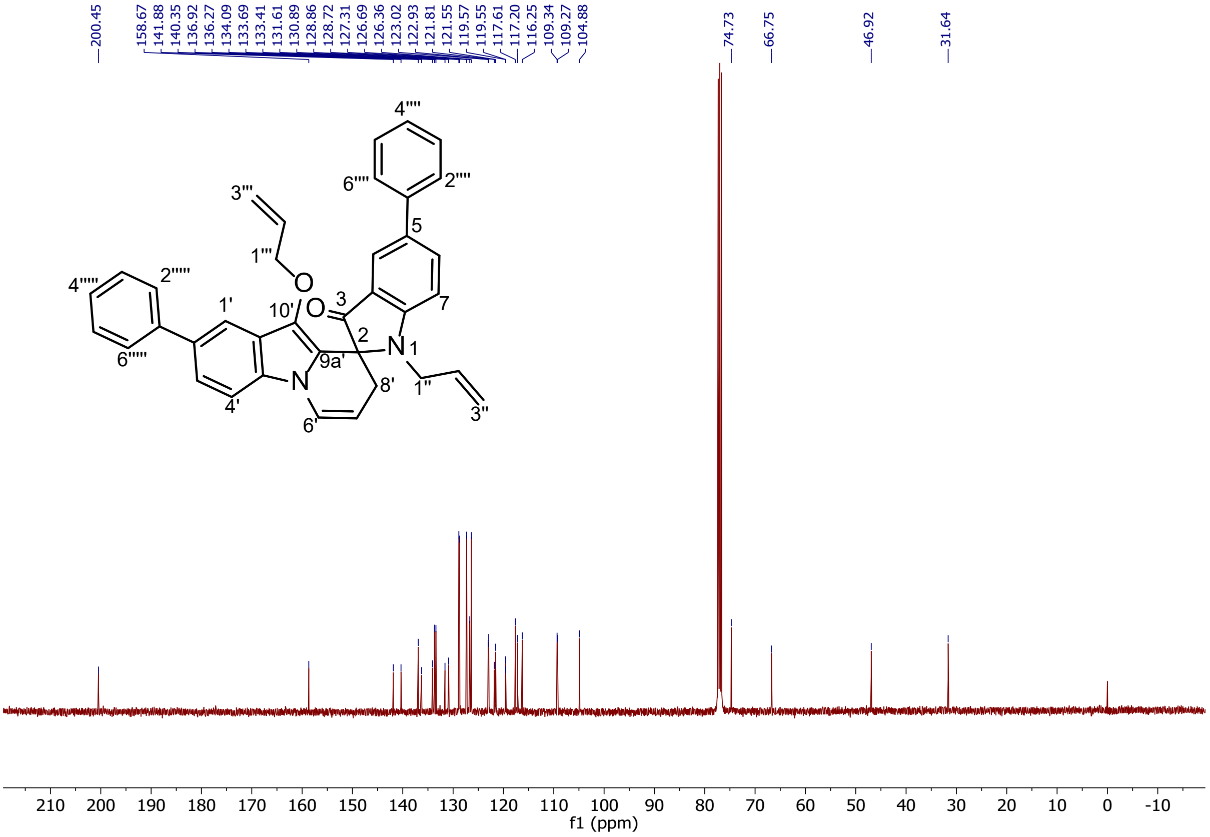

Figure S33: The ^{13}C NMR spectrum of 5,2'-diphenylspiroindolinepyridoindolone **20** (101 MHz, $CDCl_3$).

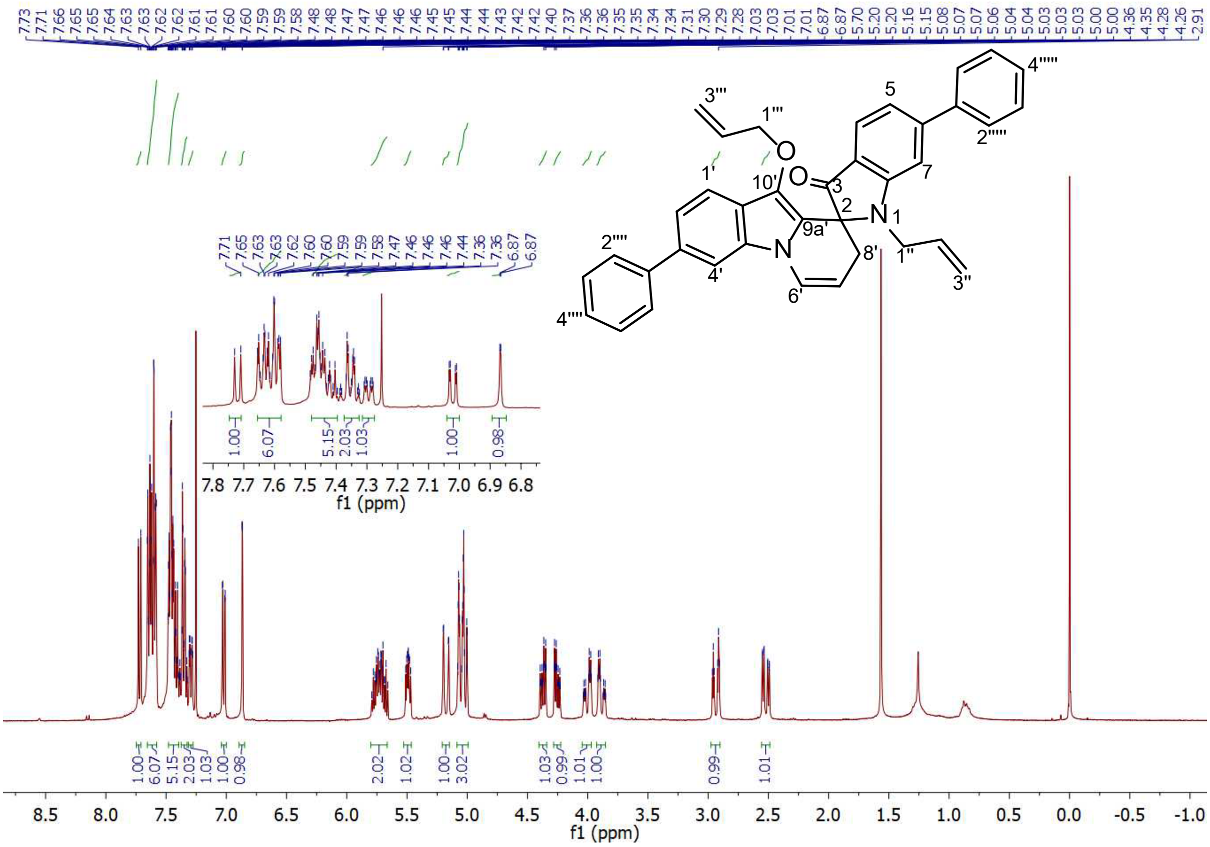

Figure S34: The ^{1}H NMR spectrum of 6,3'-diphenylspiroindolinepyridoindolone **21** (400 MHz, $CDCl_3$).

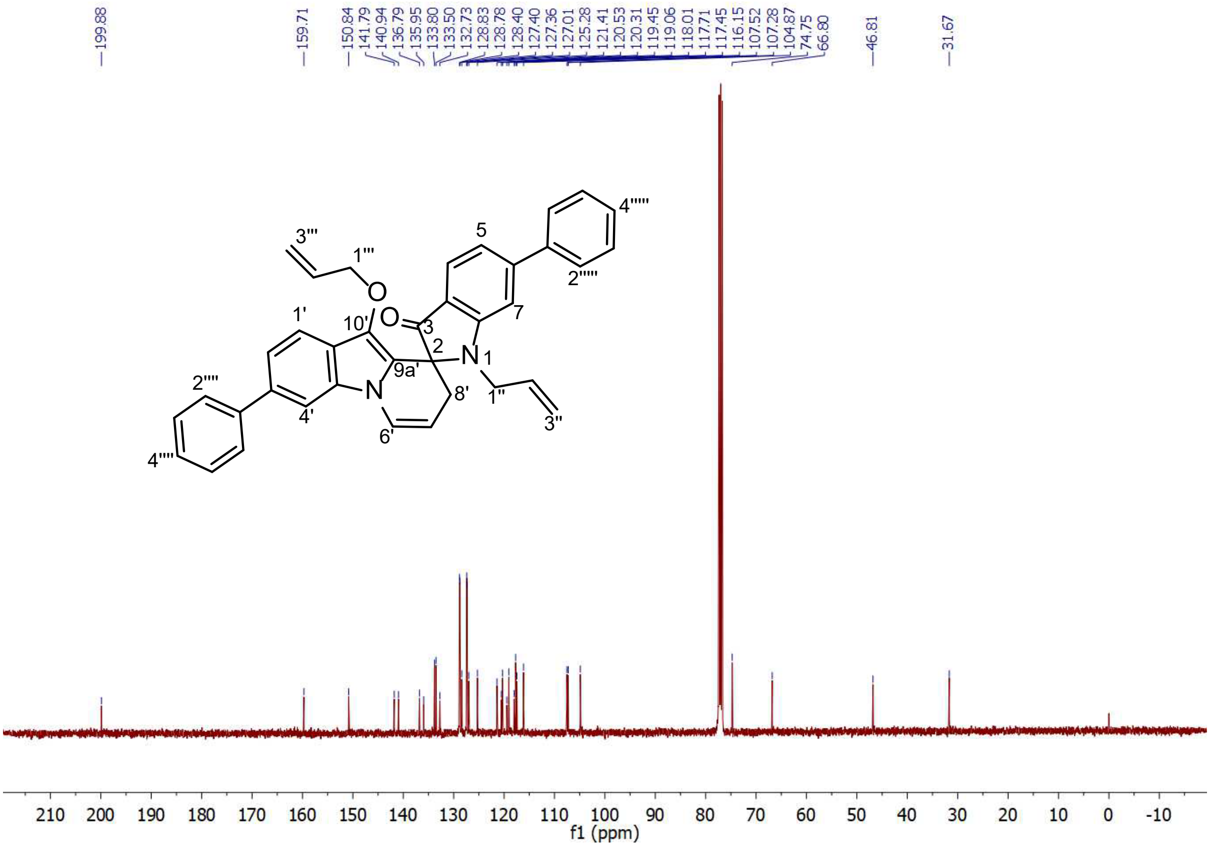

Figure S35: The ^{13}C NMR spectrum of 6,3'-diphenylspiroindolinepyridoindolone **21** (101 MHz, $CDCl_3$).

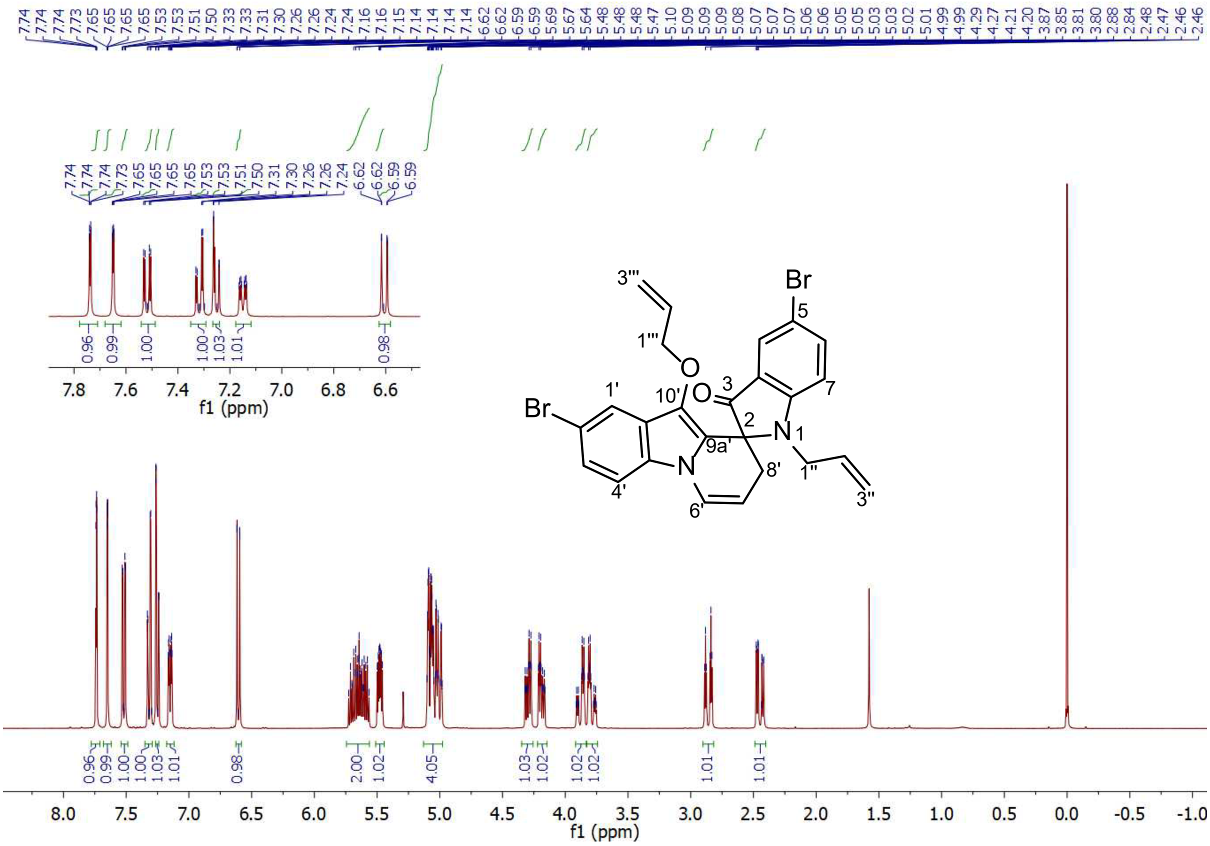

Figure S36: The ^{1}H NMR spectrum of 5,2'-dibromospiroindolinepyridoindolone **22** (400 MHz, $CDCl_3$).

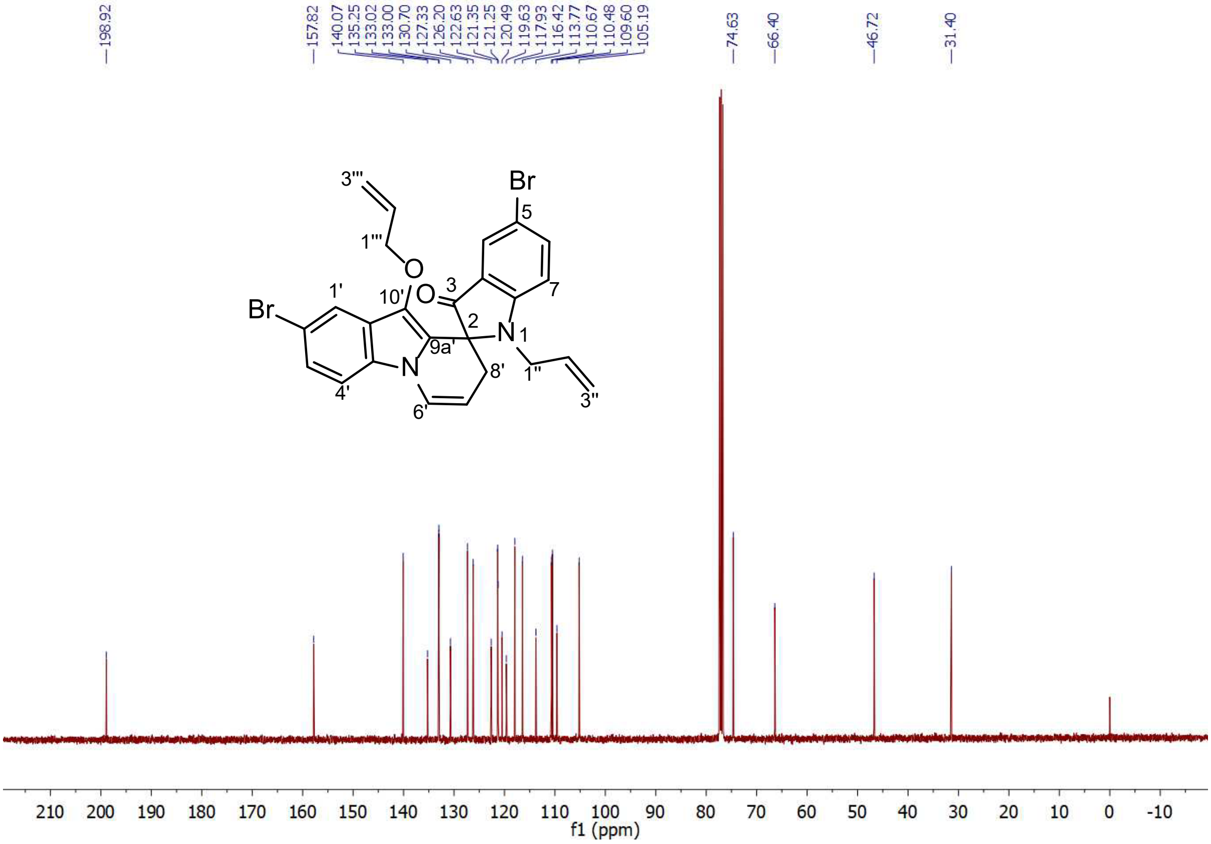

Figure S37: The ^{13}C NMR spectrum of 5,2'-dibromospiroindolinepyridoindolone **22** (101 MHz, $CDCl_3$).

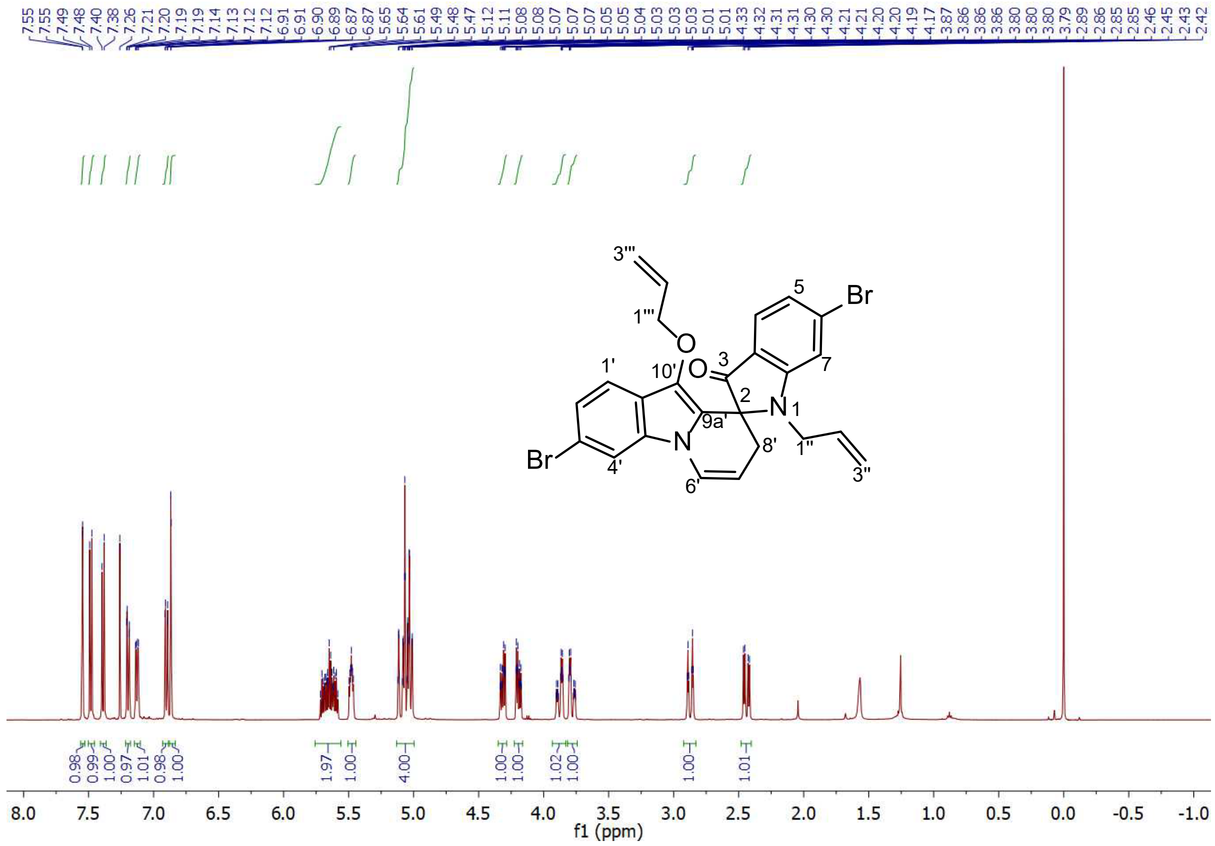

Figure S38: The ^{1}H NMR spectrum of 6,3'-dibromospiroindolinepyridoindolone **23** (500 MHz, $CDCl_3$).

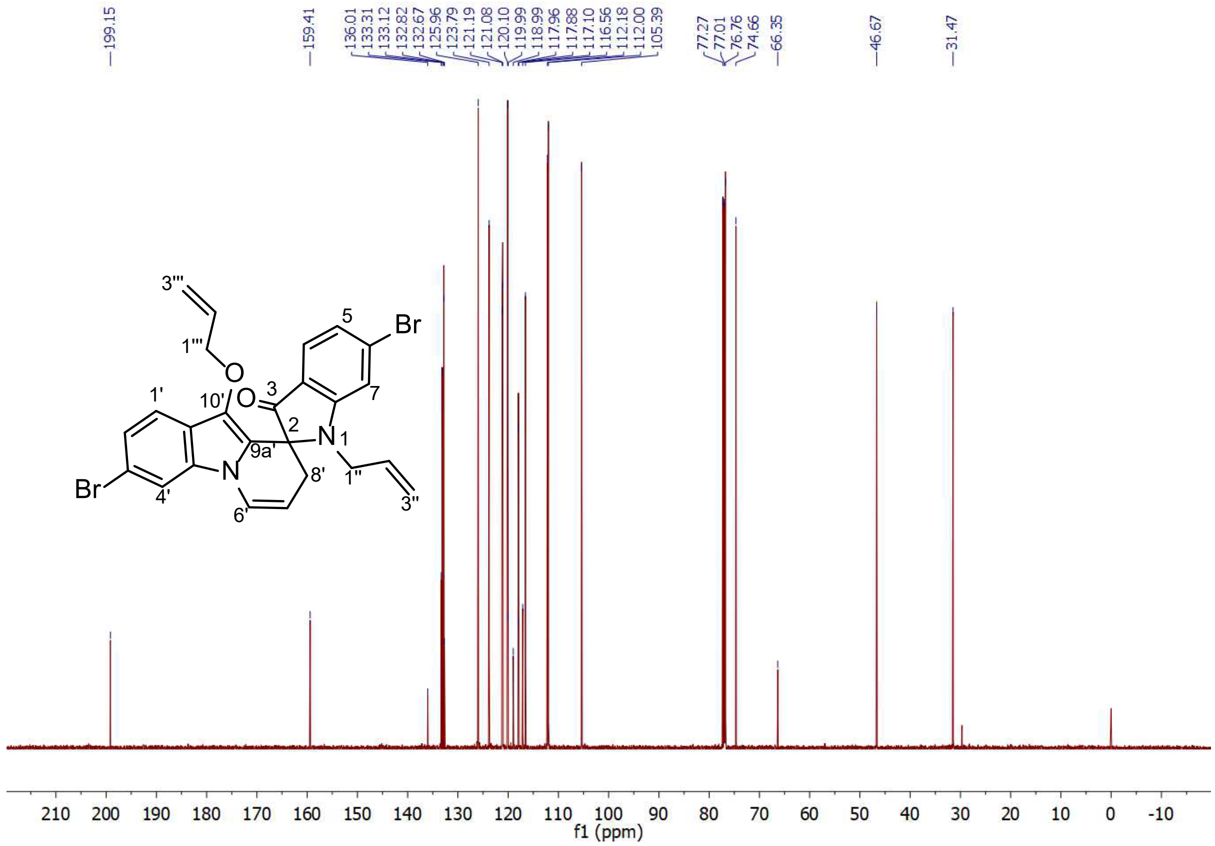

Figure S39: The ^{13}C NMR spectrum of 6,3'-dibromospiroindolinepyridoindolone **23** (126 MHz, $CDCl_3$).

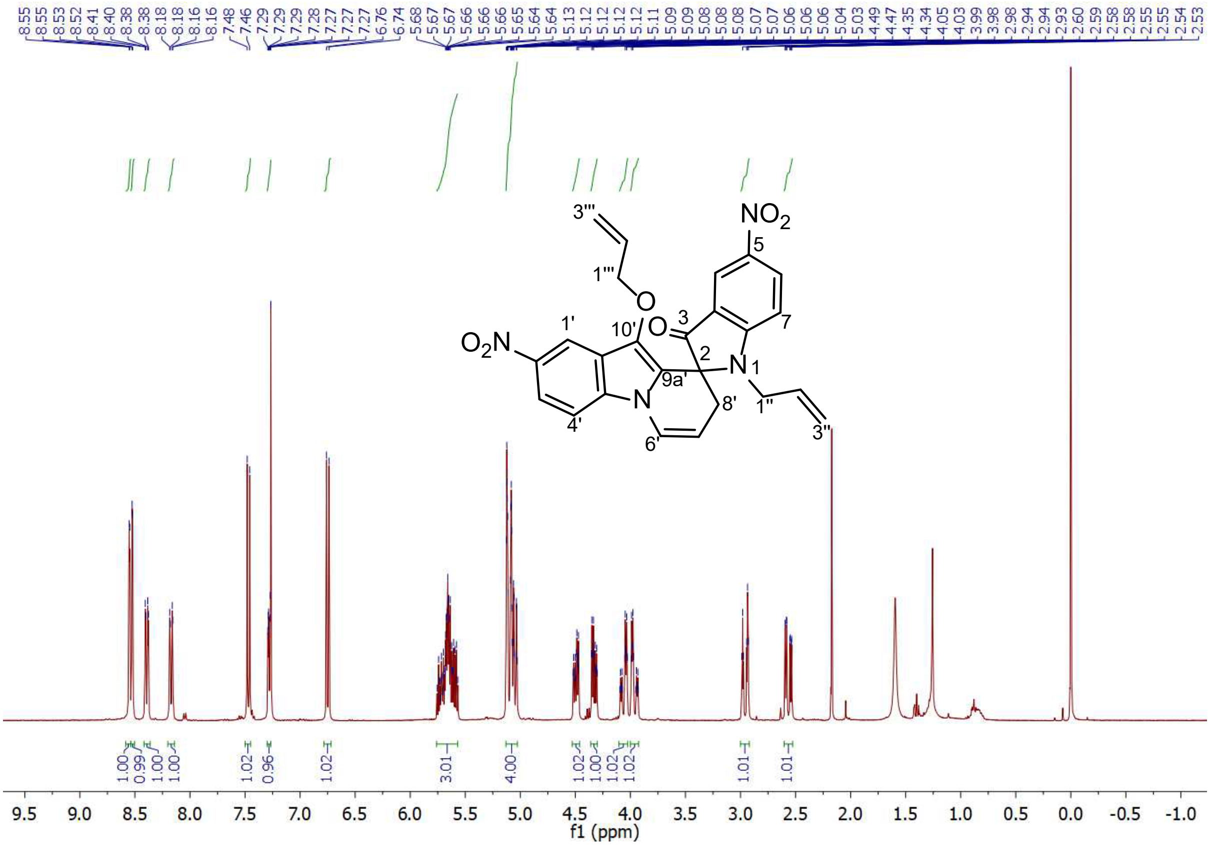

Figure S40: The ^{1}H NMR spectrum of 5,2'-dinitrospiroindolinepyridoindolone **24** (400 MHz, CDCl$_3$).

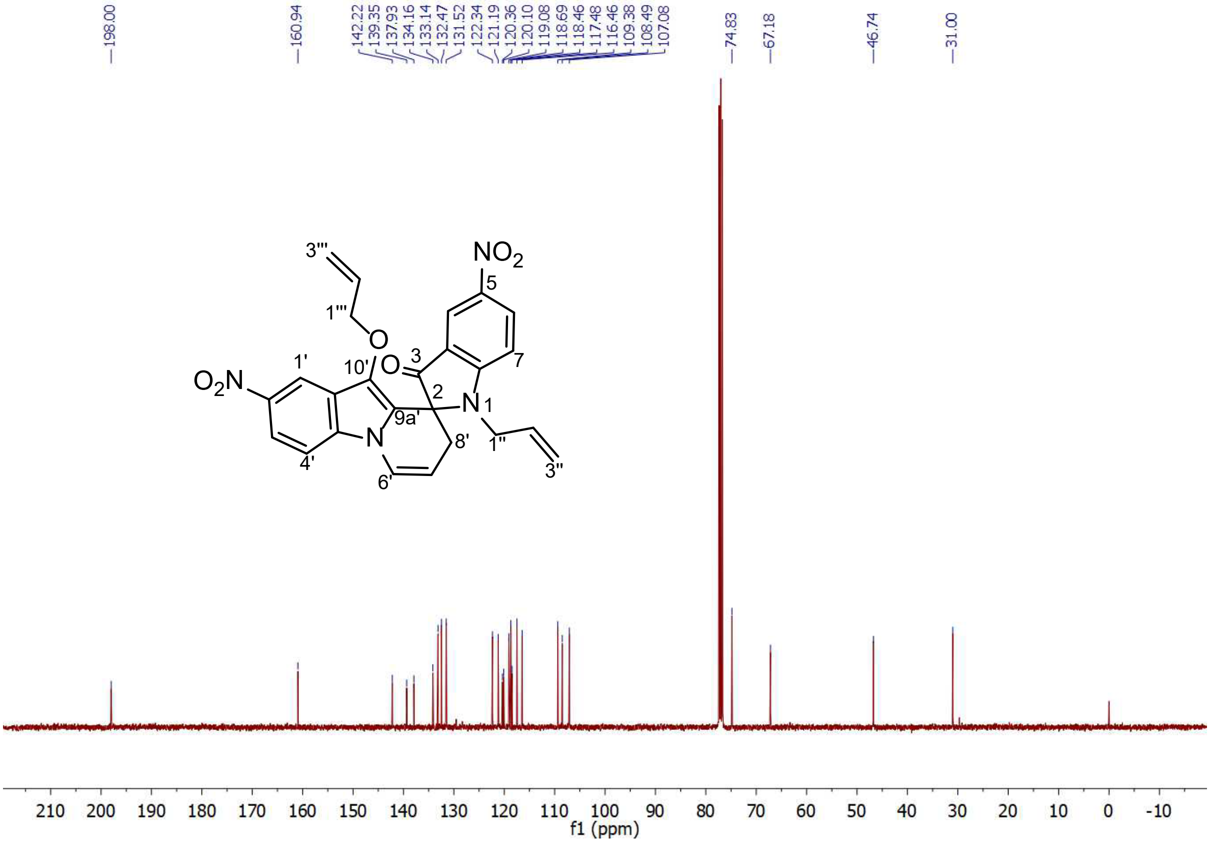

Figure S41: The ^{13}C NMR spectrum of 5,2'-dinitrospiroindolinepyridoindolone **24** (101 MHz, CDCl$_3$).

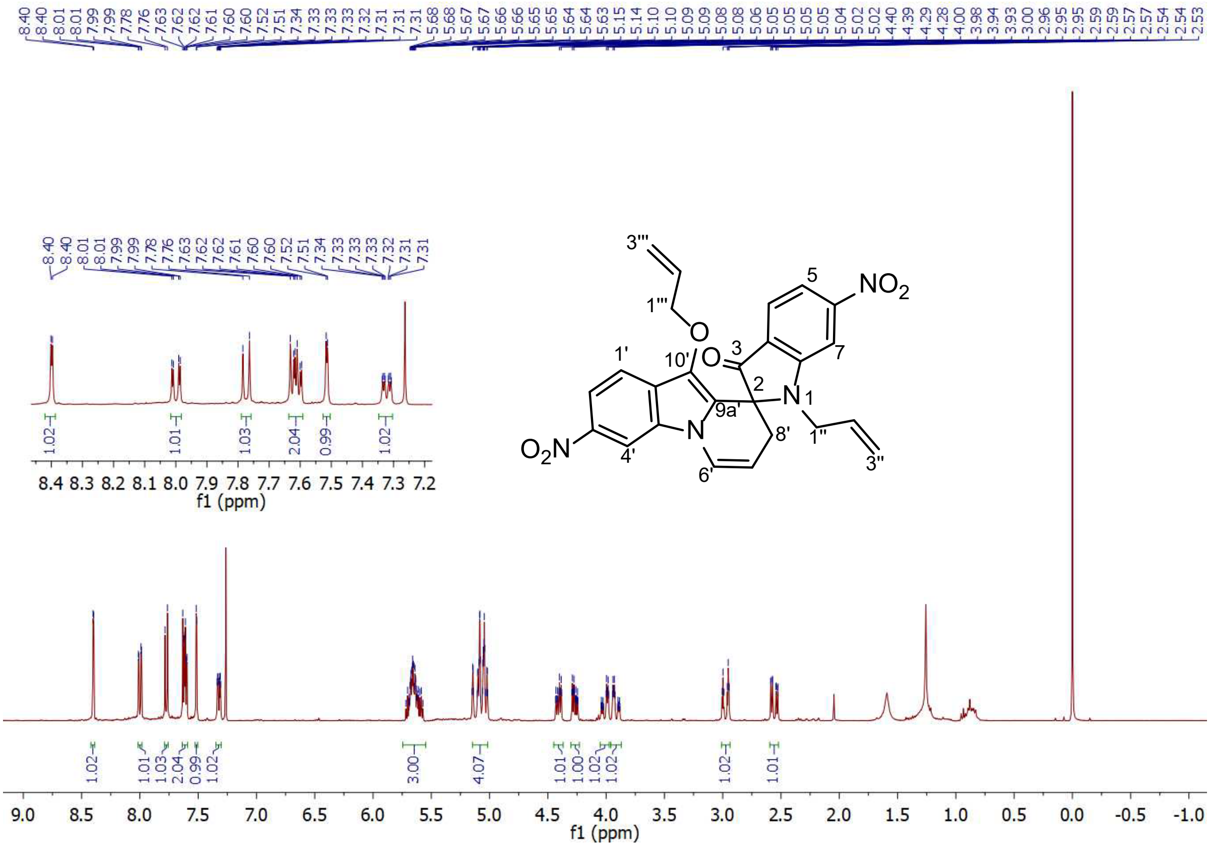

Figure S42: The ^{1}H NMR spectrum of 6,3'-dinitrospiroindolinepyridoindolone **25** (400 MHz, $CDCl_3$).

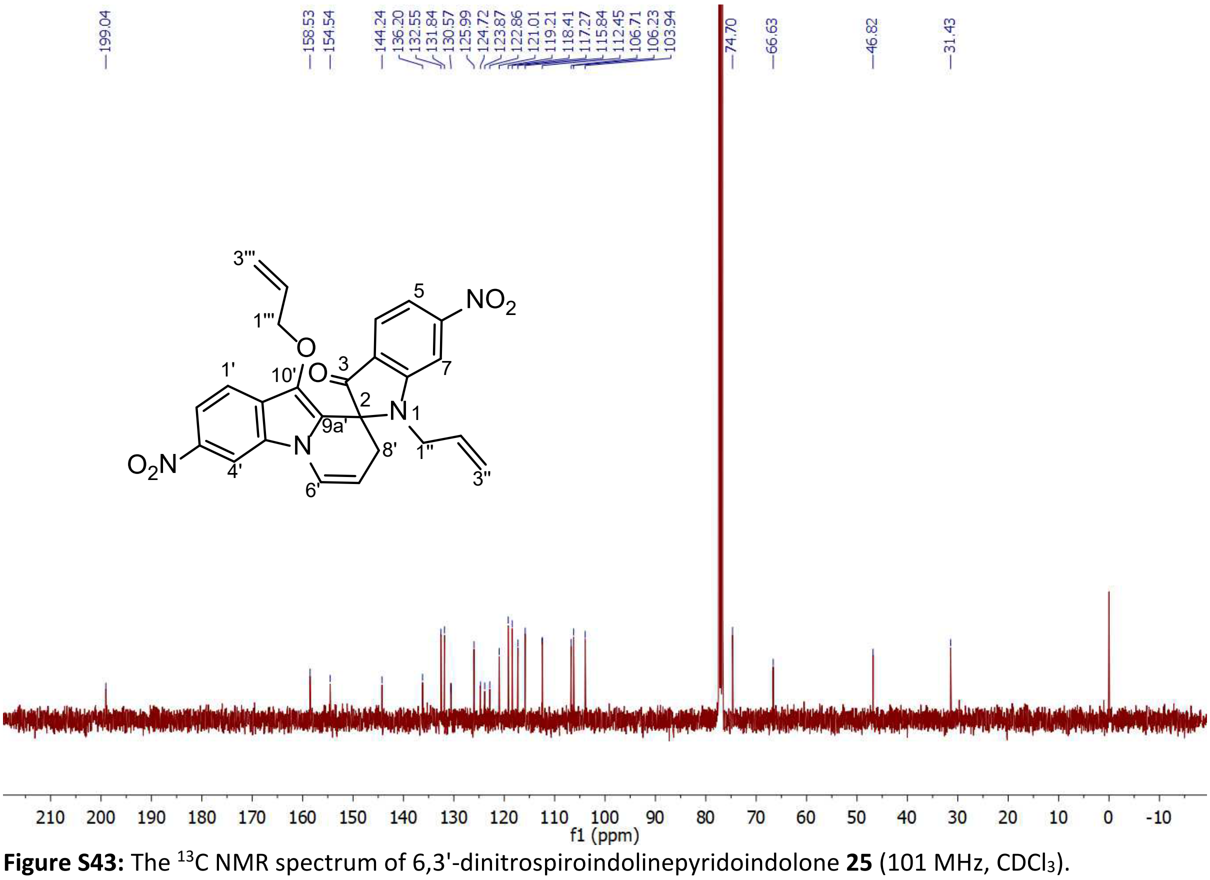

Figure S43: The ^{13}C NMR spectrum of 6,3'-dinitrospiroindolinepyridoindolone **25** (101 MHz, $CDCl_3$).

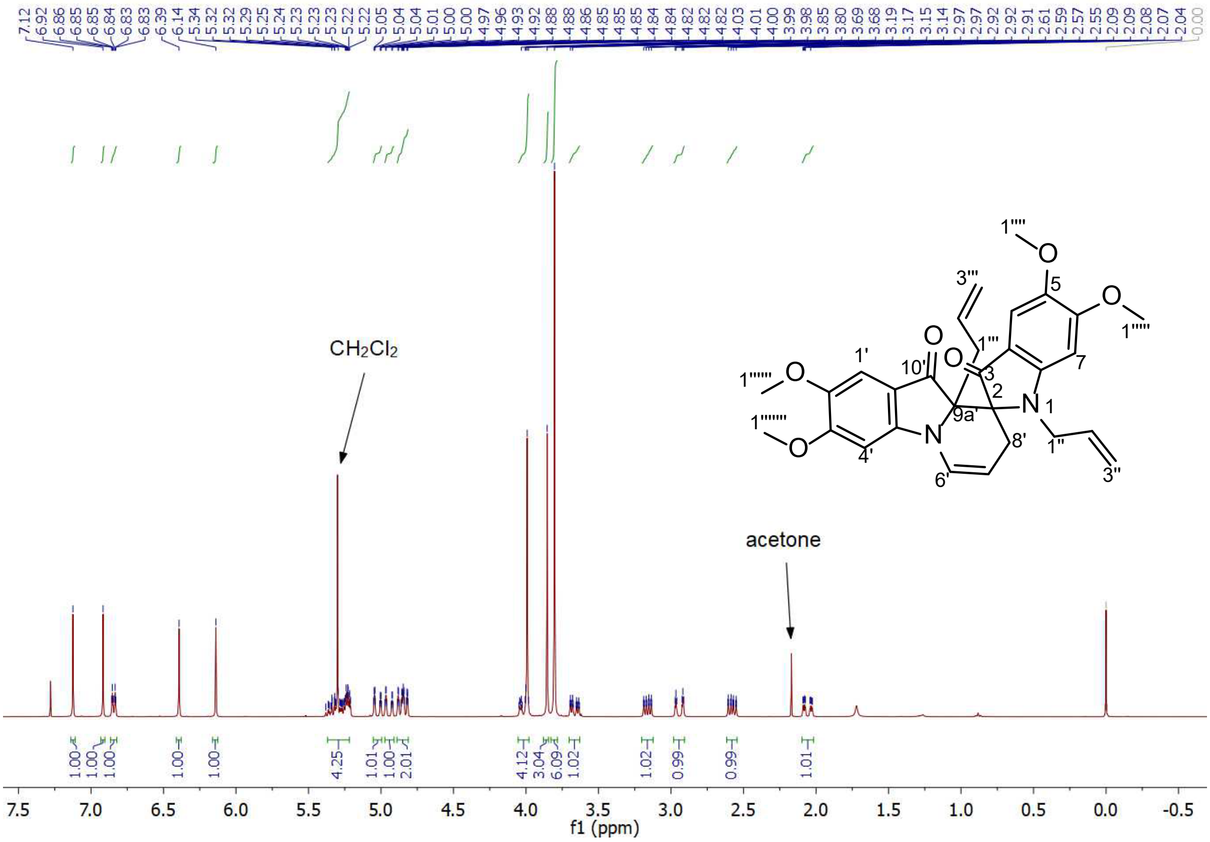

Figure S44: The ^{1}H NMR spectrum of 5,6,2',3'-tetramethoxyspiroindolinepyridoindoledione **26** (400 MHz, $CDCl_3$).

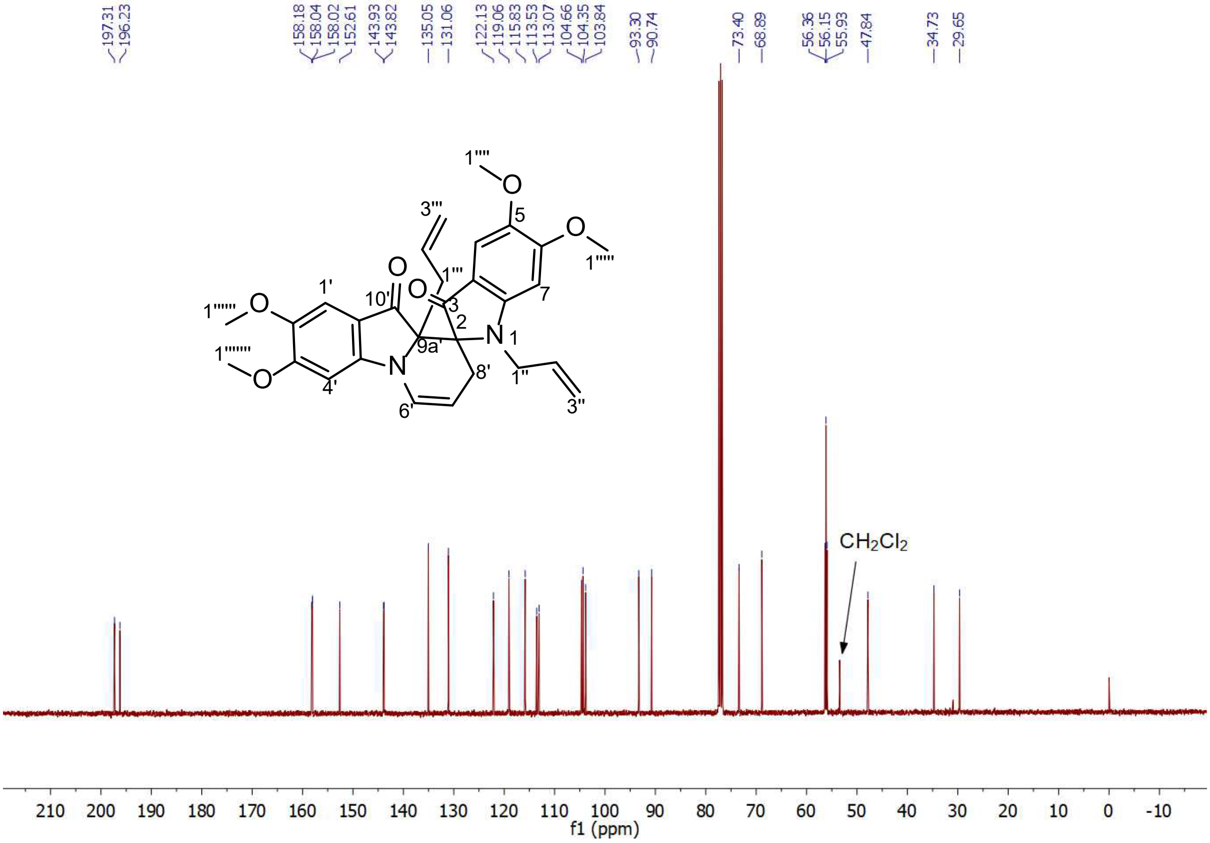

Figure S45: The ^{13}C NMR spectrum of 5,6,2',3'-tetramethoxyspiroindolinepyridoindoledione **26** (101 MHz, $CDCl_3$).

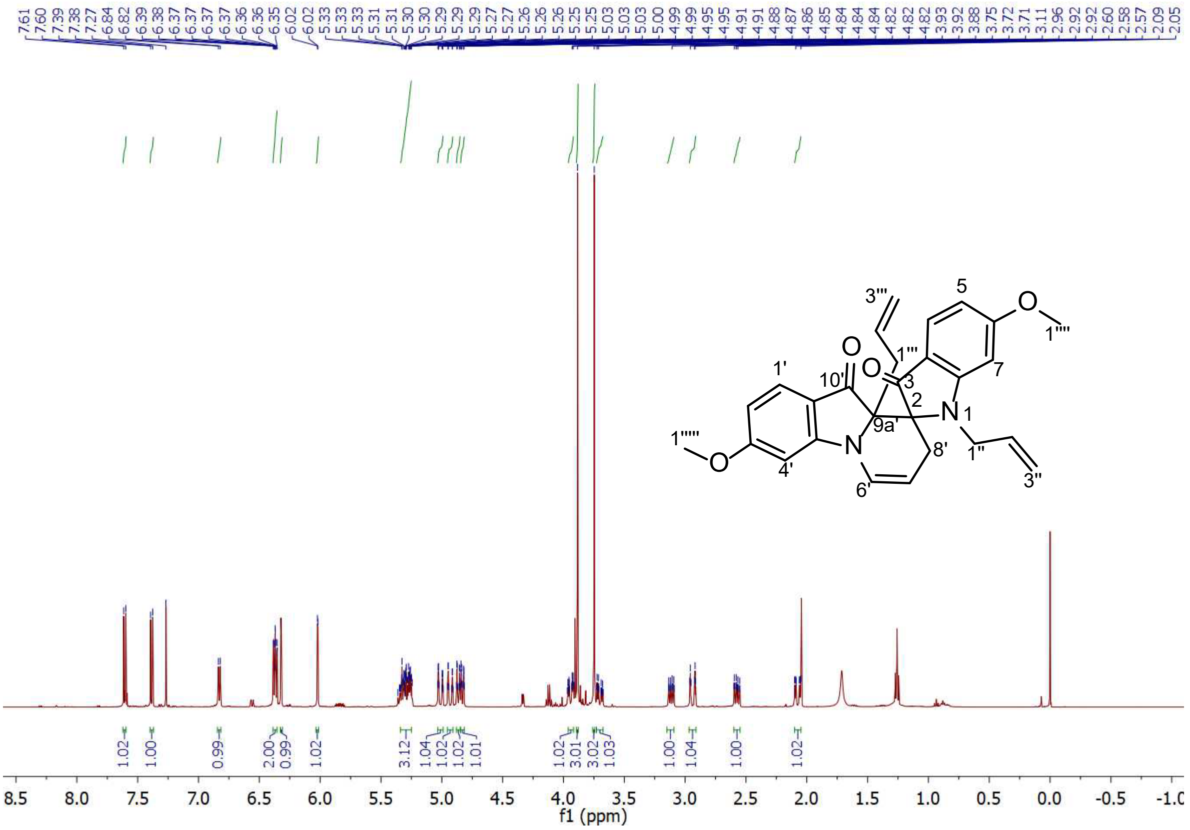

Figure S46: The ^{1}H NMR spectrum of 6,3'-dimethoxyspiroindolinepyridoindoledione **27** (500 MHz, $CDCl_3$). 'Impurity' present is likely the diastereomer.

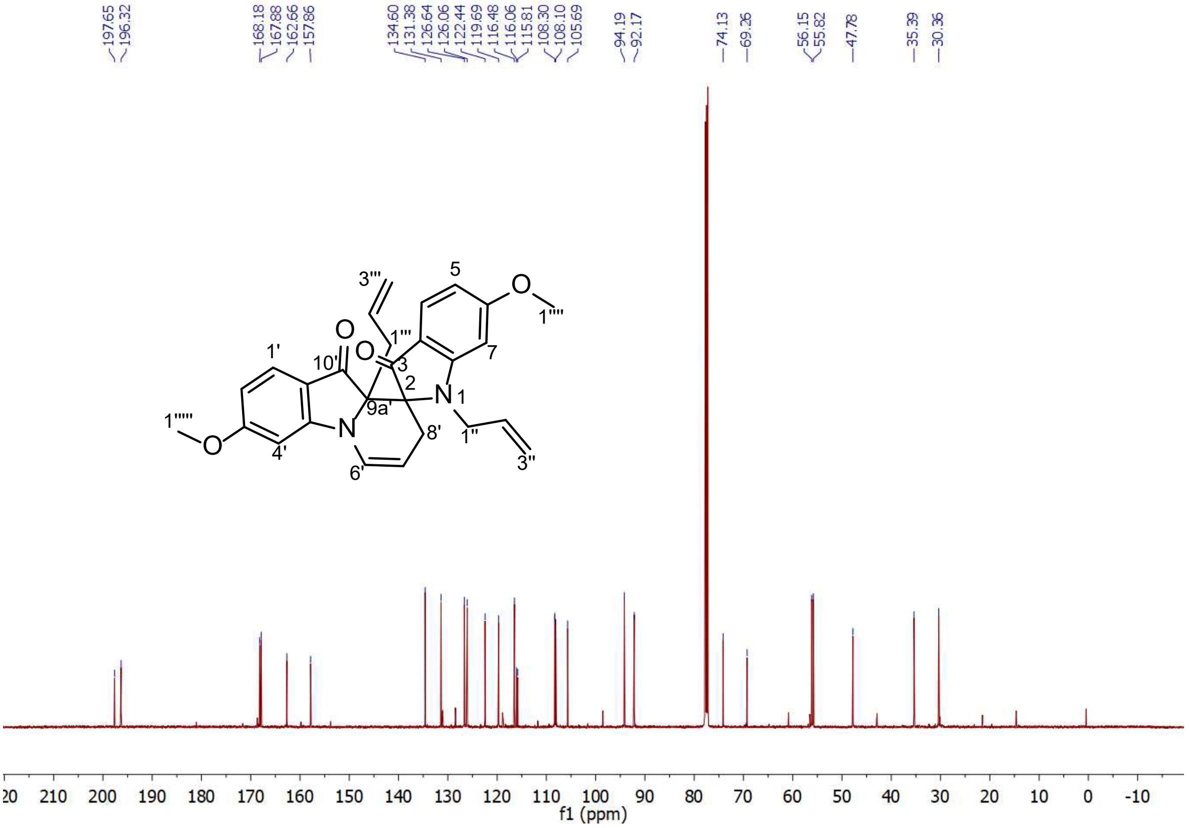

Figure S47: The ^{13}C NMR spectrum of 6,3'-dimethoxyspiroindolinepyridoindoledione **27** (126 MHz, $CDCl_3$). 'Impurity' present is likely the *transoid* diastereomer.

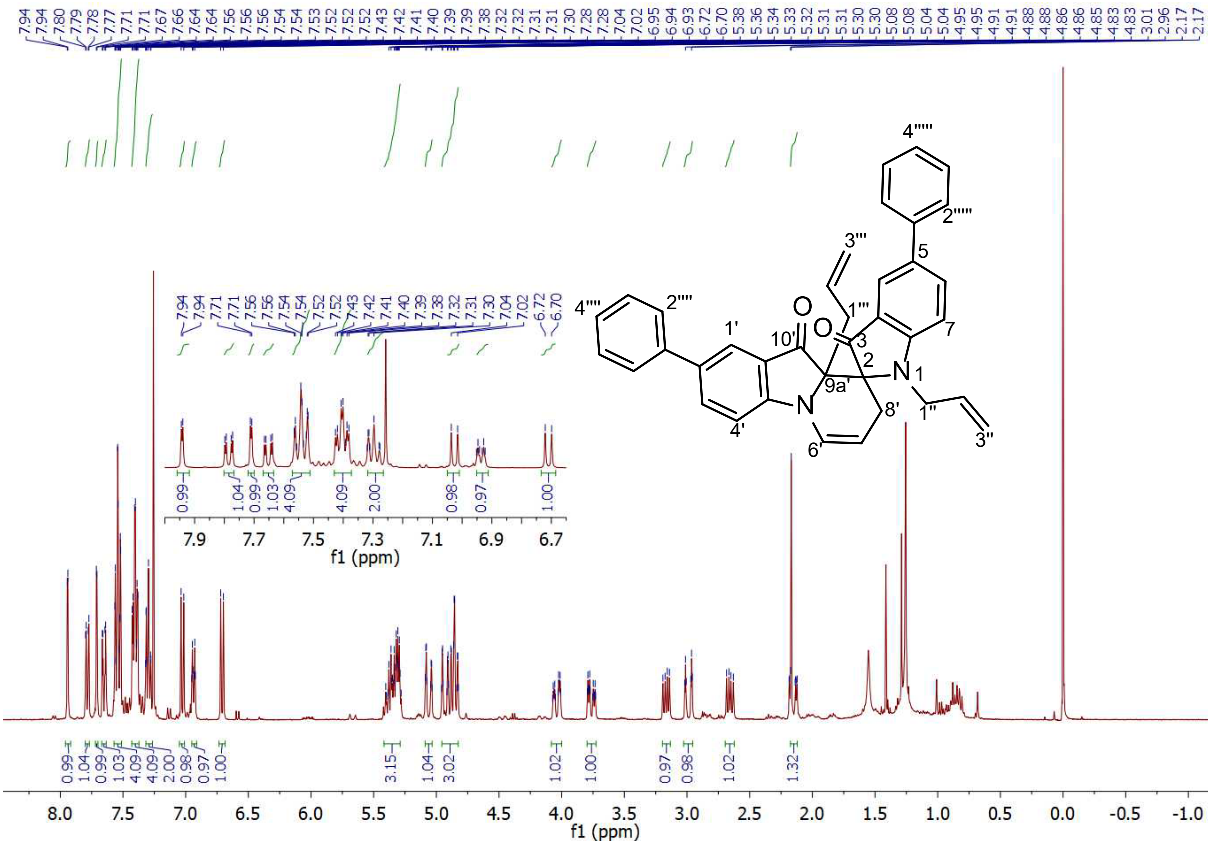

Figure S48: The ^{1}H NMR spectrum of 5,2'-diphenylspiroindolinepyridoindoledione **28** (400 MHz, $CDCl_3$) ‘Impurity’ present is likely the *transoid* diastereomer.

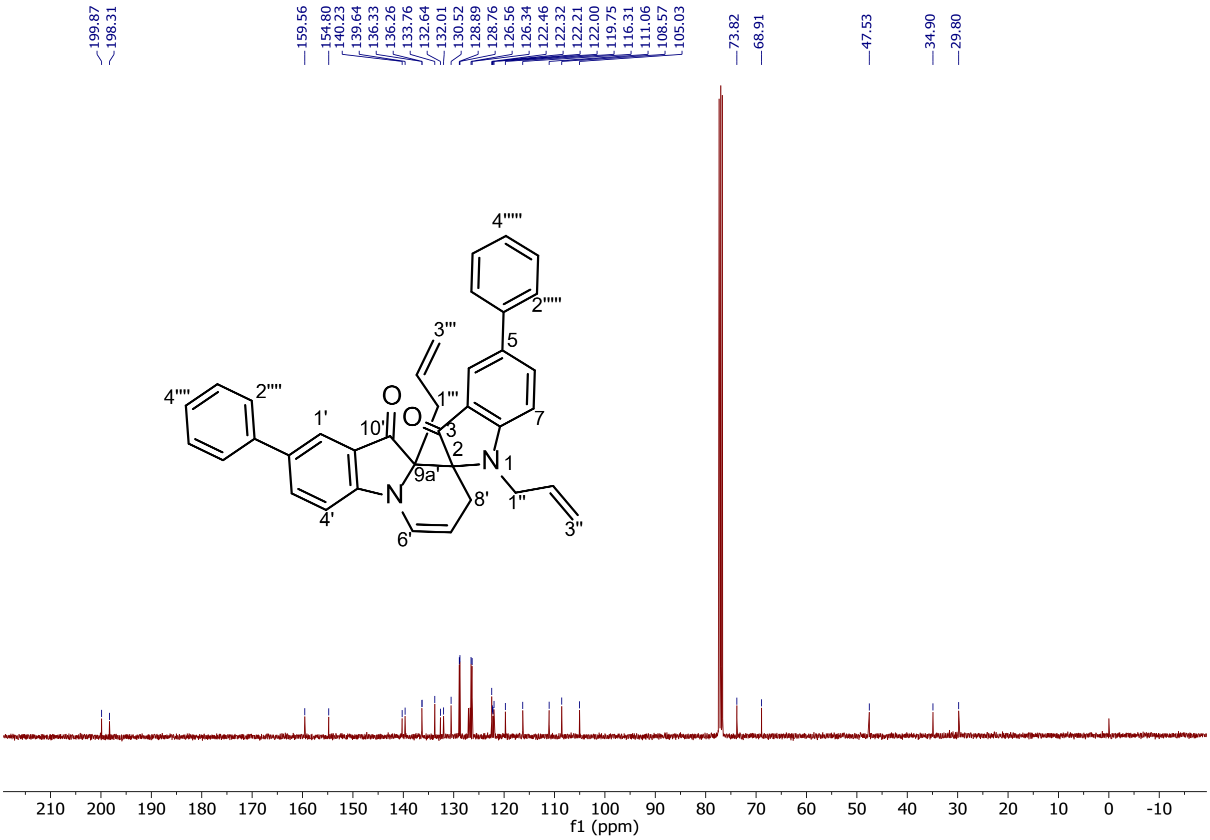

Figure S49: The ^{13}C NMR spectrum of 5,2'-diphenylspiroindolinepyridoindoledione **28** (101 MHz, $CDCl_3$). ‘impurity’ present is likely the *transoid* diastereomer.

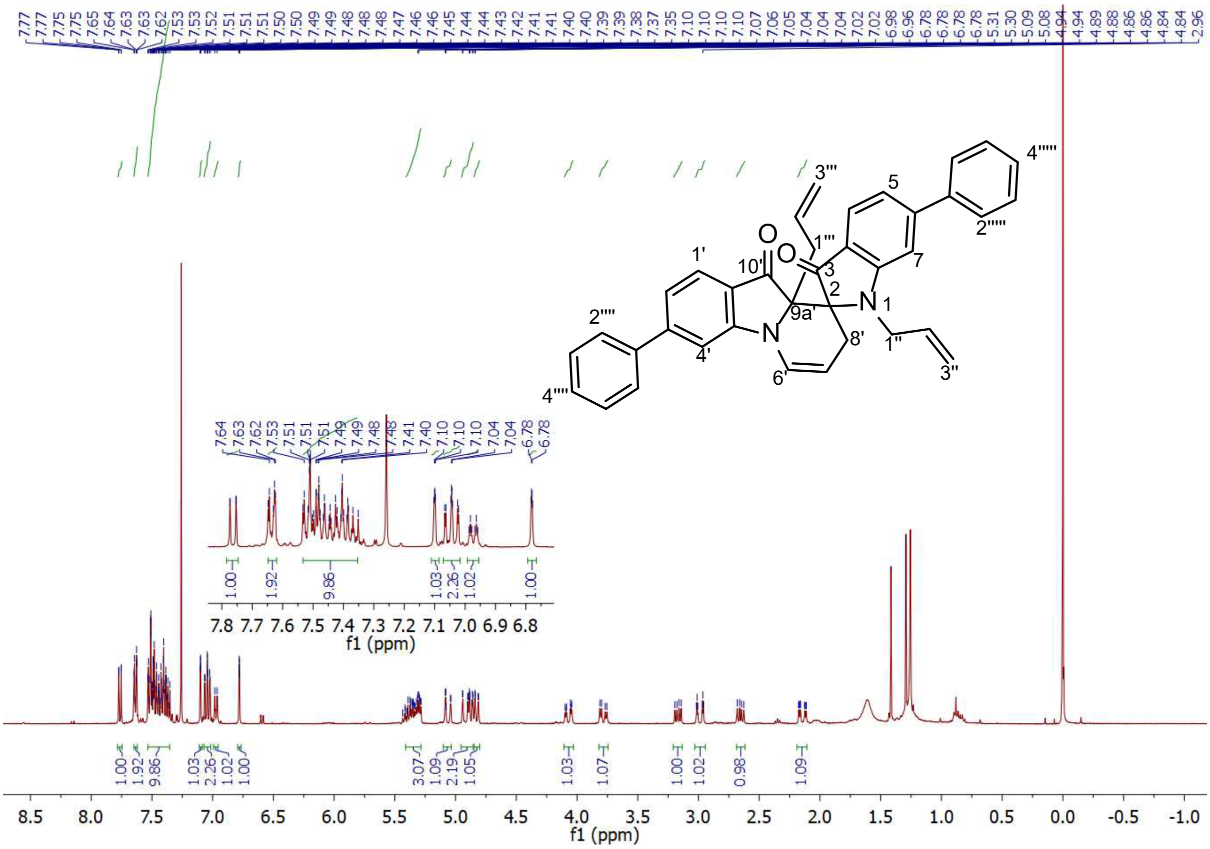

Figure S50: The ^{1}H NMR spectrum of 6,3'-diphenylspiroindolinepyridoindoledione **29** (400 MHz, $CDCl_3$). 'Impurity' present is likely the *transoid* diastereomer.

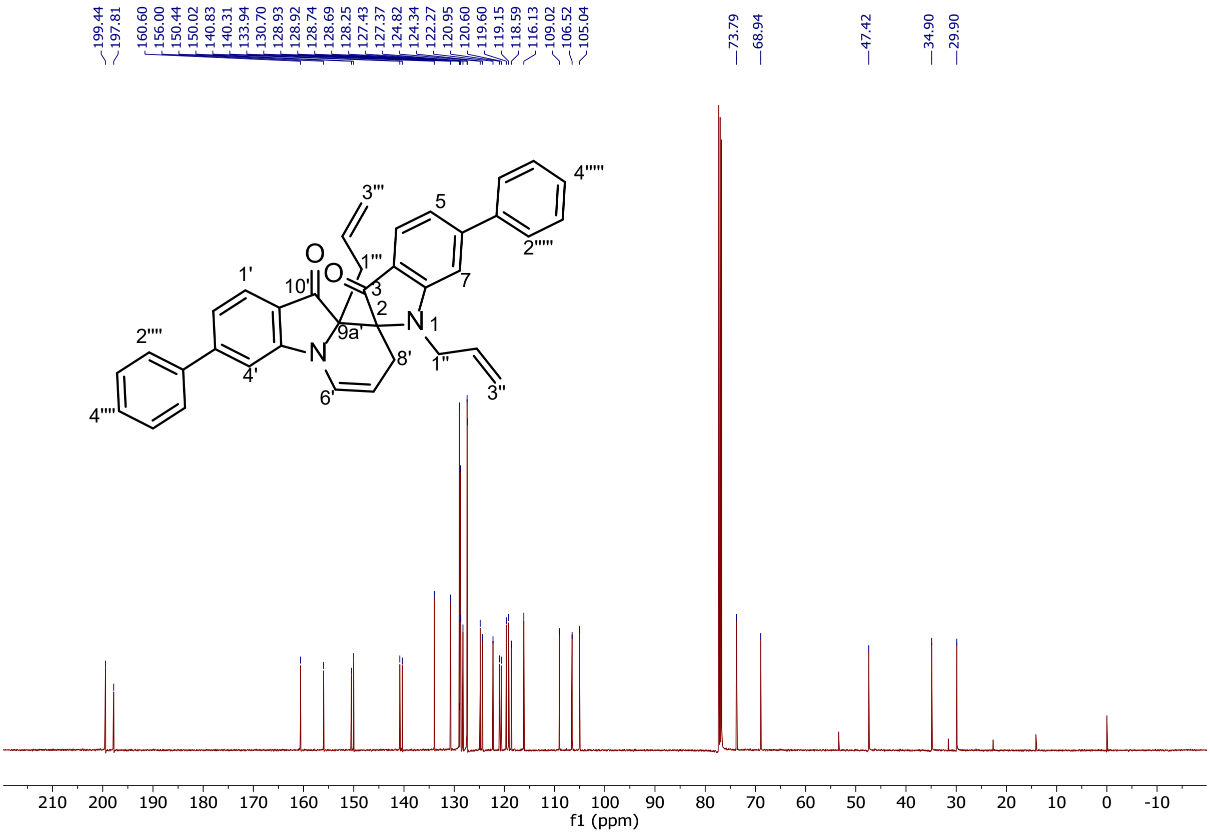

Figure S51: The ^{13}C NMR spectrum of 6,3'-diphenylspiroindolinepyridoindoledione **29** (126 MHz, $CDCl_3$). 'Impurity' present is likely the *transoid* diastereomer.

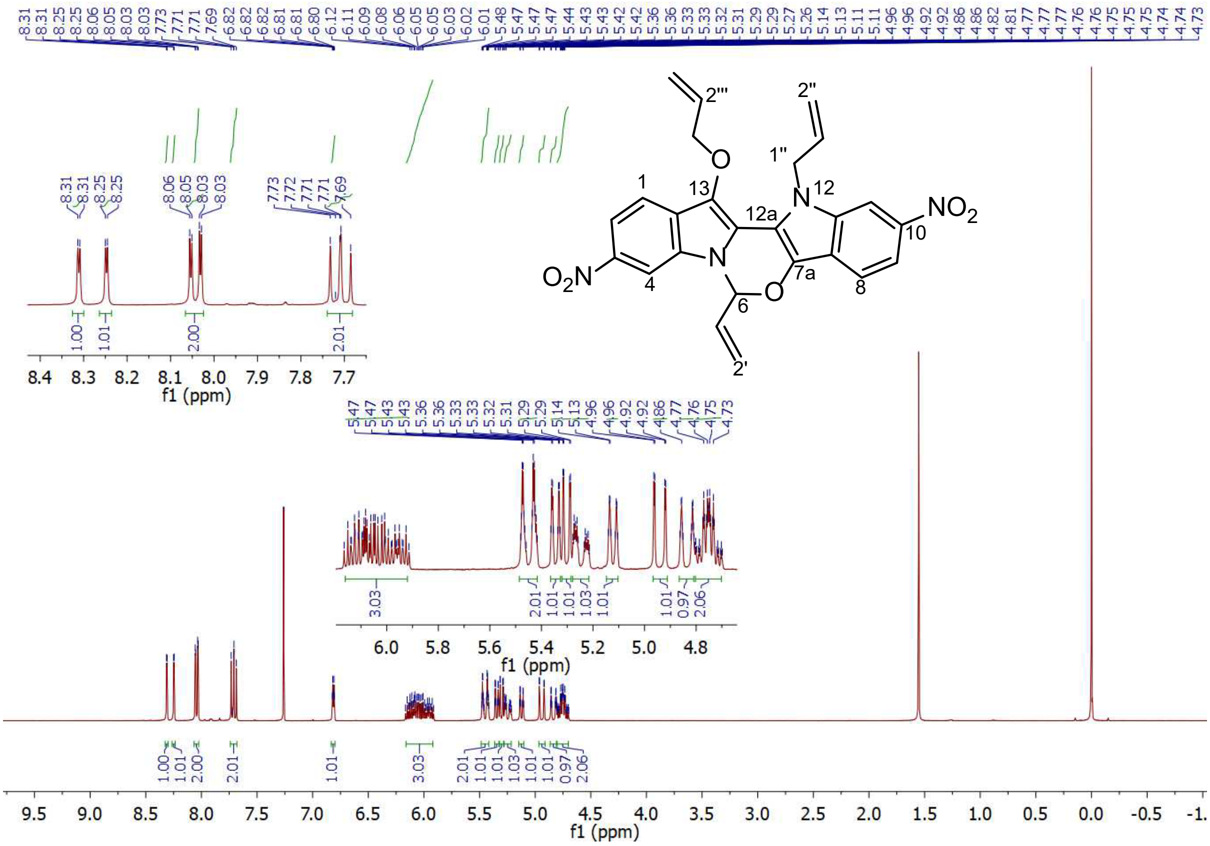

Figure S52: The ^{1}H NMR spectrum of 3,10-dinitrooxazinodiindole **30** (400 MHz, $CDCl_3$).

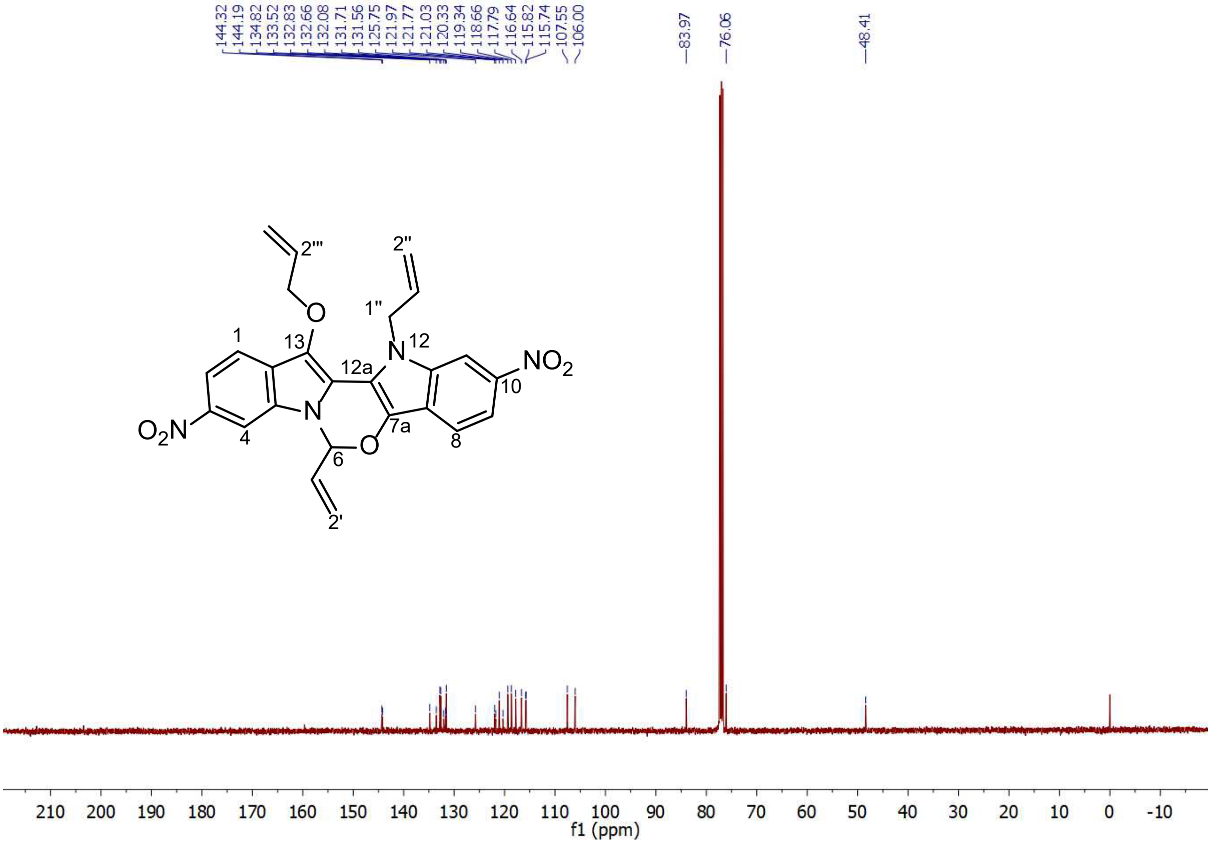

Figure S53: The ^{13}C NMR spectrum of 3,10-dinitrooxazinodiindole **30** (101 MHz, $CDCl_3$).

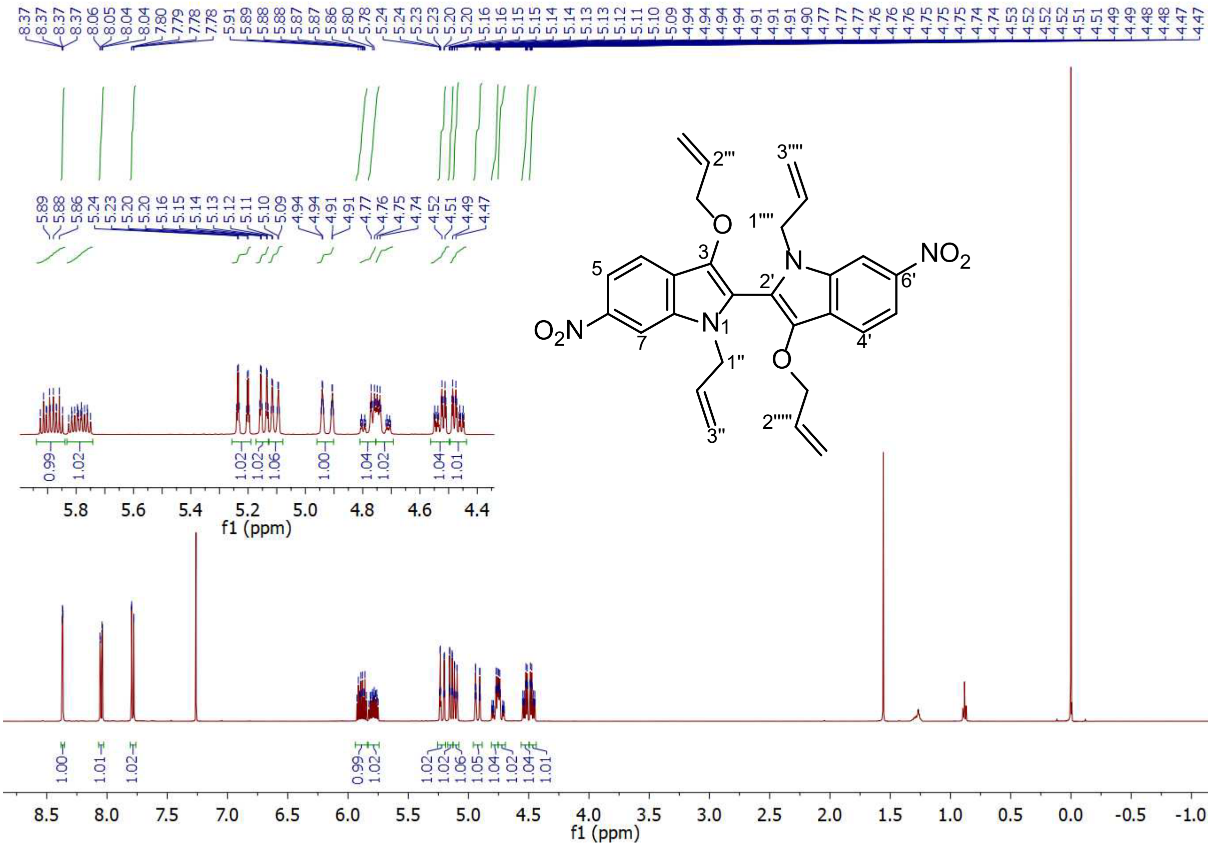

Figure S54: The ^{1}H NMR spectrum of *N*,*N′*-diallyl-3,3'-bis(allyloxy)-6,6'-dinitrobiindole **31** (500 MHz, $CDCl_3$).

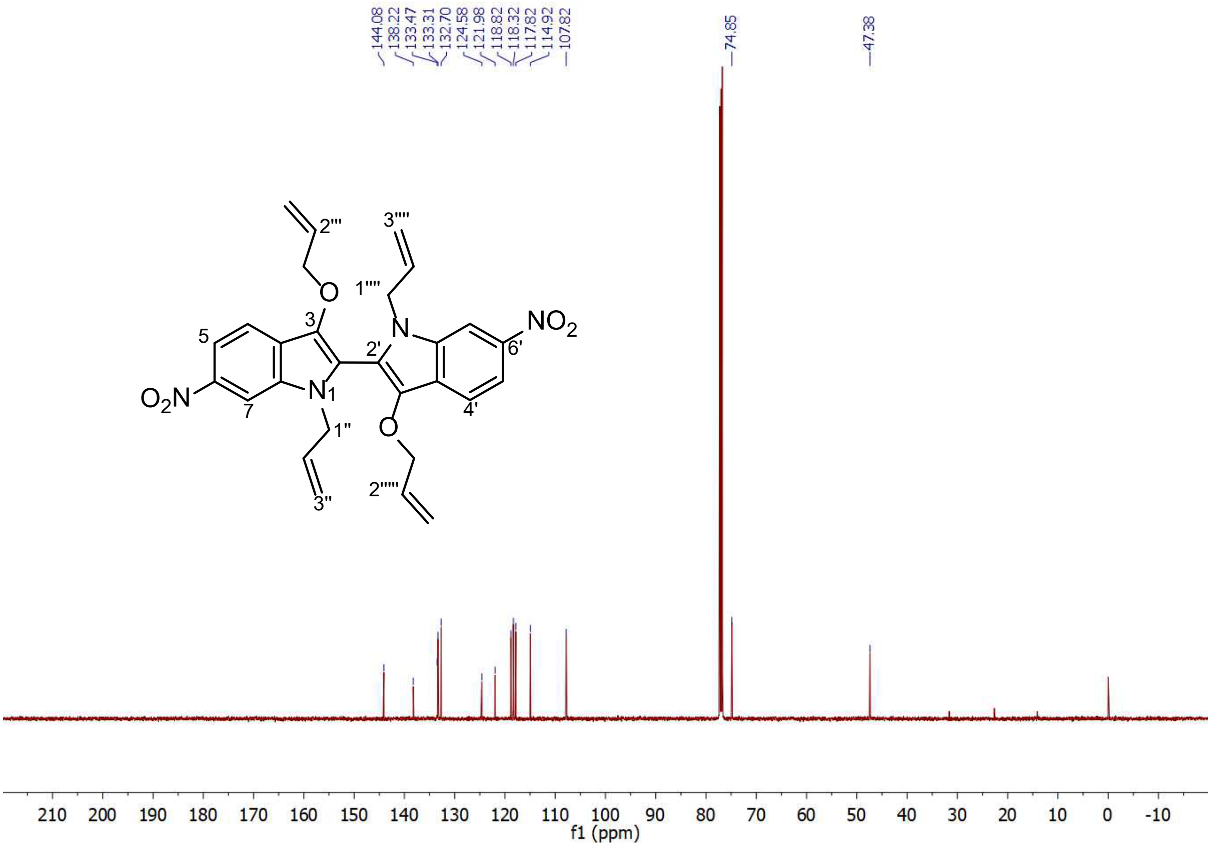

Figure S55: The ^{13}C NMR spectrum of *N*,*N′*-diallyl-3,3'-bis(allyloxy)-6,6'-dinitrobiindole **31** (126 MHz, $CDCl_3$).

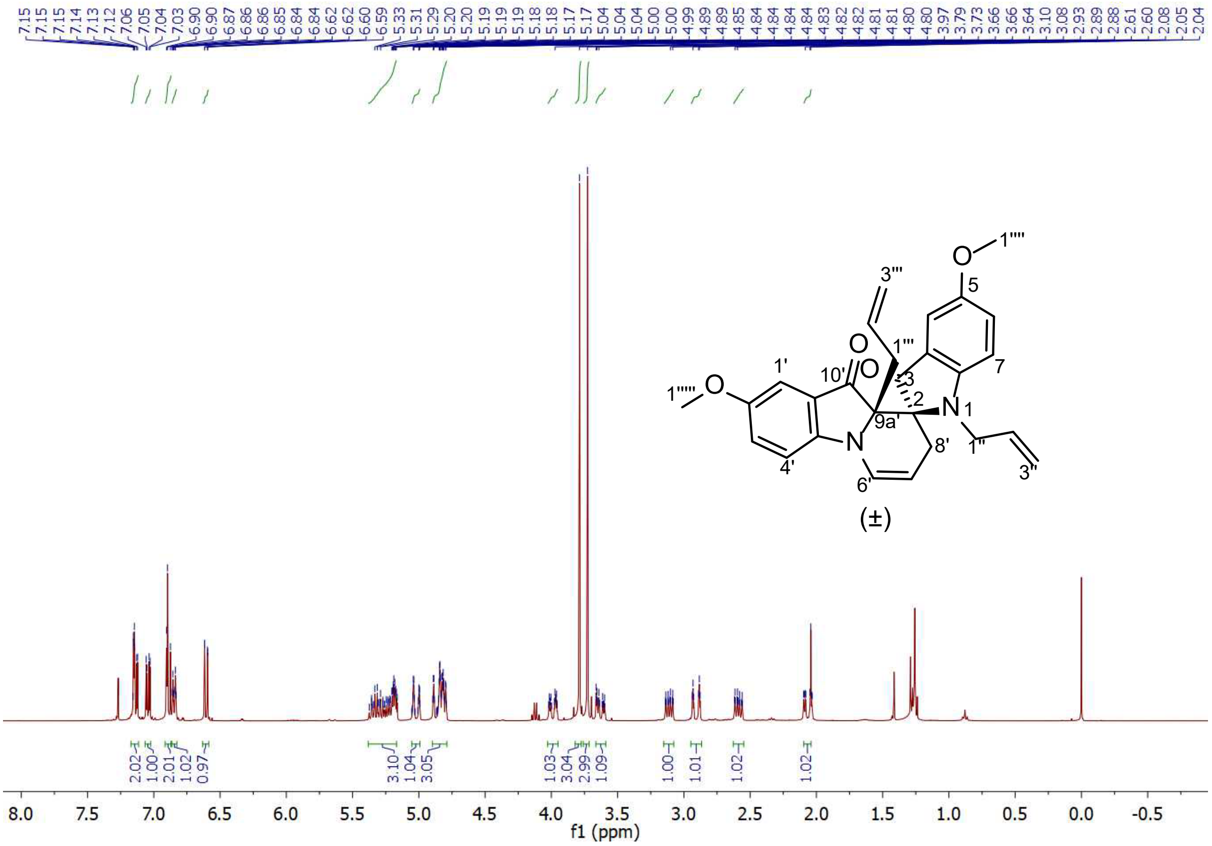

Figure S56: The ^{1}H NMR spectrum of *cisoid*-5,2'-dimethoxyspiroindolinepyridoindoledione **33a** (400 MHz, $CDCl_3$).

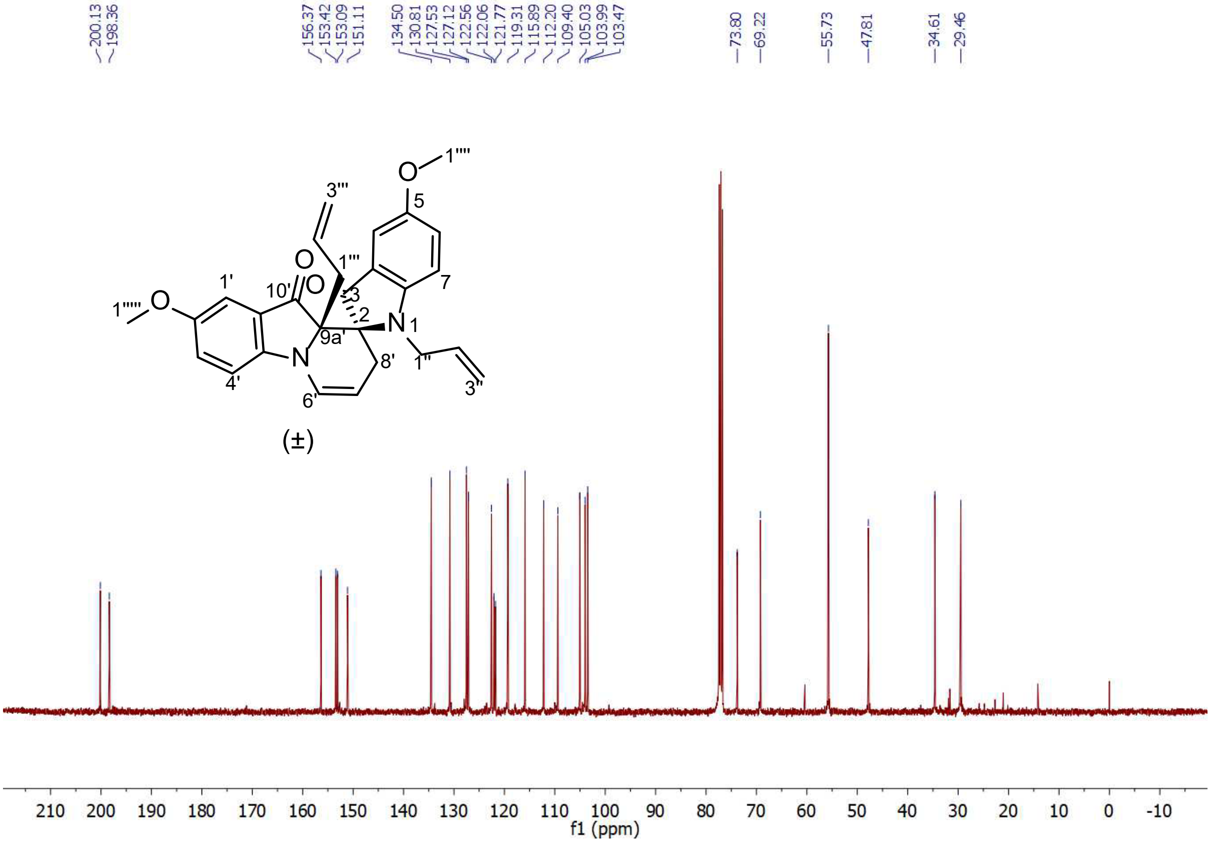

Figure S57: The ^{13}C NMR spectrum of *cisoid*-5,2'-dimethoxyspiroindolinepyridoindoledione **33a** (101 MHz, $CDCl_3$).

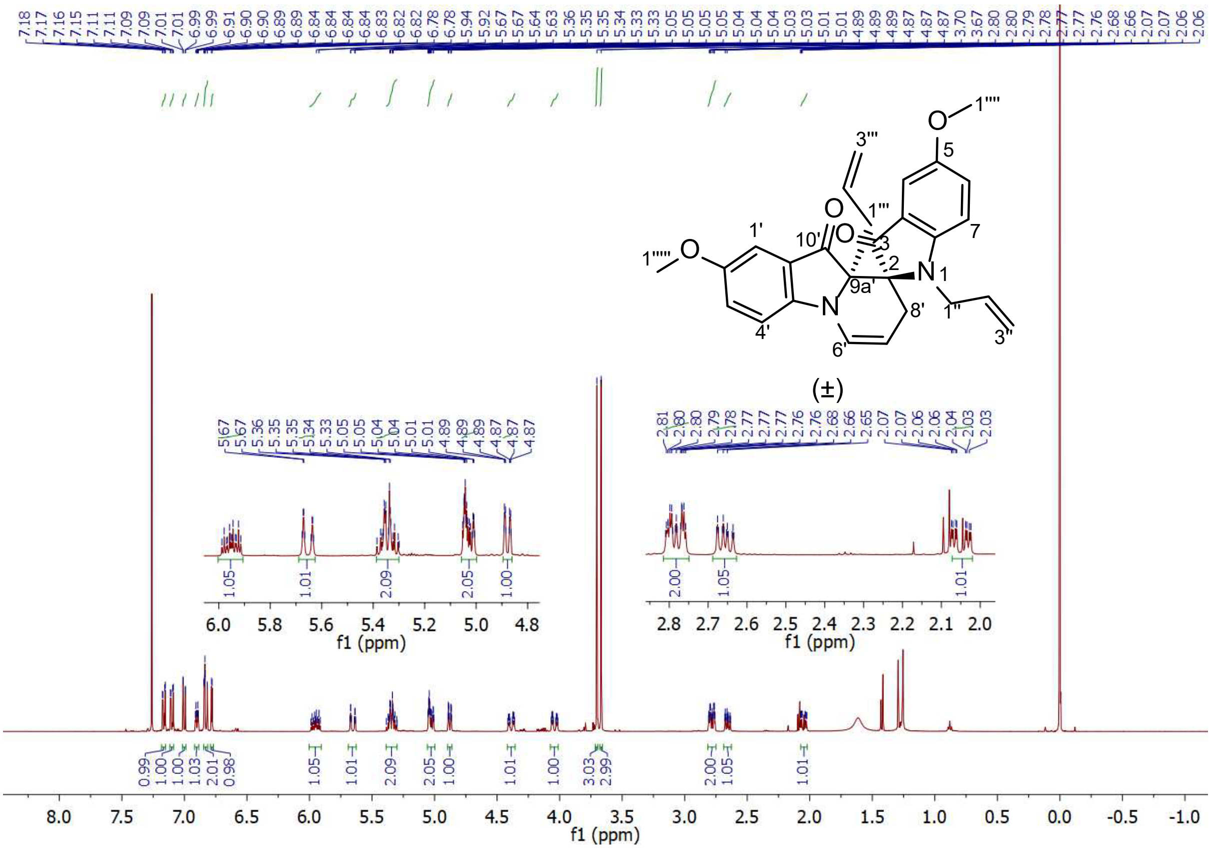

Figure S58: The ^{1}H NMR spectrum of *transoid*-5,2'-dimethoxyspiroindolinepyridoindoledione **33b** (400 MHz, $CDCl_3$).

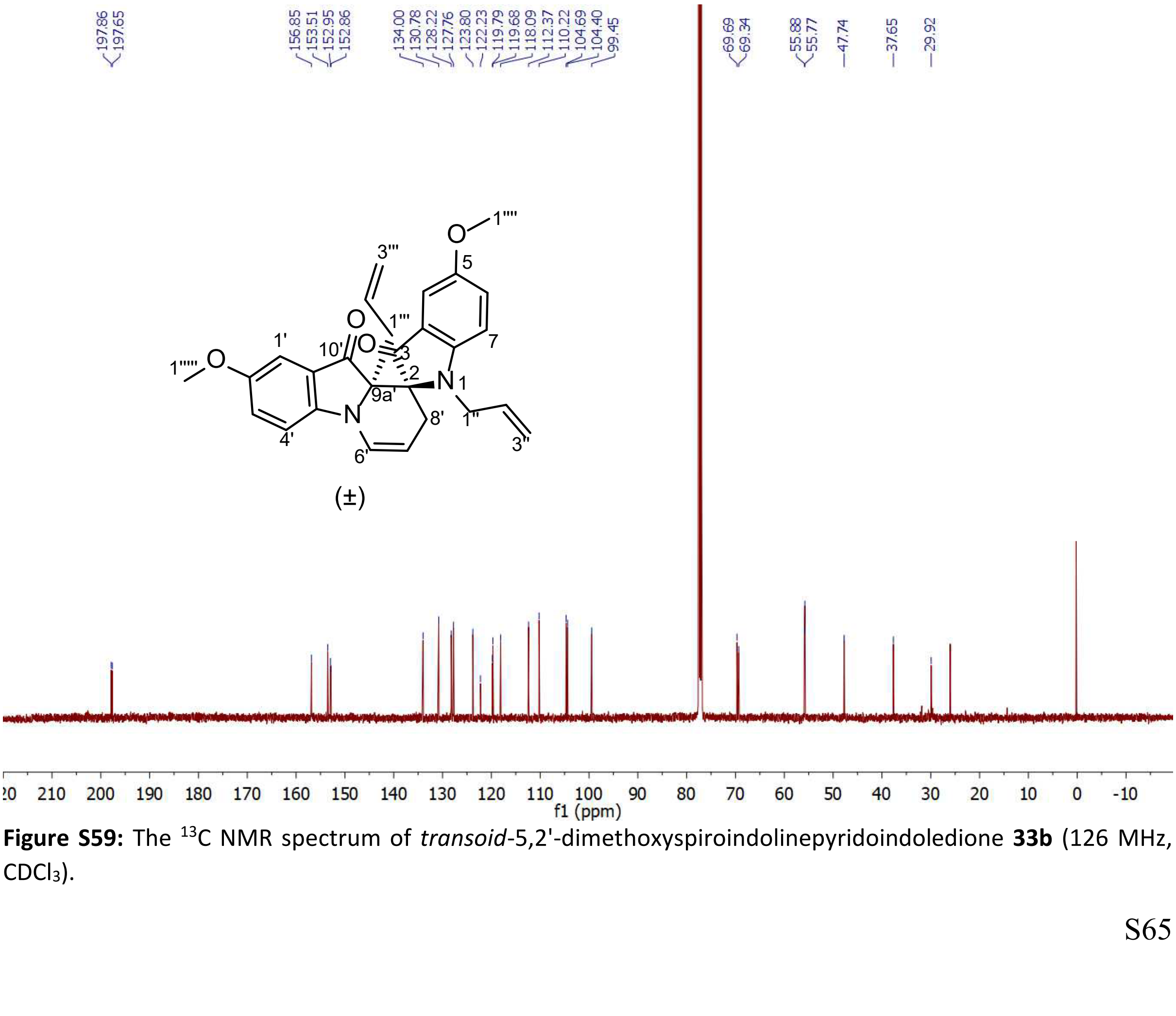

Figure S59: The ^{13}C NMR spectrum of *transoid*-5,2'-dimethoxyspiroindolinepyridoindoledione **33b** (126 MHz, $CDCl_3$).

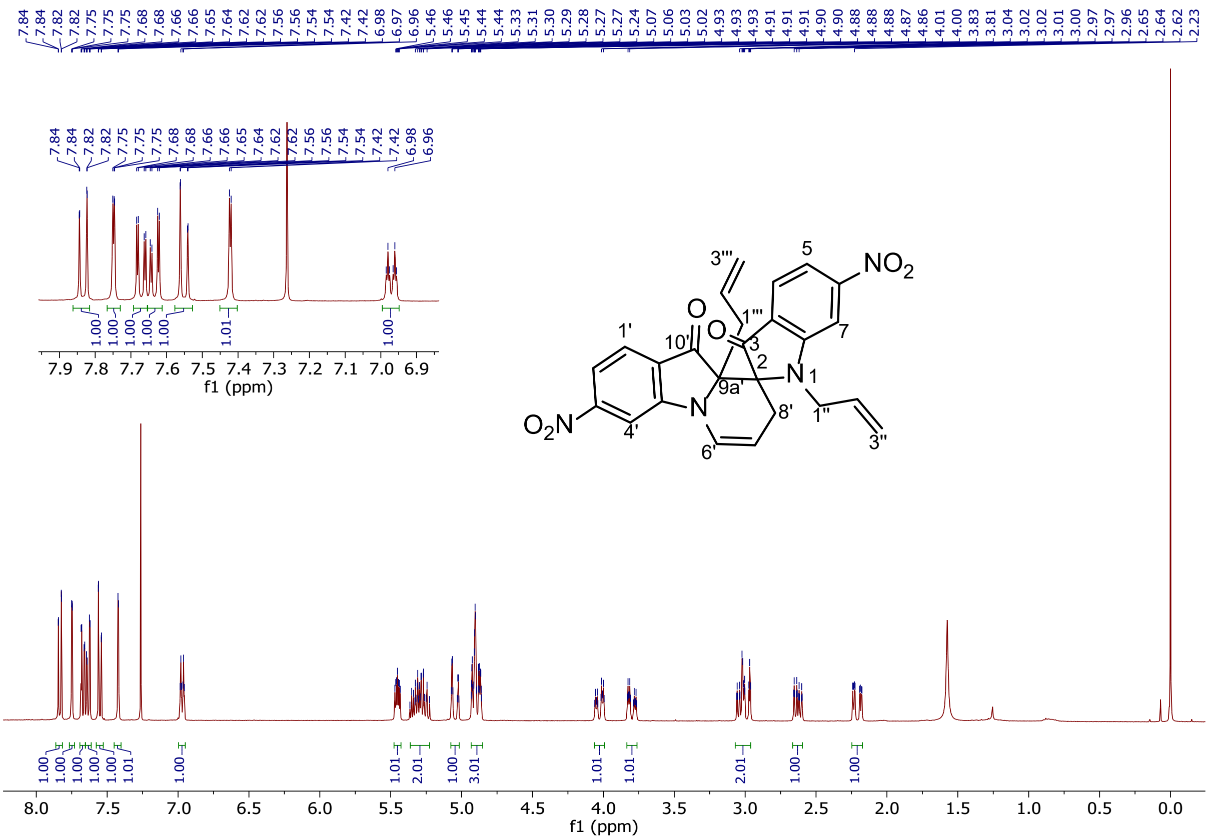

Figure S60: The ^{1}H NMR spectrum of 6,3'-dinitrospiroindolinepyridoindoledione **34** (400 MHz, $CDCl_3$).

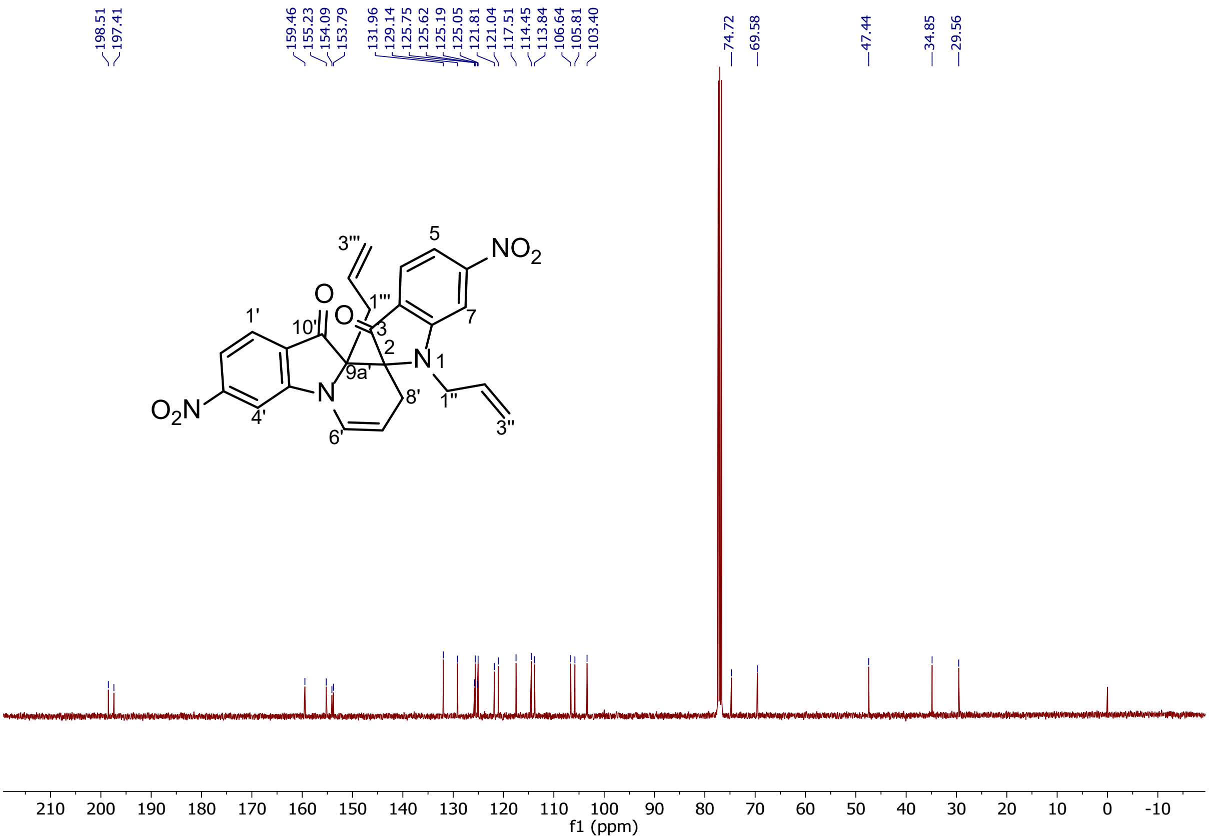

Figure S61: The ^{13}C NMR spectrum of 6,3'-dinitrospiroindolinepyridoindoledione **34** (101 MHz, $CDCl_3$).

S4. Structural Elucidation of Novel Polyheterocycles

S4.1 Structural elucidation of 3-acetoxy-5-nitroindole 10

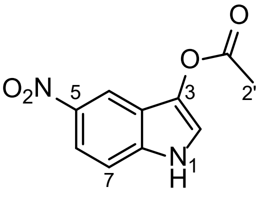

10

The synthesis of 3-acetoxy-5-nitroindole **10** was reported previously by Huang *et al.*,[18] however comparison between the ^{1}H NMR and ^{13}C NMR spectra reported here revealed discrepancies relative to spectra reported by Huang *et al.*[18] for **10** (Table S1), prompting complete structural elucidation of this derivative.

Analysis of the LRMS (ESI$^-$) of 3-acetoxy-5-nitroindole **10** revealed a peak at *m/z* 219, suggesting the addition of an acetate moiety to 5-nitroindole **9**. Examination of the ^{1}H NMR spectrum revealed three aromatic resonances at 8.57 ppm (dd, *J* = 2.3, 0.7 Hz), 8.13 ppm (dd, *J* = 9.0, 2.2 Hz) and 7.39 ppm (dd, *J* = 9.0, 0.6 Hz), assigned as H4, H6 and H7, respectively. The

Table S1: A comparison of the ^{1}H NMR and ^{13}C NMR chemical shifts (ppm) observed for 3-acetoxy-5-nitroindole **10** to previously reported data.[18]

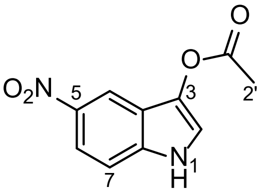

10

Spectrum	ppm (observed)	ppm (reported)[18]	Difference (ppm)
^{1}H NMR	8.57 (dd, *J* = 2.3, 0.7 Hz, 1H)	8.95 (s, 1 H)	+0.38
	8.27 (bs, 1H)	7.71 (s, 1 H)	-0.56
	8.13 (dd, *J* = 9.0, 2.2 Hz, 1H)		-0.69 – -0.82
	7.56 (d, *J* = 2.6 Hz, 1H)	7.44 – 7.31 (m, 3 H)	-0.12 – -0.25
	7.39 (dd, *J* = 9.0, 0.6 Hz, 1H)		+0.05 – -0.08
	2.41 (s, 3H)	2.05 (s, 3 H)	-0.36
^{13}C NMR	168.4	176.8	+8.4
	141.9	137.5	-4.4
	135.5	132.7	-2.8
	132.0	131.8	-0.2
	119.5	130.2	+10.7
	118.3	127.4	+9.1
	116.4	118.0	+1.6
	115.3	117.6	+2.3
	111.6	111.0	-0.6
	20.9	29.67	+8.77

presence of a broad singlet in the ^{1}H NMR spectrum at 8.27 ppm and the observation of a stretch at 3284 cm^{-1} in the IR spectrum were attributed to NH proton H1. Analysis of the HMBC spectrum revealed a strong correlation between protons H4 and H6 and a ^{13}C resonance at 135.5 ppm, assigned as C7a, which further correlated to an aromatic ^{1}H NMR resonance at 7.56 ppm, assigned as H2 (Figure S62, red). Protons H2, H4 and H7 were also observed to correlated to a carbon resonance at 132.0 ppm, assigned as C3 (Figure S62, blue). A weak correlation between C3 and a singlet integrating to three protons at 2.41 ppm, assigned as acetoxy methyl protons H2' (Figure S62, green). Analysis of the HRMS of revealed a prominent peak at *m/z* 219.0397, confirming the molecular formula $C_{10}H_7N_2O_4^-$, assigned as the [M-H]$^-$ ion of **10**. The mp observed was from 139 – 143 °C, comparable to that obtained by Davis *et al*. in the first reported synthesis of **10** (138 – 140 °C)[24] and unlike that reported by Huang *et al*. (172 – 173 °C).[18]

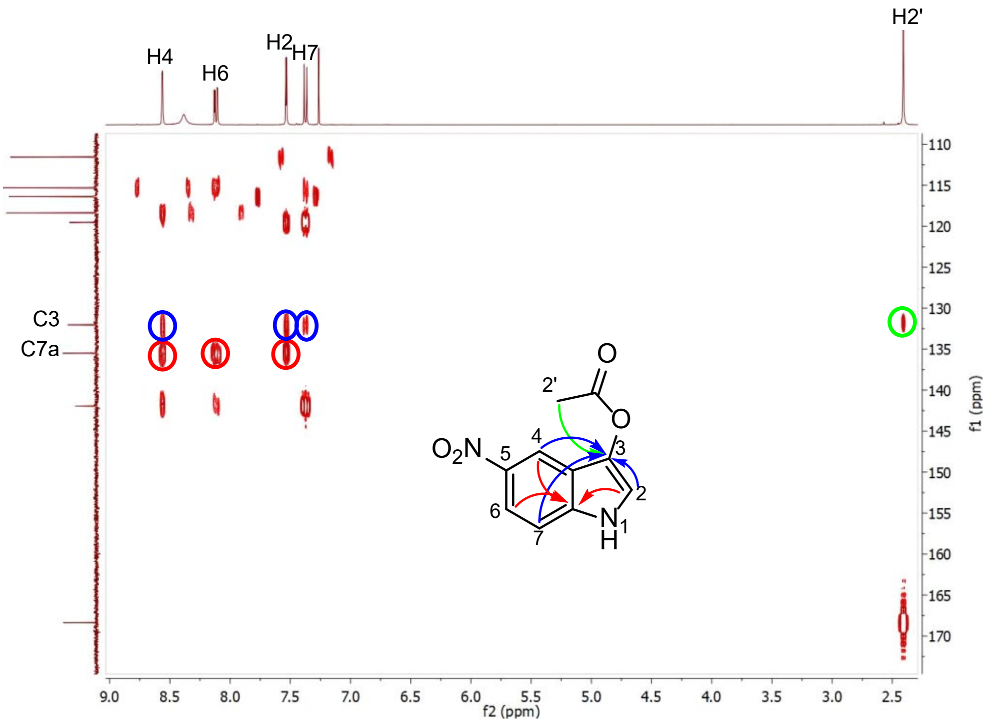

Figure S62: Analysis of the HMBC spectrum of 3-acetoxy-5-nitroindole **10**.

S4.2 Structural elucidation of *N,N',O,O'*-tetraBoc-biindole-3,3'-diols 11 and 13

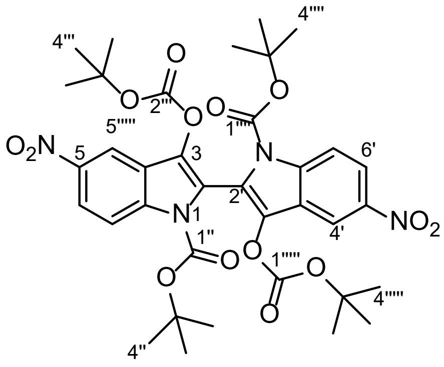

11

Examination of the LRMS (ESI$^+$) of *N,N',O,O'*-tetraBoc-5,5'-dinitrobiindole-3,3'-diol **11** revealed the base peak at *m/z* 777, assigned as the $[M+Na]^+$ ion, which indicated the addition of four Boc moieties to 5,5'-dinitroindigo **1h**. Analysis of the ^{1}H NMR spectrum revealed three aromatic resonances and two alkyl resonances, indicating a high degree of symmetry. Proton resonances at 8.53 ppm (d, *J* = 9.3 Hz), 8.45 ppm (d, *J* = 2.2 Hz) and 8.30 ppm (dd, *J* = 9.3, 2.3 Hz) were assigned as H7 & H7', H4 & H4' and H6 & H6', respectively. Analysis of the NOESY spectrum of **11** revealed a prominent resonance between H7 & H7' and a singlet integrating to eighteen protons at 1.25 ppm, assigned as *N*-Boc protons H4'' & H4'''' (Figure S63, blue). Further analysis revealed a second correlation between H4 & H4' and a singlet integrating to eighteen protons at 1.42 ppm, assigned as *O*-Boc protons H4''' & H4''''' (Figure S63, red). The HRMS (ESI$^+$) showed a major peak at *m/z* 777.2603 confirming the molecular formula $C_{36}H_{42}N_4O_{14}Na^+$, assigned as the $[M+Na]^+$ ion of **11**.

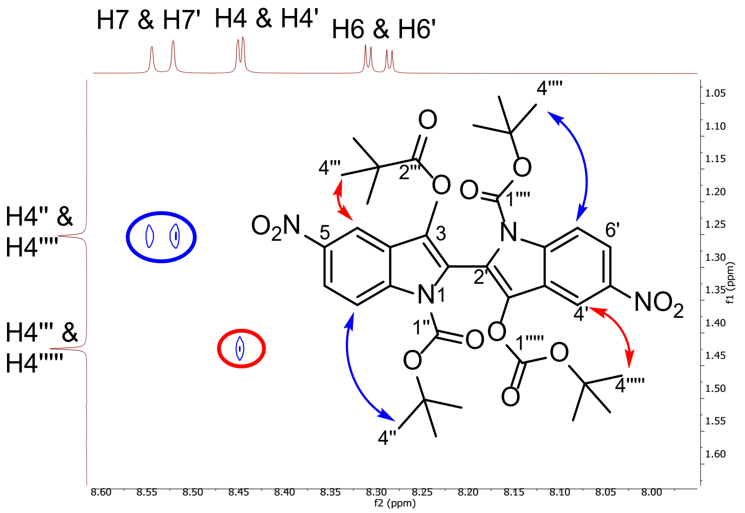

Figure S63: Analysis of the NOESY spectrum of **11**.

Analysis of the NMR spectra of *N,N',O,O'*-tetraBoc-6,6'-dinitrobiindole-3,3'-diol **13** revealed similar spectral properties to the 5,5'-dinitro derivative **11** and was assigned based upon key 2D NMR correlations (Figure S64). Examination of the HRMS (ESI$^+$) revealed a peak at

m/z 777.2612 confirming the molecular formula $C_{36}H_{42}N_4O_{14}Na^+$, assigned as the $[M+Na]^+$ ion of **13**.

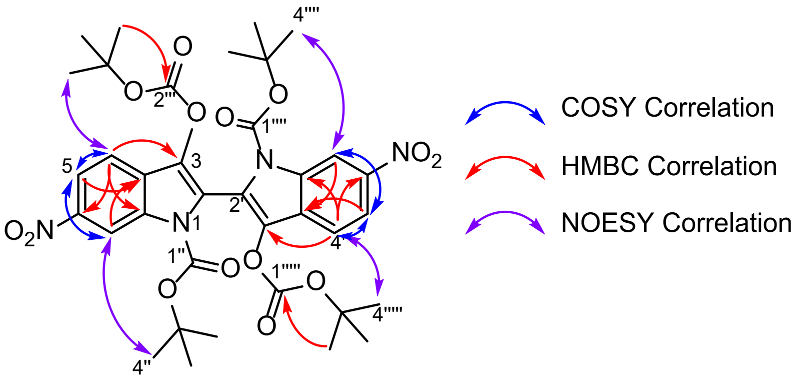

13

Figure S64: Key 2D NMR correlations of *N*,*N'*,*O*,*O'*-tetraBoc-6,6'-dinitroindigo **13**.

S4.3 Structural elucidation of diallylbiindolone 16

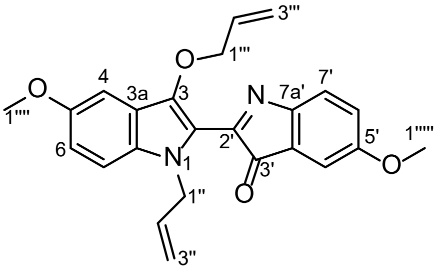

16

The LRMS spectrum of **16** showed a prominent peak at m/z 403, assigned as the $[M+H]^+$ ion, suggesting the addition of two allyl substituents to 5,5'-dimethoxyindigo **1a**. The ^1H NMR spectrum showed two methoxy resonances at 3.86 ppm and 3.85 ppm, six resonances in the aromatic region and allyl-associated resonances integrating to 10 protons in total, indicating the presence of asymmetry in **16** which would not be observed in the case of *N*,*N'*-diallylation. Correlations were observed in the COSY spectrum between resonances at 7.22 ppm, 7.08 ppm and 6.97 ppm, assigned as H7, H4 and H6, respectively (Figure S65, purple, red). Further analysis showed correlations between ^1H NMR resonances at 7.28 ppm, 7.13 ppm and a second proton in the multiplet at 6.97 ppm, assigned as H7', H4' and H6', respectively (Figure S65, black, blue). Examination of the ^1H NMR spectrum revealed that protons H4, H6 and H7 were further upfield than H4', H6' and H7', respectively, suggesting the *N*-allyl and *O*-allyl substituents are shielding these protons.

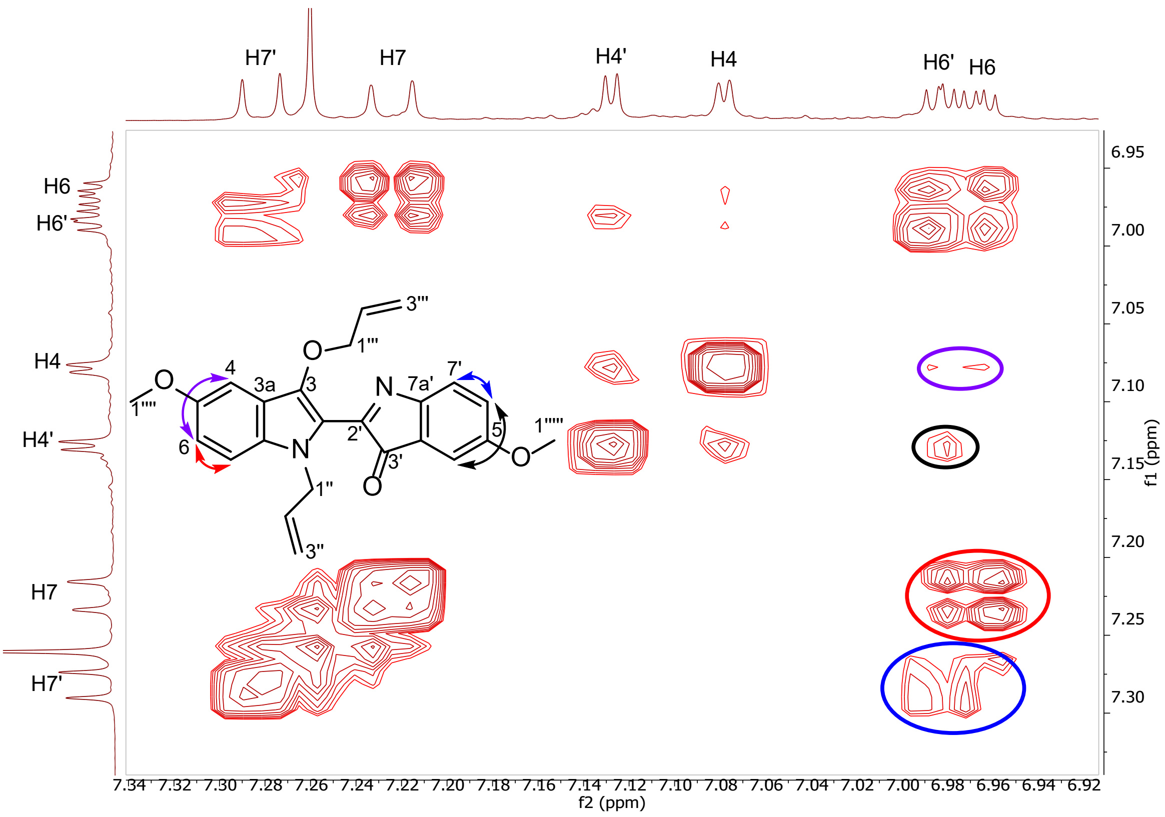

Figure S65: Analysis of the COSY spectrum of diallylbiindolone **16**.

Analysis of the HMBC spectrum of **16** revealed a strong correlation between H4' and a ^{13}C resonance at 191.3 ppm, assigned as C3' (Figure S66, black). Further examination revealed a correlation between H4 and a carbon resonance at 144.1 ppm, assigned as C3, which also correlated to a resonance at 4.78 ppm integrating to 2 protons, assigned as H1''' (Figure S66, blue). A strong correlation was also observed between H4 and a carbon resonance at 134.08 ppm, assigned as C7a, which further correlated to a proton multiplet at 5.11 – 5.00 ppm assigned as H1''. Protons H1'' and H1''' were also observed to correlate to carbon resonances at 117.8 ppm and 116.3 ppm, assigned as alkenyl carbons C3'' and C3''', respectively (Figure S66, purple, magenta). The position of the two allyl substituents was confirmed by the observation of strong NOESY correlations between H1'' & H7 and H1''' & H4 (Figure S67, red, black). Analysis of the HRMS revealed a peak at *m/z* 403.1645, confirming the molecular formula $C_{24}H_{23}N_2O_4^+$, assigned as the $[M+H]^+$ ion of **16**.

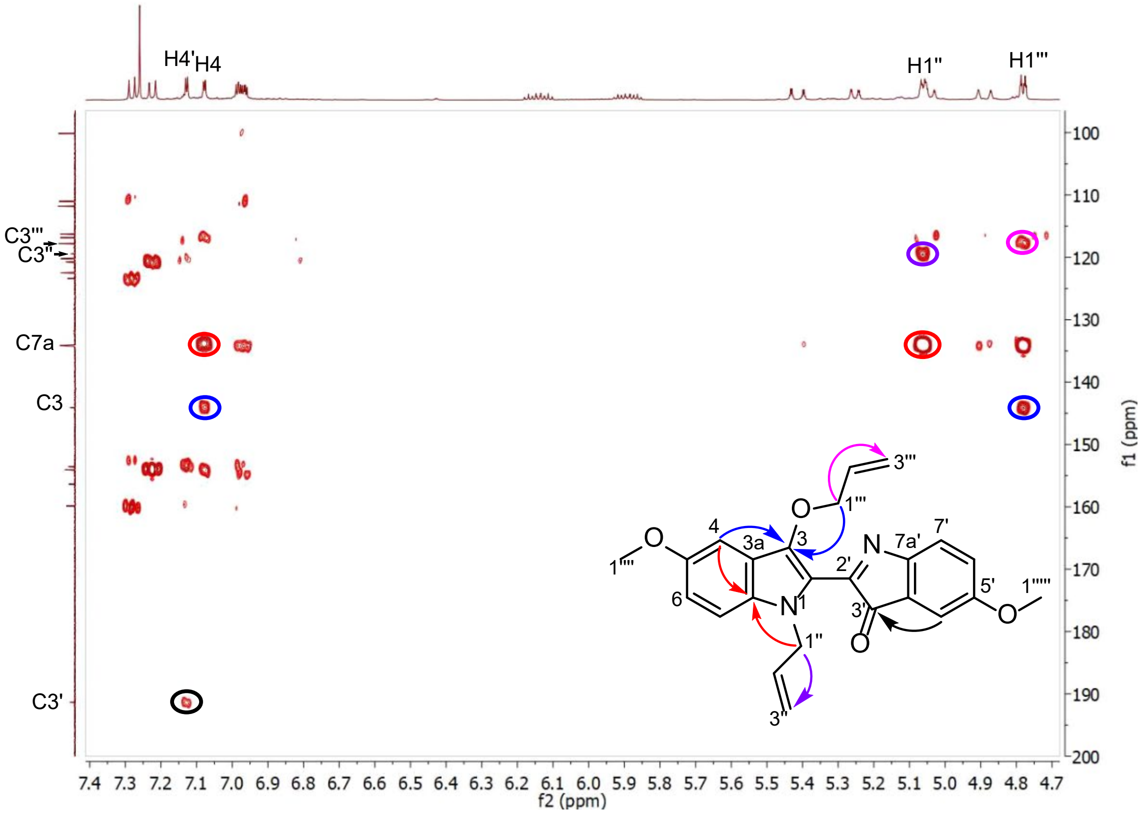

Figure S66: Analysis of the HMBC spectrum of diallylbiindolone **16**.

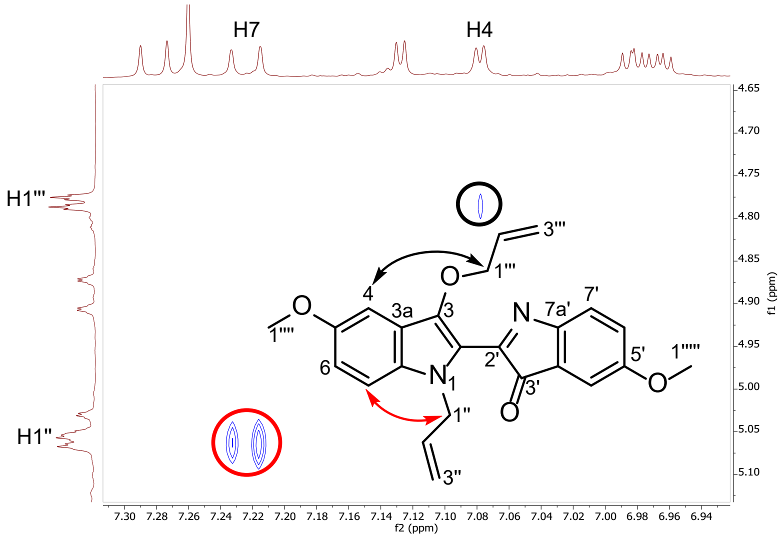

Figure S67: Analysis of the NOESY Spectrum of diallylbiindolone **16**.

S4.4 Structural elucidation of spiroindolinepyridoindolones 17 – 25

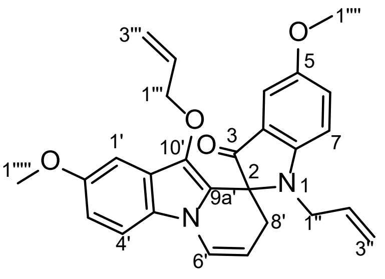

17

As a typical example, analysis of the HRMS (ESI$^+$) of 5,2'-dimethoxyspiroindolinepyridoindolone **17** a peak at *m/z* 443.1967, confirming the molecular formula $C_{27}H_{27}N_2O_4^+$, assigned as the [M+H]$^+$ ion of **17**. The COSY spectrum showed strong and weak correlations between a multiplet at 7.18 – 7.11 ppm and proton resonances at 6.68 ppm and 7.12 ppm, assigned as H6, H7 and H4, respectively (Figure S68, red, black). A second proton within the multiplet at 7.18 – 7.11 ppm showed strong and weak COSY correlations to protons at 7.28 ppm and 6.94 ppm, assigned as H3', H4' and H1', respectively (Figure S68, blue, green).

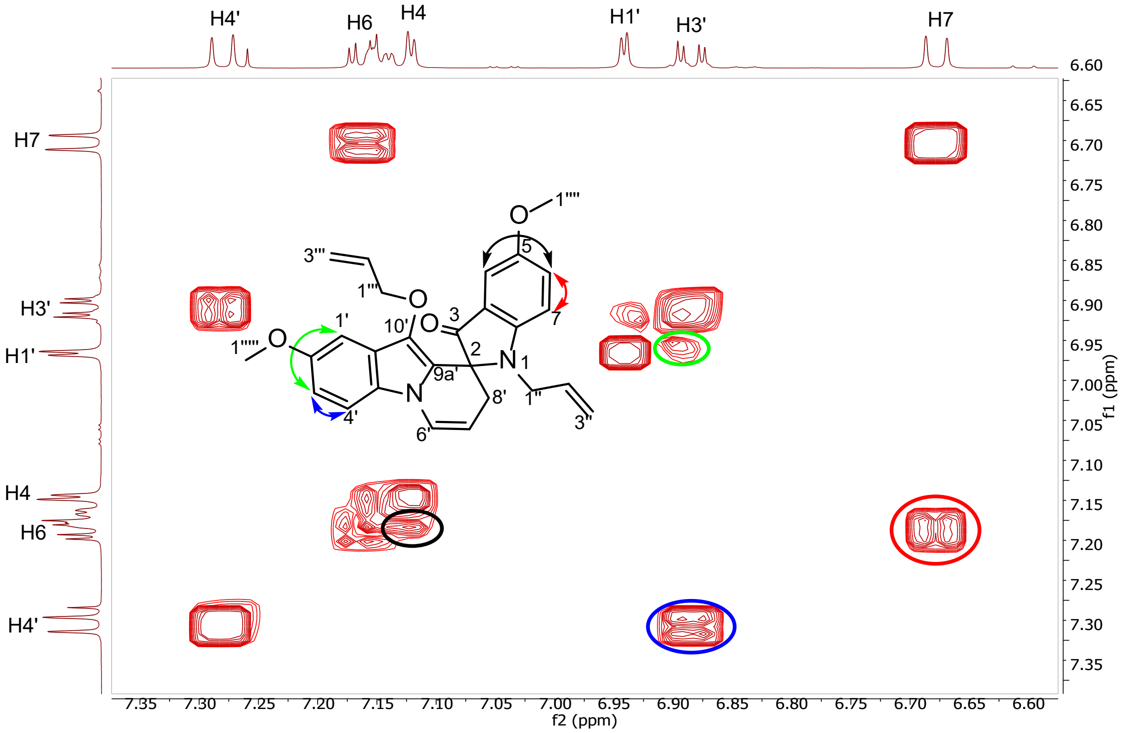

Figure S68: Analysis of the COSY spectrum of 5,2'-dimethoxyspiroindolinepyridoindolone **17**.

The HMBC spectrum of **17** showed a strong correlation between H4 and a carbon resonance at 200.7 ppm, assigned as C3 (Figure S69, black). Protons H4 and H6 also exhibited strong HMBC correlations to the carbon resonance at 155.7 ppm, assigned as the bridgehead C7a, which further correlated to the proton multiplet at 3.87 – 3.71 ppm, assigned as H1'' (Figure S69, purple). Protons H1'' exhibited two strong HMBC correlations to a carbon at 67.0 ppm, assigned as spirocyclic carbon C2 (Figure S69, blue). Carbon C2 exhibited further

correlations to proton resonances at 2.89 ppm, 2.43 ppm and 5.42 ppm, assigned as diastereotopic protons H8'a and H8'b and alkenyl proton H7' (Figure S69, blue). The HSQC spectrum of **17** showed both H8'a and H8'b exhibited strong correlations to a carbon at 31.9 ppm, assigned as C8'. Prominent HMBC correlations were observed from C8' to proton H7' and a resonance at 7.18 – 7.11 ppm, with the remaining proton in this multiplet assigned as deshielded alkenyl proton H6' (Figure S69, magenta). Two strong HMBC correlations were observed from protons H6' and H8'b to a carbon resonance at 121.7 ppm, assigned as quaternary carbon C9a' (Figure S69, teal).

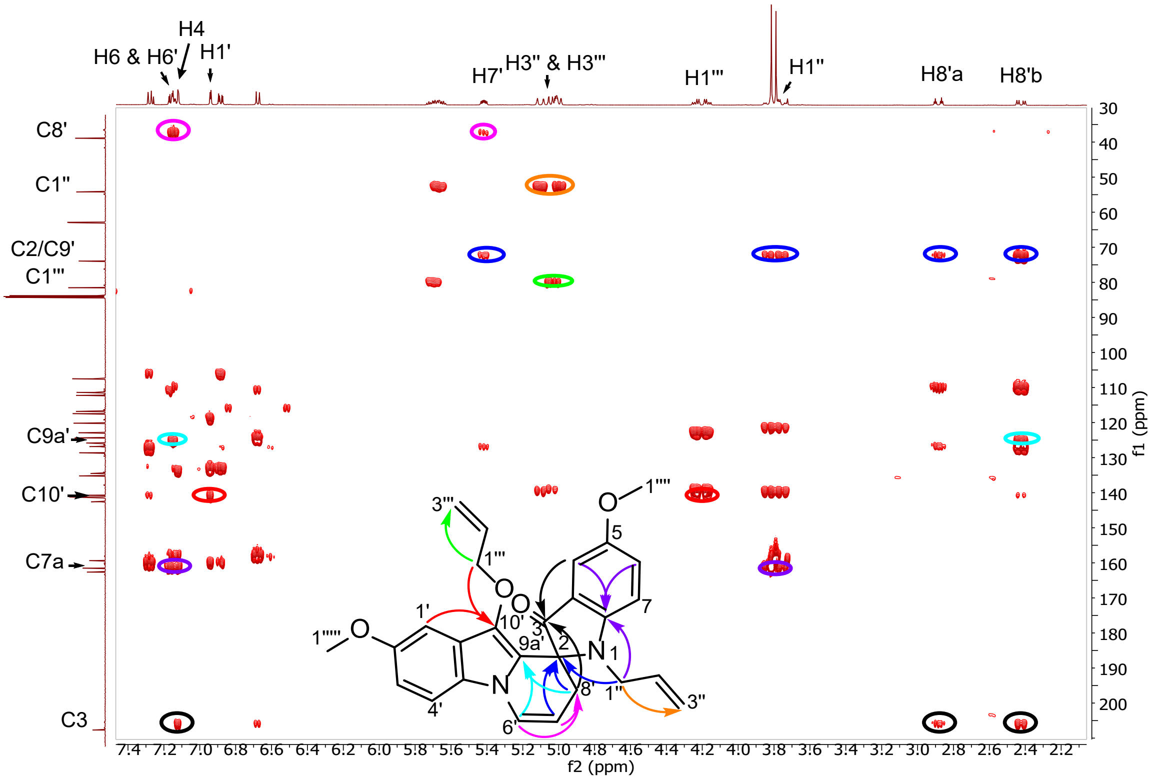

Figure S69: Analysis of the HMBC spectrum of spiroindolinepyridoindolone **17**.

Further analysis revealed the presence of a strong HMBC correlation between H1' and an aromatic carbon resonance at 135.6 ppm, assigned as C10', which further correlated to proton resonances at 4.24 ppm and 4.17 ppm, assigned as AB quartet H1''' (Figure S69, red). The HSQC spectrum of **17** displayed correlations from protons H1'' and H1''' to carbon resonances at 47.2 ppm and 74.5 ppm, assigned as C1'' and C1''', respectively. Both C1'' and C1''' showed strong HMBC correlations to four protons in ^{1}H NMR resonances at 5.10 ppm and 5.07 – 4.98 ppm, assigned as terminal alkenyl protons H3'' and H3''' (Figure S69, orange, green). All other spiroindolinepyridoindolones **18** – **25** exhibited near-identical spectroscopic

properties with the exception of the positioning of the aromatic protons and were identified according to the key COSY, HMBC and NOESY correlations and HRMS data (Figure S70).

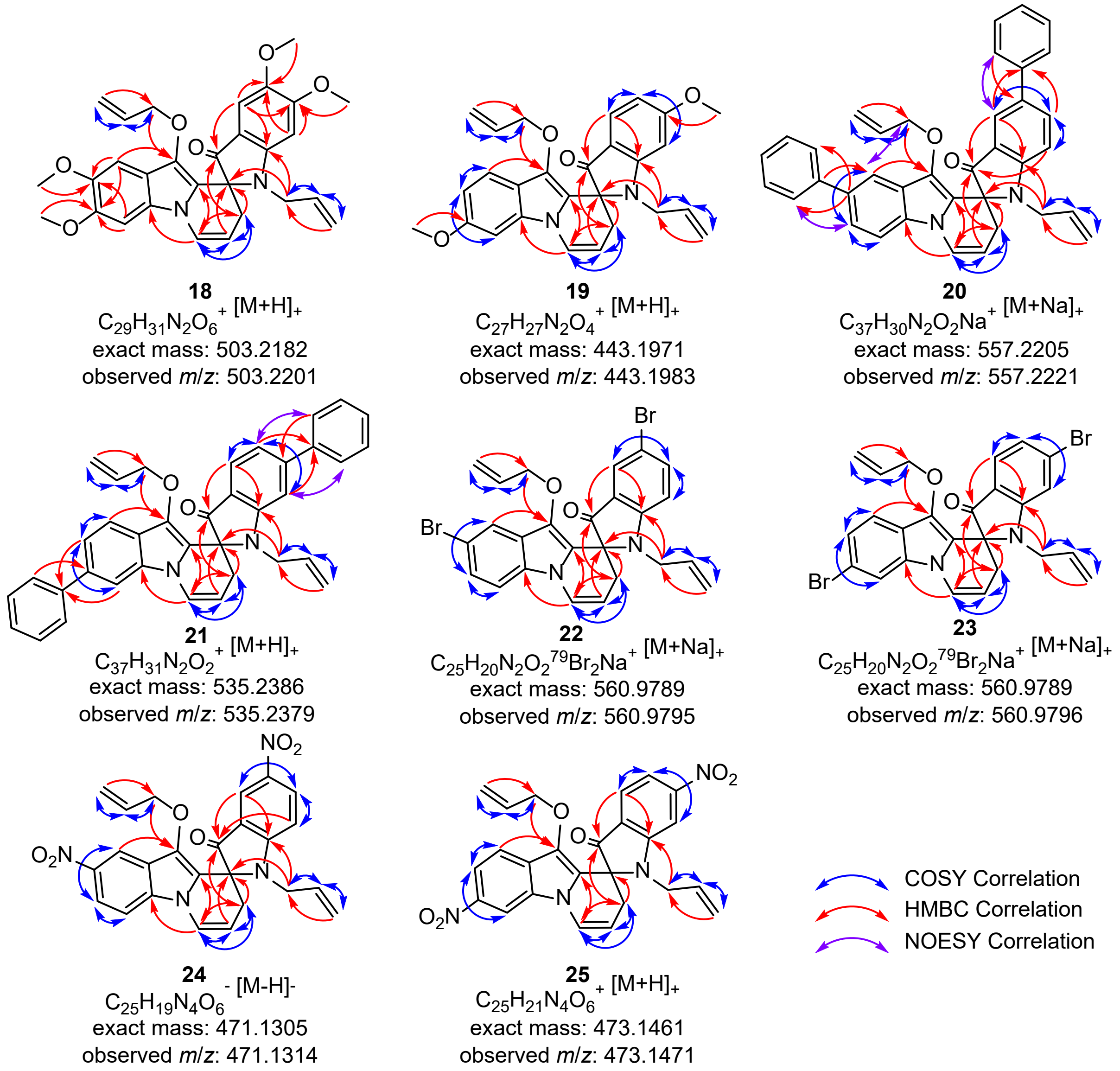

Figure S70: Summary of the HRMS data and key COSY, HMBC and NOESY correlations observed in spiroindolinepyridoindolones **18** – **25**.

S4.5 Structural elucidation of *cisoid C*-allylspiroindolinepyridoindolediones 26 – 29

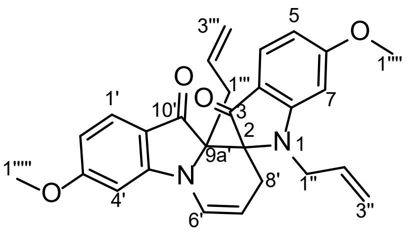

27

As a typical example, the LRMS of **27** showed a signal at *m/z* 443, assigned as the $[M+H]^+$ ion, indicating the addition of three allyl moieties to 6,6'-dimethoxyindigo **1c**. Analysis of the aromatic region of the COSY spectrum of **27** showed a strong and weak correlation between multiplet 6.39 – 6.35 ppm and doublets at 7.61 ppm and 6.02 ppm, assigned as H5, H4 and H7, respectively

(Figure S71, blue, red). The second aromatic hemisphere of **27** was assigned based upon the strong and weak COSY correlations observed between a second proton in the multiplet at 6.39 – 6.35 ppm and proton resonances at 7.38 ppm and 6.32 ppm, assigned as H2', H1' and H4', respectively (Figure S71, black, purple).

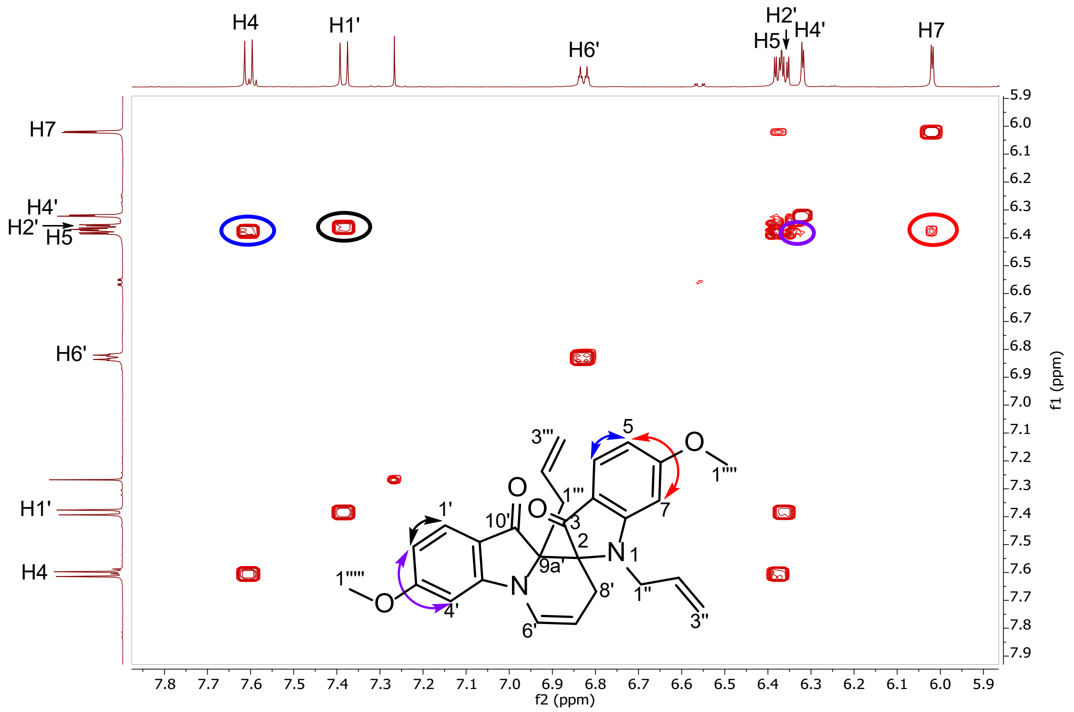

Figure S71: Analysis of the aromatic COSY correlations of *C*-allylspiroindolinepyridoindoledione **27**.

Analysis of the HMBC spectrum revealed a strong correlation between H4 and two carbon resonances at 197.2 ppm and 162.2 ppm, assigned as C3 and C7a, respectively (Figure S72, teal, lime). Bridgehead C7a was also observed to correlate to ^{1}H NMR resonances at 3.94 ppm and 3.70 ppm, assigned as AB quartet H1'', which further correlated to a ^{13}C NMR resonance at 68.8 ppm, assigned as spirocyclic carbon C2 (Figure S72, green, blue). Further correlations were observed between C2 and proton resonances at 3.11 ppm, 2.57 ppm, 2.94 ppm and 2.07 ppm, assigned as H1'''a, H1'''b, H8'a and H8'b, respectively (Figure S72, blue). A strong correlation was also observed between H8'a and C3 (Figure S72, teal). A HMBC correlation was seen between proton H1' and a carbon resonance at 195.9 ppm, assigned as carbonyl C10', which was also observed to correlate strongly to H1''' (Figure S72, magenta).

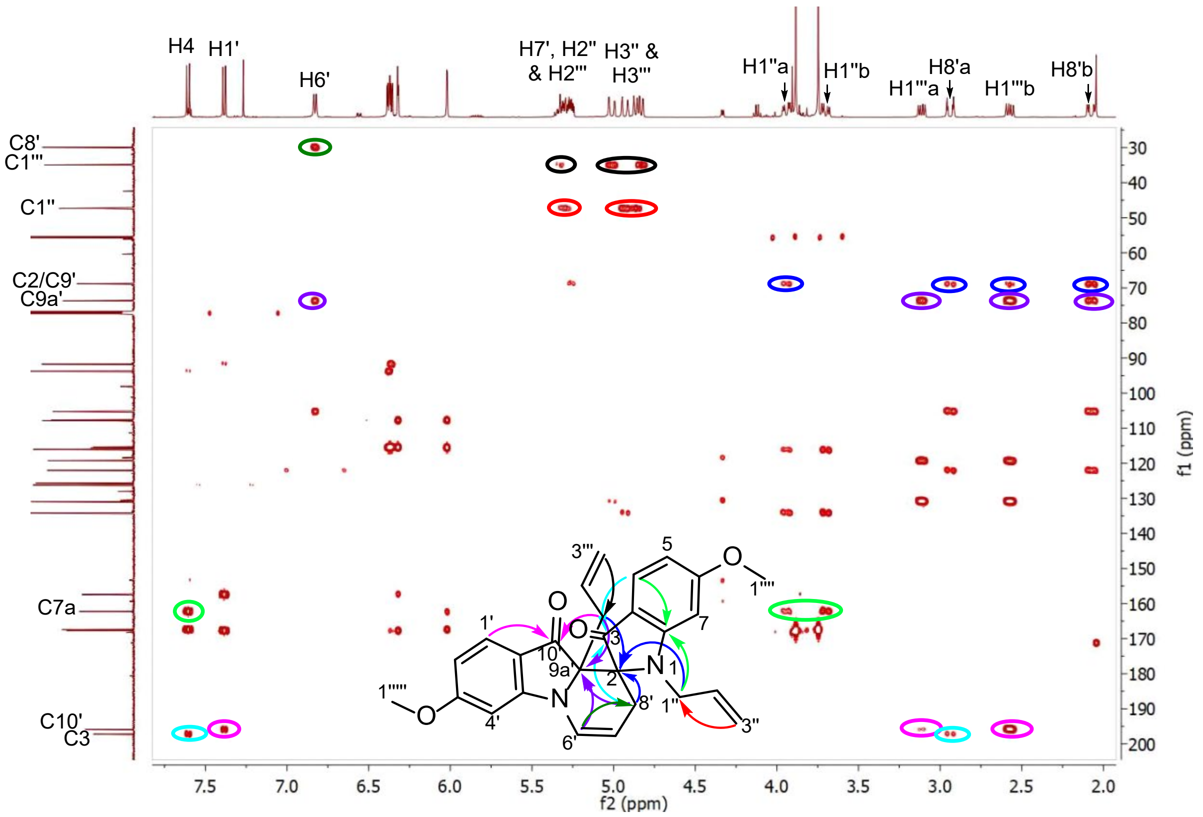

Figure S72: Analysis of the HMBC spectrum of *C*-allylspiroindolinepyridoindoledione **27**.

Analysis of the HSQC spectrum revealed the presence of strong correlations between protons H8', H1'' and H1''' and carbon resonances at 29.9 ppm, 47.3 ppm and 35.0 ppm, assigned as C8', C1'' and C1''', respectively (Figure S73a, black, red, green). The proton resonance at 6.83 ppm exhibited a strong HMBC correlation to C8', assigned as H6' (Figure S72, green). Protons H8'b, H1''' and H6' were also observed to correlate to a carbon resonance at 73.7 ppm, assigned as C9a' (Figure S72, purple). The position of H6' was confirmed by the observation of a strong NOESY correlation to H4' (Figure S73b, teal). Carbons C1'' and C1''' exhibited strong HMBC correlations to four alkenyl protons at 4.93 ppm, 4.87 ppm, 5.01 ppm and 4.83 ppm, assigned as H3''a, H3''a, H3'''a and H3'''b, respectively. The HRMS (ESI$^+$) showed a peak at *m/z* 443.1987, confirming the molecular formula $C_{27}H_{27}N_2O_4^+$, assigned as the $[M+H]^+$ ion of **27**.

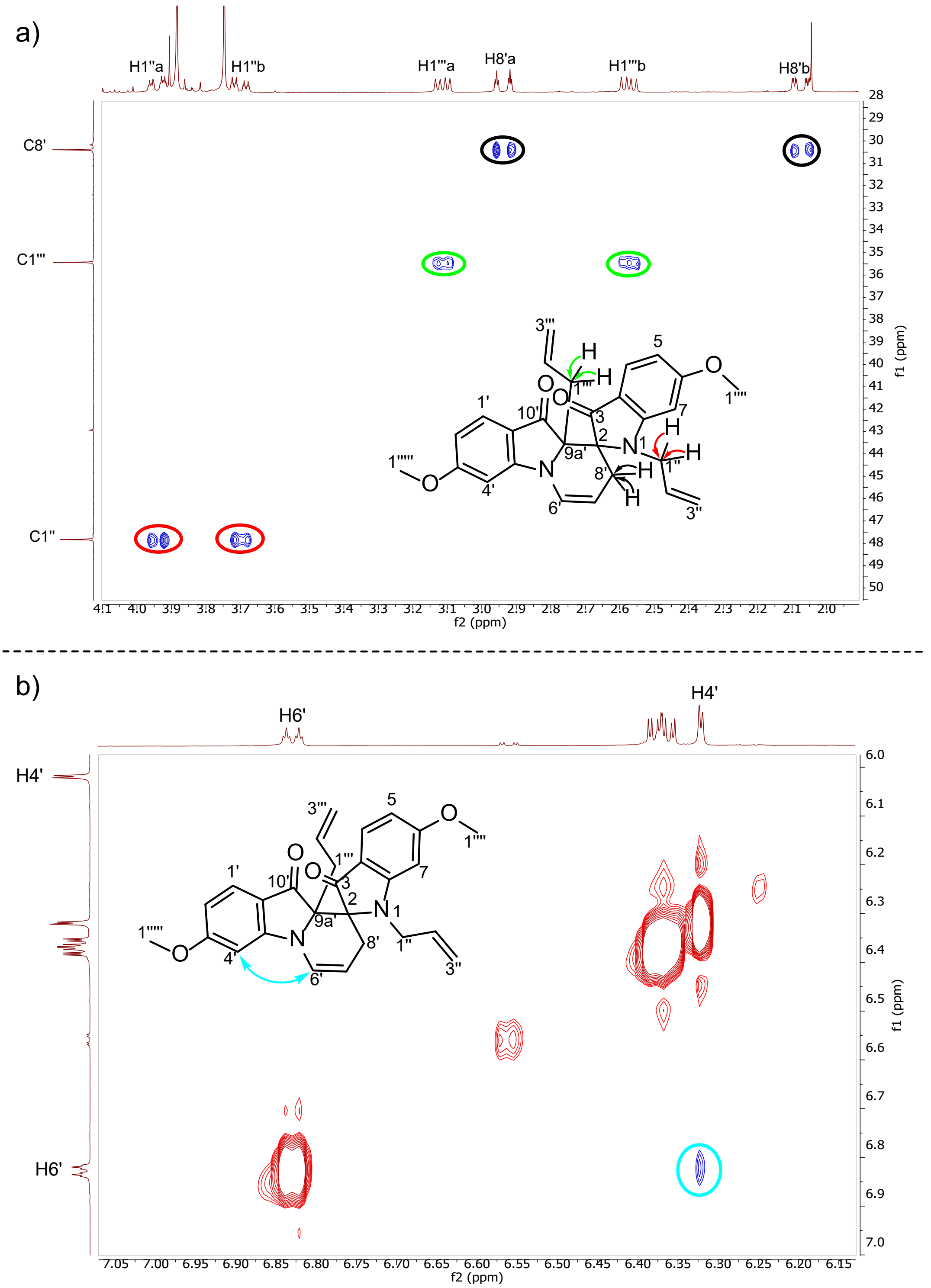

Figure S73: Analysis of the a) HSQC and b) NOESY spectra of *C*-allylspiroindolinepyridoindoledione **27**.

Analysis of the NMR spectra of *C*-allylspiroindolinepyridoindolediones **26**, **28** – **29** and **33a** showed identical spectral characteristics to **27**, and were assigned using the key COSY, HMBC and NOESY correlations with the HRMS data (Figure S74).

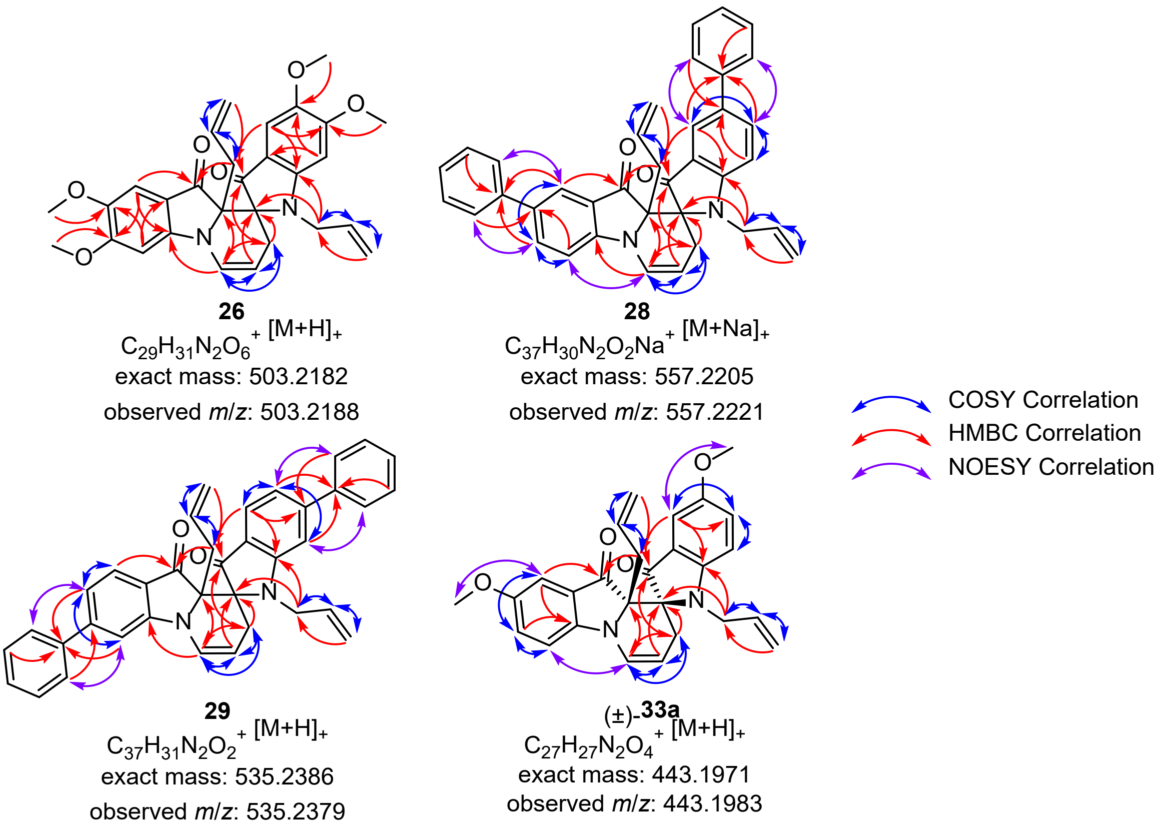

Figure S74: Summary of HRMS and key COSY, HMBC and NOESY correlations observed in *C*-allylspiroindolinepyridoindolediones **26**, **28, 29** and **33a**.

S4.6 Structural elucidation of oxazinodiindole 30

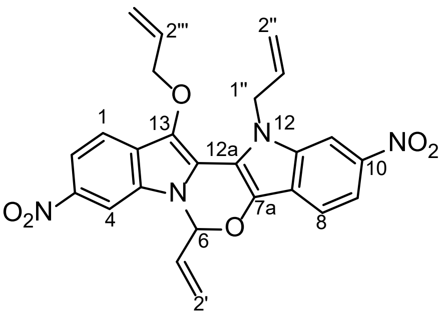

30

Examination of the LRMS (ESI$^+$) of **30** revealed the presence of a signal at *m*/*z* 473, assigned as the [M+H]$^+$ ion, suggesting the addition of three allyl moieties to 6,6'-dinitroindigo **1i** had occurred. Analysis of the alkenyl region of the HSQC spectrum revealed the presence of three terminal alkenyl moieties (teal), three internal alkenyl motifs (black), however only two methylene motifs (red) were observed with the addition of one deshielded methine moiety (green) with ^{1}H and ^{13}C NMR chemical shifts of 6.81 ppm and 84.0 ppm, respectively (Figure S75).

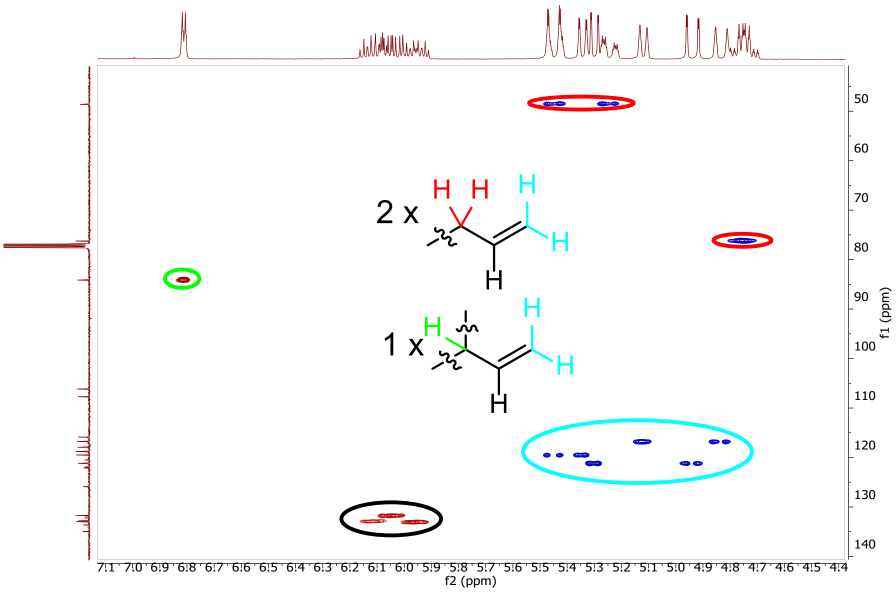

Figure S75: Analysis of the alkenyl region of the HSQC spectrum of oxazinodiindole **30**.

Examination of the ^{1}H NMR spectrum revealed two doublets at 7.72 ppm (J = 8.8 Hz) and 7.70 ppm (J = 8.9 Hz) with large coupling constants suggestive of J_3 coupling, assigned as H1 and H8, respectively. Analysis of the HMBC spectrum revealed the presence of a strong correlation between H8 and a carbon resonance at 134.8 ppm, assigned as bridgehead C11a, which was observed to correlate to proton resonances at 5.49 – 5.41 ppm and 5.25 ppm, assigned as H1''a and H1''b, respectively (Figure S76, blue). Proton H1 exhibited a strong HMBC correlation to a carbon resonance at 132.1 ppm, assigned as C4a, which further correlated to the methine proton resonance at 6.81 ppm, assigned as H6 (Figure S76, purple). Another strong HMBC correlation was observed between H1 and a carbon resonance at 131.7 ppm, assigned as C13, which further correlated to proton resonances at 4.78 ppm and 4.72 ppm, assigned as H1'''a and H1'''b (Figure S76, red). Analysis of the HSQC spectrum revealed the presence of strong correlations between H6, H1'' and H1''' and carbon resonances at 84.0 ppm, 48.4 ppm and 76.1 ppm, assigned as C6, C1'' and C1''', respectively (Figure S75). Carbons C6 and C1'' showed strong HMBC correlations to four proton resonances at 5.30 ppm, 4.94 ppm, 5.12 ppm and 4.83 ppm, assigned as terminal alkenyl protons H2'a, H2'b, H3''a and H3''b, respectively (Figure S76, orange, magenta). A strong correlation was also observed between H1''' and a carbon resonance at 119.4 ppm, assigned as C3''' (Figure S76, green).

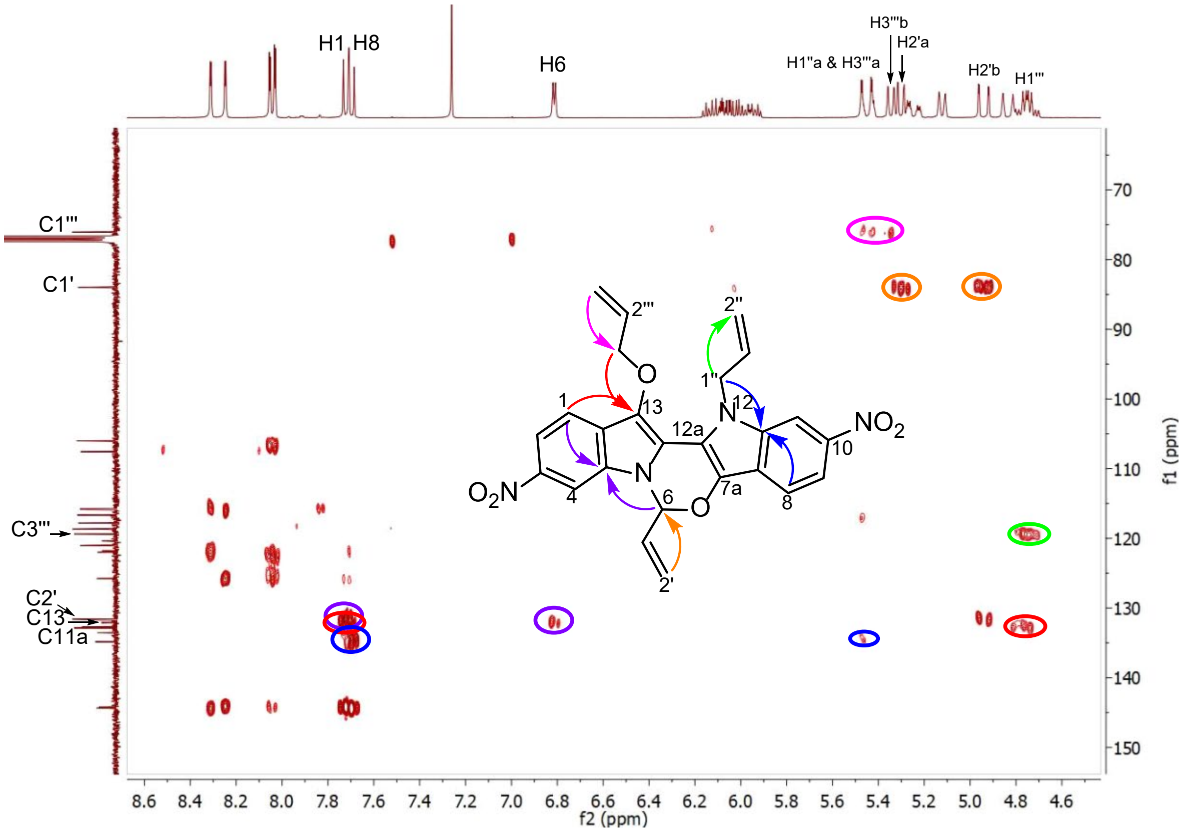

Figure S76: Analysis of the HMBC spectrum of oxazinodiindole **30**.

Analysis of the NOESY spectrum revealed weak correlations from H1 and H8 to resonances at 8.25 ppm and 8.31 ppm, assigned as H4 and H11, respectively (Figure S77, magenta, green). The positions of the allyl substituents was confirmed by the observation of correlations between H1 and H1''' (teal), H4 and H6 (red), and H11 and H1'' (purple) in the NOESY spectrum (Figure S77). Despite the lack of a HMBC correlation between H6 and C7 which would be expected, the presence of this *O*-cyclised derivative was deduced based upon the observations that: i) H6 was a methine proton, suggesting the replacement of a proton with another functionality; ii) the deshielded nature of C6 and H6, which would imply the attachment of multiple heteroatoms at this position; and iii) the carbonyl moiety which would otherwise be present at the C7 position is missing, suggesting a chemical transformation has occurred. Analysis of the HRMS (ESI$^+$) revealed a peak at *m*/*z* 473.1474, confirming the molecular formula $C_{25}H_{21}N_4O_6^+$, assigned as the $[M+H]^+$ ion of **30**.

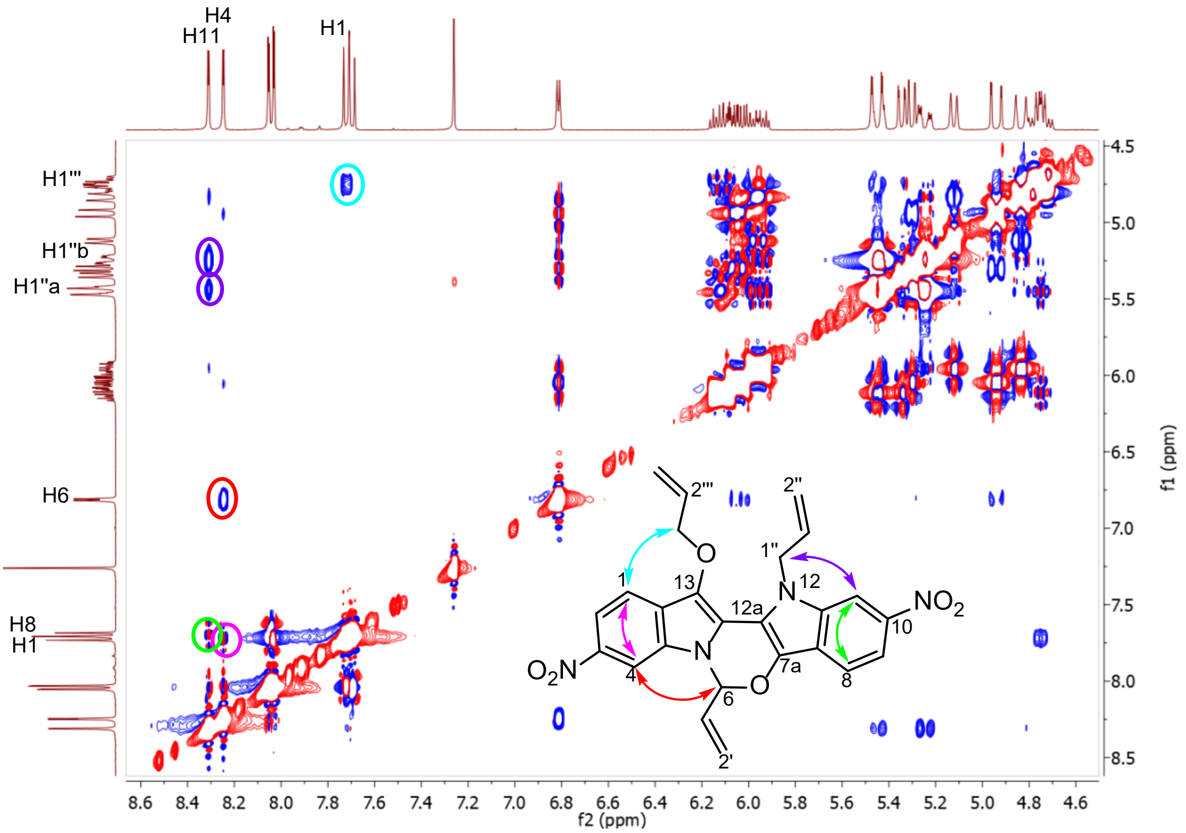

Figure S77: Analysis of the NOESY spectrum of oxazinodiindole **30**.

S4.7 Structural elucidation of *N,N'*-diallyl-3,3'-bis(allyloxy)biindole 31

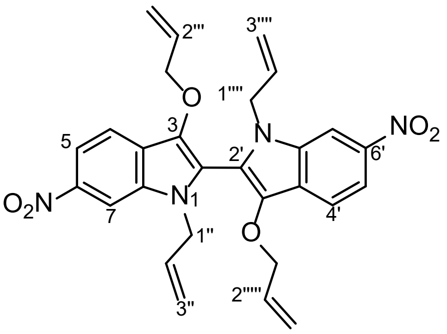

31

Examination of the LRMS (ESI$^+$) of **31** revealed a prominent peak at *m/z* 537, assigned as the [M+Na]$^+$ ion, corresponding to the addition of four allyl units to 6,6'-dinitroindigo **1i**. Analysis of the ^{1}H NMR spectrum revealed the presence of three aromatic resonances and only two sets of allyl moiety resonances, suggesting the presence of a plane of symmetry in **31**. Aromatic proton resonances at 8.37 ppm (dd, J = 2.0, 0.6 Hz), 8.05 ppm (dd, J = 8.8, 2.0 Hz) and 7.79 ppm (dd, J = 8.8, 0.5 Hz) were assigned as H7 & H7', H5 & H5' and H4 & H4', respectively.

Analysis of the HMBC spectrum revealed a strong correlation between H4 & H4' and a carbon resonance at 133.5 ppm, assigned as C7a & C7a', which in turn correlated to proton resonances at 4.78 ppm and 4.73 ppm, assigned as H1''a & H1''''a and H1''b & H1''''b, respectively (Figure S78, blue). A strong correlation was also observed between H4 & H4' and a carbon resonance at 138.2 ppm, assigned as C3 & C3', which correlated to protons at

4.53 ppm and 4.47 ppm, assigned as H1'''a & H1'''''a and H1'''b & H1'''''b, respectively (Figure S78, red). Inspection of the HSQC spectrum revealed strong correlations from H1'' & H1'''' and H1''' & H1''''' to carbon resonances at 47.4 ppm and 74.8 ppm, assigned as C1'' & C1'''' and C1''' & C1''''', respectively. Carbons C1'' & C1'''' exhibited a HMBC correlation to proton resonances at 5.22 ppm and 5.14 ppm, assigned as alkenyl protons H3''a & H3''''a and H3''b & H3''''b, respectively (Figure S78, black). Likewise, C1''' & C1''''' showed correlations to alkenyl protons at 5.11 ppm and 4.92 ppm, assigned as H3'''a & H3'''''a and H3'''b & H3'''''b, respectively (Figure S78, purple). Analysis of the HRMS (ESI$^+$) revealed a peak at *m/z* 537.1735, confirming the molecular formula $C_{28}H_{26}N_4O_6Na^+$, assigned as the $[M+Na]^+$ ion of **31**.

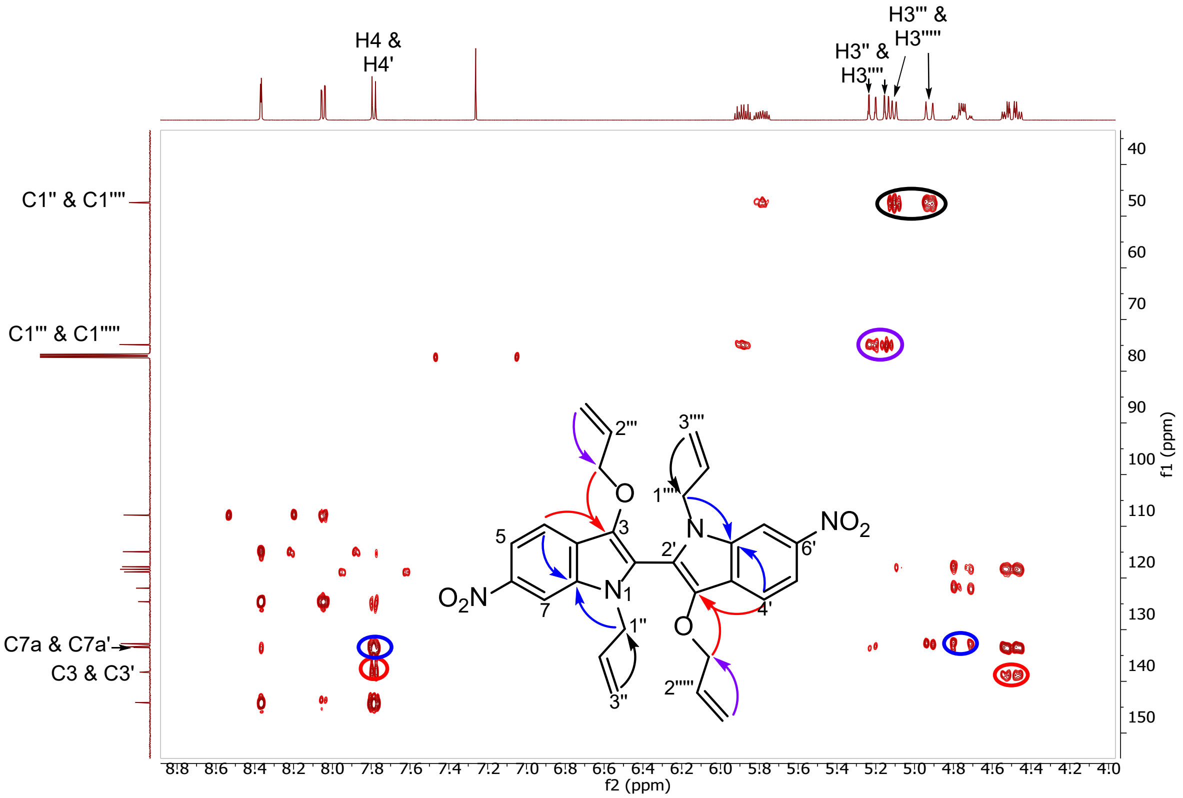

Figure S78: Analysis of the HMBC spectrum of *N*,*N'*-diallyl-3,3'-bis(allyloxy)biindole **31**.

S4.8 Structural elucidation of *transoid C*-allylspiroindolinepyridoindoledione 33b

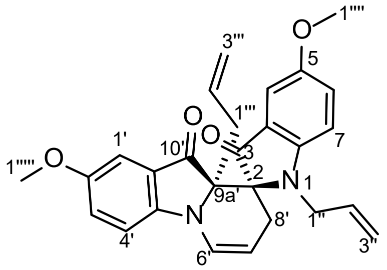

(±)-**33b**

Analysis of the LRMS (ESI$^+$) of **33b** revealed a base peak at *m*/*z* 443, assigned as the [M+H]$^+$ ion. Analysis of the COSY spectrum revealed an aromatic resonance at 7.10 ppm, which exhibited a strong and a weak correlation to a resonance at 6.78 ppm and a multiplet at 6.86 – 6.80 ppm, assigned as H6, H4 and H7, respectively (Figure S79, blue, black). Further examination revealed a resonance at 7.16 ppm exhibited a strong and a weak correlation to resonances at 7.00 ppm and the multiplet at 6.86 – 6.80 ppm, assigned to aromatic protons H3', H4' and H1', respectively (Figure S79, red, purple).

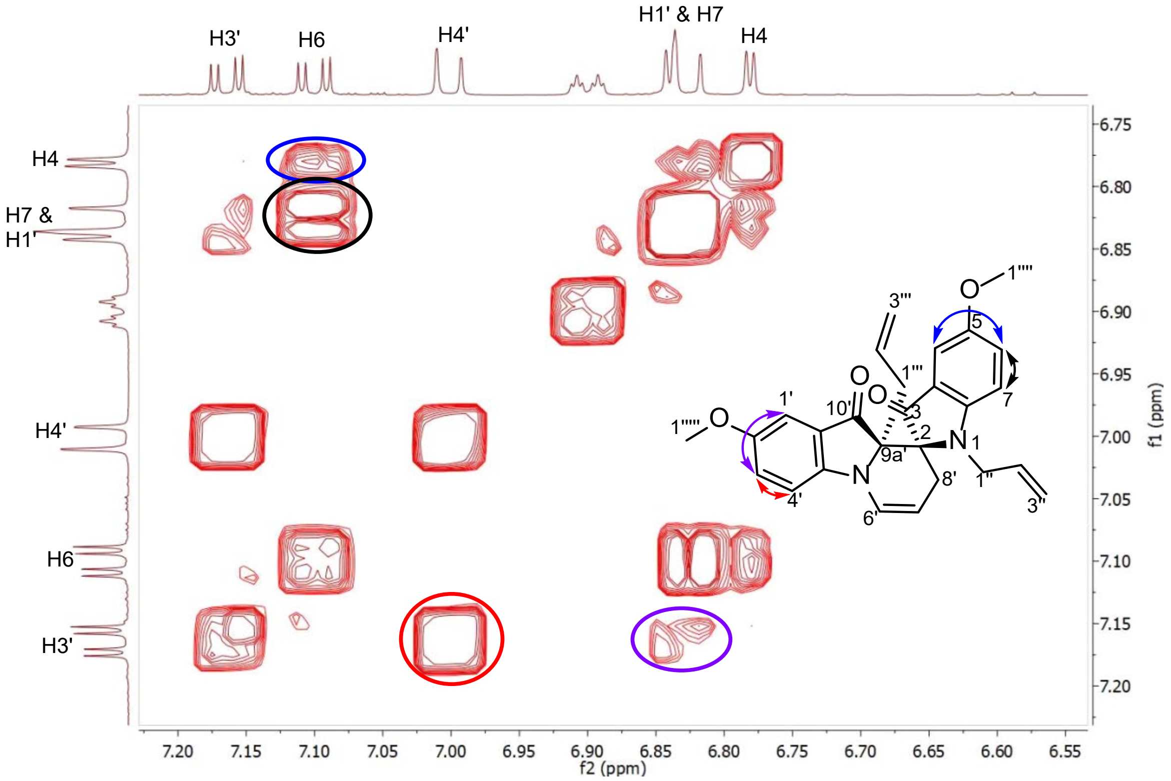

Figure S79: Analysis of the COSY spectrum of *transoid C*-allylspiroindolinepyridoindoledione **33b**.

Analysis of the HMBC spectrum of **33b** showed a strong correlation from protons H4 and H6 to a carbon resonance at 156.6 ppm, assigned as C7a, which correlated to proton resonances at 4.39 ppm and 4.04 ppm, assigned as diastereotopic protons H1''a and H1''b (Figure S80, blue). Proton H4 was also observed to correlate strongly to a deshielded carbon resonance at 197.7 ppm, assigned as carbonyl C3, which also correlated strongly to proton resonances at 2.81 – 2.75 ppm and 2.05 ppm, assigned as H8'a and H8'b (Figure S80, red). Proton H8'

exhibited a strong correlation to a carbon resonance at 69.5 ppm, assigned as C9a', which also correlated strongly to a proton resonance at 6.90 ppm, assigned as H6' (Figure S80, magenta). Examination of the HSQC spectrum revealed a correlation between H8' and a carbon resonance at 29.9 ppm, assigned as C8', which also exhibited a strong HMBC correlation to H6' (Figure S80, black). Proton H1' exhibited a strong HMBC correlation to a carbon resonance at 197.4 ppm, assigned as carbonyl C10', which also correlated to proton resonances at 2.81 – 2.75 ppm and 2.66 ppm, assigned as H1'''a and H1'''b (Figure S80, teal). A carbon resonance at 69.1 ppm was observed to correlate to protons H1''' and H8', assigned as spirocyclic carbon C2 (Scheme 57, lime). Analysis of the HSQC spectrum revealed strong correlations between protons H1'' and H1''' and carbon resonances at 47.5 ppm and 37.4 ppm, assigned as C1'' and C1''', respectively. Further analysis of the HMBC spectrum revealed a strong correlation between C1'' and proton resonances at 5.65 ppm and 5.41 – 5.28 ppm, assigned as terminal alkenyl protons H3''a and H3''b (Figure S80, orange). Carbon C1''' also exhibited strong correlations to alkenyl protons at 5.06 – 5.00 ppm and 4.88 ppm, assigned as protons H3'''a and H3'''b (Figure S80, purple). Analysis of the HRMS (ESI$^+$) revealed a peak at *m/z* 465.1784, confirming the molecular formula $C_{27}H_{26}N_2O_4Na^+$, assigned as the [M+Na]$^+$ ion of **33b**.

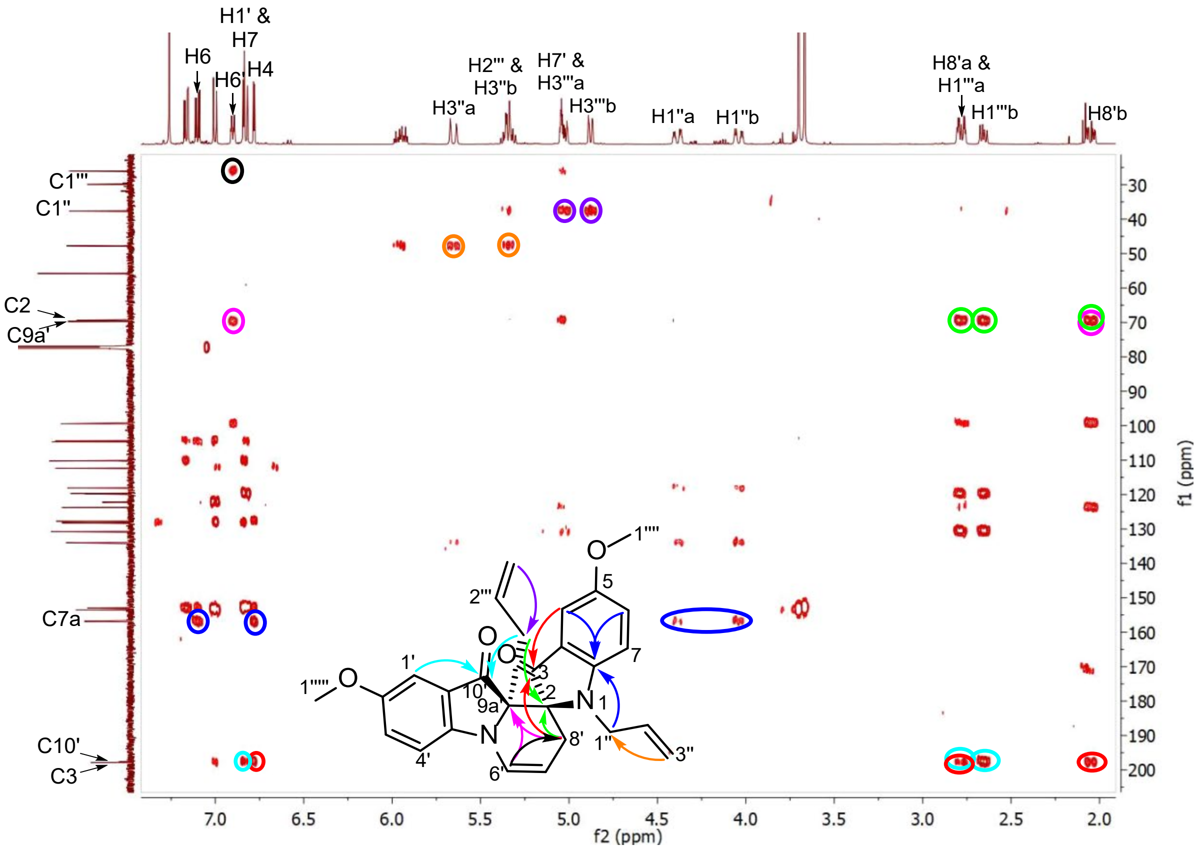

Figure S80: Analysis of the HMBC spectrum of *transoid C*-allylspiroindolinepyridoindoledione **33b**.

S4.9 Assignment of *C*-allylspiroindolinepyridoindoledione 33a-b relative stereochemistry

To assign the relative stereochemistry of the *cisoid* and *transoid* *C*-allylspiroindolinepyridoindoledione **33a**-**b**, analysis of the NOESY spectra was attempted, however this was inconclusive due to multiplet resonances hindering the clear assignment of key correlations. Analysis of the ^{1}H NMR chemical shifts of allyl methylene moieties was identified as one indicator of relative stereochemistry as wider peak splittings were expected in the *cisoid* isomer due to extra steric hindrance caused by clashing allyl moieties. Analysis of the ^{1}H NMR spectrum of the major *C*-allylspiroindolinepyridoindoledione isomer **33a** revealed peak splittings of 0.35 ppm, 0.55 ppm and 0.84 ppm for methylene protons H1'', H8' and H3''', respectively, while these protons in minor isomer **33b** showed peak splittings of 0.35 ppm, 0.13 ppm and 0.73 ppm (Figure S81a-b). The significantly reduced peak splitting observed for protons H1''' in **33b** suggest rotation is less restricted than in the case of **33a**, and therefore that **219b** possesses *transoid* relative stereochemistry.

Further analysis of the ^{1}H NMR spectrum of **33b** revealed that the *N*-allyl substituent exhibited significantly deshielded chemical shifts relative to **33a**, particularly the alkenyl protons H2'' and H3''a and H3''b, which were observed at 5.95 ppm, 5.65 ppm and 5.41 – 5.28 ppm, respectively, in **33b** compared to 5.39 – 5.14 ppm (H2'') and 4.91 – 4.78 ppm (H3'') in **33a** (Figure S81c). Analysis of the 3D structures of the relative stereoisomers of **33** reveal that in the *transoid* form, the *N*-allyl (teal) substituent is located adjacent to the pyridoindolone phenyl moiety (yellow), while in the *cisoid* form, these substituents are oriented in opposite directions (Figure S82). As a measure of the difference in distance between the *N*-allyl substituent and the pyridoindolone phenyl moieties in each isomer, the distance between methylene C1'' and bridgehead C10a' was measured, equalling 5.2 Å and 3.9 Å in the *cisoid* and *transoid* forms, respectively (Figure S82). The closer proximity of the *N*-allyl substituent to the pyridoindolone phenyl moiety in the *transoid* could cause the deshielding observed in **33b**, suggesting that **33a** is the *cisoid* isomer and **33b** is the *transoid* isomer.

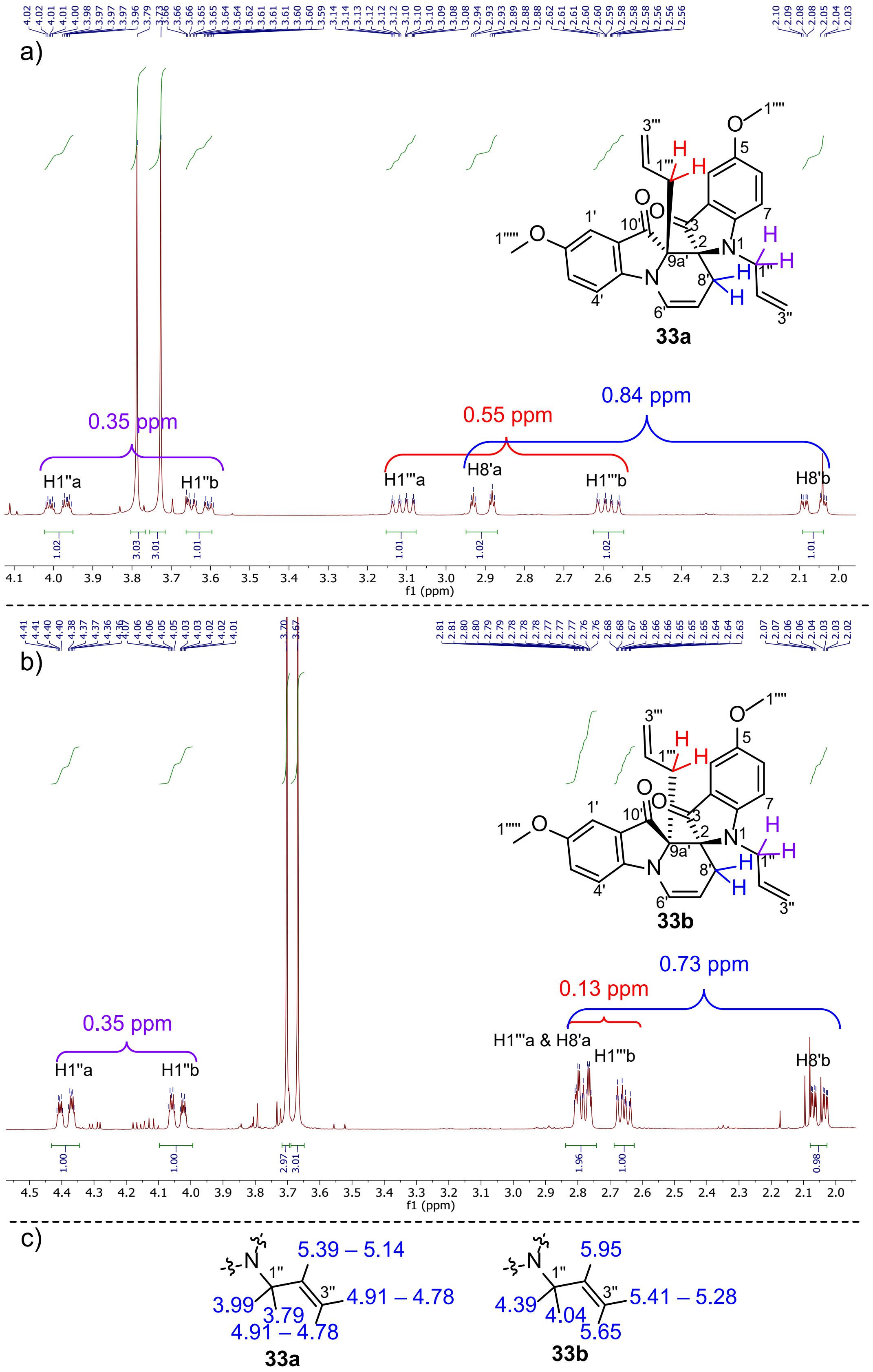

Figure S81: Analysis of the methylene peak splittings in the ^{1}H NMR spectra of spiroindolinepyridoindoledione a) major isomer **33a** (400 MHz, $CDCl_3$) and b) minor isomer **33b** (500 MHz, $CDCl_3$). c) A comparison of the ^{1}H NMR chemical shifts (ppm, blue) observed in *N*-allyl substituents of **33a**-**b**.

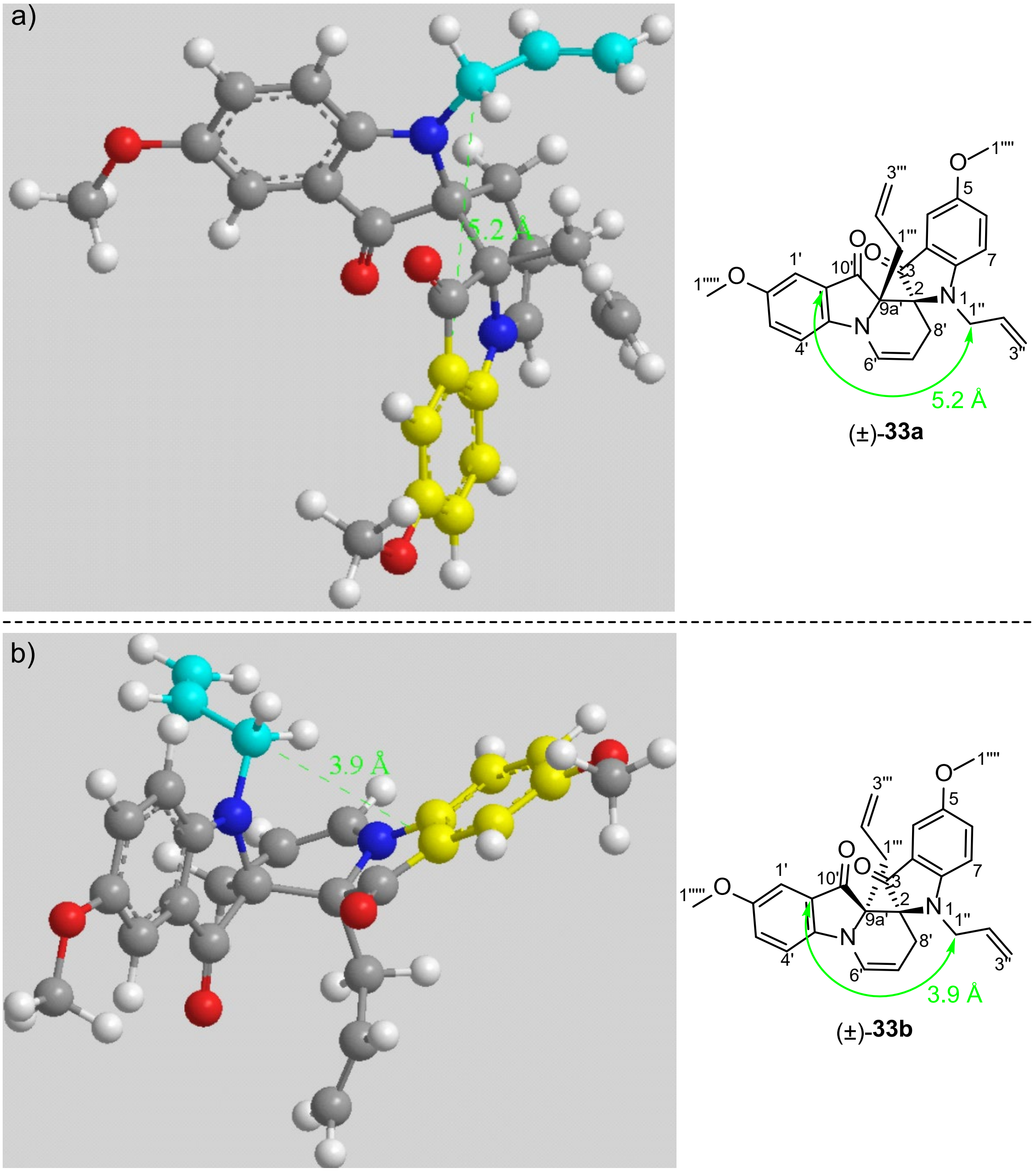

Figure S82: Representation of the 3D structures of *C*-allylspiroindolinepyridoindoledione **33** in its a) *cisoid* **33a** and b) *transoid* **33b** forms, with the *N*-allyl (teal) and pyridoindolone phenyl (yellow) moieties highlighted and the distance between C1'' and C10a' displayed (green). Structure optimisations were accomplished using an MM2 force field.

S5. X-Ray Crystal Data of Spiroindolinepyridoindolone 22

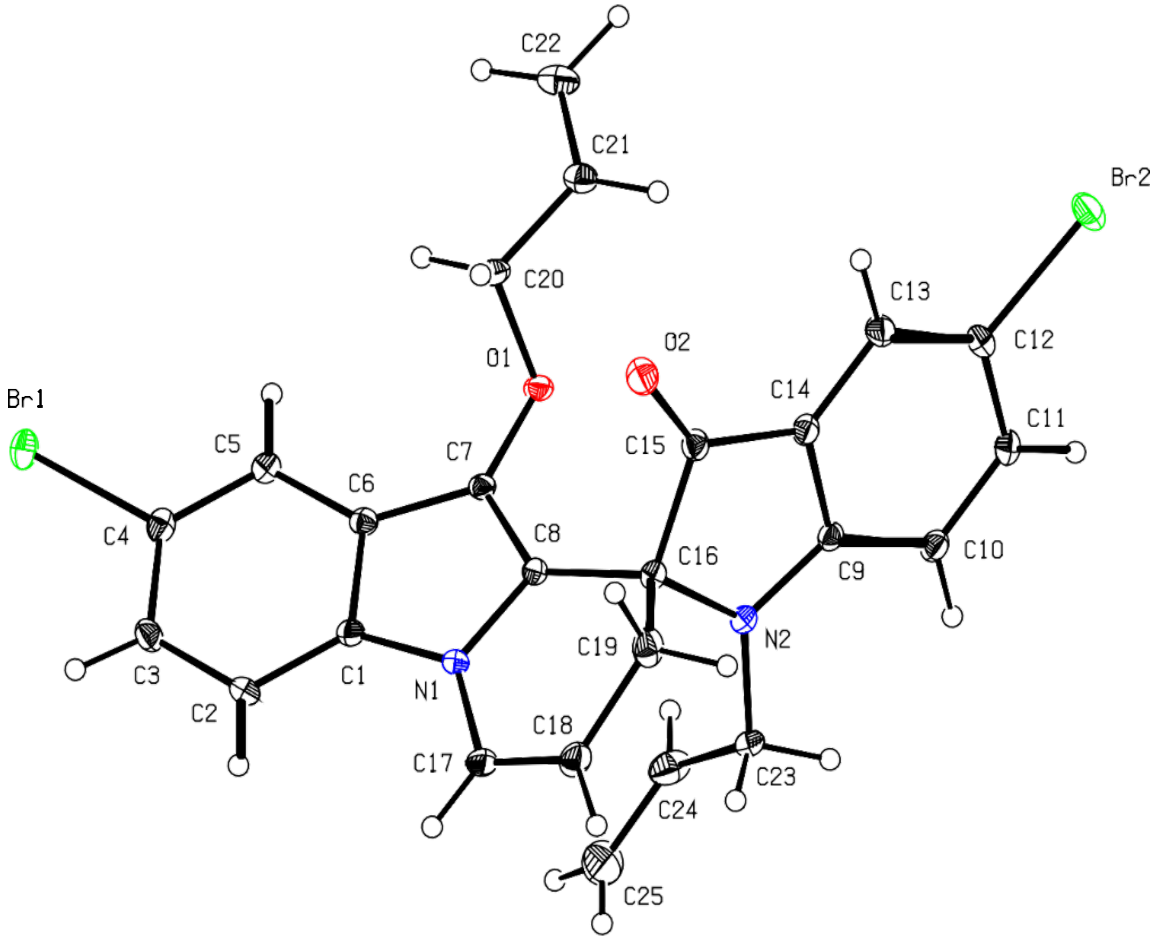

Figure S83: Spiroindolinepyridoindolone **22**, $C_{25}H_{20}Br_2N_2O_2$. Mr = 540.25, T = 150 K, Orthorhombic, space group-*Pbca*, Z = 8, a = 9.36746 (4), b = 18.28656 (9), c = 25.06240 (12) Å, V = 4293.15 (3) Å^3, D_X = 1.672 g.cm^{-3}, Cu $K\alpha$ radiation, λ = 1.54184 Å, 47329 reflections measured (q_{range} = 4 – 74°), merged to 4349 unique data, R = 0.027 [for 4249 data with $I > 2\sigma(I)$], R_W = 0.056 [all data], S = 1.03. CCDC No. 2219386.

S6. References

1. W.-Y. Li, X.-Q. Xiong, D.-M. Zhao, Y.-F. Shi, Z.-H. Yang, C. Yu, P.-W. Fan, M.-S. Cheng and J.-K. Shen, *Molecules* 2012, **17**, 5497-5507.
2. M. Ibrahimi, O. Khoumeri, R. Abderrahim, T. Terme and P. Vanelle, *Synlett*, 2021, **32**, 283-286.
3. I. V. Klimovich, A. V. Zhilenkov, L. I. Kuznetsova, L. A. Frolova, O. R. Yamilova, S. I. Troyanov, K. A. Lyssenko and P. A. Troshin, *Dyes Pigm.*, 2021, **186**, 108966.
4. W. Xiong, S. Li, B. Fu, J. Wang, Q.-A. Wang and W. Yang, *Org. Lett.*, 2019, **21**, 4173-4176.
5. H. Dinçalp, G. M. Saltan, C. Zafer and D. A. Kiymaz, *J. Mol. Struct.*, 2018, **1173**, 512-520.
6. F. P. Guengerich, J. L. Sorrells, S. Schmitt, J. A. Krauser, P. Aryal and L. Meijer, *J. Med. Chem.*, 2004, **47**, 3236-3241.
7. J. A. Shriver, K. R. Wang, A. C. Patterson, J. R. DeYoung and R. J. Lipsius, *RSC Adv.*, 2020, **10**, 36849-36852.
8. T. Kobayashi and P. M. Rentzepis, *J. Chem. Phys.*, 1979, **70**, 886-892.
9. B. Koeppe and V. R. F. Schroeder, *ChemPhotoChem*, 2019, **3**, 613-618.
10. R. Rondao, J. S. Seixas de Melo and G. Voss, *ChemPhysChem*, 2010, **11**, 1903-1908.
11. P. W. Sadler, *J. Org. Chem.*, 1956, **21**, 316-318.
12. J. S. Seixas de Melo, R. Rondao, H. D. Burrows, M. J. Melo, S. Navaratnam, R. Edge and G. Voss, *ChemPhysChem*, 2006, **7**, 2303-2311.
13. M. I. G. Travasso, P. Santos, A. M. Campos, M. M. M. Raposo and N. Prasitpan, *Adv. Colour Sci. Technol.* , 2003, **6**, 95-99.
14. O. Pitayatanakul, T. Higashino, T. Kadoya, M. Tanaka, H. Kojima, M. Ashizawa, T. Kawamoto, H. Matsumoto, K. Ishikawa and T. Mori, *J. Mater. Chem. C*, 2014, **2**, 9311-9317.
15. E. Ziegler, H. Foraita and T. Kappe, *Monatsh. Chem.*, 1967, **98**, 324-328.
16. Y. Tanoue, A. Terada, K. Sakata, M. Hashimoto, S.-I. Morishita, M. Hamada, N. Kai and T. Nagai, *Fish. Sci.*, 2001, **67**, 726-729.
17. P. Imming, I. Imhof and M. Zentgraf, *Synth. Commun.*, 2001, **31**, 3721-3727.
18. K. Liu, P. Wen, J. Liu and G. Huang, *Synthesis*, 2010, **2010**, 3623-3626.
19. C. Ramesh, G. Mahender, N. Ravindranath and B. Das, *Tetrahedron*, 2003, **59**, 1049-1054.
20. S. Namgung, H. A. Park, J. Kim, P.-G. Lee, B.-G. Kim, Y.-H. Yang and K.-Y. Choi, *Dyes Pigm.*, 2019, **162**, 80-88.
21. A. Rioz-Martinez, M. Kopacz, G. de Gonzalo, D. E. Torres Pazmino, V. Gotor and M. W. Fraaije, *Org. Biomol. Chem.*, 2011, **9**, 1337-1341.
22. X. Zhang, Y. Qu, Q. Ma, H. Zhou, X. Li, C. Kong and J. Zhou, *Process Biochem.*, 2013, **48**, 581-587.
23. Z. L. Wu, P. Aryal, O. Lozach, L. Meijer and F. P. Guengerich, *Chem. Biodivers.*, 2005, **2**, 51-65.
24. D. A. Davis, H. L. Wasserkrug, I. A. Heyman, K. Padmanabhan, G. A. Seligman, R. E. Plapinger and A. M. Seligman, *J. Histochem. Cytochem.*, 1972, **20**, 161-172.
